# A Redox-Relay Heck Approach to Substituted Tetrahydrofurans

**DOI:** 10.1021/acs.orglett.3c00769

**Published:** 2023-03-29

**Authors:** Tom J. M. Byrne, Megan E. Mylrea, James D. Cuthbertson

**Affiliations:** † GlaxoSmithKline Carbon Neutral Laboratories for Sustainable Chemistry, University of Nottingham, Jubilee Campus, Triumph Road, Nottingham NG7 2TU, U.K.; ‡ School of Chemistry, University of Nottingham, University Park, Nottingham NG7 2RD, U.K.

## Abstract

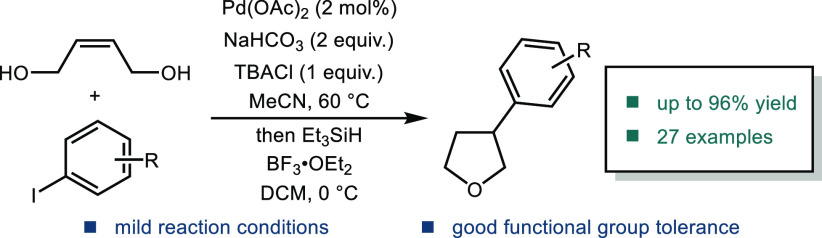

An operationally simple and efficient strategy for the synthesis of substituted tetrahydrofurans from readily available *cis*-butene-1,4-diol is described. A redox-relay Heck reaction is used to rapidly access cyclic hemiacetals that can be directly reduced to afford the corresponding 3-aryl tetrahydrofuran. Furthermore, the hemiacetals can also serve as precursors to a range of disubstituted tetrahydrofurans, including the calyxolane natural products.

Heterocycles are prevalent in a myriad of molecules essential to society. The tetrahydrofuran motif is of particular importance with a 2014 survey identifying it as the 11th most common ring system in a study of known pharmaceuticals.^1^ Furthermore, tetrahydrofurans are found in a range of structurally diverse bioactive natural product classes.^2−5^ One important class of tetrahydrofurans consists of those with an aryl or heteroaryl group at position 3 or 4, a motif that is found in molecules such as magnosalicin **1**,^6^ calyxolanes A/B **2**,^7^ and the BACE1 inhibitor, LY2886721 **3** (Figure 1).^8^

**Figure 1:**
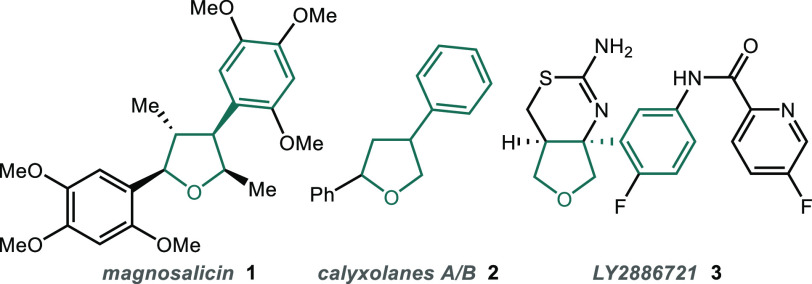
Molecules featuring an aryl-substituted tetrahydrofuran ring.

Given their importance, a range of methods have been developed to synthesize 3- or 4-aryl tetrahydrofurans, including cyclization of prefunctionalized diols or their derivatives, cyclization of prefunctionalized alkenols, and reduction of substituted furans/dihydrofurans (Figure 2a).^9,10^ However, despite their unquestionable value, these approaches often lead to products that also bear substituents at the positions adjacent to oxygen. Accessing the corresponding 3-aryl tetrahydrofurans in which positions 2 and 5 are unsubstituted in a concise manner can often present a greater synthetic challenge. While such compounds can be accessed through a range of C(sp^2^)–C(sp^3^) cross-coupling reactions,^11^ the requisite coupling partners can be expensive or have limited commercial availability. As a result, there remains a need for broadly applicable methods that enable the preparation of 3-aryl tetrahydrofurans from readily available and inexpensive precursors.

**Figure 2:**
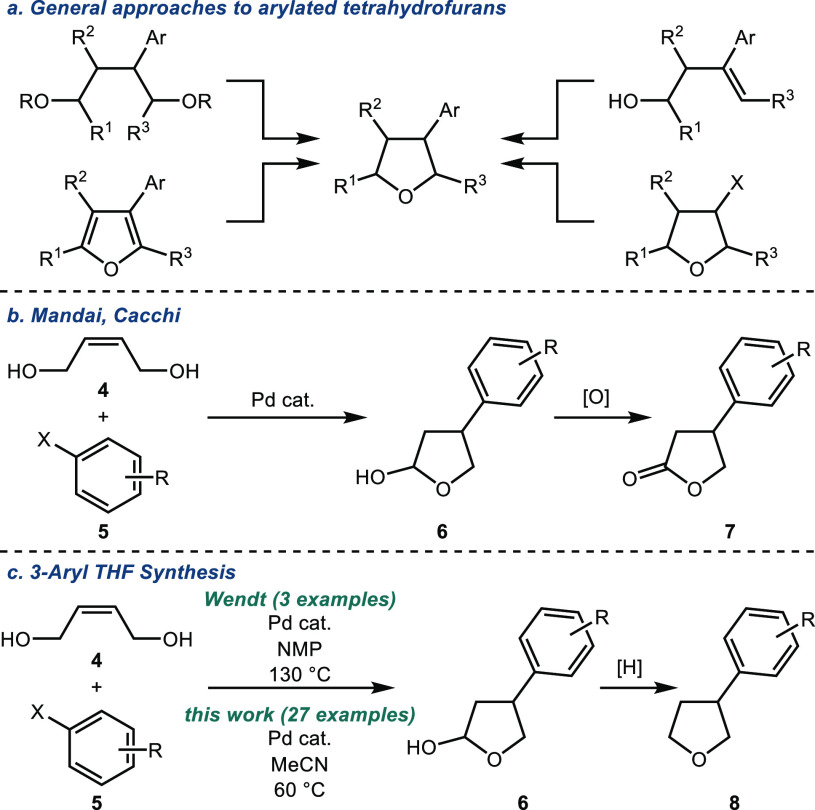
Approaches to arylated tetrahydrofuran derivatives.

An alternative approach to the tetrahydrofuran core was reported independently by the groups of Mandai and Cacchi,^12,13^ who exploited the propensity of allylic alcohols to undergo arylation by a Heck reaction to afford β-aryl aldehydes via a redox isomerization event.^14,15^ In both cases, arylation of *cis*-butene-1,4-diol (**4**) gave hemiacetals **6** that were subsequently oxidized to afford the corresponding lactones **7** (Figure 2b). If instead, hemiacetal **6** was reduced, this would provide a straightforward method for accessing pharmaceutically relevant 3-aryl tetrahydrofurans **8** (Figure 2c). This concept was demonstrated by Wendt and co-workers, who reported the arylation of *cis*-butene-1,4-diol (**4**) followed by reduction of the resulting hemiacetal to prepare 3-substituted tetrahydrofurans.^16^ However, the reactions were performed in a hazardous solvent (NMP),^17^ at high temperatures (130 °C), and the scope was limited to three examples, highlighting the need for further work to deliver a broadly applicable protocol.

Herein, we report the development of a general strategy for the assembly of 3-aryl tetrahydrofurans from an inexpensive, commercially available, precursor. Structurally diverse aryl iodides **5** are coupled with *cis*-2-butene-1,4-diol (**4**) in a redox-relay Heck reaction to afford hemiacetals **6** that, without purification, can be reduced to afford the corresponding tetrahydrofuran **8**. Furthermore, hemiacetal **6** can serve as a precursor to other useful building blocks and the calyxolane natural products. The reactions proceed under mild conditions and tolerate diverse functionality, including esters, carbamates, nitriles, halides, and unprotected alcohols and/or phenols.

We began our investigations by studying the coupling of *cis*-2-butene-1,4-diol (**4**) and methyl 4-iodobenzoate (**5a**). Pleasingly, following optimization, we found that hemiacetal **6a** could be formed in excellent yield in an operationally simple process that did not require an inert atmosphere (Table 1, entry 1). Interestingly, traces of a regioisomeric (2,3-substituted) hemiacetal were also observed in the ^1^H NMR spectrum of the unpurified reaction mixture, which was presumably generated in the redox-relay Heck reaction via a series of β-hydride elimination/migratory insertion steps.^18,19^ However, this was inconsequential for the methodology as both regioisomers would subsequently be reduced to give the desired 3-aryl tetrahydrofuran.

**Table 1:**
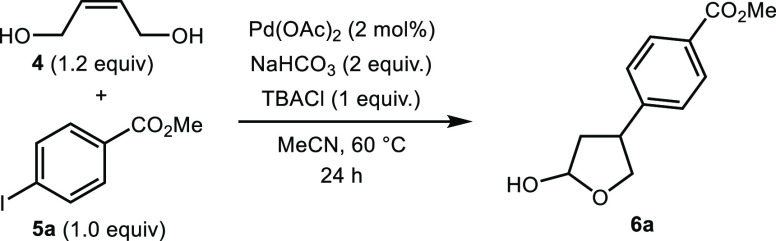
Optimization Studies and Control Reactions^a^

entry	deviation from optimized conditions	yield (%)^b^,^c^
1	none	96
2	DMF instead of MeCN	94
3	THF instead of MeCN	88
4	2-MeTHF instead of MeCN	86
5	MeCN/H_2_O (1:1) instead of MeCN	48
6	6 h reaction time	77
7	16 h reaction time	88
8	no TBACl	53
9	TBACl (0.5 equiv)	63
10	Ar atmosphere	92
11	*trans*-2-buten-1,4-diol^d^	61
12	ArBr/ArOTf instead of ArI	37/0

a Reactions performed on a 0.5 mmol scale in MeCN ([**5a**]_0_ = 0.4 M).

b Yields were determined by ^1^H NMR spectroscopy using 1,3-benzodioxole as an internal standard.

c dr ∼1.7:1.

d Reaction performed using 1.0 mmol of aryl iodide **5a**.

Pleasingly, a range of solvents could be employed with only marginal decreases in the yield (Table 1, entries 2–4). Furthermore, an aqueous solvent system was also tolerated, giving the product in 48% yield (Table 1, entry 5). A time study showed that a 24 h reaction time was required to achieve maximum conversion. However, good yields of the product could still be obtained if the reaction time was reduced (Table 1, entries 6 and 7). Tetrabutylammonium salts have been shown to have a beneficial effect in Heck reactions.^20−22^ In our studies, the reaction was found to proceed in the absence of tetrabutylammonium chloride (TBACl), albeit in lower yield (Table 1, entry 8). Attempts to reduce the loading of TBACl also resulted in a decreased yield of the product (Table 1, entry 9). The robustness of the methodology was highlighted by the fact that a reaction performed under an argon atmosphere showed no improvement in yield, demonstrating that the chemistry is tolerant of air, greatly simplifying the reaction setup (Table 1, entry 10). Interestingly, switching the geometry of the double bond in the substrate was found to have a deleterious effect on the reaction, giving the product in a reduced 61% yield (Table 1, entry 11). Finally, we explored the use of aryl bromides and aryl triflates in the reaction. While methyl 4-bromobenzoate was found to be a viable coupling partner, no product was obtained with the corresponding triflate (Table 1, entry 12).

Having optimized the redox-relay Heck reaction, we next sought to identify conditions for the reduction of the hemiacetal. Attempts to reduce hemiacetal **6a** by direct addition of a reductant/Lewis acid at the end of the redox-relay Heck reaction proved to be unsuccessful. However, purified hemiacetal **6a** could be cleanly reduced using triethylsilane in the presence of boron trifluoride diethyl etherate to afford tetrahydrofuran **8a** in quantitative yield.^16,23^ Pleasingly, it was subsequently found that similar yields (>95%) were obtained using unpurified hemiacetal **6a**, isolated following an aqueous workup, negating the need to incorporate an intermediate chromatographic purification step.

With optimized conditions in hand, the scope of the sequence was explored (Scheme 1). Pleasingly, ester-substituted aryl iodide **5a** used in optimization studies gave product **8a** in 96% yield when carried out on a 1.00 mmol scale. Furthermore, a range of electronically diverse *para*-substituted aryl iodides gave the products in good to excellent yields (**8b**–**8i**). Halide substituents could also be present on the aromatic ring (**8j** and **8k**), despite the fact that aryl halides serve as coupling partners in a multitude of palladium-mediated processes. It is noteworthy that 4-bromo-iodobenzene reacted selectively at the iodide to give product **8j** in 74% yield. Finally, a 4-iodophenylalanine derivative gave protected amino acid **8l** in excellent yield (dr 1:1). Aryl iodides bearing substituents at the *meta* position were also tolerated giving the products in good to excellent yields (**8m**–**8o**). A substrate featuring both a *m*-bromo and *o*-chloro substituent on the aromatic ring was also a viable coupling partner affording product **8p** in 79% yield. Pleasingly, other *ortho*-substituted iodides could also be employed in the reaction (**8q**–**8u**). It is noteworthy that a protected aniline **8v**, an unprotected alcohol **8w**, and an unprotected phenol **8x** were all tolerated providing useful handles for further functionalization.

**Scheme 1:**
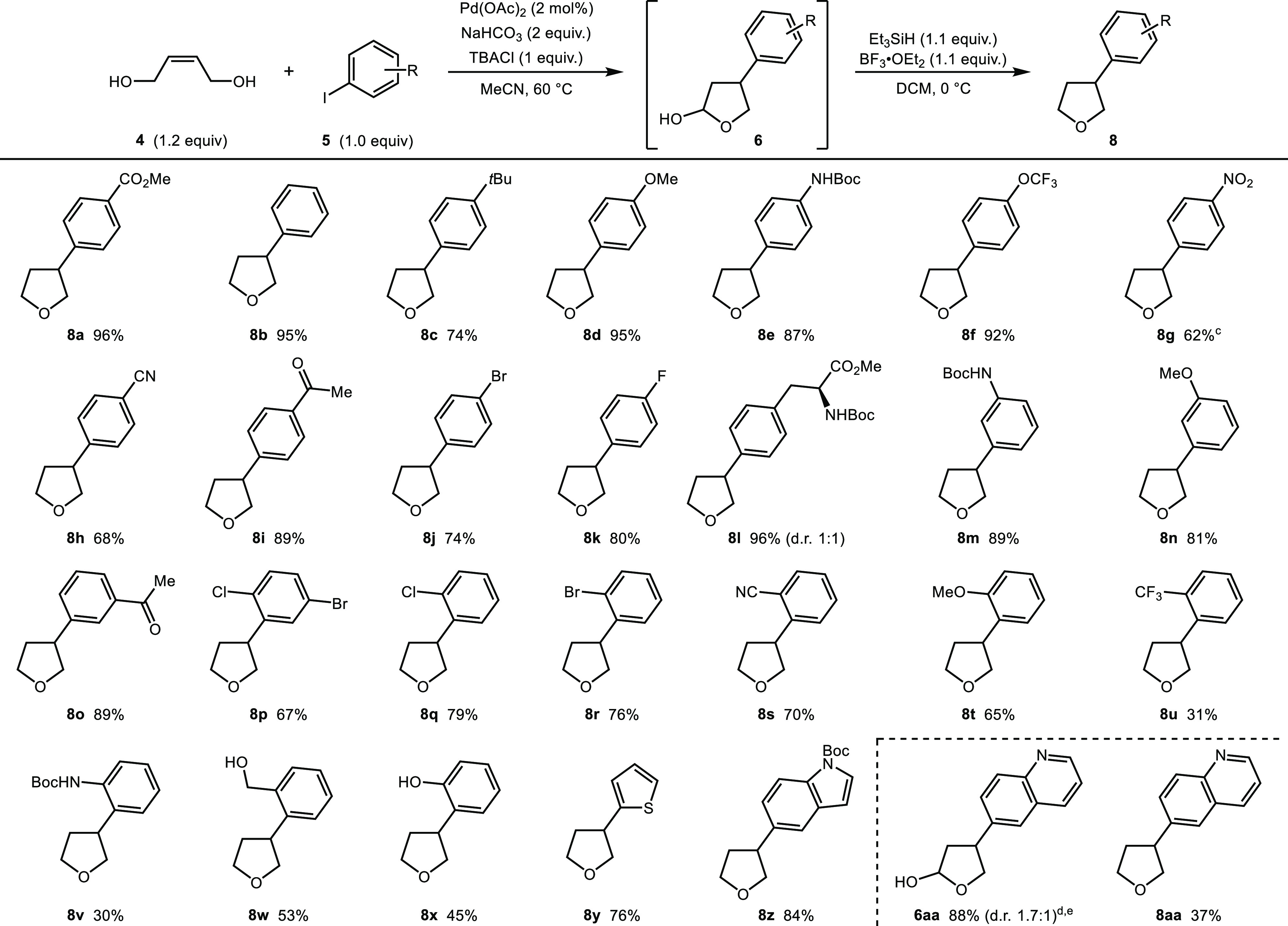
Aryl Iodide Scope, Reactions performed on a 1.0 mmol scale in MeCN ([**5**]_0_ = 0.40 M). Yields refer to material isolated after purification by column chromatography. Hemiacetal **6g** was purified by column chromatography before reduction. The reaction was performed on a 0.59 mmol scale using THF as the solvent. Isolated yield after purification by column chromatography.

Finally, a series of heteroaryl iodides were subjected to the optimized reaction conditions. A thiophene and protected indole derivative gave good yields of the products (**8y** and **8z**). A molecule containing a basic nitrogen performed well in the redox-relay Heck reaction giving hemiacetal **6aa** in excellent yield. Furthermore, synthetically useful quantities of tetrahydrofuran product **8aa** were obtained following reduction under the standard conditions.

While the focus of the study was the development of a straightforward approach to 3-aryl tetrahydrofurans, the same strategy could also be used to access other classes of substituted tetrahydrofuran. A 1,1-disubstituted alkene **9** gave benzylated tetrahydrofuran derivative **10** in 61% yield over the two-step sequence (Scheme 2a). Furthermore, a substituted diol **11** could also be employed in the reaction (Scheme 2b). Using an increased palladium loading (10 mol %) to ensure maximum conversion, the expected *syn*/*anti* 2,4-disubstituted tetrahydrofurans **12** and **13** (dr 4.3:1) were isolated along with regioisomeric *anti*/*syn* 2,3-disubstituted products **14** and **15** (dr 3.4:1) in 77% combined yield.

**Scheme 2:**
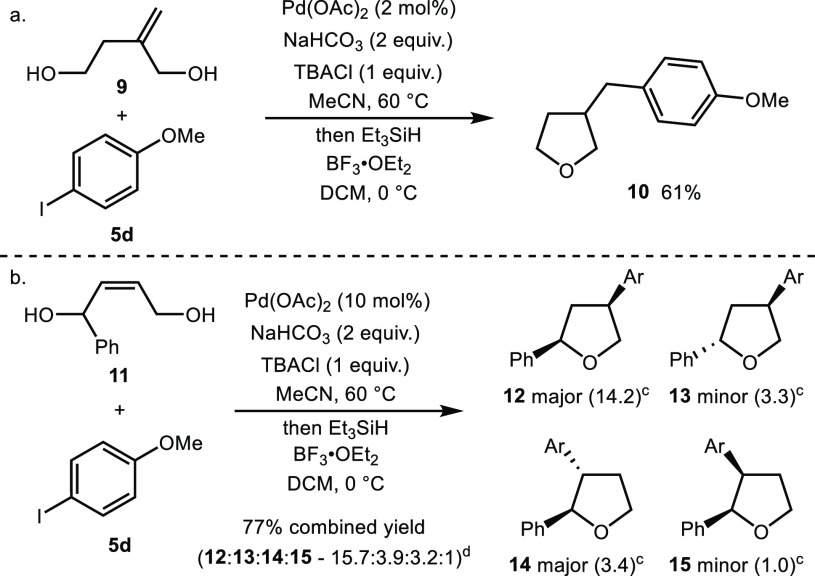
Preliminary Diol Scope Studies, Yields refer to material isolated after purification. See the Supporting Information for full experimental details. Ratios of products determined by ^1^H NMR spectroscopic analysis of the unpurified reaction mixture. Ratio of products after purification by flash column chromatography.

In addition to 3-substituted tetrahydrofurans, hemiacetal **6** could also be converted into a variety of other products, broadening the utility of the methodology (Scheme 3a). Pleasingly, the redox-relay Heck reaction proved to be scalable, affording hemiacetal **6b** in 98% yield when carried out on a 12.2 mmol scale providing material for the derivatization studies.

**Scheme 3:**
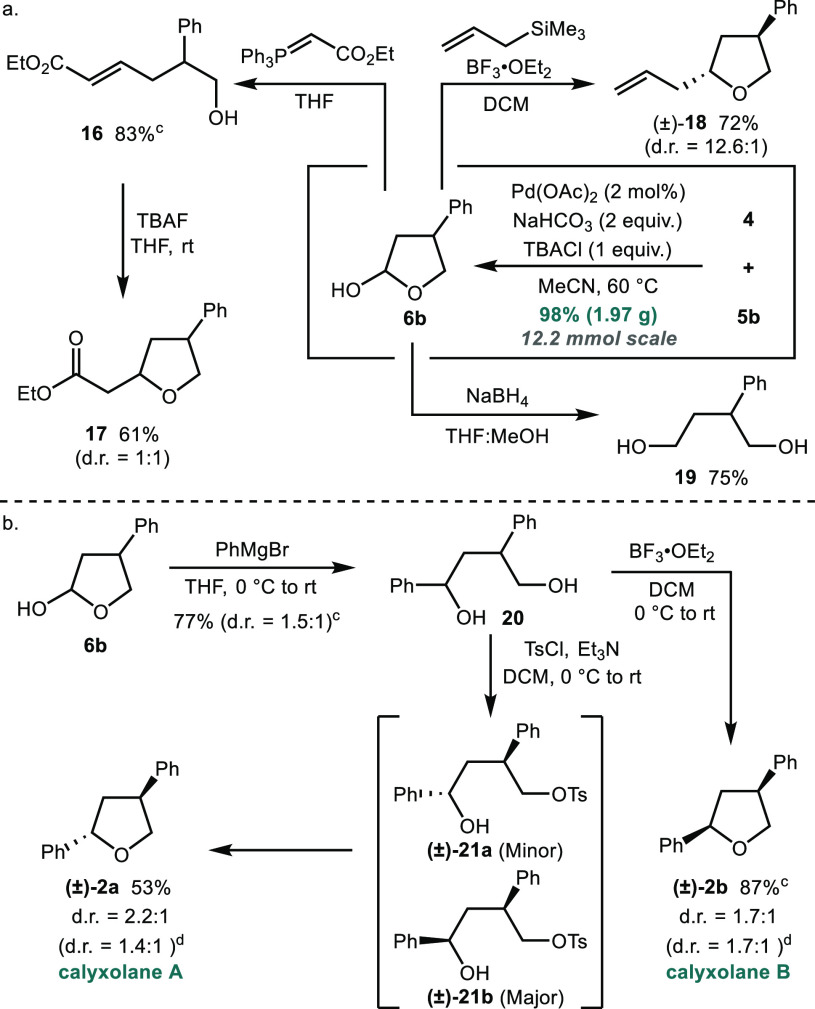
Derivatization of Hemiacetal **6b**, Yields refer to material isolated after purification. See the Supporting Information for full experimental details. Traces of a regioisomeric product derived from a 2,3-substituted hemiacetal were observed in the ^1^H NMR spectrum of the purified product. Diastereomeric ratios in parentheses were determined by ^1^H NMR spectroscopic analysis of the unpurified reaction mixture.

A Wittig reaction using (carbethoxymethylene)triphenylphosphorane gave unsaturated ester **16** in 83% yield, which, upon treatment with tetrabutylammonium fluoride, cyclized to afford disubstituted tetrahydrofuran **17** in 61% yield (dr 1:1). Lewis acid-mediated addition of allyltrimethylsilane gave 2,4-disubstituted tetrahydrofuran **18** in good yield and with high selectivity for the *anti* diastereoisomer (dr 12.6:1).^24^ Furthermore, reduction of hemiacetal **6b** with sodium borohydride gave substituted diol **19** in 75% yield.

Finally, the applications of the chemistry were demonstrated through the total synthesis of the natural products calyxolanes A and B (Scheme 3b).^7^ Addition of phenylmagnesium bromide to hemiacetal **6b** gave alcohol **20** in 77% yield as a mixture of diastereoisomers (dr 1.5:1). Treatment of diastereomeric diols **20** with boron trifluoride diethyl etherate gave calyxolane B **2b** as the major product in 87% combined yield (dr 1.7:1).^25^ The relative stereochemistry of the major product was confirmed to be that of calyxolane B **2b** by comparison of the ^1^H NMR spectroscopic data with those previously reported.^7,25^ Alternatively, the natural products could be accessed via tosylation of the primary alcohol in diol **20** to give diastereomeric tosylates **21a** and **21b** that cyclized *in situ*.^26^ Interestingly, tosylate **21a** formed from the minor diastereoisomer of diol **20** was found to cyclize at a higher rate, resulting in a sample enriched with calyxolane A **2a**. Quantities of tosylate **21b** formed from the major diastereoisomer of diol **20** could be recovered even after extended reaction times. This straightforward approach to the calyxolane natural products should enable analogues to be rapidly accessed by simply substituting the aryl iodide and Grignard reagents used in the synthesis.

In summary, an efficient strategy for the synthesis of substituted tetrahydrofurans has been developed. The scope and limitations of the methodology have been evaluated, and the applications of the chemistry in the synthesis of the calyxolane natural products have been described. Studies exploring the use of redox-relay Heck reactions in the synthesis of other heterocyclic scaffolds are currently underway in our laboratory.

## Data Availability

The data underlying this study are available in the published article and its online Supporting Information.

## Supporting Information Available

Experimental details, characterization data, and spectra (PDF)
