# Supplementary material for: A Redox-Relay Heck Approach to Substituted Tetrahydrofurans
Source: Org Lett. 2023 Mar 29;25(13):2361–5. doi: 10.1021/acs.orglett.3c00769 (PMC10088021; doi:10.1021/acs.orglett.3c00769)

Redox-Relay Heck Approach to Substituted Tetrahydrofurans

Tom J. M. Byrne,[a,b] Megan E. Mylrea,[a,b] James D. Cuthbertson*[a,b]

[a] *GlaxoSmithKline Carbon Neutral Laboratories for Sustainable Chemistry, University of Nottingham, Jubilee Campus, Triumph Road, Nottingham, NG7 2TU, U.K.*

[b] *School of Chemistry, University of Nottingham, University Park, Nottingham NG7 2RD, U.K.*

*Email: james.cuthbertson@nottingham.ac.uk

Supplementary Information

Contents

1. General Information S2

2. General Procedures S3

3. Diol Substrates S4

4. Aryl Halides and Pseudohalides S6

5. Optimization Studies (Table 1) S9

6. Synthesis of 3-Substituted Tetrahydrofurans S10

7. Diol Scope Studies: S27

8. Synthesis and Derivatization of Hemiacetal **6b**: S30

9. References: S36

10. Copies of ^{1}H NMR, ^{13}C NMR, and ^{19}F NMR Spectra: S38

1. General Information

All reagents and solvents used were of laboratory grade and were used as received without further purification, including all anhydrous solvents. All reactions were carried with magnetic stirring. TLC was performed on Merck TLC Al foils $60F_{254}$ precoated plates and was visualised by exposure to ultraviolet (UV) light (254 nm) or by dipping the plates into a solution of $KMnO_4$ or vanillin followed by gentle heating. Flash column chromatography was carried out using silica gel (Merck Supelco 60 Å particle size 40–63 µm) and the specified eluent. Petrol refers to the fraction of petroleum ether that boils in the range 40–60 °C. High-resolution electrospray ionization time-of-flight (ESI-TOF) mass spectra were recorded using a Bruker MicroTOF II mass spectrometer. High-resolution electron impact ionization (EI) mass spectra were recorded using a JEOL AccuTOF-GCx mass spectrometer. Melting points were recorded on a Stuart SMP20 melting point apparatus and are uncorrected. Infra-red (IR) spectra were recorded on a Bruker platinum alpha FTIR instrument on the neat compound using the attenuated total refraction technique. ^{1}H NMR, ^{13}C NMR and ^{19}F NMR spectra were acquired on Bruker Avance III 400HD or Avance III 500HD spectrometers. Chemical shifts (δ) are reported in parts per million (ppm); coupling constants (*J*) are quoted in Hertz (Hz) and are reported to the nearest 0.1 Hz. ^{1}H and ^{13}C chemical shifts are referenced to the appropriate residual solvent peaks (^{1}H NMR: $CDCl_3$ at 7.26 ppm; ^{13}C NMR: $CDCl_3$ at 77.0 ppm). ^{1}H NMR spectral data are reported as follows; chemical shift, number of protons, multiplicity and coupling constant. The following abbreviations (and combinations of these abbreviations) are used to label multiplicities: s (singlet), d (doublet), t (triplet), q (quartet), quint (quintet), m (multiplet), br (broad), and app (apparent). HSQC, DEPT, NOESY and COSY experiments were used to assist structural assignments.

2. General Procedures

General procedure A – Redox-Relay Heck Reaction:

To a microwave vial containing a mixture of $NaHCO_3$ (2.00 equiv.), $Pd(OAc)_2$ (0.02 equiv.), $^{n}Bu_4NCl$ (1.00 equiv.) and aryl iodide (1.00 equiv.) in MeCN (0.40 M) was added diol (1.20 equiv.) at r.t, the vial was capped, then the reaction was heated to 60 °C in a heating block and stirred for 24 h. The reaction mixture was cooled to r.t and diluted with Et_2O and water, then the aqueous phase was extracted with Et_2O (× 3). The combined organic layers were washed with brine, dried ($MgSO_4$) and concentrated *in vacuo* to afford the hemiacetal which was immediately reduced using general procedure B.

Note: Use of Et_2O in the work up is important to ensure effective removal of the tetrabutylammonium salt which was found to have a deleterious effect on the reduction step.

General procedure B – Reduction of Hemiacetals:

To a stirred solution of hemiacetal (1.00 equiv.) in DCM (0.20 M) at 0 °C were added Et_3SiH (1.10 equiv.) and $BF_3{\cdot}OEt_2$ (1.10 equiv.) dropwise over 2 min. The reaction was stirred at 0 °C for 40 min then was quenched with sat. aq. $NaHCO_3$. The reaction was diluted with DCM then the aqueous phase was separated and extracted with DCM (× 3). The combined organic layers were washed with 1M aq. HCl (except for acid sensitive compounds), brine, dried ($MgSO_4$) and concentrated *in vacuo* to afford the crude product.

3. Diol Substrates

cis-1,4-Butene diol and *trans*-1,4-butene diol were purchased from commercial sources and were used as received.

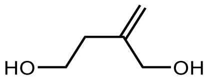

2-Methylenebutane-1,4-diol (Compound 9): Prepared using a modification of the procedure reported by Min *et al.*[1] To a solution of dimethyl itaconate (493 µL, 3.50 mmol, 1.00 equiv.) in THF (23 mL) at 0 °C was added dropwise DIBAL (1.0 M in hexanes, 14.2 mL, 4.1 equiv.). The reaction mixture was then allowed to warm to room temperature and held for 3 h. The reaction was quenched with sat. aq. Rochelle's salt (20 mL) resulting in the formation of a gel. The mixture was diluted with sat. aq. Rochelle's salt (20 mL) and EtOAc (20 mL), then stirred until a biphasic mixture was obtained (15 min.). The organic phase was separated, then the aqueous phase was extracted with EtOAc (3 × 20 mL). The combined organic phases were dried ($MgSO_4$), then concentrated *in vacuo* to afford a pale yellow oil. Purification by flash column chromatography (SiO_2, EtOAc : Cyclohexane – 4:1) gave the *title compound* **9** (130 mg, 1.27 mmol, 36%) as a colourless oil; ^{1}H NMR (400 MHz, $CDCl_3$) δ 5.12–5.08 (1H, m), 4.95 (1H, br s), 4.07 (2H, br s), 3.73 (2H, t, J = 6.0 Hz), 3.29–2.74 (2H, br m), 2.35 (2H, t, J = 6.0 Hz); ^{13}C NMR (101 MHz, $CDCl_3$) δ 146.1, 113.5, 66.1, 61.6, 36.9.

Data are consistent with those reported in the literature.[1]

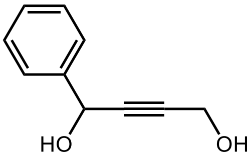

1-Phenylbut-2-yne-1,4-diol (Compound S1): To a solution of propargyl alcohol (520 µL, 8.92 mmol, 1.00 equiv.) in THF (10 mL) at −10 °C under argon was added ^{n}BuLi (7.12 mL, 17.8 mmol, 2.50 M, 2.00 equiv.) dropwise. The mixture was left to stir for 1 h, then benzaldehyde (997 µL, 9.81 mmol, 1.10 equiv.) was added dropwise over 10 min. The mixture was left to stir for 5 h before being quenched with sat. aq. NH_4Cl (20 mL). The aqueous layer was extracted with EtOAc (3 × 10 mL) then the combined organic layers were washed with brine (20 mL), dried ($MgSO_4$) and concentrated *in vacuo* to afford the crude product. Purification by flash column chromatography (SiO_2, Cyclohexane : EtOAc – 3:2) gave the *title compound* **S2** (1.06 g, 6.56 mmol, 73%) as an off-white microcrystalline solid; mp. 88–90 °C (Lit.[4] 82–84 °C); R_f = 0.20 (Cyclohexane/EtOAc, 1:1); ^{1}H NMR (400 MHz, $CDCl_3$) δ 7.57–7.49 (2H, m), 7.43–7.29 (3H, m), 5.51 (1H, app t, J = 1.8 Hz), 4.36 (2H, d, J = 1.8 Hz), 2.00 (2H, br s); ^{13}C NMR (101 MHz, $CDCl_3$) δ 140.3, 128.7, 128.5, 126.6, 85.5, 84.9, 64.6, 51.2.

Data are consistent with those reported in the literature.[2]

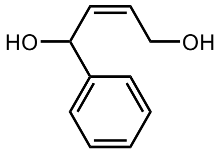

(*Z*)-1-Phenylbut-2-ene-1,4-diol (Compound 11): To a solution of 1-phenylbut-2-yne-1,4-diol **S2** (200 mg, 1.23 mmol, 1.00 equiv.) in EtOAc:pyridine (10:1, 6 mL) was added Pd/$CaCO_3$ (5 wt. % loading, 60.0 mg, 28.1 µmol, 0.02 equiv.). The flask was sealed and then evacuated and backfilled with H_2 three times. The reaction was monitored by TLC until consumption of starting material was observed (1.5 h). The mixture was filtered, then concentrated to give the crude product. Purification by flash column chromatography (SiO_2, Cyclohexane : EtOAc – 5:4) gave the *title compound* **11** (169 mg, 1.03 mmol, 83%) as an off-white microcrystalline solid; mp. 69–71 °C (Lit.[3] 73–75 °C); R_f = 0.13 (Cyclohexane/EtOAc, 1:1); ^{1}H NMR (400 MHz, $CDCl_3$) δ 7.40–7.27 (5H, m), 5.81–5.76 (2H, m), 5.61–5.50 (1H, m), 4.47–4.35 (1H, m), 4.28–4.22 (1H, m); ^{13}C NMR (101 MHz, $CDCl_3$) δ 143.0, 134.6, 130.0, 128.7, 127.7, 126.0, 70.0, 58.7.

Data are consistent with those reported in the literature.[3,4]

4. Aryl Halides and Pseudohalides

Except for those listed below, all other aryl iodides were purchased from commercial sources and were used as received.

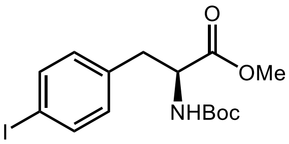

Methyl (*S*)-2-((tert-butoxycarbonyl)amino)-3-(4-iodophenyl)propanoate (Compound 5l): Prepared using a modification of the procedure reported by Verniest *et al.*[5] To a solution of *N*-Boc-4-iodo-L-phenylalanine (1.00 g, 2.56 mmol, 1.00 equiv.) in DMF (10 mL) was added $NaHCO_3$ (645 mg, 7.68 mmol, 3.00 equiv.), then iodomethane (727 mg, 5.12 mmol, 2.00 equiv.) was added dropwise over 30 min. The mixture was left to stir for 24 h then was poured into H_2O (20 mL). The aqueous layer was extracted with EtOAc (3 × 20 mL) then the organic layers were washed with brine (5 × 20 mL), dried ($MgSO_4$) and concentrated *in vacuo* to afford the crude product. Purification by flash column chromatography (SiO_2, Cyclohexane : EtOAc – 9:1) gave the *title compound* **5l** (893 mg, 2.20 mmol, 86%) as a colourless crystalline solid; ^{1}H NMR (400 MHz, $CDCl_3$) δ 7.61 (2H, d, *J* = 8.1 Hz), 6.87 (2H, d, *J* = 8.1 Hz), 4.97 (1H, d, *J* = 7.7 Hz), 4.56 (1H, ddd, *J* = 7.7, 6.4, 6.0 Hz), 3.71 (3H, s), 3.07 (1H, dd, *J* = 13.8, 6.0 Hz), 2.98 (1H, dd, *J* = 13.8, 6.4 Hz), 1.42 (9H, s).

Data are consistent with those reported in the literature.[5]

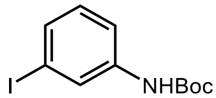

***tert*-Butyl (3-iodophenyl)carbamate (Compound 5m):** Prepared using a modification of the procedure reported by Singh *et al.*[6] To a flask was added 3-iodoaniline (657 mg, 3.00 mmol, 1.00 equiv.) and Boc_2O (655 mg, 3.00 mmol, 1.00 equiv.). The flask was then heated to 80 °C on a rotary evaporator under reduced pressure for 30 min to give the *title compound* **5m** (849 mg, 2.66 mmol, 89%) as an off-white crystalline solid; ^{1}H NMR (400 MHz, $CDCl_3$) δ 7.83 (1H, dd, *J* = 1.9, 1.8 Hz), 7.36 (1H, ddd, *J* = 7.9, 1.8, 1.0 Hz), 7.28–7.24 (1H, m), 6.99 (1H, dd, *J* = 8.0, 7.9 Hz), 1.51 (9H, s).

Data are consistent with those reported in the literature.[7]

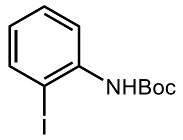

***tert*-Butyl (2-iodophenyl)carbamate (Compound 5v):** Prepared using a modification of the procedure reported by Álvarez *et al.*[8] To a solution of 2-iodoaniline (500 mg, 2.28 mmol, 1.00 equiv.) in THF (4 mL) under argon was added Boc_2O (797 mg, 3.68 mmol, 1.61 equiv.). The mixture was heated at reflux for 24 h in a heating block, then was poured into H_2O (10 mL). The aqueous layer was extracted with EtOAc (3 × 10 mL) then the organic layers were washed with brine (15 mL), dried ($MgSO_4$) and concentrated *in vacuo* to afford the crude product. Purification by flash column chromatography (SiO_2, Cyclohexane 100% → Cyclohexane : EtOAc – 9:1) gave the *title compound* **5v** (650 mg, 2.04 mmol, 89%) as a colourless crystalline solid; ^{1}H NMR (400 MHz, $CDCl_3$) δ 8.05 (1H, dd, *J* = 8.1, 1.6 Hz), 7.74 (1H, dd, *J* = 7.9, 1.5 Hz), 7.31 (1H, dddd, *J* = 8.1, 7.3, 1.5, 0.5 Hz), 6.82 (1H, s), 6.76 (1H, ddd, *J* = 7.9, 7.3, 1.6 Hz), 1.54 (9H, s).

Data are consistent with those reported in the literature.[8]

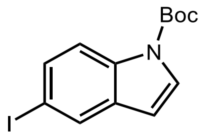

***tert*-Butyl 5-iodo-1*H*-indole-1-carboxylate (Compound 5z):** Prepared using a modification of the procedure reported by Kazmaier *et al.*[9] To a solution of 5-iodoindole (608 mg, 2.50 mmol, 1.00 equiv.) in DCM (10 mL) was added DMAP (15.9 mg, 0.13 mmol, 0.05 equiv.), triethylamine (759 mg, 7.50 mmol, 3.00 equiv.) and Boc_2O (600 mg, 2.75 mmol, 1.10 equiv.), then the mixture was stirred for 2 h. The mixture was concentrated *in vacuo* to give the crude material. Purification by flash column chromatography (SiO_2, Cyclohexane : EtOAc – 30:1) gave the *title compound* **5z** (811 mg, 2.36 mmol, 95%) as an off-white crystalline solid; ^{1}H NMR (400 MHz, $CDCl_3$) δ 7.91–7.88 (2H, m), 7.57 (1H, dd, *J* = 8.7, 1.8 Hz), 7.55 (1H, d, *J* = 3.7 Hz), 6.49 (1H, d, *J* = 3.7 Hz), 1.67 (9H, s).

Data are consistent with those reported in the literature.[9]

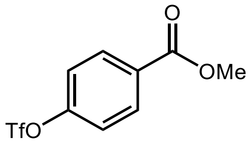

Methyl 4-(((trifluoromethyl)sulfonyl)oxy)benzoate (Compound S2): Prepared according to a modification of the procedure reported by Baran *et al.*[10] To a stirred solution of methyl 4-hydroxybenzoate (456 mg, 3.00 mmol, 1.00 equiv.) and pyridine (364 μL, 4.50 mmol, 1.50 equiv.) in DCM (5.0 mL) at 0 °C was added dropwise triflic anhydride (504 μL, 3.00 mmol, 1.00 equiv.). The resulting mixture was held at 0 °C for 5 min, then was warmed to room temperature and was held for 2 h (reaction complete by TLC). The reaction was diluted with Et_2O (10 mL), then was quenched with 1 M aq. HCl (5 mL). The aqueous phase was separated and extracted with EtOAc (3 × 10 mL), then the combined organic phases were washed with sat. aq. $NaHCO_3$ and brine (15 mL), dried ($MgSO_4$) then concentrated *in vacuo* to afford methyl 4-(((trifluoromethyl)sulfonyl)oxy)benzoate **S3** (842 mg, 2.96 mmol, 99%) as a pale orange oil; R_f = 0.39 (cyclohexane/EtOAc, 4:1); ^{1}H NMR (400 MHz, $CDCl_3$) δ 8.14 (2H, d, J = 8.7 Hz), 7.35 (2H, d, J = 8.7 Hz), 3.94 (3H, s); ^{13}C NMR (101 MHz, $CDCl_3$) δ 165.4, 152.5, 131.9, 130.3, 121.4, 118.7 (q, J = 320.7 Hz), 52.5; ^{19}F NMR (376 MHz, $CDCl_3$) δ −72.8.

Data are consistent with those reported in the literature.[10]

5. Optimization Studies (Table 1)

Optimisation reactions (0.5 mmol) were set up following general procedure A. After 24 h, the reaction mixture was cooled to r.t then 1,3-benzodioxole (1.00 equiv.) was added as a ^{1}H NMR spectroscopy standard. An aliquot of the reaction mixture was then taken and analysed by ^{1}H NMR spectroscopy.

A sample of the hemiacetal was isolated by flash column chromatography (SiO_2, Petroleum ether : EtOAc – 3:1) to confirm the structure.

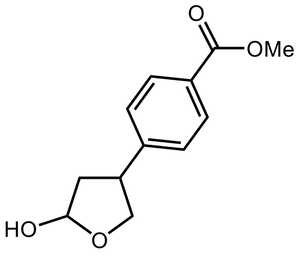

Methyl 4-(5-hydroxytetrahydrofuran-3-yl)benzoate 6a: Isolated as a colourless crystalline solid (d.r. 1.7:1); mp. 74–75 ºC; ν_{max}/cm^{-1} (thin film) 3401, 2980, 1721, 1282, 1111; **^{1}H NMR Major Diastereoisomer** (400 MHz, $CDCl_3$) δ 8.03–7.92 (2H, m), 7.31 (2H, d, *J* = 8.3 Hz), 5.74–5.69 (1H, m), 4.46 (1H, dd, *J* = 8.2, 8.1 Hz), 3.91 (3H, s), 3.85 (1H, dd, *J* = 8.2, 7.6 Hz), 3.82–3.71 (1H, m), 2.68 (1H, br s), 2.39 (1H, ddd, *J* = 13.0, 7.6, 0.8 Hz), 2.12 (1H, ddd, *J* = 13.0, 9.5, 4.9 Hz); **^{13}C NMR Major Diastereoisomer** (101 MHz, $CDCl_3$) δ 166.9, 147.6, 130.0, 128.6, 127.2, 98.9, 73.8, 52.1, 42.5, 41.8; **^{1}H NMR Minor Diastereoisomer** (400 MHz, $CDCl_3$) δ 8.03–7.92 (2H, m), 7.41 (2H, d, *J* = 8.3 Hz), 5.74–5.69 (1H, m), 4.24 (1H, dd, *J* = 8.7, 8.2 Hz), 3.97 (1H, dd, *J* = 8.9, 8.7 Hz), 3.91 (3H, s), 3.55–3.41 (1H, m), 2.65 (1H, ddd, *J* = 13.7, 10.0, 5.5 Hz), 2.01 (1H, ddd, *J* = 13.7, 7.8, 2.8 Hz), 1.58 (1H, br s); **^{13}C NMR Minor Diastereoisomer** (101 MHz, $CDCl_3$) δ 166.9, 146.7, 129.9, 128.6, 127.7, 99.2, 73.0, 52.1, 44.4, 41.6; HRMS (ESI-TOF) m/z: $[M + Na]^+$ calcd for $C_{12}H_{14}NaO_4$, 245.0784; Found 245.0790 (2.30 ppm error).

Note: The relative stereochemistry of the major and minor diastereoisomers was not determined.

6. Synthesis of 3-Substituted Tetrahydrofurans

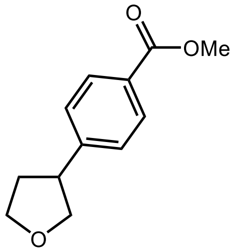

Methyl 4-(tetrahydrofuran-3-yl)benzoate (Compound 8a): Prepared according to general procedure A using $NaHCO_3$ (168 mg, 2.00 mmol, 2.00 equiv.), $Pd(OAc)_2$ (4.5 mg, 0.02 mmol, 0.02 equiv.), $^{n}Bu_4NCl$ (278 mg, 1.00 mmol, 1.00 equiv.), methyl 4-iodobenzoate (262 mg, 1.00 mmol, 1.00 equiv.), *cis*-2-butene-1,4-diol (99 µL, 1.20 mmol, 1.20 equiv.) in MeCN (2.5 mL). The crude material was then immediately reduced according to general procedure B using hemiacetal (222 mg, 1.00 mmol, 1.00 equiv.), Et_3SiH (176 µL, 1.10 mmol, 1.10 equiv.), $BF_3{\cdot}OEt_2$ (136 µL, 1.10 mmol, 1.10 equiv.) in DCM (5 mL). Purification by flash column chromatography (SiO_2, Cyclohexane : EtOAc – 15:1) gave the *title compound* **8a** (197 mg, 0.955 mmol, 96%) as a pale yellow oil; R_f = 0.52 (Pet. ether/EtOAc, 1:1); ν_{max}/cm^{-1} (thin film) 2951, 2860, 1716, 1610, 1435, 1274, 1107, 706; ^{1}H NMR (400 MHz, $CDCl_3$) δ 7.97 (2H, d, *J* = 8.3 Hz), 7.31 (2H, d, *J* = 8.3 Hz), 4.19–4.01 (2H, m), 3.94–3.88 (4H, m), 3.74 (1H, dd, *J* = 8.3, 7.3 Hz), 3.45 (1H, app quint, *J* = 7.6 Hz), 2.39 (1H, app dtd, *J* = 12.8, 7.8, 4.7 Hz), 2.00 (1H, dddd, *J* = 12.8, 8.2, 8.0, 7.7 Hz); ^{13}C NMR (101 MHz, $CDCl_3$) δ 166.9, 148.4, 129.9, 128.4, 127.2, 74.4, 68.4, 52.0, 44.9, 34.6; HRMS (ESI-TOF) m/z: $[M + Na]^+$ calcd for $C_{12}H_{14}NaO_3$, 229.0835; Found 229.0834 (0.30 ppm error).

Data are consistent with those reported in the literature.[11]

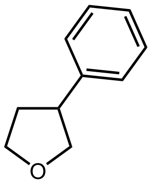

3-Phenyltetrahydrofuran (Compound 8b): Prepared according to general procedure A using $NaHCO_3$ (168 mg, 2.00 mmol, 2.00 equiv.), $Pd(OAc)_2$ (4.5 mg, 20.0 µmol, 0.02 equiv.), $^{n}Bu_4NCl$ (278 mg, 1.00 mmol, 1.00 equiv.), iodobenzene (204 mg, 1.00 mmol, 1.00 equiv.), *cis*-2-butene-1,4-diol (99 µL, 1.20 mmol, 1.20 equiv.) in MeCN (2.5 mL). The crude material was then immediately reduced according to general procedure B using hemiacetal (164 mg, 1.00 mmol, 1.00 equiv.), Et_3SiH (176 µL, 1.10 mmol, 1.10 equiv.), $BF_3{\cdot}OEt_2$ (136 µL, 1.10 mmol, 1.10 equiv.) in DCM (5 mL). Purification by flash column chromatography (SiO_2, Cyclohexane : Et_2O – 9:1) gave the *title compound* **8b** (142 mg, 0.958 mmol, 96%) containing traces of triethylsilanol (<1wt%) as a colourless oil (Yield at 100% purity: 141 mg, 0.951 mmol, 95%); R_f = 0.19 (Cyclohexane/Et_2O, 9:1); ν_{max}/cm^{-1} (thin film) 2968, 2928, 2856;

^1H NMR (400 MHz, $CDCl_3$) δ 7.37–7.17 (5H, m), 4.15 (1H, app t, *J* = 8.0 Hz), 4.08 (1H, app td, *J* = 8.4, 4.5 Hz), 3.97–3.88 (1H, m), 3.73 (1H, app t, *J* = 8.0 Hz), 3.41 (1H, app quint, *J* = 7.9 Hz), 2.43–2.30 (1H, m), 2.10–1.94 (1H, m); ^{13}C NMR (101 MHz, $CDCl_3$) δ 142.6, 128.6, 127.2, 126.5, 74.7, 68.5, 45.0, 34.6; HRMS (EI-MS) m/z: $[M]^+$ calcd for $C_{10}H_{12}O$, 148.0883; Found 148.0883 (0.18 ppm error).

Data are consistent with those reported in the literature.[12]

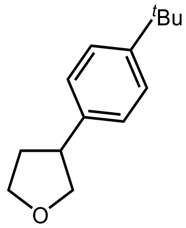

3-(4-(*tert*-Butyl)phenyl)tetrahydrofuran (Compound 8c): Prepared according to general procedure A using $NaHCO_3$ (168 mg, 2.00 mmol, 2.00 equiv.), $Pd(OAc)_2$ (4.5 mg, 20.0 µmol, 0.02 equiv.), $^nBu_4NCl$ (278 mg, 1.00 mmol, 1.00 equiv.), 4-*tert*-butyliodobenzene (260 mg, 1.00 mmol, 1.00 equiv.), *cis*-2-butene-1,4-diol (99 µL, 1.20 mmol, 1.20 equiv.) in MeCN (2.5 mL). The crude material was then immediately reduced according to general procedure B using hemiacetal (220 mg, 1.00 mmol, 1.00 equiv.), Et_3SiH (176 µL, 1.10 mmol, 1.10 equiv.), $BF_3{\cdot}OEt_2$ (136 µL, 1.10 mmol, 1.10 equiv.) in DCM (5 mL). Purification by flash column chromatography (SiO_2, Cyclohexane : EtOAc – 15:1) gave the *title compound* **8c** (152 mg, 0.742 mmol, 74%) as a pale yellow oil; R_f = 0.68 (Pet. ether/EtOAc, 1:1); ν_{max}/cm^{-1} (thin film) 2960, 2904, 2865; ^1H NMR (400 MHz, $CDCl_3$) δ 7.38 (2H, d, *J* = 8.4 Hz), 7.23 (2H, d, *J* = 8.4 Hz), 4.18 (1H, dd, *J* = 8.1, 8.0 Hz), 4.10 (1H, app td, *J* = 8.3, 4.5 Hz), 3.95 (1H, ddd, *J* = 8.3, 8.2, 7.4 Hz), 3.76 (1H, app t, *J* = 8.1 Hz), 3.42 (1H, dddd, *J* = 8.3, 8.2, 8.1, 8.0 Hz), 2.38 (1H, dddd, *J* = 12.4, 8.3, 7.4, 4.5 Hz), 2.05 (1H, app dq, *J* = 12.4, 8.2 Hz), 1.36 (9H, s); ^{13}C NMR (101 MHz, $CDCl_3$) δ 149.3, 139.3, 126.9, 125.4, 74.6, 68.5, 44.5, 34.6, 34.3, 31.3; HRMS (ESI-TOF) m/z: $[M + NH_4]^+$ calcd for $C_{14}H_{24}NO$, 222.1852; Found 222.1844 (4.00 ppm error).

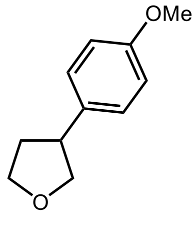

3-(4-Methoxyphenyl)tetrahydrofuran (Compound 8d): Prepared according to general procedure A using $NaHCO_3$ (168 mg, 2.00 mmol, 2.00 equiv.), $Pd(OAc)_2$ (4.5 mg, 20.0 µmol, 0.02 equiv.), $^nBu_4NCl$ (278 mg, 1.00 mmol, 1.00 equiv.), 4-iodoanisole (234 mg, 1.00 mmol, 1.00 equiv.), *cis*-2-butene-1,4-diol (99 µL, 1.20 mmol, 1.20 equiv.) in MeCN (2.5 mL). The crude material was then immediately reduced according to general procedure B using hemiacetal (194 mg, 1.00 mmol, 1.00 equiv.), Et_3SiH

(176 µL, 1.10 mmol, 1.10 equiv.), BF_3•OEt_2 (136 µL, 1.10 mmol, 1.10 equiv.) in anhydrous DCM (5 mL). Purification by flash column chromatography (SiO_2, Cyclohexane : Et_2O – 9:1) gave the *title compound* **8d** (174 mg, 0.976 mmol, 98%) containing traces of triethylsilanol (<3wt%) as a colourless oil (Yield at 100% purity: 170 mg, 0.954 mmol, 95%); R_f = 0.11 (Cyclohexane/Et_2O, 9:1); ν_{max}/cm^{-1} (thin film) 2933, 2857, 1512; ^1H NMR (400 MHz, $CDCl_3$) δ 7.17 (2H, d, *J* = 8.5 Hz), 6.86 (2H, d, *J* = 8.5 Hz), 4.12 (1H, app t, *J* = 8.0 Hz), 4.06 (1H, app td, *J* = 8.3, 4.4 Hz), 3.91 (1H, app q, *J* = 7.8 Hz), 3.79 (3H, s), 3.68 (1H, app t, *J* = 8.0 Hz), 3.36 (1H, app quint, *J* = 7.9 Hz), 2.40–2.27 (1H, m), 1.99 (1H, app dq, *J* = 12.3, 8.2 Hz); ^{13}C NMR (101 MHz, $CDCl_3$) δ 158.2, 134.5, 128.2, 114.0, 74.7, 68.5, 55.3, 44.2, 34.7; HRMS (ESI-TOF) m/z: $[M + H]^+$ calcd for $C_{11}H_{15}O_2$, 179.1067; Found 179.1065 (1.10 ppm error).

Data are consistent with those reported in the literature.[13]

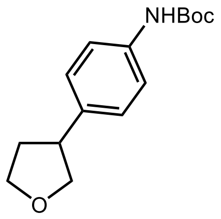

***tert*-Butyl (4-(tetrahydrofuran-3-yl)phenyl)carbamate (Compound 8e)**: Prepared according to general procedure A using $NaHCO_3$ (168 mg, 2.00 mmol, 2.00 equiv.), $Pd(OAc)_2$ (4.5 mg, 20.0 µmol, 0.02 equiv.), $^nBu_4NCl$ (278 mg, 1.00 mmol, 1.00 equiv.), *tert*-butyl (4-iodophenyl)carbamate (319 mg, 1.00 mmol, 1.00 equiv.), *cis*-2-butene-1,4-diol (99 µL, 1.20 mmol, 1.20 equiv.) in MeCN (2.5 mL). The crude material was then immediately reduced according to general procedure B using hemiacetal (279 mg, 1.00 mmol, 1.00 equiv.), Et_3SiH (176 µL, 1.10 mmol, 1.10 equiv.), BF_3•OEt_2 (136 µL, 1.10 mmol, 1.10 equiv.) in DCM (5 mL). Purification by flash column chromatography (SiO_2, Cyclohexane : EtOAc – 15:1) gave the *title compound* **8e** (230 mg, 0.874 mmol, 87%) as a colourless crystalline solid; mp. 114–116 °C; R_f = 0.51 (Pet. ether/EtOAc, 1:1); ν_{max}/cm^{-1} (thin film) 3303, 2975, 2931, 2870, 1724, 1703, 1525, 1235, 1159, 1051; ^1H NMR (400 MHz, $CDCl_3$) δ 7.30 (2H, d, *J* = 8.4 Hz), 7.15 (2H, d, *J* = 8.4 Hz), 6.71 (1H, s), 4.11 (1H, dd, *J* = 8.4, 7.6 Hz), 4.05 (1H, app td, *J* = 8.3, 4.5 Hz), 3.90 (1H, ddd, *J* = 8.3, 8.2, 7.3 Hz), 3.67 (1H, dd, *J* = 8.4, 7.7 Hz), 3.35 (1H, dddd, *J* = 8.0, 7.9, 7.7, 7.6 Hz), 2.32 (1H, dddd, *J* = 12.5, 7.9, 7.3, 4.5 Hz), 1.95 (1H, dddd, *J* = 12.5, 8.3, 8.2, 8.0 Hz), 1.51 (9H, s); ^{13}C NMR (101 MHz, $CDCl_3$) δ 152.8, 137.0, 136.8, 127.6, 118.8, 80.3, 74.5, 68.4, 44.3, 34.5, 28.3; HRMS (ESI-TOF) m/z: $[M + NH_4]^+$ calcd for $C_{15}H_{25}N_2O_3$, 281.1860; Found 281.1859 (0.20 ppm error).

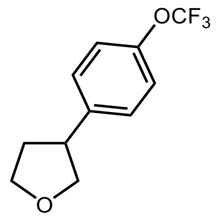

3-(4-(Trifluoromethoxy)phenyl)tetrahydrofuran (Compound 8f): Prepared according to general procedure A using $NaHCO_3$ (168 mg, 2.00 mmol, 2.00 equiv.), $Pd(OAc)_2$ (4.5 mg, 20.0 µmol, 0.02 equiv.), $^nBu_4NCl$ (278 mg, 1.00 mmol, 1.00 equiv.), 4-(trifluoromethoxy)iodobenzene (288 mg, 1.00 mmol, 1.00 equiv.), *cis*-2-butene-1,4-diol (99 µL, 1.20 mmol, 1.20 equiv.) in MeCN (2.5 mL). The crude material was then immediately reduced according to general procedure B using hemiacetal (248 mg, 1.00 mmol, 1.00 equiv.), Et_3SiH (176 µL, 1.10 mmol, 1.10 equiv.), $BF_3{\cdot}OEt_2$ (136 µL, 1.10 mmol, 1.10 equiv.) in DCM (5 mL). Purification by flash column chromatography (SiO_2, Cyclohexane : EtOAc – 15:1) gave the *title compound* **8f** (213 mg, 0.917 mmol, 92%) as a pale yellow oil; R_f = 0.57 (Pet. ether/EtOAc, 1:1); ν_{max}/cm^{-1} (thin film) 2974, 2936, 2865, 1595, 1252, 1216, 1154, 1056; ^{1}H NMR (400 MHz, $CDCl_3$) δ 7.27 (2H, d, *J* = 8.7 Hz), 7.15 (2H, d, *J* = 8.7 Hz), 4.15–4.03 (2H, m), 3.91 (1H, ddd, *J* = 8.5, 7.8, 7.7 Hz), 3.72 (1H, dd, *J* = 8.6, 7.1 Hz), 3.41 (1H, dddd, *J* = 7.9, 7.8, 7.4, 7.1 Hz), 2.37 (1H, dddd, *J* = 12.4, 7.8, 7.7, 4.6 Hz), 1.97, (1H, dddd, *J* = 12.4, 8.3, 7.9, 7.8 Hz); ^{13}C NMR (101 MHz, $CDCl_3$) δ 147.7, 141.7, 128.4, 121.1, 120.5 (q, *J* = 256.6 Hz), 74.5, 68.3, 44.3, 34.6; ^{19}F NMR (376 MHz, $CDCl_3$) δ −57.9; HRMS (ESI-TOF) m/z: $[M + H]^+$ calcd for $C_{11}H_{12}F_3O_2$, 233.0784; Found 233.0789 (2.20 ppm error).

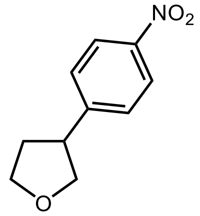

3-(4-Nitrophenyl)tetrahydrofuran (Compound 8g): Prepared according to general procedure A using $NaHCO_3$ (168 mg, 2.00 mmol, 2.00 equiv.), $Pd(OAc)_2$ (4.5 mg, 20.0 µmol, 0.02 equiv.), $^nBu_4NCl$ (278 mg, 1.00 mmol, 1.00 equiv.), 1-iodo-4-nitrobenzene (249 mg, 1.00 mmol, 1.00 equiv.), *cis*-2-butene-1,4-diol (99 µL, 1.20 mmol, 1.20 equiv.) in MeCN (2.5 mL). Purification by flash column chromatography (~10 g SiO_2, Cyclohexane : EtOAc – 4:1 → 1:1) gave the hemiacetal intermediate (151 mg, 0.721 mmol, 72%). The intermediate was then reduced according to general procedure B using hemiacetal (151 mg, 0.72 mmol, 1.00 equiv.), Et_3SiH (126 µL, 0.79 mmol, 1.10 equiv.), $BF_3{\cdot}OEt_2$ (98 µL, 0.79 mmol, 1.10 equiv.) in DCM (4 mL). Purification by flash column chromatography (SiO_2, Cyclohexane : EtOAc – 8:1) gave the *title compound* **8g** (120 mg, 0.623 mmol, 87%, 62% over two steps) as a yellow oil; R_f = 0.43 (Pet. ether/EtOAc, 1:1); ν_{max}/cm^{-1} (thin film) 2973, 2937, 2861, 1598,

1513; ^{1}H NMR (400 MHz, $CDCl_3$) δ 8.12 (2H, d, *J* = 8.7 Hz), 7.39 (2H, d, *J* = 8.7 Hz), 4.13–4.03 (2H, m), 3.89 (1H, ddd, *J* = 8.2, 7.9, 7.8 Hz), 3.75 (1H, dd, *J* = 8.7, 6.7 Hz), 3.49 (1H, dddd, *J* = 8.0, 7.9, 7.5, 6.7 Hz), 2.42 (1H, dddd, *J* = 12.6, 8.0, 7.9, 4.8 Hz), 1.97 (1H, dddd, *J* = 12.6, 8.1, 7.8, 7.5 Hz); ^{13}C NMR (101 MHz, $CDCl_3$) δ 151.1, 146.5, 127.9, 123.7, 74.2, 68.2, 44.7, 34.5; HRMS (ESI-TOF) m/z: $[M + Na]^+$ calcd for $C_{10}H_{11}NNaO_3$, 216.0631; Found 216.0630 (0.60 ppm error).

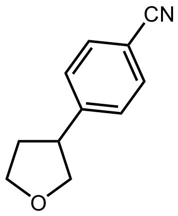

4-(Tetrahydrofuran-3-yl)benzonitrile (Compound 8h): Prepared according to general procedure A using $NaHCO_3$ (168 mg, 2.00 mmol, 2.00 equiv.), $Pd(OAc)_2$ (4.5 mg, 20.0 µmol, 0.02 equiv.), ^{n}Bu$_4$NCl (278 mg, 1.00 mmol, 1.00 equiv.), 4-iodobenzonitrile (229 mg, 1.00 mmol, 1.00 equiv.), *cis*-2-butene-1,4-diol (99 µL, 1.20 mmol, 1.20 equiv.) in MeCN (2.5 mL). The crude material was then immediately reduced according to general procedure B using hemiacetal (189 mg, 1.00 mmol, 1.00 equiv.), Et_3SiH (176 µL, 1.10 mmol, 1.10 equiv.), BF_3•OEt_2 (136 µL, 1.10 mmol, 1.10 equiv.) in DCM (5 mL). Purification by flash column chromatography (SiO_2, Cyclohexane : EtOAc – 15:1) gave the *title compound* **8h** (117 mg, 0.675 mmol, 68%) as a pale yellow oil; R_f = 0.43 (Pet. ether/EtOAc, 1:1); ν_{max}/cm^{-1} (thin film) 2972, 2935, 2864, 2226, 1054, 834, 561; ^{1}H NMR (400 MHz, $CDCl_3$) δ 7.58 (2H, d, *J* = 8.2 Hz), 7.35 (2H, d, *J* = 8.2 Hz), 4.14–4.01 (2H, m), 3.90 (1H, ddd, *J* = 8.3, 7.9, 7.8 Hz), 3.74 (1H, dd, *J* = 8.6, 6.5 Hz), 3.45 (1H, dddd, 7.7, 7.6, 7.4, 6.5 Hz), 2.40 (1H, dddd, *J* = 12.6, 7.8, 7.6, 4.7 Hz), 1.96 (1H, app dq, *J* = 12.6, 7.7 Hz); ^{13}C NMR (101 MHz, $CDCl_3$) δ 148.8, 132.3, 128.0, 118.8, 110.3, 74.2, 68.3, 44.9, 34.5; HRMS (ESI-TOF) m/z: $[M + Na]^+$ calcd for $C_{11}H_{11}NNaO$, 196.0733; Found 196.0732 (0.20 ppm error).

Data are consistent with those reported in the literature.[13]

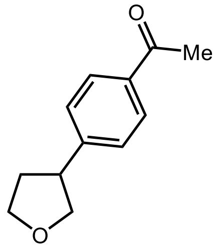

1-(4-(Tetrahydrofuran-3-yl)phenyl)ethan-1-one (Compound 8i): Prepared according to general procedure A using $NaHCO_3$ (168 mg, 2.00 mmol, 2.00 equiv.), $Pd(OAc)_2$ (4.5 mg, 20.0 µmol, 0.02 equiv.), ^{n}Bu$_4$NCl (278 mg, 1.00 mmol, 1.00 equiv.), 4'-iodoacetophenone (246 mg, 1.00 mmol, 1.00 equiv.), *cis*-2-butene-1,4-diol (99 µL, 1.20 mmol, 1.20 equiv.) in MeCN (2.5 mL). The crude material was then immediately reduced according to general procedure B using hemiacetal (206 mg, 1.00 mmol,

1.00 equiv.), Et_3SiH (176 µL, 1.10 mmol, 1.10 equiv.), BF_3•OEt_2 (136 µL, 1.10 mmol, 1.10 equiv.) in DCM (5 mL). Purification by flash column chromatography (SiO_2, Cyclohexane : EtOAc – 15:1 → 11:1) gave the *title compound* **8i** (170 mg, 0.894 mmol, 89%) as a pale yellow oil; R_f = 0.41 (Pet. ether/EtOAc, 1:1); ν_{max}/cm^{-1} (thin film) 2969, 2929, 2860, 1678, 1606; ^{1}H NMR (400 MHz, $CDCl_3$) δ 7.87 (2H, d, *J* = 8.2 Hz), 7.31 (2H, d, *J* = 8.2 Hz), 4.10 (1H, dd, *J* = 8.2, 7.4 Hz), 4.05 (1H, ddd, *J* = 8.2, 8.0, 4.7 Hz), 3.89 (1H, ddd, *J* = 8.2, 7.9, 7.7 Hz), 3.72 (1H, dd, *J* = 8.2, 7.2 Hz), 3.43 (1H, dddd, *J* = 7.7, 7.6, 7.5, 7.2 Hz), 2.55 (3H, s), 2.37 (1H, dddd, *J* = 12.5, 7.9, 7.7, 4.7 Hz), 1.97 (1H, dddd, *J* = 12.5, 8.0, 7.7, 7.6 Hz); ^{13}C NMR (101 MHz, $CDCl_3$) δ 197.5, 148.6, 135.4, 128.6, 127.3, 74.3, 68.3, 44.8, 34.5, 26.4; HRMS (ESI-TOF) m/z: $[M + Na]^+$ calcd for $C_{12}H_{14}NaO_2$, 213.0886; Found 213.0884 (0.90 ppm error).

Data are consistent with those reported in the literature.[13]

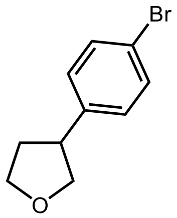

3-(4-Bromophenyl)tetrahydrofuran (Compound 8j): Prepared according to general procedure A using $NaHCO_3$ (168 mg, 2.00 mmol, 2.00 equiv.), $Pd(OAc)_2$ (4.5 mg, 20.0 µmol, 0.02 equiv.), ^{n}Bu_4NCl (278 mg, 1.00 mmol, 1.00 equiv.), 4-bromoiodobenzene (283 mg, 1.00 mmol, 1.00 equiv.), *cis*-2-butene-1,4-diol (99 µL, 1.20 mmol, 1.20 equiv.) in MeCN (2.5 mL). The crude material was then immediately reduced according to general procedure B using hemiacetal (243 mg, 1.00 mmol, 1.00 equiv.), Et_3SiH (176 µL, 1.10 mmol, 1.10 equiv.), BF_3•OEt_2 (136 µL, 1.10 mmol, 1.10 equiv.) in DCM (5 mL). Purification by flash column chromatography (SiO_2, Cyclohexane : EtOAc – 15:1) gave the *title compound* **8j** (182 mg, 0.741 mmol, 74%) as a yellow oil; R_f = 0.61 (Pet. ether/EtOAc, 1:1); ν_{max}/cm^{-1} (thin film) 2969, 2930, 2856, 1489; ^{1}H NMR (400 MHz, $CDCl_3$) δ 7.42 (2H, d, *J* = 8.5 Hz), 7.12 (2H, d, *J* = 8.5 Hz), 4.14–4.00 (2H, m), 3.90 (1H, ddd, *J* = 8.4, 8.0, 7.9 Hz), 3.69 (1H, dd, *J* = 8.3, 7.1 Hz), 3.36 (1H, app quint, *J* = 7.7 Hz), 2.36 (1H, dddd, *J* = 12.7, 7.9, 7.8, 4.7 Hz), 1.95 (1H, dddd, *J* = 12.7, 8.2, 8.0, 7.8 Hz); ^{13}C NMR (101 MHz, $CDCl_3$) δ 141.9, 131.6, 128.9, 120.1, 74.4, 68.4, 44.4, 34.6; HRMS (EI-MS) m/z: $[M]^+$ calcd for $C_{10}H_{11}{}^{79}BrO$, 225.9988; Found 225.9989 (0.62 ppm error).

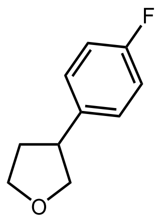

3-(4-Fluorophenyl)tetrahydrofuran (Compound 8k): Prepared according to general procedure A using $NaHCO_3$ (168 mg, 2.00 mmol, 2.00 equiv.), $Pd(OAc)_2$ (4.5 mg, 20.0 µmol, 0.02 equiv.), $^nBu_4NCl$ (278 mg, 1.00 mmol, 1.00 equiv.), 4-fluoroiodobenzene (222 mg, 1.00 mmol, 1.00 equiv.), *cis*-2-butene-1,4-diol (99 µL, 1.20 mmol, 1.20 equiv.) in MeCN (2.5 mL). The crude material was then immediately reduced according to general procedure B using hemiacetal (182 mg, 1.00 mmol, 1.00 equiv.), Et_3SiH (176 µL, 1.10 mmol, 1.10 equiv.), BF_3•OEt_2 (136 µL, 1.10 mmol, 1.10 equiv.) in DCM (5 mL). Purification by flash column chromatography (SiO_2, Cyclohexane : EtOAc – 15:1) gave the *title compound* **8k** (132 mg, 0.796 mmol, 80%) as a pale yellow oil; R_f = 0.52 (Pet. ether/EtOAc, 1:1); ν_{max}/cm^{-1} (thin film) 2971, 2933, 2862, 1509, 1222; ^{1}H NMR (500 MHz, $CDCl_3$) δ 7.26–7.14 (2H, m), 7.04–6.94 (2H, m), 4.11 (1H, dd, *J* = 8.5, 7.6 Hz), 4.06 (1H, ddd, *J* = 8.5, 8.2, 4.6 Hz), 3.90 (1H, ddd, *J* = 8.5, 8.1, 7.9 Hz), 3.69 (1H, dd, *J* = 8.5, 7.4 Hz), 3.38 (1H, dddd, *J* = 7.8, 7.7, 7.6, 7.4 Hz), 2.35 (1H, dddd, *J* = 12.5, 8.1, 7.7, 4.6 Hz), 1.96 (1H, dddd, *J* = 12.5, 8.2, 7.9, 7.8 Hz); ^{13}C NMR (126 MHz, $CDCl_3$) δ 161.5 (d, *J* = 245.1 Hz), 138.4 (d, *J* = 3.2 Hz), 128.6 (d, *J* = 7.8 Hz), 115.3 (d, *J* = 20.9 Hz), 74.6, 68.4, 44.2, 34.7; ^{19}F NMR (376 MHz, $CDCl_3$) δ −116.7; HRMS (EI-MS) m/z: $[M]^+$ calcd for $C_{10}H_{11}FO$, 166.0788; Found 166.0788 (0.46 ppm error).

Data are consistent with those reported in the literature.[13]

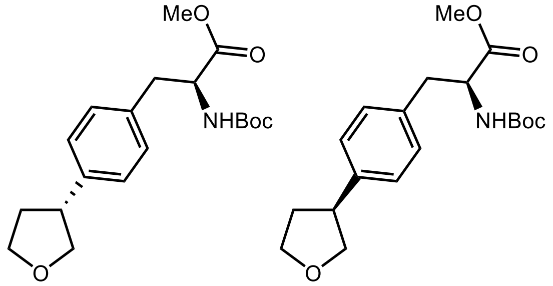

Methyl (*S*)-2-((*tert*-butoxycarbonyl)amino)-3-(4-((*R*)-tetrahydrofuran-3-yl)phenyl)propanoate and Methyl (S)-2-((*tert*-butoxycarbonyl)amino)-3-(4-((*S*)-tetrahydrofuran-3-yl)phenyl)propanoate (Compound 8l and 8l'): Prepared according to general procedure A using $NaHCO_3$ (168 mg, 2.00 mmol, 2.00 equiv.), $Pd(OAc)_2$ (4.5 mg, 20.0 µmol, 0.02 equiv.), $^nBu_4NCl$ (278 mg, 1.00 mmol, 1.00 equiv.), *N*-boc-4-iodo-L-phenylalanine methyl ester (405 mg, 1.00 mmol, 1.00 equiv.), *cis*-2-butene-1,4-diol (99 µL, 1.20 mmol, 1.20 equiv.) in MeCN (2.5 mL). The crude material was then immediately reduced according to general procedure B using hemiacetal (365 mg, 1.00 mmol,

1.00 equiv.), Et_3SiH (176 µL, 1.10 mmol, 1.10 equiv.), $BF_3{\cdot}OEt_2$ (136 µL, 1.10 mmol, 1.10 equiv.) in DCM (5 mL). Purification by flash column chromatography (SiO_2, Cyclohexane : EtOAc – 12:1) gave an inseparable mixture of *title compounds* **8l** and **8l'** (335 mg, 0.958 mmol, 96%, d.r. 1:1) as a colourless oil; R_f = 0.45 (Pet. ether/EtOAc, 1:1); ν_{max}/cm^{-1} (thin film) 3327, 2974, 2932, 2866, 1745, 1711, 1513; ^{1}H NMR (500 MHz, $CDCl_3$) δ 7.17 (2H, d, *J* = 7.8 Hz), 7.06 (2H, d, *J* = 7.8 Hz), 4.99 (1H, d, *J* = 7.9 Hz), 4.57 (1H, ddd, *J* = 7.9, 6.2, 5.4 Hz), 4.11 (1H, app t, *J* = 8.0 Hz), 4.05 (1H, ddd, *J* = 8.2, 8.1, 4.5 Hz), 3.90 (1H, ddd, *J* = 8.1, 8.0, 7.8 Hz), 3.71 (3H, s), 3.68 (1H, app t, *J* = 8.0 Hz), 3.36 (1H, app qd, *J* = 8.0, 7.7 Hz), 3.09 (1H, dd, *J* = 14.0, 5.4 Hz), 3.00 (1H, dd, *J* = 14.0, 6.2 Hz), 2.33 (1H, dddd, *J* = 12.3, 7.8, 7.7, 4.5 Hz), 1.97 (1H, app dq, *J* = 12.3, 8.0 Hz), 1.40 (9H, s); ^{13}C NMR (126 MHz, $CDCl_3$) δ 172.3, 155.0, 141.3, 134.1, 129.5, 127.4, 79.8, 74.6, (74.5), 68.4, 54.3, 52.2, 44.6, 37.9, 34.6, (34.5), 28.2 (3 × CH_3); HRMS (ESI-TOF) m/z: $[M + H]^+$ calcd for $C_{19}H_{28}NO_5$, 350.1962; Found 350.1966 (1.20 ppm error).

Note: The ^{13}C signals for both diastereoisomers overlap, except for those reported in parentheses.

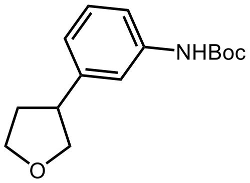

***tert*-Butyl (3-(tetrahydrofuran-3-yl)phenyl)carbamate (Compound 8m)**: Prepared according to general procedure A using $NaHCO_3$ (168 mg, 2.00 mmol, 2.00 equiv.), $Pd(OAc)_2$ (4.5 mg, 20.0 µmol, 0.02 equiv.), $^nBu_4NCl$ (278 mg, 1.00 mmol, 1.00 equiv.), *tert*-butyl (3-iodophenyl)carbamate (319 mg, 1.00 mmol, 1.00 equiv.), *cis*-2-butene-1,4-diol (99 µL, 1.20 mmol, 1.20 equiv.) in MeCN (2.5 mL). The crude material was then immediately reduced according to general procedure B using hemiacetal (180 mg, 1.00 mmol, 1.00 equiv.), Et_3SiH (176 µL, 1.10 mmol, 1.10 equiv.), $BF_3{\cdot}OEt_2$ (136 µL, 1.10 mmol, 1.10 equiv.) in DCM (5 mL). Purification by flash column chromatography (SiO_2, Cyclohexane : EtOAc – 15:1 → 10:1) gave the *title compound* **8m** (234 mg, 0.887 mmol, 89%) as a colourless oil; R_f = 0.57 (Pet. ether/EtOAc, 1:1); ν_{max}/cm^{-1} (thin film) 3308, 2974, 2931, 2871, 1725, 1701; ^{1}H NMR (400 MHz, $CDCl_3$) δ 7.32 (1H, dd, *J* = 1.9, 1.8 Hz), 7.22 (1H, dd, *J* = 8.2, 7.6 Hz), 7.17 (1H, ddd, *J* = 8.2, 1.9, 1.6 Hz), 6.92 (1H, ddd, *J* = 7.6, 1.8, 1.6 Hz), 6.56 (1H, br s), 4.12 (1H, dd, *J* = 8.4, 7.7 Hz), 4.05 (1H, ddd, *J* = 8.4, 8.2, 4.6 Hz), 3.90 (1H, ddd, *J* = 8.4, 7.9, 7.7 Hz), 3.72 (1H, dd, *J* = 8.4, 7.7), 3.37 (1H, app quint, *J* = 7.7 Hz), 2.34 (1H, dddd, *J* = 12.4, 8.1, 7.7, 4.6 Hz), 2.00 (1H, dddd, *J* = 12.4, 8.2, 8.0, 7.9 Hz), 1.52 (9H, s); ^{13}C NMR (101 MHz, $CDCl_3$) δ 152.7, 143.7, 138.6, 129.1, 121.8, 117.4, 116.7, 80.5, 74.5, 68.5, 45.0, 34.5, 28.3; HRMS (ESI-TOF) m/z: $[M + Na]^+$ calcd for $C_{15}H_{21}NNaO_3$, 286.1414; Found 286.1411 (0.80 ppm error).

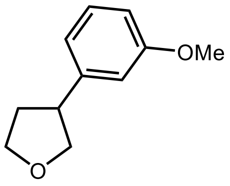

3-(3-Methoxyphenyl)tetrahydrofuran (Compound 8n): Prepared according to general procedure A using $NaHCO_3$ (168 mg, 2.00 mmol, 2.00 equiv.), $Pd(OAc)_2$ (4.5 mg, 20.0 µmol, 0.02 equiv.), $^nBu_4NCl$ (278 mg, 1.00 mmol, 1.00 equiv.), 3-iodoanisole (234 mg, 1.00 mmol, 1.00 equiv.), *cis*-2-butene-1,4-diol (99 µL, 1.20 mmol, 1.20 equiv.) in MeCN (2.5 mL). The crude material was then immediately reduced according to general procedure B using hemiacetal (194 mg, 1.00 mmol, 1.00 equiv.), Et_3SiH (176 µL, 1.10 mmol, 1.10 equiv.), $BF_3{\cdot}OEt_2$ (136 µL, 1.10 mmol, 1.10 equiv.) in DCM (5 mL). Purification by flash column chromatography (SiO_2, Cyclohexane : EtOAc – 15:1) gave the *title compound* **8n** (145 mg, 0.813 mmol, 81%) as a pale yellow oil; R_f = 0.61 (Pet. ether/EtOAc, 1:1); ν_{max}/cm^{-1} (thin film) 2935, 2860, 1601, 1583; ^1H NMR (400 MHz, $CDCl_3$) δ 7.24 (1H, dd, *J* = 8.1, 7.7 Hz), 6.86 (1H, d, *J* =7.7 Hz), 6.82 (1H, s), 6.80–6.75 (1H, m), 4.14 (1H, app t, *J* = 8.0 Hz), 4.07 (1H, app td, *J* = 8.2, 4.5 Hz), 3.92 (1H, ddd, *J* = 8.2, 8.0, 7.8 Hz), 3.81 (3H, s), 3.74 (1H, dd, *J* = 8.0, 7.9 Hz), 3.39 (1H, app quint, *J* = 7.7 Hz), 2.36 (1H, dddd, *J* = 12.3, 7.8, 7.7, 4.5 Hz), 2.02 (1H, dddd, *J* = 12.3, 8.2, 8.0, 7.9 Hz); ^{13}C NMR (101 MHz, $CDCl_3$) δ 159.7, 144.3, 129.4, 119.5, 113.1, 111.4, 74.4, 68.4, 55.0, 44.9, 34.5; HRMS (ESI-TOF) m/z: $[M + NH_4]^+$ calcd for $C_{11}H_{18}NO_2$, 196.1332; Found 196.1328 (1.90 ppm error).

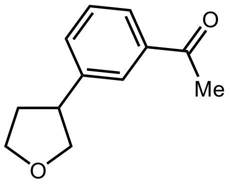

1-(3-(Tetrahydrofuran-3-yl)phenyl)ethan-1-one (Compound 8o): Prepared according to general procedure A using $NaHCO_3$ (168 mg, 2.00 mmol, 2.00 equiv.), $Pd(OAc)_2$ (4.5 mg, 20.0 µmol, 0.02 equiv.), $^nBu_4NCl$ (278 mg, 1.00 mmol, 1.00 equiv.), 3'-iodoacetophenone (246 mg, 1.00 mmol, 1.00 equiv.), *cis*-2-butene-1,4-diol (99 µL, 1.20 mmol, 1.20 equiv.) in MeCN (2.5 mL). The crude material was then immediately reduced according to general procedure B using hemiacetal (206 mg, 1.00 mmol, 1.00 equiv.), Et_3SiH (176 µL, 1.10 mmol, 1.10 equiv.), $BF_3{\cdot}OEt_2$ (136 µL, 1.10 mmol, 1.10 equiv.) in DCM (5 mL). Purification by flash column chromatography (SiO_2, Cyclohexane : EtOAc – 15:1) gave the *title compound* **8o** (170 mg, 0.894 mmol, 89%) as a pale yellow oil; R_f = 0.55 (Pet. ether/EtOAc, 1:1); ν_{max}/cm^{-1} (thin film) 2968, 2931, 2860, 1681; ^1H NMR (400 MHz, $CDCl_3$) δ 7.84 (1H, dd, *J* = 1.8, 1.7 Hz), 7.80 (1H, ddd, *J* = 7.6, 1.8, 1.6 Hz), 7.46 (1H, ddd, *J* = 7.6, 1.7, 1.6 Hz), 7.41 (1H, app t, *J* = 7.6 Hz), 4.15 (1H, dd, *J* = 8.5, 7.6 Hz), 4.08 (1H, ddd, *J* = 8.5, 8.2, 4.6 Hz), 3.92 (1H, ddd, *J* = 8.5, 7.7,

7.5 Hz), 3.74 (1H, dd, J = 8.5, 7.5 Hz), 3.46 (1H, dddd, J = 8.1, 7.9, 7.6, 7.5 Hz), 2.60 (3H, s), 2.40 (1H, dddd, J = 12.5, 8.1, 7.5, 4.6 Hz), 2.02 (1H, dddd, J = 12.5, 8.2, 7.9, 7.7 Hz) ; ^{13}C NMR (101 MHz, $CDCl_3$) δ 198.1, 143.5, 137.4, 131.9, 128.8, 126.9, 126.7, 74.5, 68.4, 44.8, 34.6, 26.7; HRMS (ESI-TOF) m/z: $[M + Na]^+$ calcd for $C_{12}H_{14}NaO_2$, 213.0886; Found 213.0887 (0.40 ppm error).

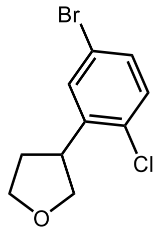

3-(5-Bromo-2-chlorophenyl)tetrahydrofuran (Compound 8p): Prepared according to general procedure A using $NaHCO_3$ (168 mg, 2.00 mmol, 2.00 equiv.), $Pd(OAc)_2$ (4.5 mg, 20.0 µmol, 0.02 equiv.), ^{n}Bu$_4$NCl (278 mg, 1.00 mmol, 1.00 equiv.), 5-bromo-2-chloroiodobenzene (317 mg, 1.00 mmol, 1.00 equiv.), *cis*-2-butene-1,4-diol (99 µL, 1.20 mmol, 1.20 equiv.) in MeCN (2.5 mL). The crude material was then immediately reduced according to general procedure B using hemiacetal (278 mg, 1.00 mmol, 1.00 equiv.), Et_3SiH (176 µL, 1.10 mmol, 1.10 equiv.), BF_3•OEt_2 (136 µL, 1.10 mmol, 1.10 equiv.) in DCM (5 mL). Purification by flash column chromatography (SiO_2, Cyclohexane : EtOAc – 15:1) gave the *title compound* **8p** (174 mg, 0.667 mmol, 67%) as a pale yellow oil; R_f = 0.73 (Pet. ether/EtOAc, 1:1); ν_{max}/cm^{-1} (thin film) 2974, 2932, 2861, 1465, 1039; ^{1}H NMR (400 MHz, $CDCl_3$) δ 7.49–7.43 (1H, m), 7.28–7.23 (1H, m), 7.22–7.16 (1H, m), 4.11–3.98 (2H, m), 3.93–3.85 (1H, m), 3.84–3.72 (2H, m), 2.45–2.30 (1H, m), 2.03–1.85 (1H, m); ^{13}C NMR (101 MHz, $CDCl_3$) δ 142.8, 132.7, 130.7, 130.5, 130.3, 120.8, 73.0, 67.8, 41.0, 33.1; HRMS (EI-MS) m/z: $[M]^+$ calcd for $C_{10}H_{10}{}^{79}Br^{35}ClO$, 259.9598; Found 259.9599 (0.35 ppm error).

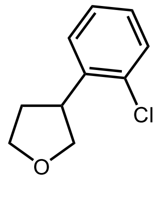

3-(2-Chlorophenyl)tetrahydrofuran (Compound 8q): Prepared according to general procedure A using $NaHCO_3$ (168 mg, 2.00 mmol, 2.00 equiv.), $Pd(OAc)_2$ (4.5 mg, 20.0 µmol, 0.02 equiv.), ^{n}Bu$_4$NCl (278 mg, 1.00 mmol, 1.00 equiv.), 2-chloroiodobenzene (239 mg, 1.00 mmol, 1.00 equiv.), *cis*-2-butene-1,4-diol (99 µL, 1.20 mmol, 1.20 equiv.) in MeCN (2.5 mL). The crude material was then immediately reduced according to general procedure B using hemiacetal (199 mg, 1.00 mmol, 1.00 equiv.), Et_3SiH (176 µL, 1.10 mmol, 1.10 equiv.), BF_3•OEt_2 (136 µL, 1.10 mmol, 1.10 equiv.) in DCM (5 mL). Purification by flash column chromatography (SiO_2, Cyclohexane : EtOAc – 15:1) gave the *title compound* **8q** (144 mg, 0.789 mmol, 79%) as a pale yellow oil; R_f = 0.59 (Pet. ether/EtOAc, 1:1);

ν_{max}/cm^{-1} (thin film) 2974, 2933, 2861, 1475, 1442; ^{1}H NMR (400 MHz, $CDCl_3$) δ 7.36 (1H, dd, *J* = 8.0, 1.4 Hz), 7.35 (1H, dd, *J* = 7.7, 1.8 Hz), 7.24 (1H, ddd, *J* = 7.7, 7.6, 1.4 Hz), 7.15 (1H, ddd, *J* = 8.0, 7.6, 1.8 Hz), 4.13 (1H, dd, *J* = 8.4, 6.8 Hz), 4.04 (1H, ddd, *J* = 8.3, 8.0, 5.5 Hz), 3.96–3.84 (2H, m), 3.80 (1H, dd, *J* = 8.4, 6.3 Hz), 2.39 (1H, dddd, *J* = 12.5, 8.0, 7.5, 5.5 Hz), 1.99 (1H, dddd, *J* = 12.5, 8.0, 7.0, 6.7 Hz); ^{13}C NMR (101 MHz, $CDCl_3$) δ 140.4, 133.9, 129.5, 127.5, 127.2, 127.1, 73.3, 68.0, 41.1, 33.2; HRMS (EI-MS) m/z: $[M]^+$ calcd for $C_{10}H_{11}{}^{35}ClO$, 182.0493; Found 182.0502 (4.72 ppm error).

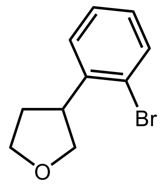

3-(2-Bromophenyl)tetrahydrofuran (Compound 8r): Prepared according to general procedure A using $NaHCO_3$ (168 mg, 2.00 mmol, 2.00 equiv.), $Pd(OAc)_2$ (4.5 mg, 20.0 µmol, 0.02 equiv.), $^{n}Bu_4NCl$ (278 mg, 1.00 mmol, 1.00 equiv.), 2-bromoiodobenzene (283 mg, 1.00 mmol, 1.00 equiv.), *cis*-2-butene-1,4-diol (99 µL, 1.20 mmol, 1.20 equiv.) in MeCN (2.5 mL). The crude material was then immediately reduced according to general procedure B using hemiacetal (243 mg, 1.00 mmol, 1.00 equiv.), Et_3SiH (176 µL, 1.10 mmol, 1.10 equiv.), $BF_3{\cdot}OEt_2$ (136 µL, 1.10 mmol, 1.10 equiv.) in DCM (5 mL). Purification by flash column chromatography (SiO_2, Cyclohexane : EtOAc – 15:1) gave the *title compound* **8r** (172 mg, 0.756 mmol, 76%) as a colourless oil; R_f = 0.61 (Pet. ether/EtOAc, 1:1); ν_{max}/cm^{-1} (thin film) 3060, 2973, 2860, 1470; ^{1}H NMR (400 MHz, $CDCl_3$) δ 7.55 (1H, dd, *J* = 7.8, 1.4 Hz), 7.34 (1H, dd, *J* = 7.9, 1.8 Hz), 7.28 (1H, ddd, 7.9, 7.5, 1.4 Hz), 7.07 (1H, ddd, *J* = 7.8, 7.5, 1.8 Hz), 4.12 (1H, dd, *J* = 8.4, 6.8 Hz), 4.04 (1H, ddd, *J* = 8.3, 8.1, 5.5 Hz), 3.92 (1H, ddd, *J* = 8.3, 7.5, 6.9 Hz), 3.87 (1H, dddd, *J* = 7.9, 6.9, 6.8, 5.8 Hz), 3.80 (1H, dd, *J* = 8.4, 5.8 Hz), 2.40 (1H, dddd, *J* = 13.0, 7.9, 7.5, 5.5 Hz), 1.97 (1H, dddd, *J* = 13.0, 8.1, 6.9, 6.6 Hz); ^{13}C NMR (101 MHz, $CDCl_3$) δ 142.2, 132.8, 127.8, 127.7, 127.3, 124.7, 73.4, 68.0, 43.7, 33.5; HRMS (ESI-TOF) m/z: $[M + Na]^+$ calcd for $C_{10}H_{11}{}^{79}BrNaO$, 248.9885; Found 248.9890 (1.70 ppm error).

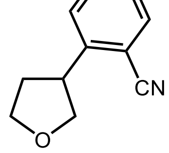

2-(Tetrahydrofuran-3-yl)benzonitrile (Compound 8s): Prepared according to general procedure A using $NaHCO_3$ (168 mg, 2.00 mmol, 2.00 equiv.), $Pd(OAc)_2$ (4.5 mg, 20.0 µmol, 0.02 equiv.), $^{n}Bu_4NCl$ (278 mg, 1.00 mmol, 1.00 equiv.), 2-iodobenzonitrile (229 mg, 1.00 mmol, 1.00 equiv.), *cis*-2-butene-

1,4-diol (99 µL, 1.20 mmol, 1.20 equiv.) in MeCN (2.5 mL). The crude material was then immediately reduced according to general procedure B using hemiacetal (189 mg, 1.00 mmol, 1.00 equiv.), Et_3SiH (176 µL, 1.10 mmol, 1.10 equiv.), BF_3•OEt_2 (136 µL, 1.10 mmol, 1.10 equiv.) in DCM (5 mL). Purification by flash column chromatography (SiO_2, Cyclohexane : EtOAc – 15:1) gave the *title compound* **8s** (121 mg, 0.697 mmol, 70%) as a pale yellow oil; R_f = 0.55 (Pet. ether/EtOAc, 1:1); ν_{max}/cm^{-1} (thin film) 2973, 2954, 2936, 2866, 2223; ^1H NMR (400 MHz, $CDCl_3$) δ 7.60 (1H, d, *J* = 7.6 Hz), 7.55 (1H, dd, *J* = 8.2, 7.2 Hz), 7.45 (1H, d, *J* = 8.2 Hz), 7.30 (1H, dd, *J* = 7.6, 7.2 Hz), 4.15–4.03 (2H, m), 3.90 (1H, ddd, *J* = 8.1, 7.8, 7.4 Hz), 3.87–3.78 (2H, m), 2.47 (1H, dddd, *J* = 12.8, 7.8, 7.6, 5.1 Hz), 1.97 (1H, dddd, *J* = 12.8, 7.9, 7.4, 6.7 Hz); ^{13}C NMR (101 MHz, $CDCl_3$) δ 147.4, 133.2, 132.7, 126.8, 126.7, 117.9, 112.2, 73.8, 68.2, 42.7, 34.4; HRMS (ESI-TOF) m/z: $[M+Na]^+$ calcd for $C_{11}H_{11}NNaO$, 196.0733; Found 196.0730 (1.60 ppm error).

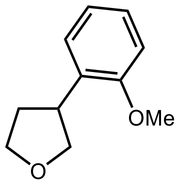

3-(2-Methoxyphenyl)tetrahydrofuran (Compound 8t): Prepared according to general procedure A using $NaHCO_3$ (168 mg, 2.00 mmol, 2.00 equiv.), $Pd(OAc)_2$ (4.5 mg, 20.0 µmol, 0.02 equiv.), $^nBu_4NCl$ (278 mg, 1.00 mmol, 1.00 equiv.), 2-iodoanisole (234 mg, 1.00 mmol, 1.00 equiv.), *cis*-2-butene-1,4-diol (99 µL, 1.20 mmol, 1.20 equiv.) in MeCN (2.5 mL). The crude material was then immediately reduced according to general procedure B using hemiacetal (194 mg, 1.00 mmol, 1.00 equiv.), Et_3SiH (176 µL, 1.10 mmol, 1.10 equiv.), BF_3•OEt_2 (136 µL, 1.10 mmol, 1.10 equiv.) in DCM (5 mL). Purification by flash column chromatography (SiO_2, Cyclohexane : EtOAc – 15:1) gave the *title compound* **8t** (115 mg, 0.647 mmol, 65%) as a pale yellow oil; R_f = 0.64 (Pet. ether/EtOAc, 1:1); ν_{max}/cm^{-1} (thin film) 2937, 2863, 1600, 1585, 1492; ^1H NMR (400 MHz, $CDCl_3$) δ 7.29–7.16 (2H, m), 6.96 (1H, app t, *J* = 7.5 Hz), 6.89 (1H, d, *J* = 8.1 Hz), 4.17 (1H, dd, *J* = 7.2, 6.9 Hz), 4.05 (1H, ddd, *J* = 8.1, 7.9, 4.9 Hz), 3.94 (1H, ddd, *J* = 7.9, 7.6, 7.5 Hz), 3.85 (3H, s), 3.82–3.70 (2H, m), 2.31 (1H, dddd, *J* = 12.4, 7.5, 7.3, 4.9 Hz), 2.05 (1H, dddd, *J* = 12.4, 8.1, 7.7, 7.6 Hz); ^{13}C NMR (101 MHz, $CDCl_3$) δ 157.2, 130.5, 127.3, 126.9, 120.5, 110.2, 73.2, 68.1, 55.2, 38.3, 32.6; HRMS (ESI-TOF) m/z: $[M + H]^+$ calcd for $C_{11}H_{15}O_2$, 179.1067; Found 179.1065 (0.90 ppm error).

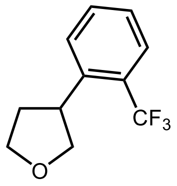

3-(2-(Trifluoromethyl)phenyl)tetrahydrofuran (Compound 8u): Prepared according to general procedure A using $NaHCO_3$ (168 mg, 2.00 mmol, 2.00 equiv.), $Pd(OAc)_2$ (4.5 mg, 20.0 µmol, 0.02 equiv.), $^nBu_4NCl$ (278 mg, 1.00 mmol, 1.00 equiv.), 2-iodobenzotrifluoride (272 mg, 1.00 mmol, 1.00 equiv.), *cis*-2-butene-1,4-diol (99 µL, 1.20 mmol, 1.20 equiv.) in MeCN (2.5 mL). The crude material was then immediately reduced according to general procedure B using hemiacetal (232 mg, 1.00 mmol, 1.00 equiv.), Et_3SiH (176 µL, 1.10 mmol, 1.10 equiv.), $BF_3{\cdot}OEt_2$ (136 µL, 1.10 mmol, 1.10 equiv.) in DCM (5 mL). Purification by flash column chromatography (SiO_2, Cyclohexane : EtOAc – 15:1) gave the *title compound* **8u** (67.5 mg, 0.312 mmol, 31%) as a yellow oil; R_f = 0.64 (Pet. ether/EtOAc, 1:1); ν_{max}/cm^{-1} (thin film) 2955, 2923, 2853, 1738, 1462; ^1H NMR (400 MHz, $CDCl_3$) δ 7.62 (1H, d, *J* = 7.8 Hz), 7.55–7.51 (2H, m), 7.34–7.27 (1H, m), 4.15–4.05 (2H, m), 3.91 (1H, app dt, *J* = 8.6, 7.6 Hz), 3.85–3.79 (2H, m), 2.43 (1H, app dtd, *J* = 12.8, 7.8, 4.9 Hz), 2.03–1.92 (1H, m); ^{13}C NMR (101 MHz, $CDCl_3$) δ 143.1, 132.3, 128.4 (q, *J* = 29.4 Hz), 127.8, 126.2, 125.6 (q, *J* = 6.0 Hz), 124.5 (q, *J* = 273.4 Hz), 75.3, 68.5, 40.0 (q, *J* = 2.0 Hz), 35.7; ^{19}F NMR (376 MHz, $CDCl_3$) δ −58.7; HRMS (EI-MS) m/z: $[M]^+$ calcd for $C_{11}H_{11}F_3O$, 216.0757; Found 216.0740 (7.68 ppm error).

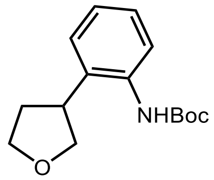

***tert*-Butyl (2-(tetrahydrofuran-3-yl)phenyl)carbamate (Compound 8v)**: Prepared according to general procedure A using $NaHCO_3$ (168 mg, 2.00 mmol, 2.00 equiv.), $Pd(OAc)_2$ (4.5 mg, 20.0 µmol, 0.02 equiv.), $^nBu_4NCl$ (278 mg, 1.00 mmol, 1.00 equiv.), *tert*-butyl (2-iodophenyl)carbamate (319 mg, 1.00 mmol, 1.00 equiv.), *cis*-2-butene-1,4-diol (99 µL, 1.20 mmol, 1.20 equiv.) in MeCN (2.5 mL). The crude material was then immediately reduced according to general procedure B using hemiacetal (279 mg, 1.00 mmol, 1.00 equiv.), Et_3SiH (176 µL, 1.10 mmol, 1.10 equiv.), $BF_3{\cdot}OEt_2$ (136 µL, 1.10 mmol, 1.10 equiv.) in DCM (5 mL). Purification by flash column chromatography (SiO_2, Cyclohexane : EtOAc – 10:1) gave the *title compound* **8v** (79.9 mg, 0.303 mmol, 30%) as a yellow oil; R_f = 0.39 (Pet. ether/EtOAc, 1:1); ν_{max}/cm^{-1} (thin film) 3427, 2974, 2929, 1672, 1491, 1366 ; ^1H NMR (400 MHz, $CDCl_3$) δ 7.65 (1H, app d, *J* = 8.2 Hz), 7.23–7.13 (2H, m), 7.03 (1H, ddd, *J* = 7.5, 7.4, 1.3 Hz), 3.86–3.71 (3H, m), 3.66 (1H, ddd, *J* = 12.9, 6.8, 5.3 Hz), 3.01 (1H, app quint, *J* = 6.1 Hz), 2.09–1.94 (2H, m), 1.69 (1H, br s), 1.52 (9H, s); ^{13}C NMR (101 MHz, $CDCl_3$) δ 153.8, 139.2, 129.6, 128.2, 126.4,

124.5, 123.5, 80.9, 66.2, 42.4, 39.1, 28.4, 26.0; HRMS (ESI-TOF) m/z: $[M + Na]^+$ calcd for $C_{15}H_{21}NNaO_3$, 286.1414; Found 286.1418 (1.50 ppm error).

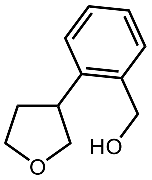

(2-(Tetrahydrofuran-3-yl)phenyl)methanol (Compound 8w): Prepared according to general procedure A using $NaHCO_3$ (168 mg, 2.00 mmol, 2.00 equiv.), $Pd(OAc)_2$ (4.5 mg, 20.0 µmol, 0.02 equiv.), $^nBu_4NCl$ (278 mg, 1.00 mmol, 1.00 equiv.), 2-iodobenzyl alcohol (234 mg, 1.00 mmol, 1.00 equiv.), *cis*-2-butene-1,4-diol (99 µL, 1.20 mmol, 1.20 equiv.) in MeCN (2.5 mL). The crude material was then immediately reduced according to general procedure B using hemiacetal (194 mg, 1.00 mmol, 1.00 equiv.), Et_3SiH (176 µL, 1.10 mmol, 1.10 equiv.), $BF_3{\cdot}OEt_2$ (136 µL, 1.10 mmol, 1.10 equiv.) in DCM (5 mL). Purification by flash column chromatography (SiO_2, Cyclohexane : EtOAc – 12:1) gave the *title compound* **8w** (94.9 mg, 0.532 mmol, 53%) as a yellow oil; R_f = 0.25 (Pet. ether/EtOAc, 1:1); ν_{max}/cm^{-1} (thin film) 3392, 2970, 2932, 2868, 1488, 1452; ^1H NMR (400 MHz, $CDCl_3$) δ 7.39–7.28 (3H, m), 7.21 (1H, app td, *J* = 7.4, 1.5 Hz), 4.73 (2H, s), 4.14–4.02 (2H, m), 3.91 (1H, app q, *J* = 7.8 Hz), 3.81–3.70 (2H, m), 2.42–2.30 (1H, m), 2.15–1.93 (2H, m); ^{13}C NMR (101 MHz, $CDCl_3$) δ 141.6, 138.2, 128.6 (2 × ArCH), 126.4, 126.3, 74.7, 68.4, 63.6, 39.8, 34.8; HRMS (ESI-TOF) m/z: $[M + NH_4]^+$ calcd for $C_{11}H_{18}NO_2$, 196.1332; Found 196.1329 (1.50 ppm error).

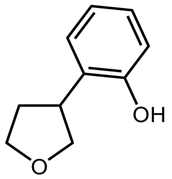

2-(Tetrahydrofuran-3-yl)phenol (Compound 8x): Prepared according to general procedure A using $NaHCO_3$ (168 mg, 2.00 mmol, 2.00 equiv.), $Pd(OAc)_2$ (4.5 mg, 20.0 µmol, 0.02 equiv.), $^nBu_4NCl$ (278 mg, 1.00 mmol, 1.00 equiv.), 2-iodophenol (220 mg, 1.00 mmol, 1.00 equiv.), *cis*-2-butene-1,4-diol (99 µL, 1.20 mmol, 1.20 equiv.) in MeCN (2.5 mL). The crude material was then immediately reduced according to general procedure B using hemiacetal (180 mg, 1.00 mmol, 1.00 equiv.), Et_3SiH (176 µL, 1.10 mmol, 1.10 equiv.), $BF_3{\cdot}OEt_2$ (136 µL, 1.10 mmol, 1.10 equiv.) in DCM (5 mL). Purification by flash column chromatography (SiO_2, Cyclohexane : EtOAc – 15:1 → 10:1) gave the *title compound* **8x** (74.2 mg, 0.452 mmol, 45%) as a pale yellow oil; R_f = 0.50 (Pet. ether/EtOAc, 1:1); ν_{max}/cm^{-1} (thin film) 3241, 2953, 2875; ^1H NMR (400 MHz, $CDCl_3$) δ 7.33 (1H, br s), 7.14–7.08 (2H, m), 6.86–6.80

(2H, m), 4.25 (1H, ddd, *J* = 8.7, 8.6, 3.9 Hz), 4.04 (1H, dd, *J* = 9.1, 4.1 Hz), 3.98 (1H, dd, *J* = 9.1, 7.3 Hz), 3.79 (1H, app q, *J* = 8.5 Hz), 3.59 (1H, dddd, *J* = 9.7, 7.3, 5.4, 4.1 Hz), 2.43 (1H, dddd, *J* = 12.9, 9.7, 7.9, 3.9 Hz), 2.05 (1H, dddd, *J* = 12.9, 8.7, 8.6, 5.4 Hz); ^{13}C NMR (101 MHz, $CDCl_3$) δ 154.0, 129.5, 129.0, 127.9, 120.0, 116.6, 73.4, 68.9, 41.2, 33.0; HRMS (ESI-TOF) m/z: $[M + Na]^+$ calcd for $C_{10}H_{12}NaO_2$, 187.0730; Found 187.0726 (1.90 ppm error).

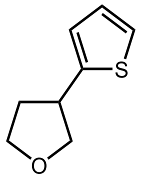

3-(Thiophen-2-yl)tetrahydrofuran (Compound 8y): Prepared according to general procedure A using $NaHCO_3$ (168 mg, 2.00 mmol, 2.00 equiv.), $Pd(OAc)_2$ (4.5 mg, 20.0 µmol, 0.02 equiv.), $^nBu_4NCl$ (278 mg, 1.00 mmol, 1.00 equiv.), 2-iodothiophene (210 mg, 1.00 mmol, 1.00 equiv.), *cis*-2-butene-1,4-diol (99 µL, 1.20 mmol, 1.20 equiv.) in MeCN (2.5 mL). The crude material was then immediately reduced according to general procedure B using hemiacetal (170 mg, 1.00 mmol, 1.00 equiv.), Et_3SiH (176 µL, 1.10 mmol, 1.10 equiv.), $BF_3{\cdot}OEt_2$ (136 µL, 1.10 mmol, 1.10 equiv.) in DCM (5 mL). Purification by flash column chromatography (SiO_2, Cyclohexane : EtOAc – 15:1) gave the *title compound* **8y** (118 mg, 0.764 mmol, 76%) as a yellow oil; R_f = 0.66 (Pet. ether/EtOAc, 1:1); ν_{max}/cm^{-1} (thin film) 2955, 2924, 2854, 1737; ^{1}H NMR (400 MHz, $CDCl_3$) δ 7.16 (1H, dd, *J* = 5.1, 1.1 Hz), 6.95 (1H, dd, *J* = 5.1, 3.5 Hz), 6.90 (1H, dt, *J* = 3.5, 1.0 Hz), 4.15 (1H, dd, *J* = 7.1, 7.0 Hz), 4.05 (1H, ddd, *J* = 8.5, 8.1, 5.1 Hz), 3.93 (1H, app dt, *J* = 8.5, 7.5 Hz), 3.76–3.64 (2H, m), 2.40 (1H, dddd, *J* = 12.5, 7.6, 7.5, 5.1 Hz), 2.05 (1H, dddd, *J* = 12.5, 8.1, 7.7, 7.5 Hz); ^{13}C NMR (101 MHz, $CDCl_3$) δ 145.7, 126.7, 123.5, 123.2, 74.7, 68.1, 40.3, 35.1; HRMS (EI-MS) m/z: $[M]^+$ calcd for $C_8H_{10}OS$, 154.0447; Found 154.0440 (4.30 ppm error).

Data are consistent with those reported in the literature.[13]

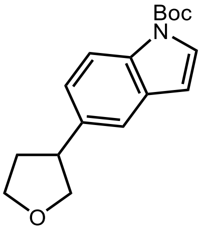

***tert*-Butyl 5-(tetrahydrofuran-3-yl)-1*H*-indole-1-carboxylate (Compound 8z)**: Prepared according to general procedure A using $NaHCO_3$ (168 mg, 2.00 mmol, 2.00 equiv.), $Pd(OAc)_2$ (4.5 mg, 20.0 µmol, 0.02 equiv.), $^nBu_4NCl$ (278 mg, 1.00 mmol, 1.00 equiv.), *N*-Boc-5-iodoindole (343 mg, 1.00 mmol,

1.00 equiv.), *cis*-2-butene-1,4-diol (99 µL, 1.20 mmol, 1.20 equiv.) in MeCN (2.5 mL). The crude material was then immediately reduced according to general procedure B using hemiacetal (303 mg, 1.00 mmol, 1.00 equiv.), Et_3SiH (176 µL, 1.10 mmol, 1.10 equiv.), BF_3•OEt_2 (136 µL, 1.10 mmol, 1.10 equiv.) in DCM (5 mL). Purification by flash column chromatography (SiO_2, Cyclohexane : EtOAc – 15:1) gave the *title compound* **8z** (242 mg, 0.840 mmol, 84%) as an off-white crystalline solid; mp. 47–49 °C; R_f = 0.64 (Pet. ether/EtOAc, 1:1); ν_{max}/cm^{-1} (thin film) 2974, 2931, 2865, 1728; ^{1}H NMR (500 MHz, $CDCl_3$) δ 8.07 (1H, d, *J* = 8.6 Hz), 7.59 (1H, d, *J* = 3.7 Hz), 7.44 (1H, d, *J* = 1.8 Hz), 7.22 (1H, dd, *J* = 8.6, 1.8 Hz), 6.53 (1H, d, *J* = 3.7 Hz), 4.18 (1H, app t, *J* = 8.0 Hz), 4.11 (1H, ddd, *J* = 8.2, 8.1, 4.5 Hz), 3.95 (1H, ddd, *J* = 8.1, 8.0, 7.8 Hz), 3.78 (1H, 1H, dd, *J* = 8.0, 7.8 Hz), 3.50 (1H, dddd, *J* = 8.2, 8.0, 7.8, 7.7 Hz), 2.40 (1H, dddd, *J* = 12.2, 7.8, 7.7, 4.5 Hz), 2.06 (1H, app dq, *J* = 12.2, 8.0 Hz), 1.67 (9H, s); ^{13}C NMR (126 MHz, $CDCl_3$) δ 149.7, 137.0, 133.9, 130.8, 126.2, 123.6, 119.1, 115.2, 107.1, 83.6, 74.9, 68.5, 44.9, 35.0, 28.2 (3 × CH_3); HRMS (ESI-TOF) m/z: $[M + NH_4]^+$ calcd for $C_{17}H_{25}N_2O_3$, 305.1860; Found 305.1858 (0.50 ppm error).

Data are consistent with those reported in the literature.[14]

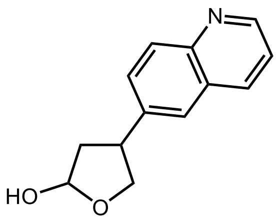

4-(Quinolin-6-yl)tetrahydrofuran-2-ol (Compound 6aa): Prepared according to a modified version of general procedure A (reaction carried out on a 0.59 mmol scale using THF as the solvent) using $NaHCO_3$ (99.1 mg, 1.18 mmol, 2.00 equiv.), $Pd(OAc)_2$ (2.7 mg, 0.012 mmol, 0.02 equiv.), $^nBu_4NCl$ (164 mg, 0.59 mmol, 1.00 equiv.), 6-iodoquinoline (150 mg, 0.59 mmol, 1.00 equiv.), *cis*-2-butene-1,4-diol (58.4 µL, 0.71 mmol, 1.20 equiv.) in THF (1.5 mL). Purification by flash column chromatography (SiO_2, EtOAc) gave the *title compound* **6aa** (112 mg, 0.520 mmol, 88%, d.r. 1.7:1) as an off-white microcrystalline solid; mp. 138–140 °C; R_f = 0.17 (EtOAc); ν_{max}/cm^{-1} (thin film) 3171, 2942, 2872, 1502, 1025; **Major:** ^{1}H NMR (400 MHz, $CDCl_3$) δ 8.95–8.80 (1H, m), 8.15–8.05 (2H, m), 7.80–7.58 (2H, m), 7.44–7.35 (1H, m), 5.88–5.74 (1H, m), 4.59–4.47 (1H, m), 4.01–3.86 (3H, m), 2.46 (1H, dd, *J* = 13.0, 6.7 Hz), 2.30–2.18 (1H, m); ^{13}C NMR (101 MHz, $CDCl_3$) δ 150.0, 147.3, 140.7, 135.8, 129.8, 129.2, 128.2, 125.4, 121.4, 99.0, 73.8, 42.5, 42.0; **Minor:** ^{1}H NMR (400 MHz, $CDCl_3$) δ 8.95–8.80 (1H, m), 8.15–8.05 (2H, m), 7.70–7.58 (2H, m), 7.44–7.35 (1H, m), 5.88–5.74 (1H, m), 4.31 (1H, app t, *J* = 8.2 Hz), 4.08 (1H, app t, *J* = 9.0 Hz), 4.01–3.86 (1H, m), 3.69–3.57 (1H, m), 2.73 (1H, ddd, *J* = 13.6, 10.0, 5.5 Hz), 2.12 (1H, ddd, *J* = 13.6, 8.0, 2.9 Hz); ^{13}C NMR (101 MHz, $CDCl_3$) δ 150.0, 147.3,

139.7, 135.9, 129.8, 129.7, 128.2, 125.9, 121.3, 99.2, 73.1, 44.4, 41.8; HRMS (ESI-TOF) m/z: [M + H]$^+$ calcd for $C_{13}H_{14}NO_2$, 216.1019; Found 216.1021 (0.80 ppm error).

Note: The relative stereochemistry of the major and minor diastereoisomers was not determined.

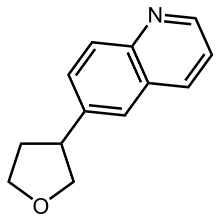

6-(Tetrahydrofuran-3-yl)quinoline (Compound 8aa): Prepared according to a modified version (THF as solvent) of general procedure A using $NaHCO_3$ (168 mg, 2.00 mmol, 2.00 equiv.), $Pd(OAc)_2$ (4.5 mg, 20.0 µmol, 0.02 equiv.), $^{n}Bu_4NCl$ (278 mg, 1.00 mmol, 1.00 equiv.), 6-iodoquinoline (255 mg, 1.00 mmol, 1.00 equiv.), *cis*-2-butene-1,4-diol (99 µL, 1.20 mmol, 1.20 equiv.) in THF (2.5 mL). The crude material was then immediately reduced according to general procedure B using hemiacetal (215 mg, 1.00 mmol, 1.00 equiv.), Et_3SiH (176 µL, 1.10 mmol, 1.10 equiv.), $BF_3{\cdot}OEt_2$ (136 µL, 1.10 mmol, 1.10 equiv.) in DCM (25 mL). Purification by flash column chromatography (SiO_2, Cyclohexane : EtOAc – 15:1 → 6:1) gave the *title compound* **8aa** (74.0 mg, 0.371 mmol, 37%) as a colourless oil; R_f = 0.04 (Cyclohexane/EtOAc, 9:1); ν_{max}/cm^{-1} (thin film) 2926, 2854, 1499, 1054; ^1H NMR (400 MHz, $CDCl_3$) δ 8.85 (1H, dd, *J* = 4.3, 1.7 Hz), 8.11–8.01 (2H, m), 7.65–7.55 (2H, m), 7.35 (1H, dd, *J* = 8.2, 4.2 Hz), 4.17 (1H, dd, *J* = 8.6, 7.4 Hz), 4.10 (1H, ddd, *J* = 8.4, 8.1, 4.7 Hz), 3.93 (1H, ddd, *J* = 8.4, 8.0, 7.8 Hz), 3.83 (1H, dd, *J* = 8.6, 7.3 Hz), 3.57 (1H, dddd, *J* = 8.1, 7.8, 7.5, 7.3 Hz), 2.42 (1H, dddd, *J* = 12.6, 8.1, 8.0, 4.7 Hz), 2.07 (1H, app ddt, *J* = 12.6, 8.1, 7.8 Hz); ^{13}C NMR (101 MHz, $CDCl_3$) δ 149.9, 147.3, 141.2, 135.6, 129.7, 129.3, 128.1, 125.2, 121.2, 74.4, 68.4, 44.8, 34.6; HRMS (ESI-TOF) m/z: [M + H]$^+$ calcd for $C_{13}H_{14}NO$, 200.1070; Found 200.1069 (0.30 ppm error).

7. Diol Scope Studies:

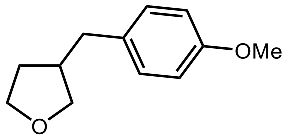

3-(4-Methoxybenzyl)tetrahydrofuran (Compound 10): Prepared according to a modification of general procedure A (0.42 mmol scale) using $NaHCO_3$ (70.6 mg, 0.84 mmol, 2.00 equiv.), $Pd(OAc)_2$ (1.8 mg, 0.01 mmol, 0.02 equiv.), $^nBu_4NCl$ (117 mg, 0.42 mmol, 1.00 equiv.), 4-iodoanisole (98.3 mg, 0.42 mmol, 1.00 equiv.), 2-methylenebutane-1,4-diol **9** (51.1 mg, 0.50 mmol, 1.20 equiv.) in MeCN (2 mL). The crude material was then immediately reduced according to general procedure B using hemiacetal (87.5 mg, 0.42 mmol, 1.00 equiv.), Et_3SiH (73.5 µL, 0.46 mmol, 1.10 equiv.), $BF_3{\cdot}OEt_2$ (56.8 µL, 0.46 mmol, 1.10 equiv.) in DCM (2 mL). Purification by flash column chromatography (SiO_2, Cyclohexane : EtOAc – 15:1) gave the *title compound* **10** (49.5 mg, 0.257 mmol, 61%) as a pale yellow oil; R_f = 0.25 (Cyclohexane/EtOAc, 9:1); ν_{max}/cm^{-1} (thin film) 2930, 2850, 1612, 1511, 1243, 1034; ^{1}H NMR (500 MHz, $CDCl_3$) δ 7.09 (2H, d, *J* = 8.6 Hz), 6.83 (2H, d, *J* = 8.6 Hz), 3.89 (1H, ddd, *J* = 8.3, 7.9, 5.0 Hz), 3.82 (1H, dd, *J* = 8.4, 7.0 Hz), 3.79 (3H, s), 3.76 (1H, ddd, *J* = 8.3, 7.5, 7.4 Hz), 3.45 (1H, dd, *J* = 8.4, 6.9 Hz), 2.68–2.58 (2H, m), 2.53–2.43 (1H, m), 1.99 (1H, dddd, *J* = 12.4, 7.5, 7.4, 5.0 Hz), 1.61 (1H, dddd, *J* = 12.4, 7.9, 7.4, 7.3 Hz); ^{13}C NMR (126 MHz, $CDCl_3$) δ 157.9, 132.9, 129.5, 113.8, 73.0, 67.9, 55.2, 41.1, 38.4, 32.1; HRMS (ESI-TOF) m/z: [M + Na]$^+$ calcd for $C_{12}H_{16}NaO_2$, 215.1043; Found 215.1041 (0.60 ppm error).

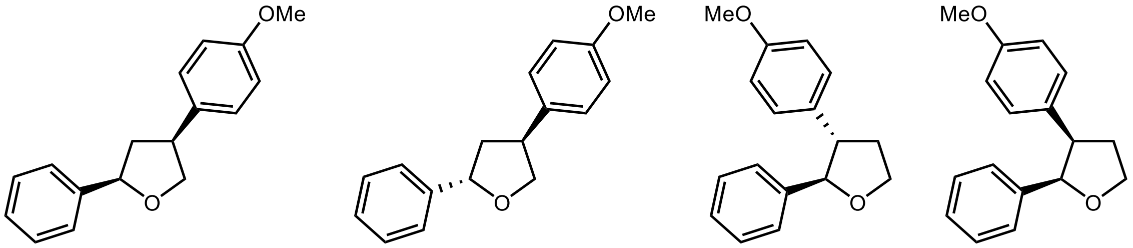

(±)-**12** (±)-**13** (±)-**14** (±)-**15**

(2*R,4*S**)-4-(4-Methoxyphenyl)-2-phenyltetrahydrofuran (Compound 12)**: To a microwave vial containing a mixture of $NaHCO_3$ (126 mg, 1.50 mmol, 2.00 equiv.), $Pd(OAc)_2$ (18 mg, 0.08 mmol, 0.10 equiv.), $^nBu_4NCl$ (208 mg, 0.75 mmol, 1.00 equiv.), and 4-iodoanisole (176 mg, 0.75 mmol, 1.00 equiv.), in MeCN (1.9 mL, 0.4 M) was added (*Z*)-1-phenylbut-2-ene-1,4-diol **11** (148 mg, 0.90 mmol, 1.20 equiv.). The vial was capped, then the reaction was heated at 60 °C in a heating block and was held for 24 h. The reaction mixture was cooled to r.t and diluted with Et_2O (10 mL) and water (15 mL), then the aqueous phase was extracted with Et_2O (3 × 10 mL). The combined organic layers were washed with brine (15 mL), dried ($MgSO_4$) and concentrated *in vacuo* to afford the ketone intermediate which was immediately reduced using general procedure B using ketone (203 mg, 0.75 mmol, 1.00 equiv.),

Et_3SiH (133 µL, 0.83 mmol, 1.10 equiv.), $BF_3{\cdot}OEt_2$ (102 µL, 0.83 mmol, 1.10 equiv.) in DCM (3.75 mL). Purification by flash column chromatography (SiO_2, Cyclohexane : EtOAc – 15:1) gave an inseparable mixture of the *title compound* **12** and isomeric products **13**, **14**, and **15** (147 mg, 0.576 mmol, 77%, 15.7:3.9:3.2:1.0) as a colourless oil; **Compound 12**: R_f = 0.33 (Cyclohexane/EtOAc, 9:1); ν_{max}/cm^{-1} (thin film) 3032, 2935, 1612, 1584, 1248; ^1H NMR (400 MHz, $CDCl_3$) δ 7.46–7.28 (5H, m), 7.20 (2H, d, *J* = 8.7 Hz), 6.86 (2H, d, *J* = 8.7 Hz), 5.08 (1H, dd, *J* = 10.4, 5.8 Hz), 4.34 (1H, dd, *J* = 8.4, 8.2 Hz), 3.97 (1H, dd, *J* = 8.4, 8.3 Hz), 3.80 (3H, s), 3.60 (1H, dddd, *J* = 10.7, 8.3, 8.2, 7.7 Hz), 2.74 (1H, ddd, *J* = 12.5, 7.7, 5.8 Hz), 1.98 (1H, ddd, *J* = 12.5, 10.7, 10.4 Hz); ^{13}C NMR (101 MHz, $CDCl_3$) δ 158.3, 142.8, 133.5, 128.4, 128.2, 127.4, 125.7, 114.0, 81.8, 75.2, 55.3, 45.3, 43.8; HRMS (ESI-TOF) m/z: $[M + NH_4]^+$ calcd for $C_{17}H_{22}NO_2$, 272.1645; Found 272.1638 (2.60 ppm error).

Data are consistent with those reported in the literature.[15]

Resolved signals for the minor isomers are reported below.

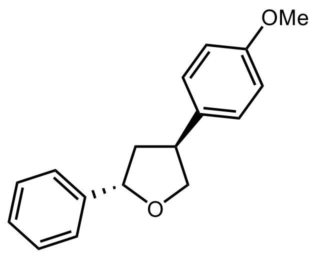

(2*S,4*S**)-4-(4-Methoxyphenyl)-2-phenyltetrahydrofuran (Compound 13):** ^1H NMR (400 MHz, $CDCl_3$) δ 7.46–7.28 (5H, m), 7.25–7.22 (2H, m), 6.85–6.83 (2H, m), 5.23 (1H, dd, *J* = 7.8, 5.7 Hz), 4.45 (1H, dd, *J* = 8.4, 7.5 Hz), 3.91 (1H, dd, *J* = 8.4, 8.1 Hz), 3.81–3.78 (3H, m), 3.50 (1H, dddd, *J* = 8.1, 7.9, 7.8, 7.5 Hz), 2.54–2.38 (1H, m), 2.36–2.17 (1H, m).

Data are consistent with those reported in the literature.[15]

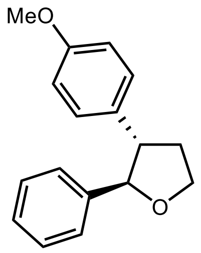

(2*R,3*S**)-3-(4-Methoxyphenyl)-2-phenyltetrahydrofuran (Compound 14)**

^1H NMR (400 MHz, $CDCl_3$) δ 7.10 (2H, d, *J* = 8.7 Hz), 6.88–6.83 (2H, m), 4.78 (1H, d, *J* = 8.5 Hz), 4.27–4.21 (2H, m), 3.81–3.78 (3H, m), 3.17 (1H, app q, *J* = 8.6 Hz), 2.49–2.38 (1H, m), 2.36–2.18 (1H, m).

Data are consistent with those reported in the literature.[16]

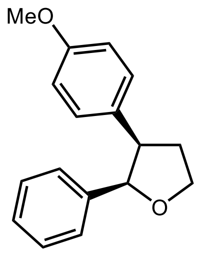

(2*R,3*R**)-3-(4-Methoxyphenyl)-2-phenyltetrahydrofuran (Compound 15)**

^{1}H NMR (400 MHz, $CDCl_3$) δ 5.16 (1H, d, *J* = 6.8 Hz).

8. Synthesis and Derivatization of Hemiacetal 6b:

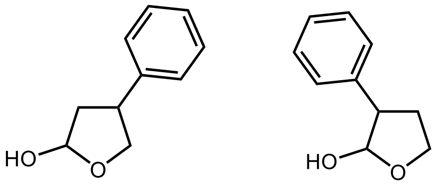

6b S3

4-Phenyltetrahydrofuran-2-ol (Compounds 6b): Prepared according to general procedure A using $NaHCO_3$ (2.05 g, 24.4 mmol, 2.00 equiv.), $Pd(OAc)_2$ (53.9 mg, 240 µmol, 0.02 equiv.), $^{n}Bu_4NCl$ (3.39 g, 12.2 mmol, 1.00 equiv.), iodobenzene (1.37 mL, 12.2 mmol, 1.00 equiv.), *cis*-2-butene-1,4-diol (1.20 mL, 14.6 mmol, 1.20 equiv.) in MeCN (31 mL). Purification by flash column chromatography (SiO_2, Cyclohexane : EtOAc – 4:1 → 3:1) gave the *title compound* **6b** (1.97 g, 12.0 mmol, 98%, d.r. 1.8:1) as a colourless oil; R_f = 0.35 (Cyclohexane/EtOAc, 1:1); ν_{max}/cm^{-1} (thin film) 3397, 2948, 2883, 983, 697; **Major Diastereoisomer:** ^1H NMR (400 MHz, $CDCl_3$) δ 7.37–7.30 (3H, m), 7.30–7.20 (2H, m), 5.77–5.69 (1H, m), 4.47 (1H, dd, *J* = 8.1, 7.9 Hz), 3.85 (1H, dd, *J* = 8.1, 8.0 Hz), 3.75 (1H, dddd, *J* = 9.7, 8.0, 7.9, 7.5 Hz), 2.37 (1H, ddd, *J* = 12.8, 7.5, 1.0 Hz), 2.14 (1H, ddd, *J* = 12.8, 9.7, 5.0 Hz); **Major Diastereoisomer:** ^{13}C NMR (101 MHz, $CDCl_3$) δ 141.8, 128.6, 127.6, 127.1, 98.9, 74.1, 42.4, 41.8; **Minor Diastereoisomer:** ^1H NMR (400 MHz, $CDCl_3$) δ 7.37–7.30 (3H, m), 7.30–7.20 (2H, m), 5.77–5.69 (1H, m), 4.22 (1H, dd, *J* = 8.5, 8.3 Hz), 3.99 (1H, dd, *J* = 9.8, 8.3 Hz), 3.44 (1H, dddd, *J* = 9.8, 9.5, 9.0, 8.5 Hz), 2.66 (1H, ddd, *J* = 13.4, 9.5, 5.5 Hz), 2.03 (1H, ddd, *J* = 13.4, 9.0, 3.3 Hz); **Minor Diastereoisomer:** ^{13}C NMR (101 MHz, $CDCl_3$) δ 140.8, 128.6, 126.7, 126.6, 99.2, 73.0, 44.5, 41.6; HRMS (ESI-TOF) m/z: $[M + Na]^+$ calcd for $C_{10}H_{12}NaO_2$, 187.0730; Found 187.0734 (2.40 ppm error).

Data are consistent with those reported in the literature.[17]

Note 1: It was not possible to assign the relative stereochemistry of the major and minor diastereoisomers

Note 2: Traces of a regioisomeric species believed to be 2,3-substituted tetrahydrofuran **S3** were evident in the ^1H NMR spectrum.

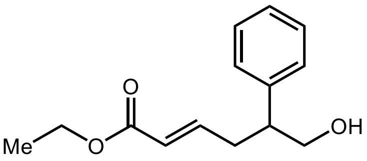

Ethyl (*E*)-6-hydroxy-5-phenylhex-2-enoate (Compound 16): To a solution of 4-phenyltetrahydrofuran-2-ol **6b** (164 mg, 1.00 mmol, 1.00 equiv.) in THF (4 mL) was added (carbethoxymethylene)triphenylphosphorane (383 mg, 1.10 mmol, 1.10 equiv.), the mixture was stirred for 24 h. The reaction was poured into sat. aq. $NaHCO_3$ (10 mL) and extracted with EtOAc (3 × 7 mL),

the combined organics were washed with brine (10 mL), dried ($MgSO_4$) and concentrated *in vacuo* to afford the crude material. Purification by flash column chromatography (SiO_2, Cyclohexane : EtOAc – 5:1) gave the *title compound* **16** (195 mg, 0.832 mmol, 83%) as a colourless oil; R_f = 0.38 (Cyclohexane/EtOAc, 1:1); ν_{max}/cm^{-1} (thin film) 3419, 2930, 1713, 1040; ^1H NMR (400 MHz, $CDCl_3$) δ 7.37–7.30 (2H, m), 7.28–7.18 (3H, m), 6.86 (1H, ddd, *J* = 15.6, 7.6, 7.0 Hz), 5.81 (1H, ddd, *J* = 15.6, 1.6, 1.5 Hz), 4.14 (2H, q, *J* = 7.1 Hz), 3.77 (2H, app d, *J* = 6.4 Hz), 2.97 (1H, app dq, *J* = 8.4, 6.4 Hz), 2.67 (1H, dddd, *J* = 14.8, 7.6, 6.4, 1.5 Hz), 2.54 (1H, dddd, *J* = 14.8, 8.4, 7.0, 1.6 Hz), 1.50 (1H, br s), 1.25 (3H, t, *J* = 7.1 Hz); ^{13}C NMR (101 MHz, $CDCl_3$) δ 166.3, 146.4, 140.9, 128.8, 127.9, 127.1, 123.0, 66.8, 60.2, 47.4, 34.7, 14.2; HRMS (ESI-TOF) m/z: $[M + NH_4]^+$ calcd for $C_{14}H_{22}NO_3$, 252.1594; Found 252.1594 (0.10 ppm error).

Note: Traces of a minor isomeric compound, formed from the regioisomeric 2,3-hemiacetal **S3**, were observed in the $^1H/^{13}C$ NMR spectra of the purified product.

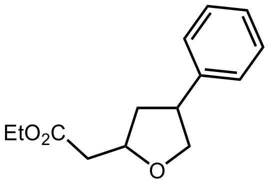

Ethyl 2-(4-phenyltetrahydrofuran-2-yl)acetate (Compound 17): To a solution of ethyl (*E*)-6-hydroxy-5-phenylhex-2-enoate **16** (117 mg, 0.50 mmol, 1.00 equiv.) in THF (2 mL) at r.t was added TBAF (0.65 mL, 0.65 mmol, 1.30 equiv., 1.00 M) for 28 h. The mixture was then concentrated *in vacuo* to afford the crude material. Purification by flash column chromatography (SiO_2, Cyclohexane : EtOAc – 15:1) gave an inseparable mixture of *title compound* **17** (72 mg, 0.306 mmol, 61%, d.r. 1:1) as a colourless oil; R_f = 0.62 (Cyclohexane/EtOAc, 1:1); ν_{max}/cm^{-1} (thin film) 3029, 2978, 1733; **Diastereoisomer 1**: ^1H NMR (400 MHz, $CDCl_3$) δ 7.34–7.28 (2H, m), 7.27–7.20 (3H, m), 4.59 (1H, dddd, *J* = 7.3, 6.9, 6.6, 6.0 Hz), 4.26 (1H, dd, *J* = 8.5, 7.7 Hz), 4.21–4.14 (2H, m), 3.74 (1H, dd, *J* = 8.5, 8.3 Hz), 3.56–3.41 (1H, m), 2.79–2.49 (2H, m), 2.24 (1H, ddd, *J* = 12.8, 7.5, 7.3 Hz), 2.12 (1H, ddd, *J* = 12.8, 8.7, 6.0 Hz), 1.28 (3H, t, *J* = 7.1 Hz); **Diastereoisomer 2:** ^1H NMR (400 MHz, $CDCl_3$) δ 7.34–7.28 (2H, m), 7.27 (3H, m), 4.45 (1H, app ddt, *J* = 9.8, 7.2, 5.7 Hz), 4.21–4.14 (3H, m), 3.83 (1H, dd, *J* = 8.4, 8.3 Hz), 3.56–3.41 (1H, m), 2.79–2.49 (3H, m), 1.76 (1H, ddd, *J* = 12.3, 10.0, 9.8 Hz), 1.28 (3H, t, *J* = 7.1 Hz); ^{13}C NMR (101 MHz, $CDCl_3$) δ 171.14, 171.09, 142.0, 141.8, 128.6 (4 × ArCH), 127.24 (2 × ArCH), 127.15 (2 × ArCH), 126.62, 126.58, 76.2, 75.6, 74.7 , 74.3, 60.54, 60.53, 45.4, 44.4, 41.1, 40.7, 40.6, 39.4, 14.2 (2 × CH_3); HRMS (ESI-TOF) m/z: $[M + H]^+$ calcd for $C_{14}H_{19}O_3$, 235.1329; Found 235.1327 (0.90 ppm error).

Note: It was not possible to assign the ^{13}C signals to the two diastereoisomers. The ^{13}C NMR spectroscopic data are therefore reported as single list.

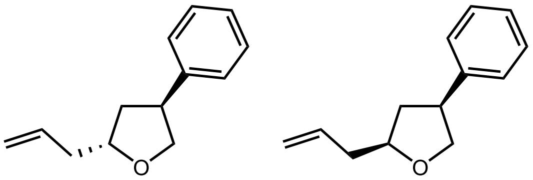

(2*R,4*S**)-2-Allyl-4-phenyltetrahydrofuran and (2*S**,4*S**)-2-allyl-4-phenyltetrahydrofuran (Compound 18 and 18’)**: To a solution of 4-phenyltetrahydrofuran-2-ol **6b** (164 mg, 1.00 mmol, 1.00 equiv.) in DCM (4 mL) at 0 °C was added $BF_3·OEt_2$ (247 µL, 2.00 mmol, 2.00 equiv.) followed by allyltrimethylsilane (318 µL, 2.00 mmol, 2.00 equiv.). The reaction was allowed to warm to r.t and stirred for 24 h. The reaction was poured into sat. aq. $NaHCO_3$ (10 mL) and extracted with DCM (3 × 10 mL), then the combined organics were dried ($MgSO_4$) and concentrated *in vacuo* to afford the crude material. Purification by flash column chromatography (SiO_2, Cyclohexane : EtOAc – 19:1) gave an inseparable mixture of the *title compounds* **18** and **18’** (136 mg, 0.722 mmol, 72%, d.r. 12.6:1) as a colourless oil; R_f = 0.29 (Cyclohexane/EtOAc, 19:1); **Major Diastereoisomer 18:** ^1H NMR (500 MHz, $CDCl_3$) δ 7.34–7.29 (2H, m), 7.27–7.20 (3H, m), 5.87 (1H, app ddt, *J* = 17.1, 10.2, 6.9 Hz), 5.14 (1H, dddd, *J* = 17.1, 1.9, 1.7, 1.6 Hz), 5.10 (1H, app ddt, *J* = 10.2, 1.9, 1.1 Hz), 4.26 (1H, dd, *J* = 8.6, 7.4 Hz), 4.26–4.20 (1H, m), 3.73 (1H, dd, *J* = 8.6, 7.9 Hz), 3.44 (1H, app quint, *J* = 7.7, Hz), 2.45–2.39 (1H, m), 2.36–2.30 (1H, m), 2.15–2.05 (2H, m); **Major Diastereoisomer 18:** ^{13}C NMR (126 MHz, $CDCl_3$) δ 142.5, 134.8, 128.5, 127.3, 126.5, 117.1, 78.7, 74.7, 44.5, 40.4, 39.1.

Data are consistent with those reported in the literature.[18]

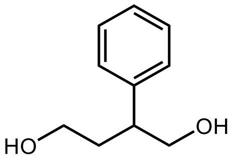

2-Phenylbutane-1,4-diol (Compound 19): To a solution of 4-phenyltetrahydrofuran-2-ol **6b** (164 mg, 1.00 mmol, 1.00 equiv.) in THF:MeOH (3:1, 5 mL) at 0 °C was added $NaBH_4$ (76 mg, 2.00 mmol), then the mixture was stirred for 5 h. The reaction was poured into H_2O (15 mL) and extracted with DCM (3 × 10 mL). The combined organic layers were washed with brine (10 mL) which was then back extracted with DCM (2 × 10 mL). The combined organic layers were dried ($MgSO_4$) and concentrated *in vacuo* to afford the crude material. Purification by filtration through a plug of silica gave the *title compound* **19** (125 mg, 0.751 mmol, 75%) as a colourless oil. R_f = 0.04 (Cyclohexane/EtOAc, 1:1); ^1H NMR (400 MHz, $CDCl_3$) δ 7.36–7.31 (2H, m), 7.27–7.21 (3H, m), 3.79 (2H, d, *J* = 6.6 Hz), 3.70 (1H, ddd, *J* = 10.8, 6.0, 5.6 Hz), 3.59 (1H, ddd, *J* = 10.8, 8.1, 5.4 Hz), 2.97 (1H, dddd, *J* = 8.3, 6.6, 6.5, 6.3 Hz), 2.03 (1H, dddd, *J* = 14.0, 8.1, 6.3, 6.0 Hz), 1.90 (1H, dddd, *J* = 14.0, 8.3, 5.6, 5.4 Hz), 1.72 (2H, br s); ^{13}C NMR (101 MHz, $CDCl_3$) δ 142.4, 128.5, 127.7, 126.6, 67.2, 60.7, 45.7, 35.7.

Data are consistent with those reported in the literature.[19]

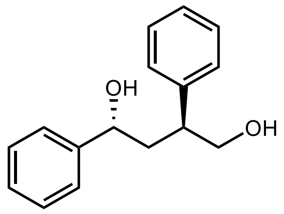

(±)-**20a**

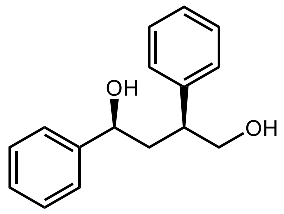

(±)-**20b**

(1*R,3*S**)-1,3-Diphenylbutane-1,4-diol (Compound 20a) and (1*S**,3*S**)-1,3-Diphenylbutane-1,4-diol (Compound 20b)**: To a solution of 4-phenyltetrahydrofuran-2-ol **6b** (500 mg, 3.05 mmol, 1.00 equiv.) in THF (13 mL) at 0 °C was added phenylmagnesium bromide (2.03 mL, 6.10 mmol, 3.00 M in Et_2O) dropwise over 5 min, then the mixture was stirred for 5 h. The reaction was poured into sat. aq. NH_4Cl (20 mL) and extracted with EtOAc (3 × 10 mL). The combined organic layers were washed with brine (10 mL), dried ($MgSO_4$) and concentrated *in vacuo* to afford the crude material. Purification by flash column chromatography (SiO_2, Cyclohexane : EtOAc – 8:1 → 2:1) gave an inseparable mixture of *title compounds* **20a** and **20b** (571 mg, 2.36 mmol, 77%, d.r. 1.5:1) as a colourless oil. R_f = 0.19 (Cyclohexane/EtOAc, 1:1); **Major Diastereoisomer 20a:** ^{1}H NMR (400 MHz, $CDCl_3$) δ 7.38–7.18 (10H, m), 4.57 (1H, dd, *J* = 10.2, 2.8 Hz), 3.80 (2H, app d, *J* = 6.6 Hz), 3.18 (1H, app dq, *J* = 8.6, 6.4 Hz), 2.35–2.06 (1H, m), 2.00 (1H, ddd, *J* = 14.4, 8.6, 2.8 Hz), 1.58 (2H, br s); ^{13}C NMR (101 MHz, $CDCl_3$) δ 145.0, 142.3, 128.8, 128.5, 128.0, 127.6, 126.9, 125.5, 72.7, 67.9, 46.2, 42.8; **Minor Diastereoisomer 20b** ^{1}H NMR (400 MHz, $CDCl_3$) δ 7.38–7.18 (10H, m), 4.65 (1H, app t, *J* = 6.8 Hz), 3.75 (2H, d, *J* = 6.8 Hz), 2.76 (1H, app dtd, *J* = 8.2, 6.8, 6.4 Hz), 2.35–2.06 (4H, m); ^{13}C NMR (101 MHz, $CDCl_3$) δ 144.0, 142.0, 128.8, 128.6, 128.0, 127.8, 127.0, 126.2, 72.7, 67.4, 44.8, 41.5.

Data are consistent with those reported in the literature.[20]

Note: The relative stereochemistry was determined by conversion of the diols into the known *bis*-acetoxylated derivatives following the procedure reported by Oshima.[21]

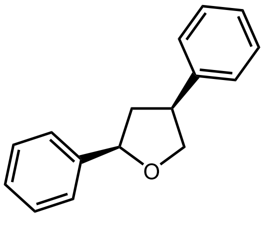

calyxolane B (±)-**2b**

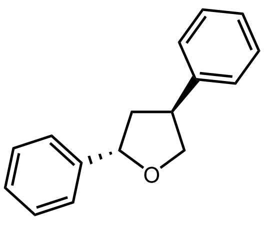

calyxolane A (±)-**2a**

(2*R,4*S**)-2,4-Diphenyltetrahydrofuran (Compound 2b, calyxolane B) and (2*S**,4*S**)-2,4-Diphenyltetrahydrofuran (Compound 2a, calyxolane A)**: To a solution of 1,3-diphenylbutane-1,4-diol **20** (100 mg, 0.413 mmol, 1.00 equiv.) in DCM (4 mL) at −10 °C was added $BF_3{\cdot}OEt_2$ (61.1 µL, 0.495 mmol, 1.20 equiv.) dropwise over 2 min, then the mixture was stirred for 1 h. The reaction was poured

into sat. aq. $NaHCO_3$ (10 mL) and extracted with DCM (3 × 10 mL). The combined organic layers were washed with brine (15 mL), dried ($MgSO_4$) and concentrated *in vacuo* to afford the crude material. Purification by flash column chromatography (SiO_2, Cyclohexane : EtOAc – 48:1) gave an inseparable mixture of *title compound* **2b** and **2a** (80.6 mg, 0.359 mmol, 87%, d.r. 1.7:1) as a colourless oil. R_f = 0.33 (Cyclohexane/EtOAc, 19:1); ν_{max}/cm^{-1} (thin film) 3061, 2863, 1494, 1063, 753, 698; **Major isomer (calyxolane B, 2b):** ^1H NMR (400 MHz, $CDCl_3$) δ 7.45–7.18 (10H, m), 5.11 (1H, dd, *J* = 10.3, 5.7 Hz), 4.40 (1H, dd, *J* = 8.3, 8.1 Hz), 4.06 (1H, dd, *J* = 8.3, 8.2 Hz), 3.67 (1H, dddd, *J* = 10.6, 8.2, 8.1, 7.6 Hz), 2.79 (1H, ddd, *J* = 12.6, 7.6, 5.7 Hz), 2.06 (1H, ddd, *J* = 12.6, 10.6, 10.3 Hz); ^{13}C NMR (101 MHz, $CDCl_3$) δ 142.7, 141.7, 128.6, 128.4, 127.4, 127.2, 126.6, 125.7, 81.8, 75.1, 46.0, 43.7; **Minor isomer (calyxolane A, 2a):** ^1H NMR (400 MHz, $CDCl_3$) δ 7.45–7.18 (10H, m), 5.27 (1H, dd, *J* = 7.8, 5.8 Hz), 4.51 (1H, dd, *J* = 8.3, 7.5 Hz), 3.99 (1H, dd, *J* = 8.3, 8.0 Hz), 3.57 (1H, dddd, *J* = 8.1, 8.0, 7.7, 7.5 Hz), 2.52 (1H, ddd, *J* = 12.5, 7.8, 7.7 Hz), 2.35 (1H, ddd, *J* = 12.5, 8.1, 5.8 Hz); ^{13}C NMR (101 MHz, $CDCl_3$) δ 143.6, 142.0, 128.6, 128.3, 127.3, 127.2, 126.6, 125.5, 80.6, 75.1, 44.4, 42.7; HRMS (ESI-TOF) m/z: $[M + Na]^+$ calcd for $C_{16}H_{16}NaO$, 247.1093; Found 247.1089 (1.60 ppm error).

Data are consistent with those reported in the literature.[22]

Note: Traces of a minor isomeric compound, formed from the regioisomeric 2,3-hemiacetal **S3**, were observed in the ^1H NMR spectrum of the purified product.

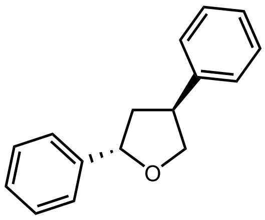

calyxolane A (±)-**2a**

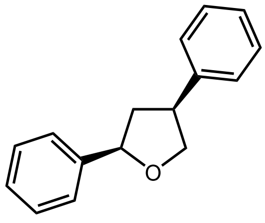

calyxolane B (±)-**2b**

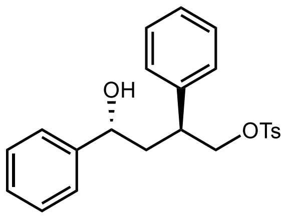

(±)-**21b**

(2*S,4*S**)-2,4-Diphenyltetrahydrofuran (Compound 2a, calyxolane A) and (2*R**,4*S**)-2,4-Diphenyltetrahydrofuran (Compound 2b, calyxolane B)**: To a solution of 1,3-diphenylbutane-1,4-diol **20** (100 mg, 0.413 mmol, 1.00 equiv.) in DCM (4 mL) was added tosyl chloride (157 mg, 0.826 mmol, 2.00 equiv.) and triethylamine (164 µL, 1.18 mmol, 2.85 equiv.), then the mixture was heated to 40 °C in a heating block and stirred for 48 h. The reaction was poured into sat. aq. NH_4Cl (10 mL) and extracted with DCM (3 × 10 mL). The combined organic layers were washed with brine (15 mL), dried ($MgSO_4$) and concentrated *in vacuo* to afford the crude material. Purification by flash column chromatography (SiO_2, Cyclohexane : EtOAc – 48:1) gave an inseparable mixture of *title compounds* **2a** and **2b** (48.7 mg, 0.217 mmol, 53%, d.r. 2.2:1) as a colourless oil and the tosylated species **21b** (39.6 mg, 99.9 µmol) as a colourless crystalline solid.

The characterisation data for compound **2a** and **2b** are consistent with those reported above.

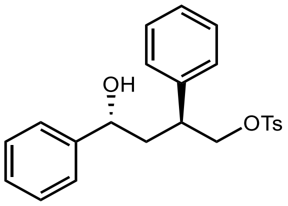

(2*S,4*R**)-4-Hydroxy-2,4-diphenylbutyl 4-methylbenzenesulfonate (Compound 21b)**: R_f = 0.29 (Cyclohexane/EtOAc, 2:1); ν_{max}/cm^{-1} (thin film) 3029, 2924, 1697, 1598, 1494, 1357, 1173; ^{1}H NMR (400 MHz, $CDCl_3$) δ 7.64 (2H, d, *J* = 8.3 Hz), 7.34–7.13 (12H, m), 4.37 (1H, dd, *J* = 10.3, 3.0 Hz), 4.17 (1H, dd, *J* = 9.6, 6.8 Hz), 4.12 (1H, dd, *J* = 9.6, 6.6 Hz), 3.35 (1H, dddd, *J* = 10.8, 6.8, 6.6, 4.3 Hz), 2.42 (3H, s), 2.12 (1H, ddd, *J* = 14.2, 10.3, 4.3 Hz), 1.89 (1H, ddd, *J* = 14.2, 10.8, 3.0 Hz), 1.81 (1H, br s); ^{13}C NMR (101 MHz, $CDCl_3$) δ 144.6, 144.5, 139.6, 132.8, 129.7, 128.7, 128.5, 128.0, 127.8, 127.6, 127.2, 125.5, 73.9, 71.4, 41.9, 41.2, 21.6; HRMS (ESI-TOF) m/z: $[M + Na]^+$ calcd for $C_{23}H_{24}NaO_4S$, 419.1288; Found 419.1279 (1.90 ppm error).

9. References:

(1) Kim, Y.; Lee, J.; Pae, A.; Cho, Y.; Min, S.-J. Diastereoselective Synthesis of 2,6-Disubstituted 4-(Dimethoxymethyl)Tetra-hydropyrans Using TMSOTf-Promoted Prins-Pinacol Cyclization. *Synlett* **2013**, *24* (17), 2292–2296.

(2) Robinson, E. R. T.; Frost, A. B.; Elías-Rodríguez, P.; Smith, A. D. Enantioselective Isothiourea-Catalysed Michael-Michael-Lactonisation Cascade Reaction for the Synthesis of δ-Lactones and 1,2,3,4-Substituted Cyclopentanes. *Synthesis* **2017**, *49* (2), 409–423.

(3) Boeckman, R. K.; Thomas, E. W. A Total Synthesis of Dl-Cerulenin. *J. Am. Chem. Soc.* **1979**, *101* (4), 987–994.

(4) Burks, H. E.; Kliman, L. T.; Morken, J. P. Asymmetric 1,4-Dihydroxylation of 1,3-Dienes by Catalytic Enantioselective Diboration. *J. Am. Chem. Soc.* **2009**, *131* (26), 9134–9135.

(5) Verlinden, S.; Ballet, S.; Verniest, G. Synthesis of Heterocycle-Bridged Peptidic Macrocycles through 1,3-Diyne Transformations. *Eur. J. Org. Chem.* **2016**, *2016* (35), 5807–5812.

(6) Viswanadham, B.; Mahomed, A. S.; Friedrich, H. B.; Singh, S. Efficient and Expeditious Chemoselective BOC Protection of Amines in Catalyst and Solvent-Free Media. *Res. Chem. Intermed.* **2017**, *43* (3), 1355–1363.

(7) Renault, K.; Debieu, S.; Richard, J.-A.; Romieu, A. Deeper Insight into Protease-Sensitive "Covalent-Assembly" Fluorescent Probes for Practical Biosensing Applications. *Org. Biomol. Chem.* **2019**, *17* (39), 8918–8932.

(8) Denis, J. G.; Franci, G.; Altucci, L.; Aurrecoechea, J. M.; de Lera, Á. R.; Álvarez, R. Synthesis of 7-Alkylidene-7,12-Dihydroindolo[3,2-*d*]Benzazepine-6-(5*H*)-Ones (7-Alkylidene-Paullones) by N-Cyclization–Oxidative Heck Cascade and Characterization as Sirtuin Modulators. *Org. Biomol. Chem.* **2015**, *13* (9), 2800–2810.

(9) Kinsinger, T.; Kazmaier, U. Mono-Selective β-C–H Arylation of N -Methylated Amino Acids and Peptides Promoted by the 2-(Methylthio)Aniline Directing Group. *Org. Biomol. Chem.* **2019**, *17* (22), 5595–5600.

(10) Li, C.; Kawamata, Y.; Nakamura, H.; Vantourout, J. C.; Liu, Z.; Hou, Q.; Bao, D.; Starr, J. T.; Chen, J.; Yan, M.; Baran, P. S. Electrochemically Enabled, Nickel-Catalyzed Amination. *Angew. Chem. Int. Ed.* **2017**, *56* (42), 13088–13093.

(11) Li, H.; Liu, Y.; Chiba, S. Anti-Markovnikov Hydroarylation of Alkenes *via* Polysulfide Anion Photocatalysis. *Chem. Commun.* **2021**, *57* (51), 6264–6267.

(12) Asai, S.; Kato, M.; Monguchi, Y.; Sajiki, H.; Sawama, Y. Cyclic Ether Synthesis from Diols Using Trimethyl Phosphate. *Chem. Commun.* **2017**, *53* (35), 4787–4790.

(13) Green, S. A.; Vásquez-Céspedes, S.; Shenvi, R. A. Iron-Nickel Dual-Catalysis: A New Engine for Olefin Functionalization and the Formation of Quaternary Centers. *J. Am. Chem. Soc.* **2018**, *140* (36), 11317–11324.

(14) Molander, G. A.; Traister, K. M.; ONeill, B. T. Reductive Cross-Coupling of Nonaromatic, Heterocyclic Bromides with Aryl and Heteroaryl Bromides. *J. Org. Chem.* **2014**, *79* (12), 5771–5780.

(15) Hilt, G.; Bolze, P.; Harms, K. An Improved Catalyst System for the Iron-Catalyzed Intermodular Ring-Expansion Reactions of Epoxides. *Chem. Eur. J.* **2007**, *13* (15), 4312–4325.

(16) Zuo, Z.; Daniliuc, C. G.; Studer, A. Cooperative NHC/Photoredox Catalyzed Ring-Opening of Aryl Cyclopropanes to 1-Aroyloxylated-3-Acylated Alkanes. *Angew. Chem. Int. Ed.* **2021**, *60* (48), 25252–25257.

(17) Schmitt, A.; Reibig, H.-U. Stereoselective Substitution at Phenyl-Substituted γ-Lactols with Organometallic Compounds. *Chem. Ber.* **1995**, *128* (9), 871–876.

(18) Schmitt, A.; Reißig, H.-U. On the Stereoselectivity of γ-Lactol Substitutions with Allyl- and Propargylsilanes − Synthesis of Disubstituted Tetrahydrofuran Derivatives. *Eur. J. Org. Chem.* **2000**, *2000* (23), 3893–3901.

(19) Ito, M.; Ootsuka, T.; Watari, R.; Shiibashi, A.; Himizu, A.; Ikariya, T. Catalytic Hydrogenation of Carboxamides and Esters by Well-Defined Cp*Ru Complexes Bearing a Protic Amine Ligand. *J. Am. Chem. Soc.* **2011**, *133* (12), 4240–4242.

(20) Chaudhuri, S. K.; Saha, M.; Saha, A.; Bhar, S. Systematic Investigations on the Reduction of 4-Aryl-4-Oxoesters to 1-Aryl-1,4-Butanediols with Methanolic Sodium Borohydride. *Beilstein J. Org. Chem.* **2010**, *6*, 748–755.

(21) Sumida, Y.; Yorimitsu, H.; Oshima, K. Nickel-Catalyzed Borylation of Aryl Cyclopropyl Ketones with Bis(Pinacolato)Diboron to Synthesize 4-Oxoalkylboronates. *J. Org. Chem.* **2009**, *74* (8), 3196–3198.

(22) Rodriguez, A. D.; Cobar, O. M.; Padilla, O. L. The Calyxolanes: New 1,3-Diphenylbutanoid Metabolites Isolated from the Caribbean Marine Sponge *Calyx podatypa*. *J. Nat. Prod.* **1997**, *60* (9), 915–917.

10. Copies of ^{1}H NMR, ^{13}C NMR, and ^{19}F NMR Spectra:

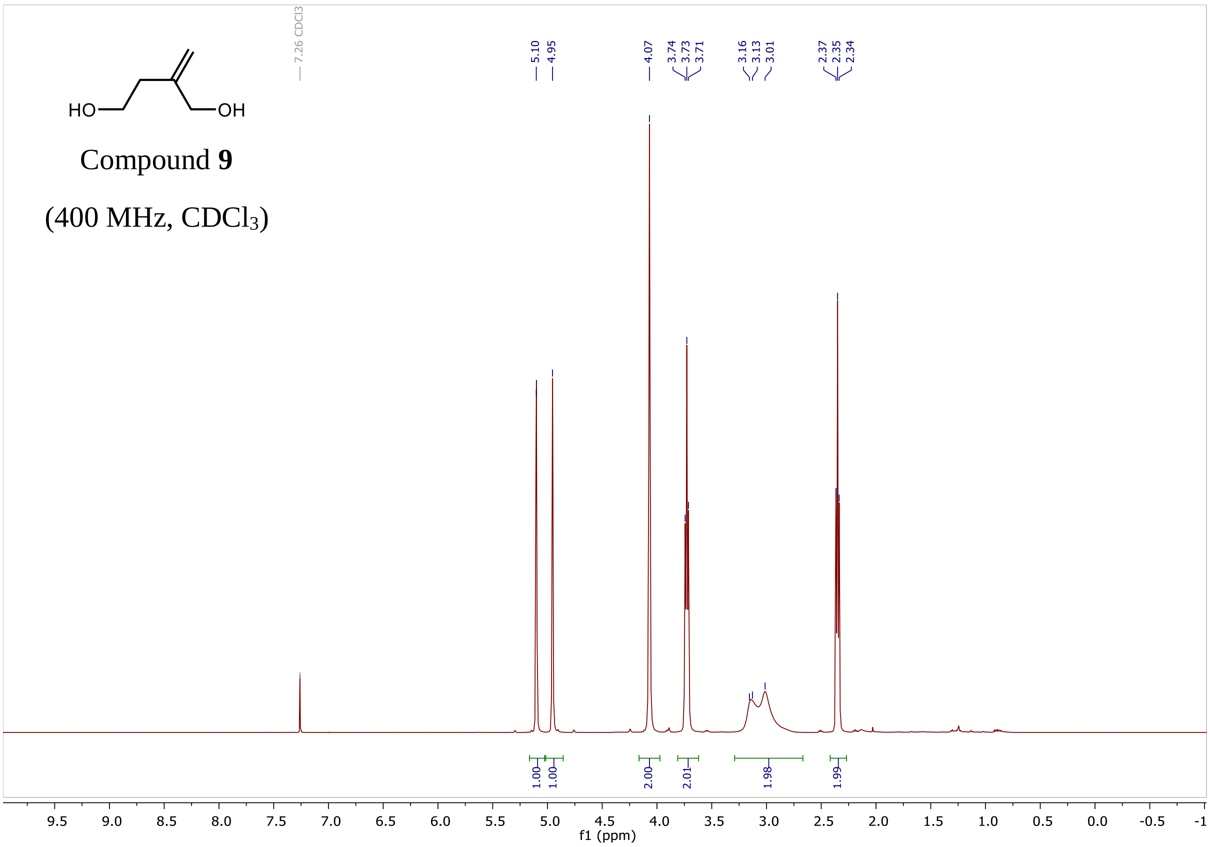

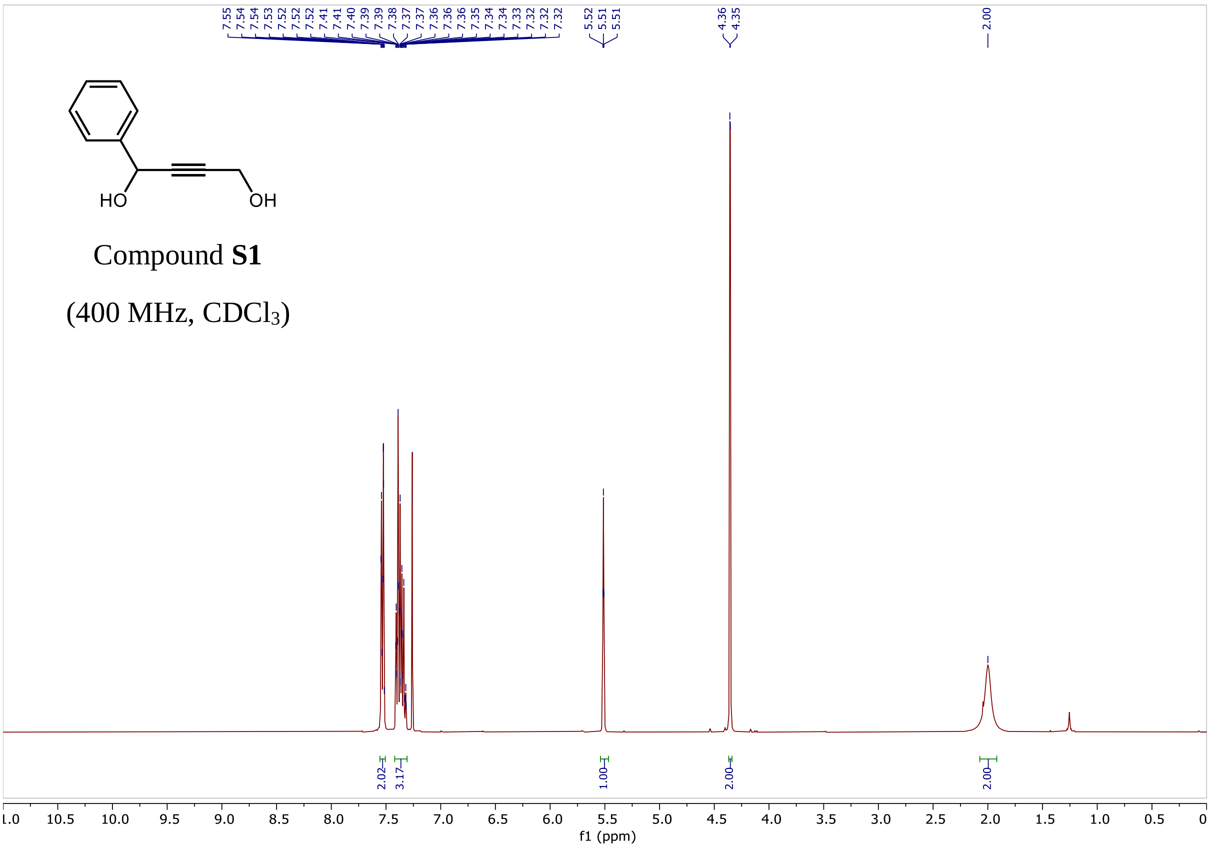

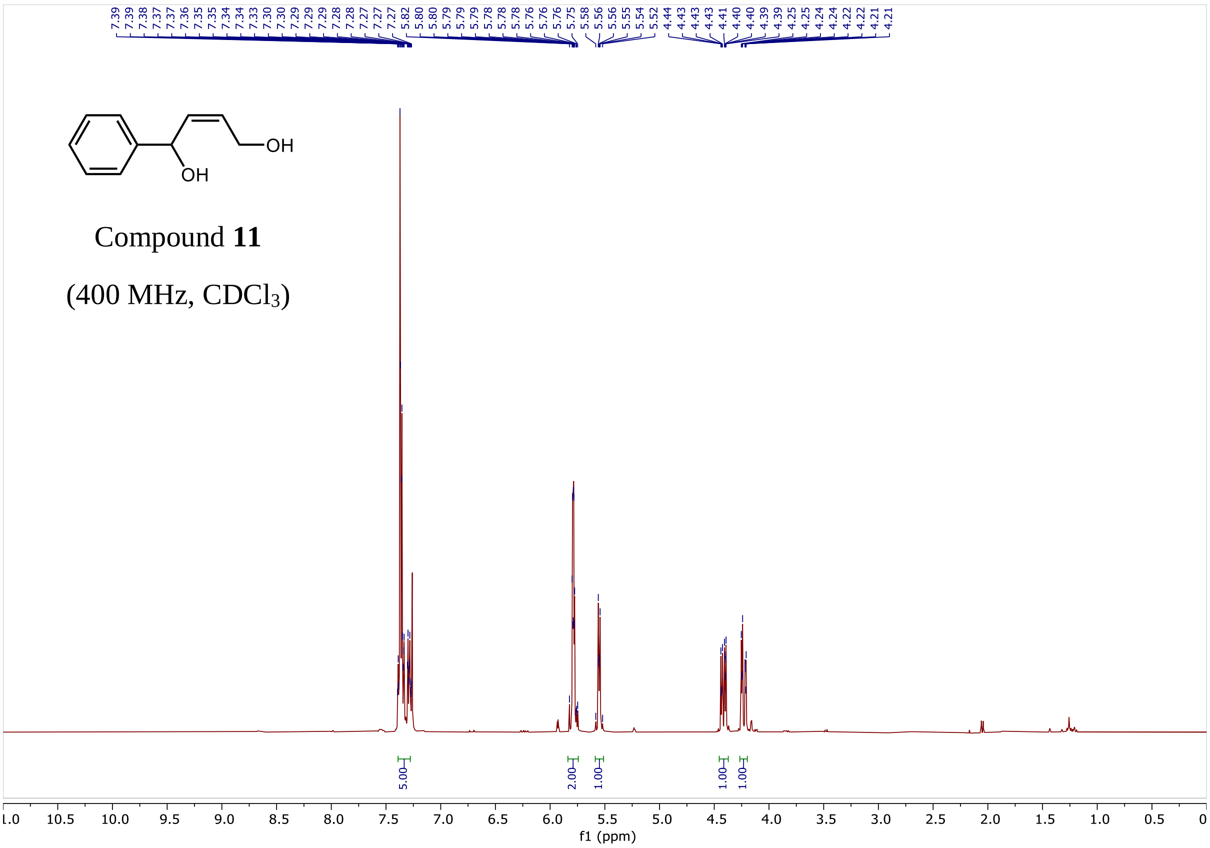
OH
OH
Compound 11
(400 MHz, CDCl3)
f1 (ppm)

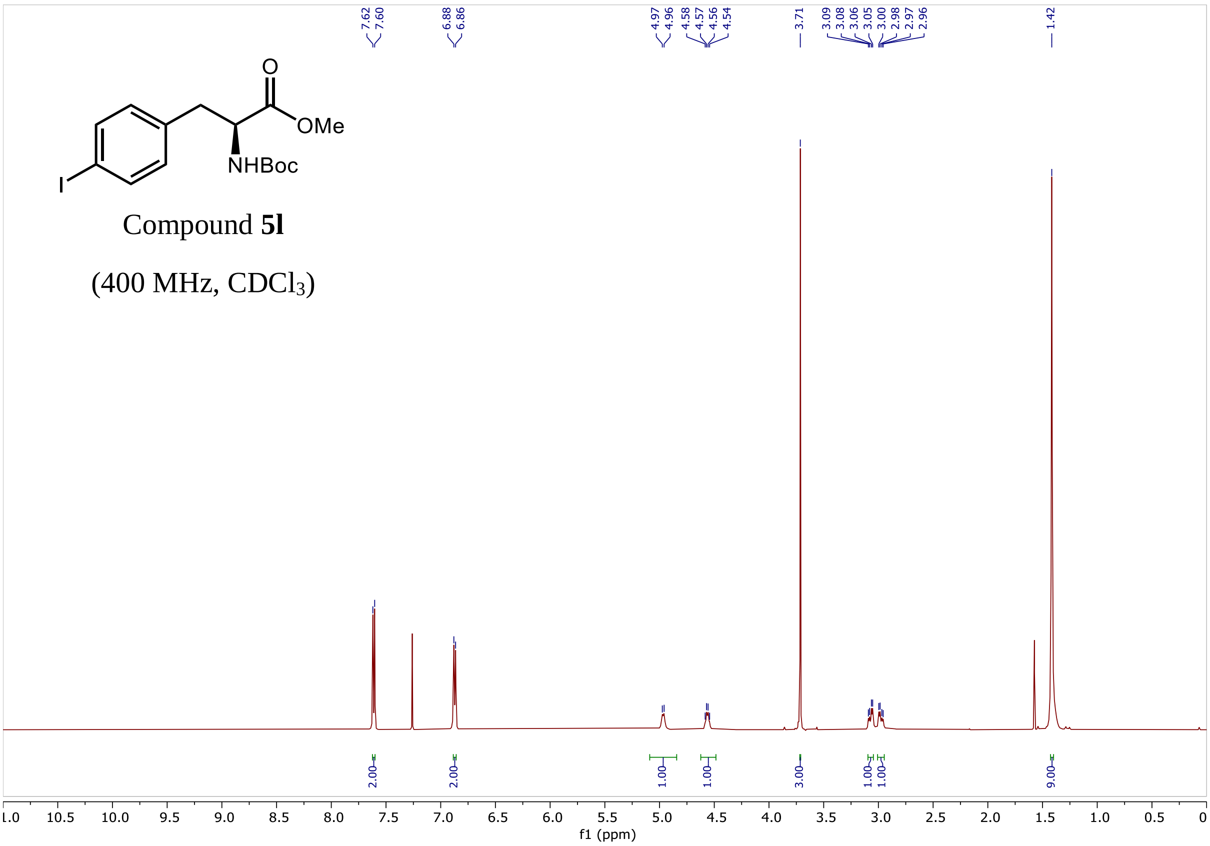
O
OMe
I
NHBoc
Compound 5l
(400 MHz, CDCl3)
f1 (ppm)

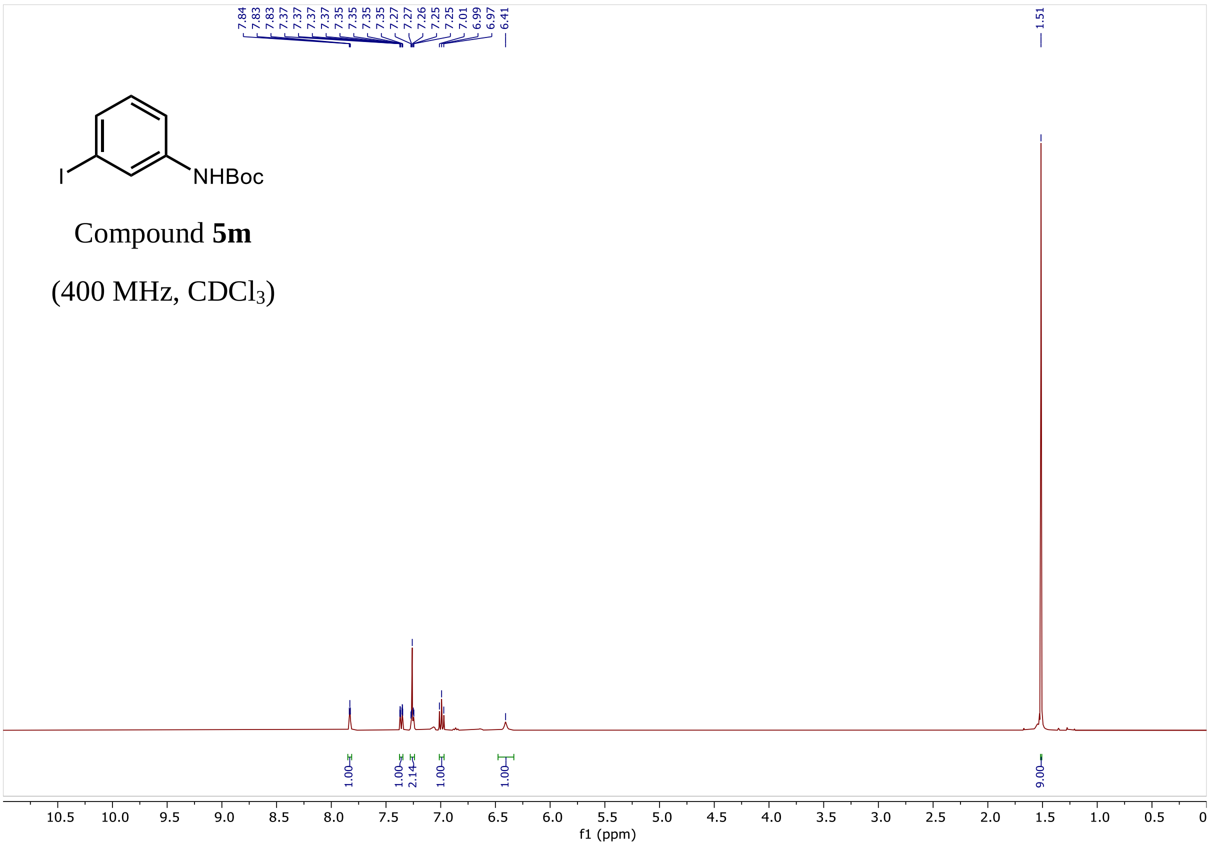
7.84
7.83
7.83
7.37
7.37
7.37
7.37
7.35
7.35
7.35
7.35
7.27
7.27
7.26
7.25
7.25
7.01
6.99
6.97
6.41
1.51
NHBoc
I
Compound **5m**
(400 MHz, $CDCl_3$)
1.00
1.00
2.14
1.00
1.00
9.00
10.5
10.0
9.5
9.0
8.5
8.0
7.5
7.0
6.5
6.0
5.5
5.0
4.5
4.0
3.5
3.0
2.5
2.0
1.5
1.0
0.5
0
f1 (ppm)

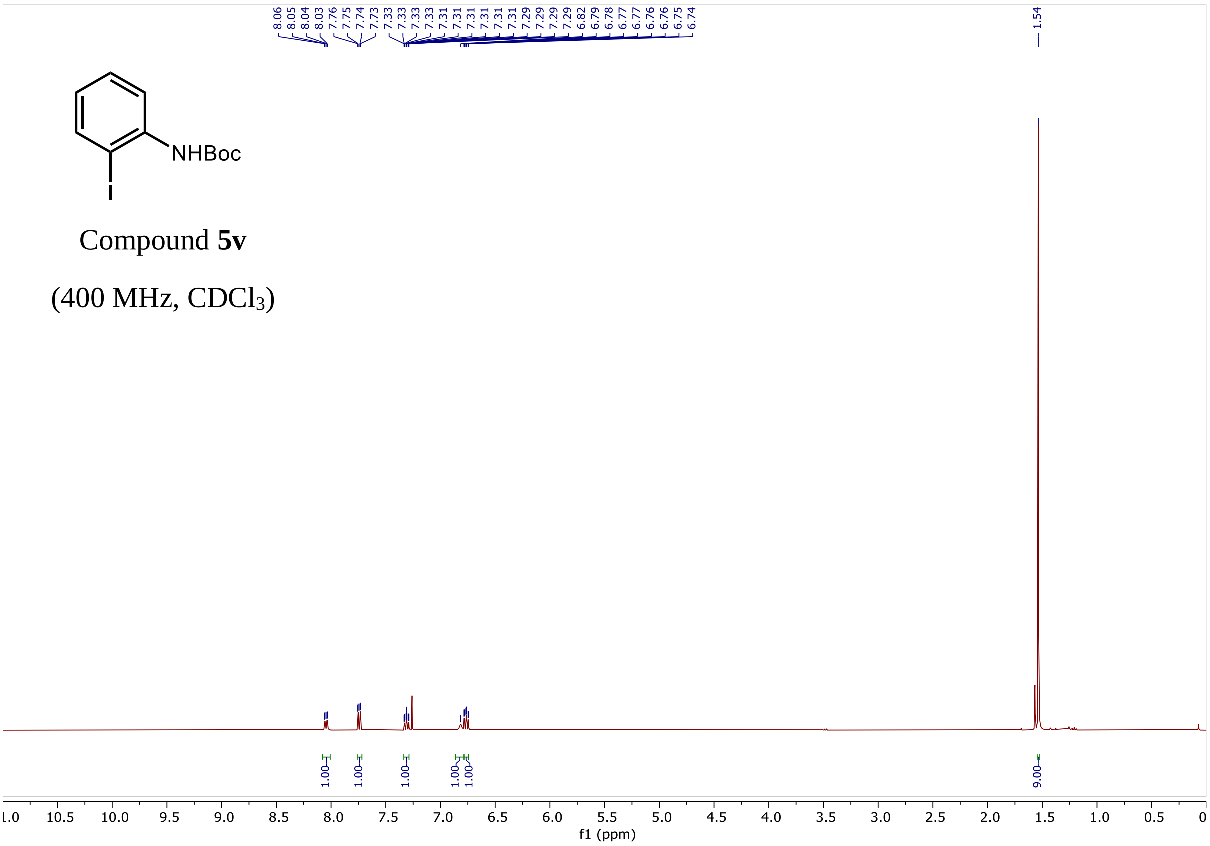
8.06
8.05
8.04
8.03
7.76
7.75
7.74
7.73
7.33
7.33
7.33
7.33
7.31
7.31
7.31
7.31
7.31
7.29
7.29
7.29
7.29
6.82
6.79
6.78
6.77
6.77
6.76
6.76
6.75
6.74
1.54
NHBoc
I
Compound **5v**
(400 MHz, $CDCl_3$)
1.00
1.00
1.00
1.00
1.00
9.00
10.5
10.0
9.5
9.0
8.5
8.0
7.5
7.0
6.5
6.0
5.5
5.0
4.5
4.0
3.5
3.0
2.5
2.0
1.5
1.0
0.5
0
f1 (ppm)

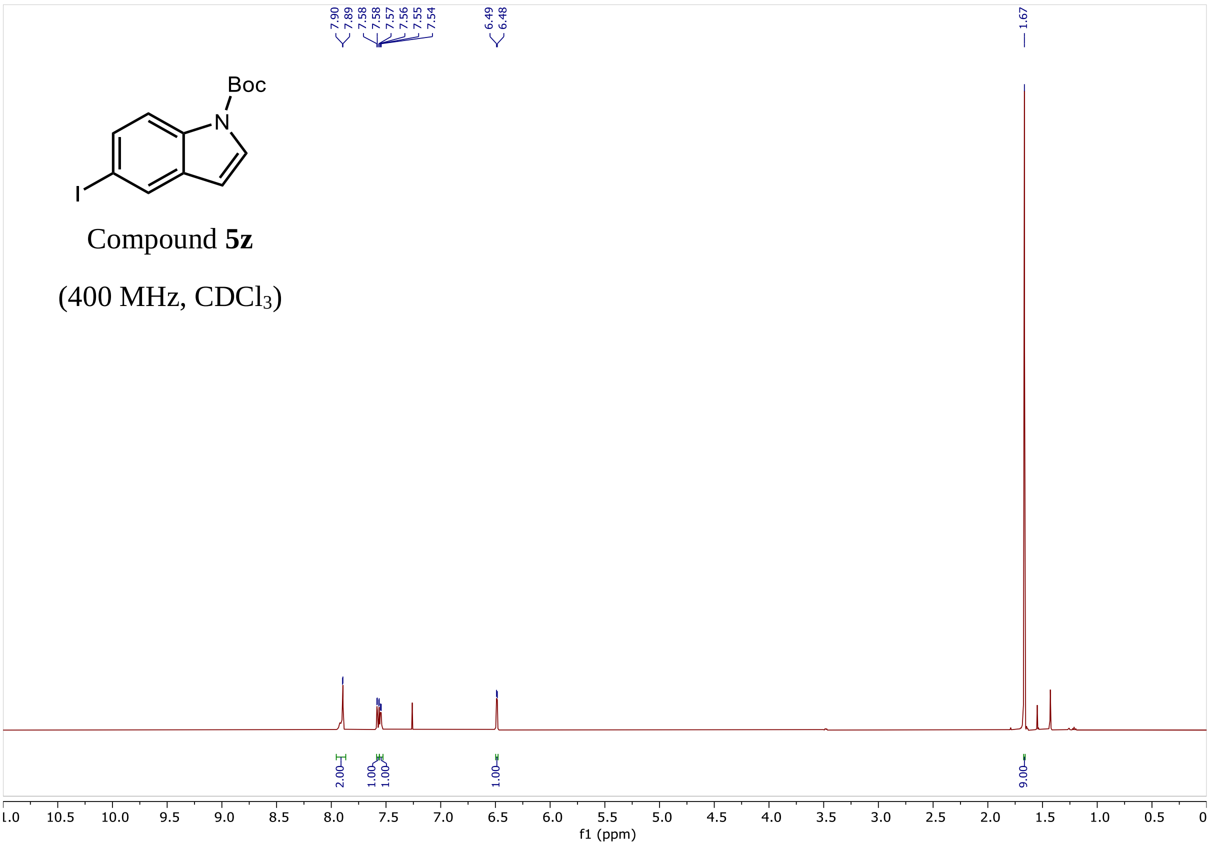
Boc
N
I
Compound **5z**
(400 MHz, $CDCl_3$)
7.90
7.89
7.58
7.58
7.57
7.56
7.55
7.54
6.49
6.48
1.67
2.00
1.00
1.00
1.00
9.00
f1 (ppm)

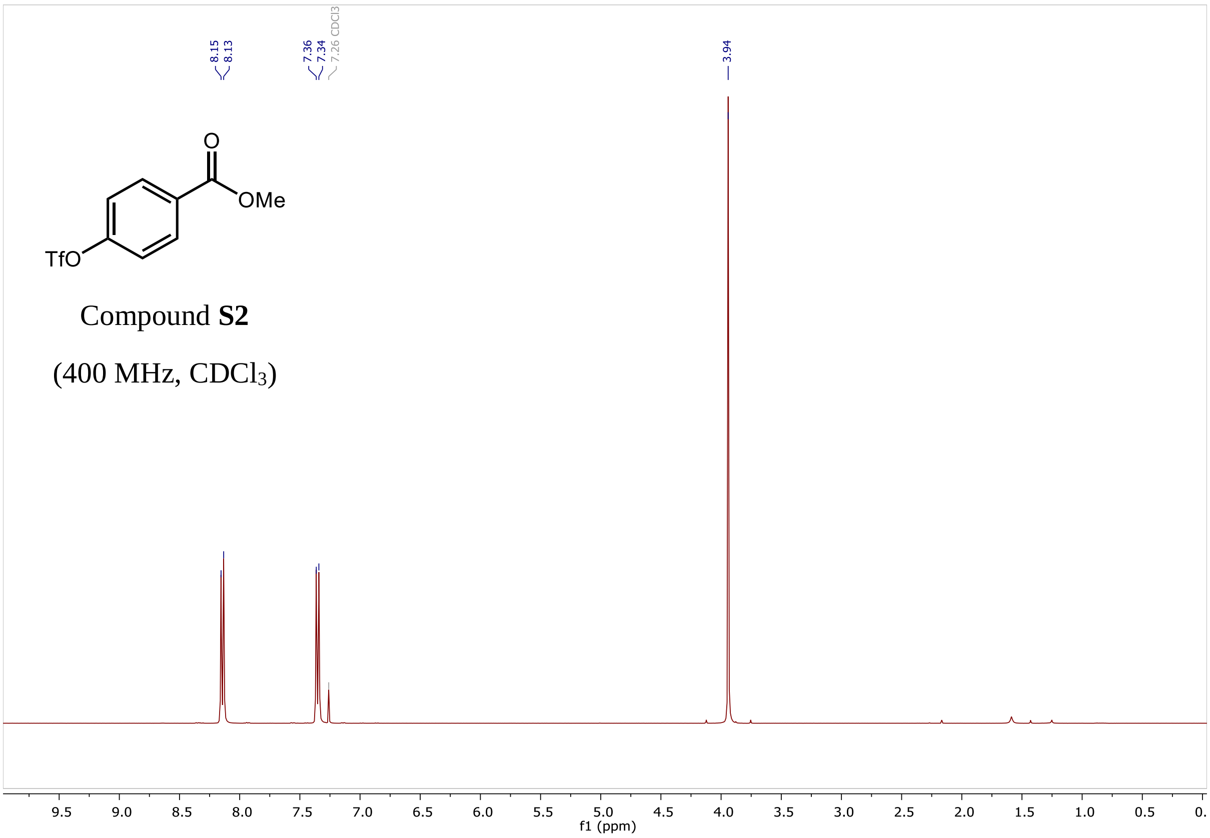
O
OMe
TfO
Compound **S2**
(400 MHz, $CDCl_3$)
8.15
8.13
7.36
7.34
7.26 CDCl3
3.94
f1 (ppm)

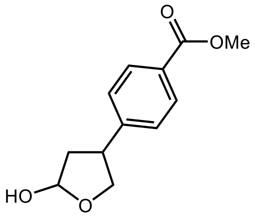

Table 1, Compound **6a**

(400 MHz, $CDCl_3$)

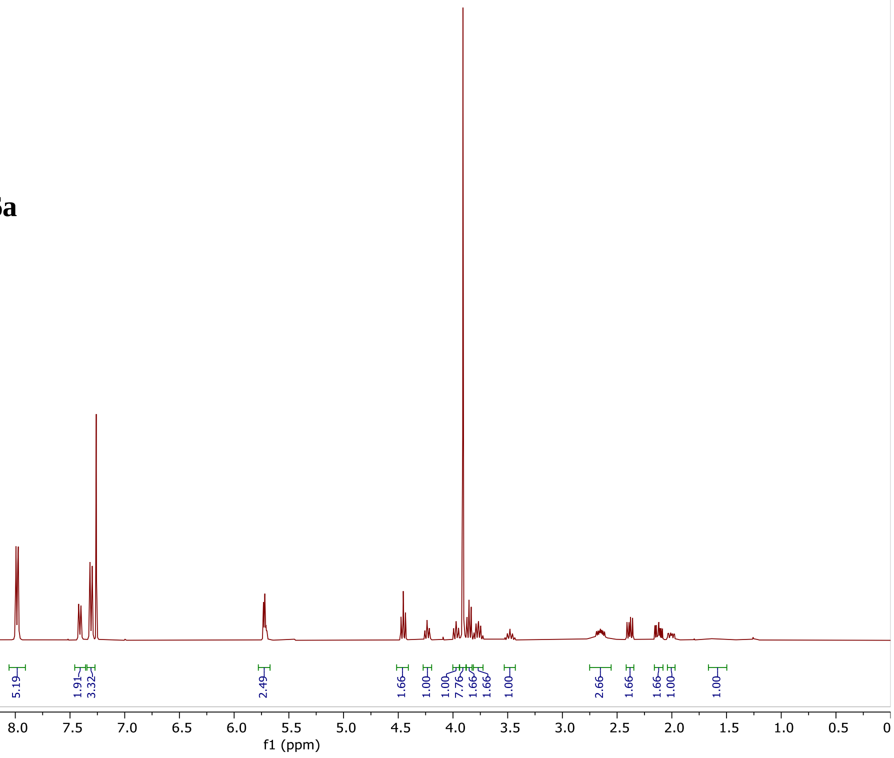

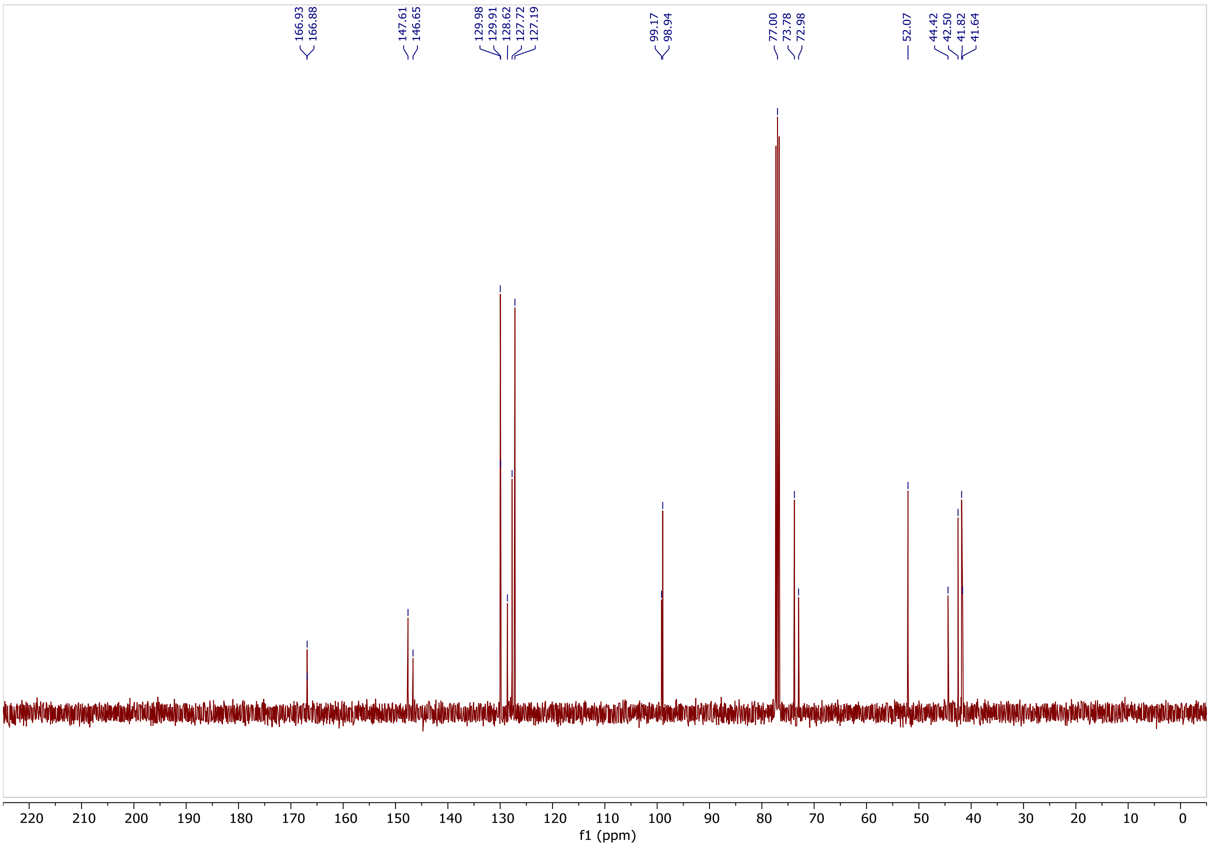

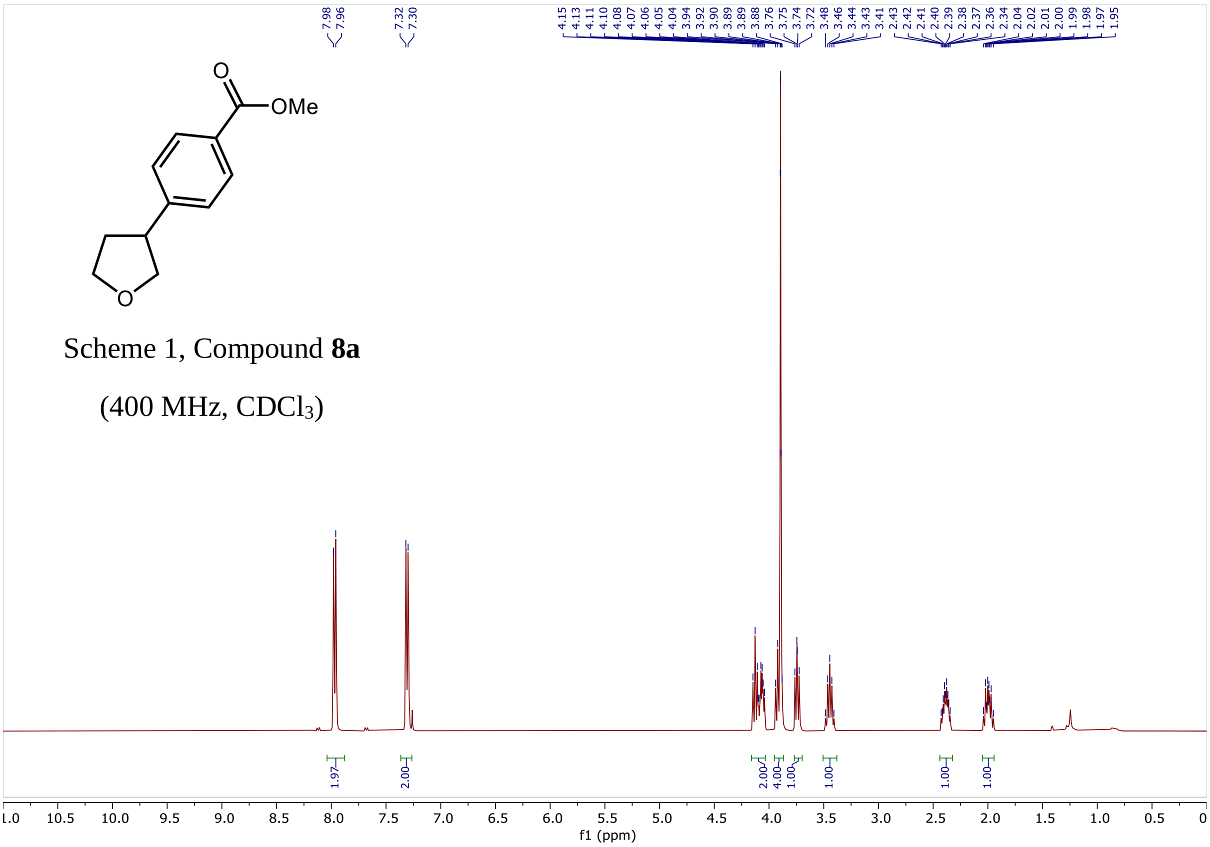

Scheme 1, Compound **8a**

(400 MHz, $CDCl_3$)

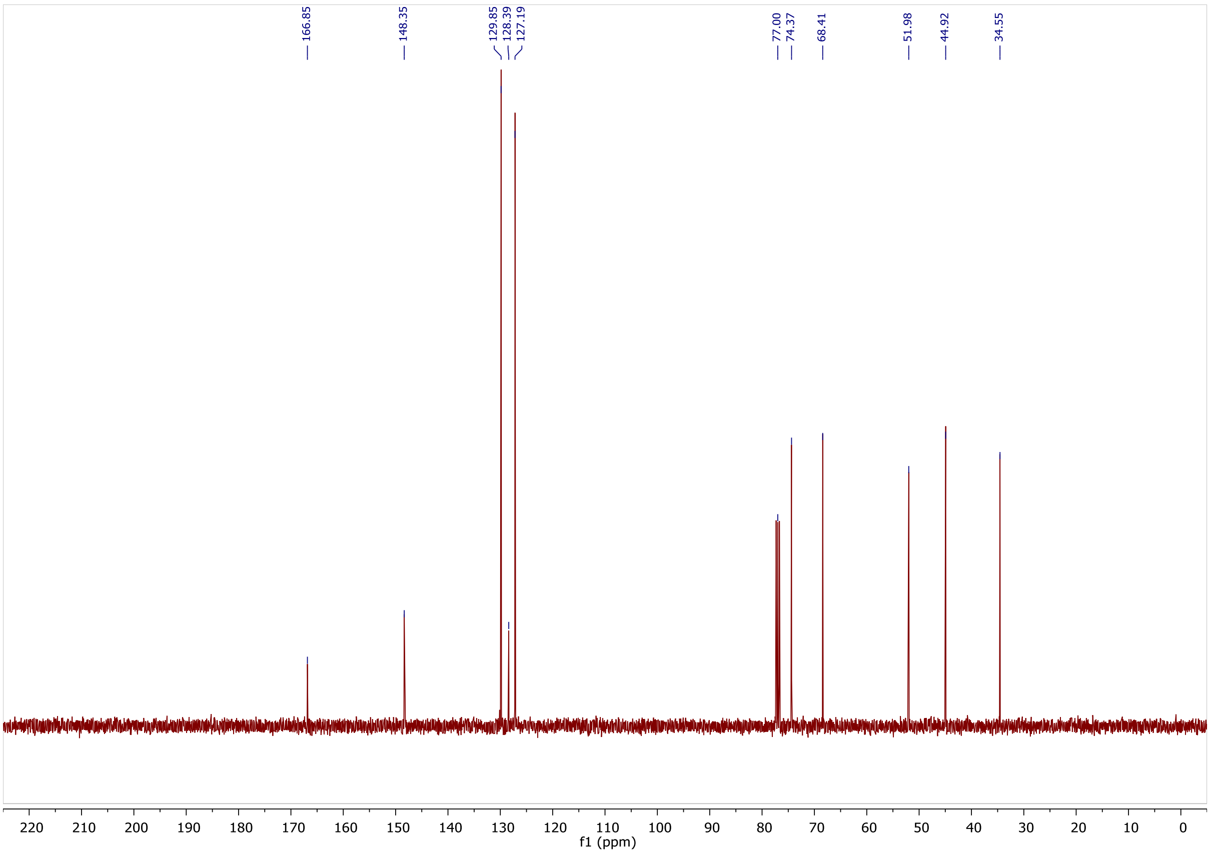

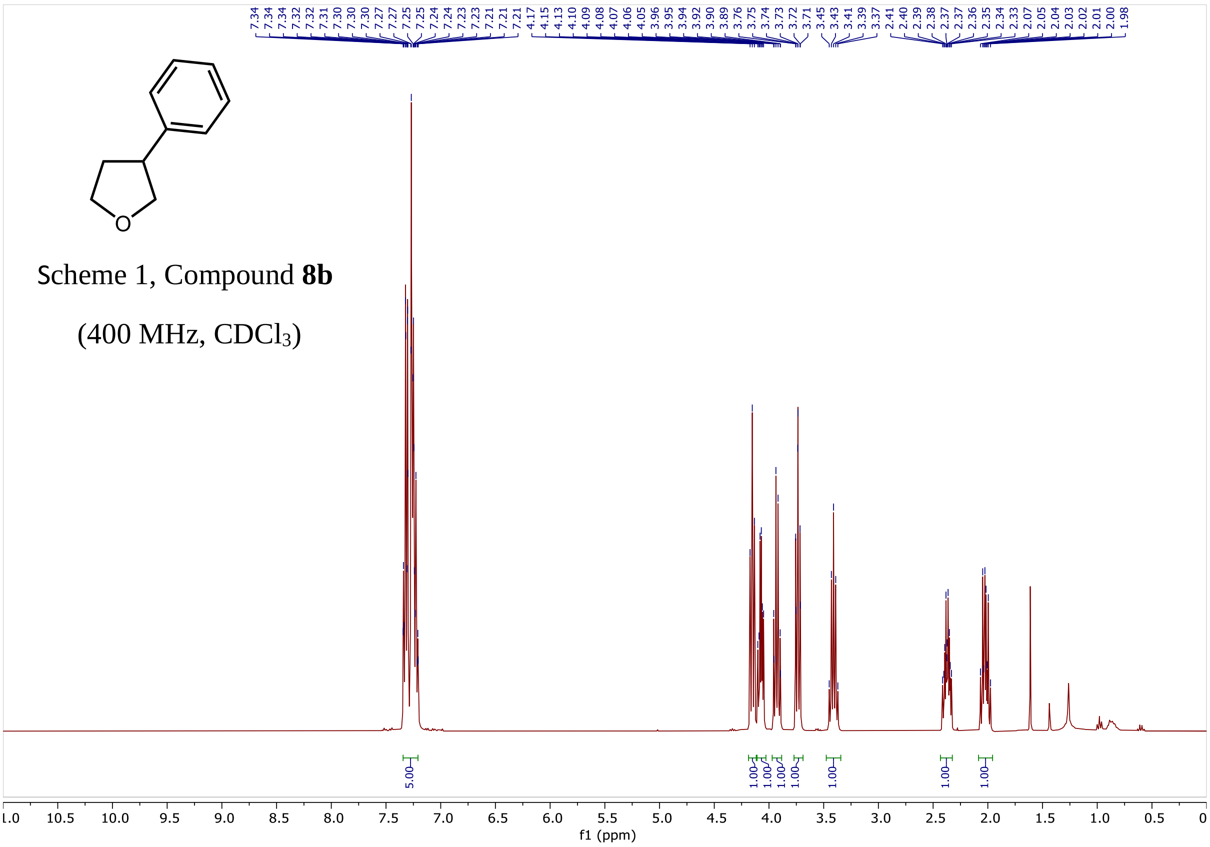
Scheme 1, Compound 8b
(400 MHz, CDCl3)
7.34 7.34 7.34 7.32 7.32 7.31 7.30 7.30 7.30 7.27 7.25 7.25 7.24 7.24 7.23 7.23 7.21 7.21 7.21 4.17 4.15 4.13 4.10 4.09 4.08 4.07 4.06 4.05 3.96 3.95 3.94 3.92 3.90 3.89 3.76 3.75 3.74 3.73 3.72 3.71 3.45 3.43 3.41 3.39 3.37 2.41 2.40 2.39 2.38 2.37 2.37 2.36 2.35 2.34 2.33 2.07 2.05 2.04 2.03 2.02 2.01 2.00 1.98
5.00
1.00
1.00
1.00
1.00
1.00
1.00
1.00
f1 (ppm)

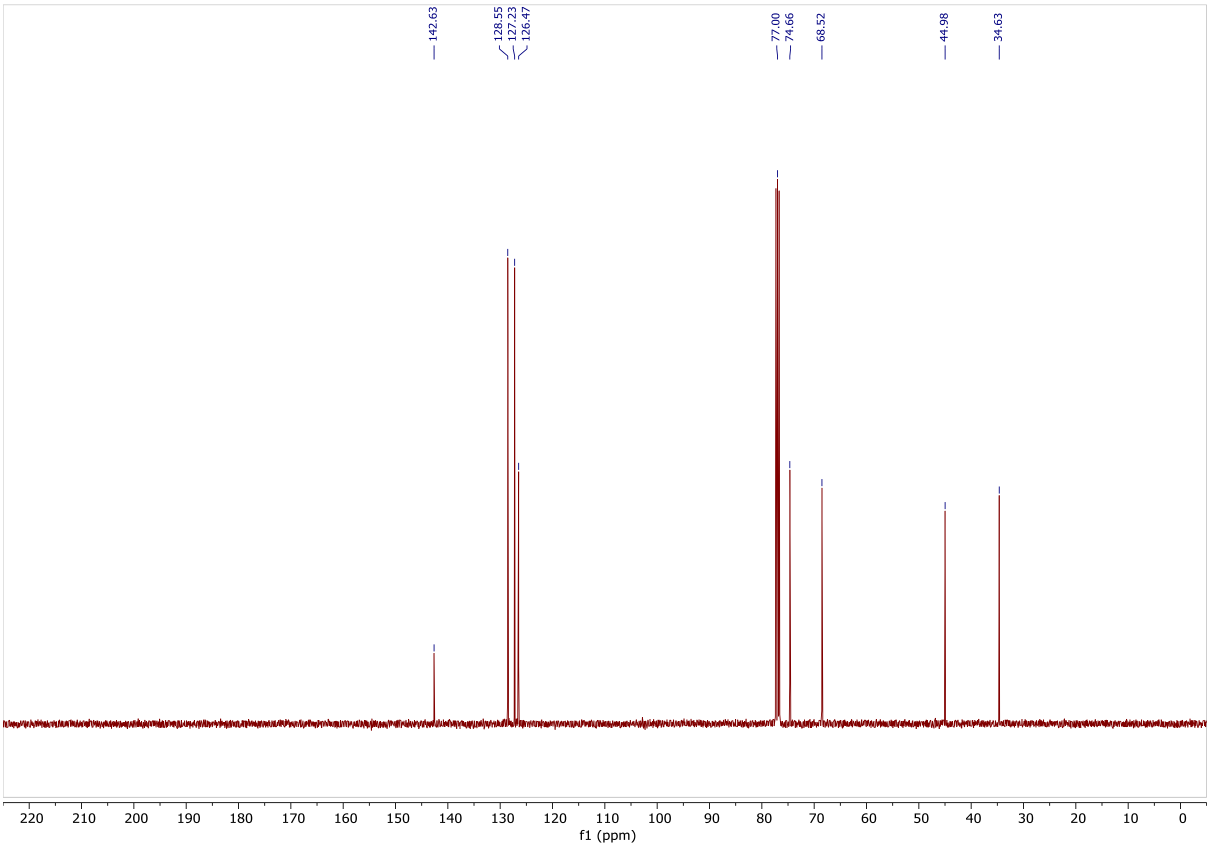
142.63
128.55
127.23
126.47
77.00
74.66
68.52
44.98
34.63
f1 (ppm)

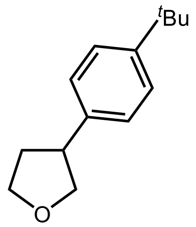

Scheme 1, Compound **8c**

(400 MHz, $CDCl_3$)

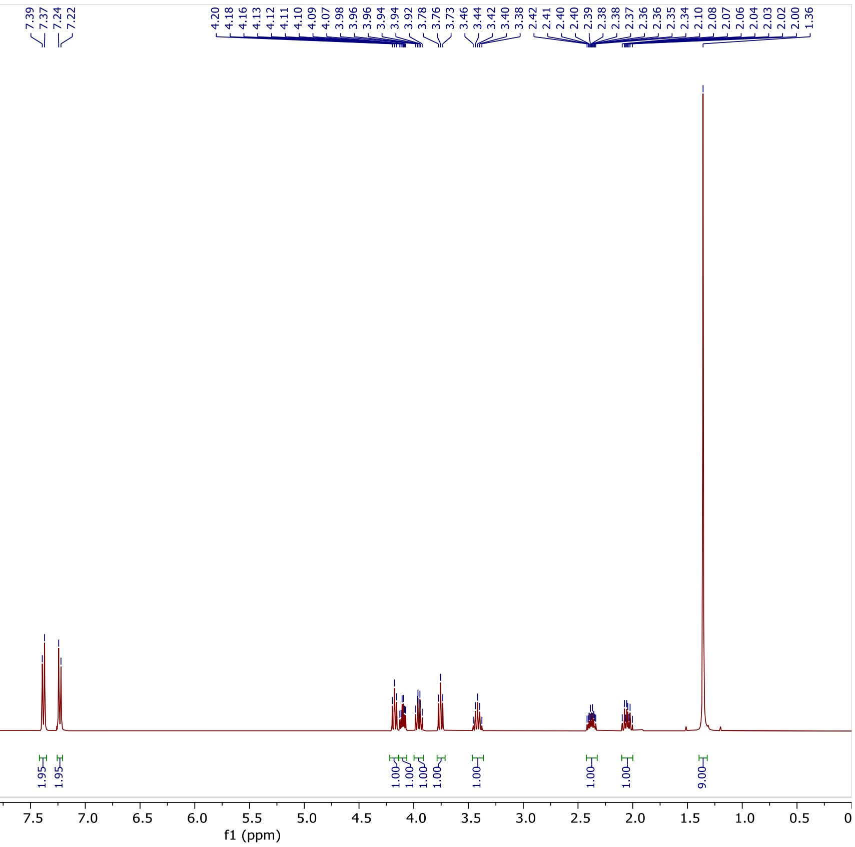

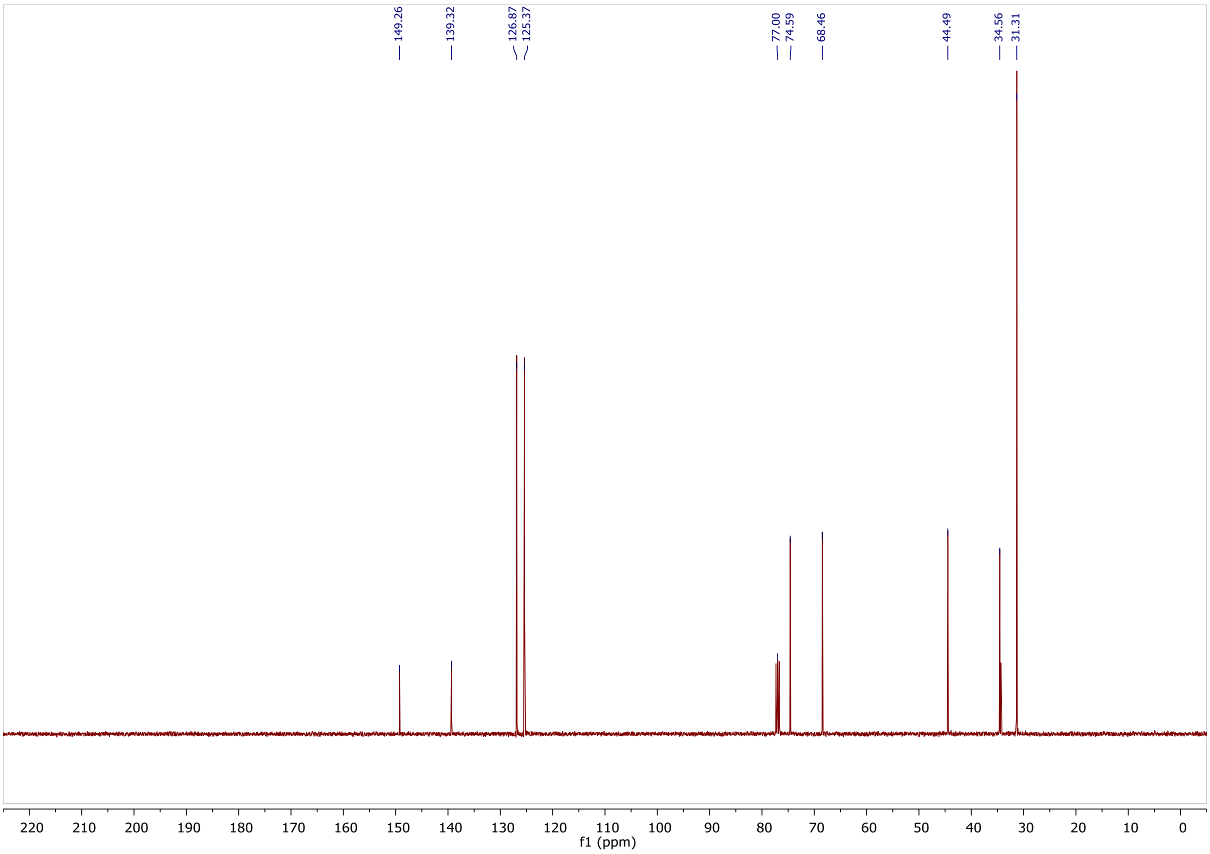

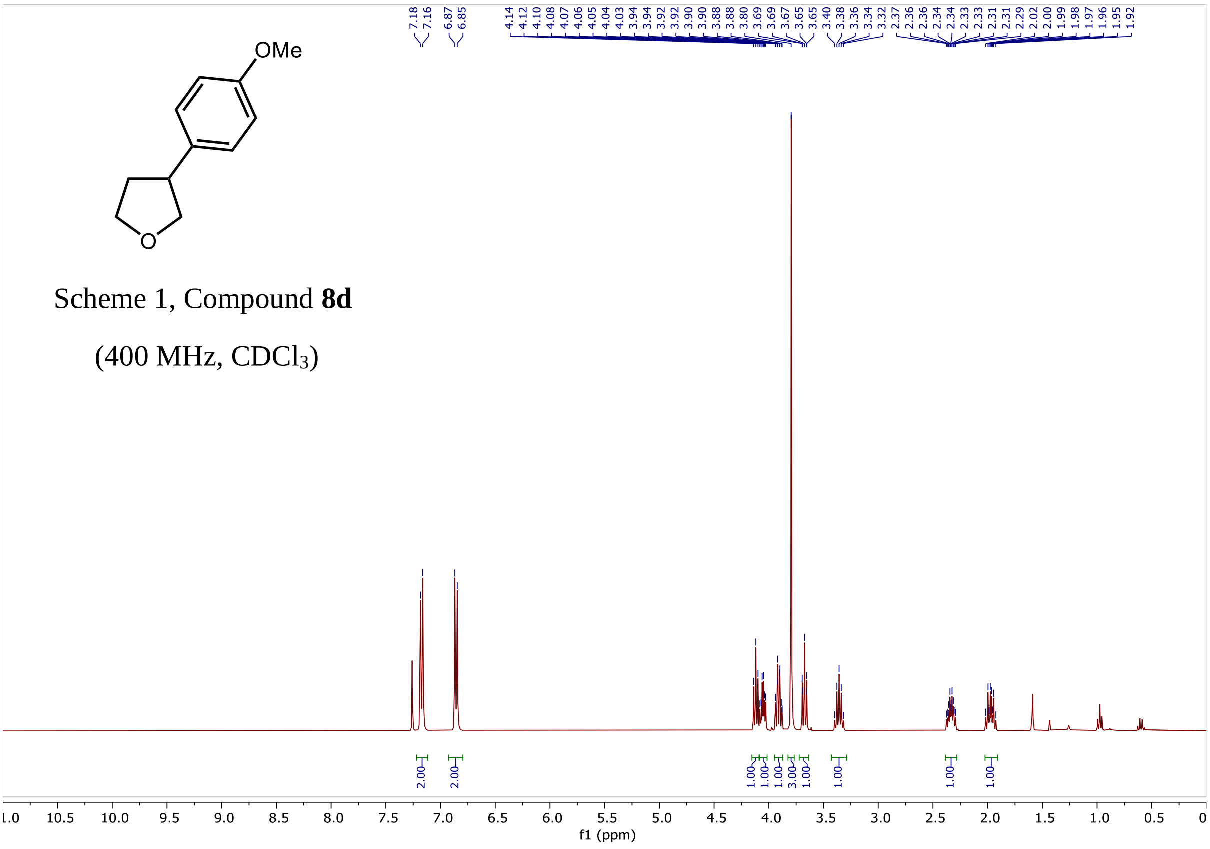
OMe
O
Scheme 1, Compound 8d
(400 MHz, CDCl3)
7.18
7.16
6.87
6.85
4.14
4.12
4.10
4.08
4.07
4.06
4.05
4.04
4.03
3.94
3.94
3.92
3.92
3.90
3.90
3.88
3.88
3.80
3.69
3.69
3.69
3.67
3.65
3.65
3.40
3.38
3.36
3.34
3.32
2.37
2.36
2.36
2.34
2.34
2.33
2.33
2.31
2.31
2.29
2.02
2.00
1.99
1.98
1.97
1.96
1.95
1.92
2.00
2.00
1.00
1.00
1.00
3.00
1.00
1.00
1.00
1.00
f1 (ppm)

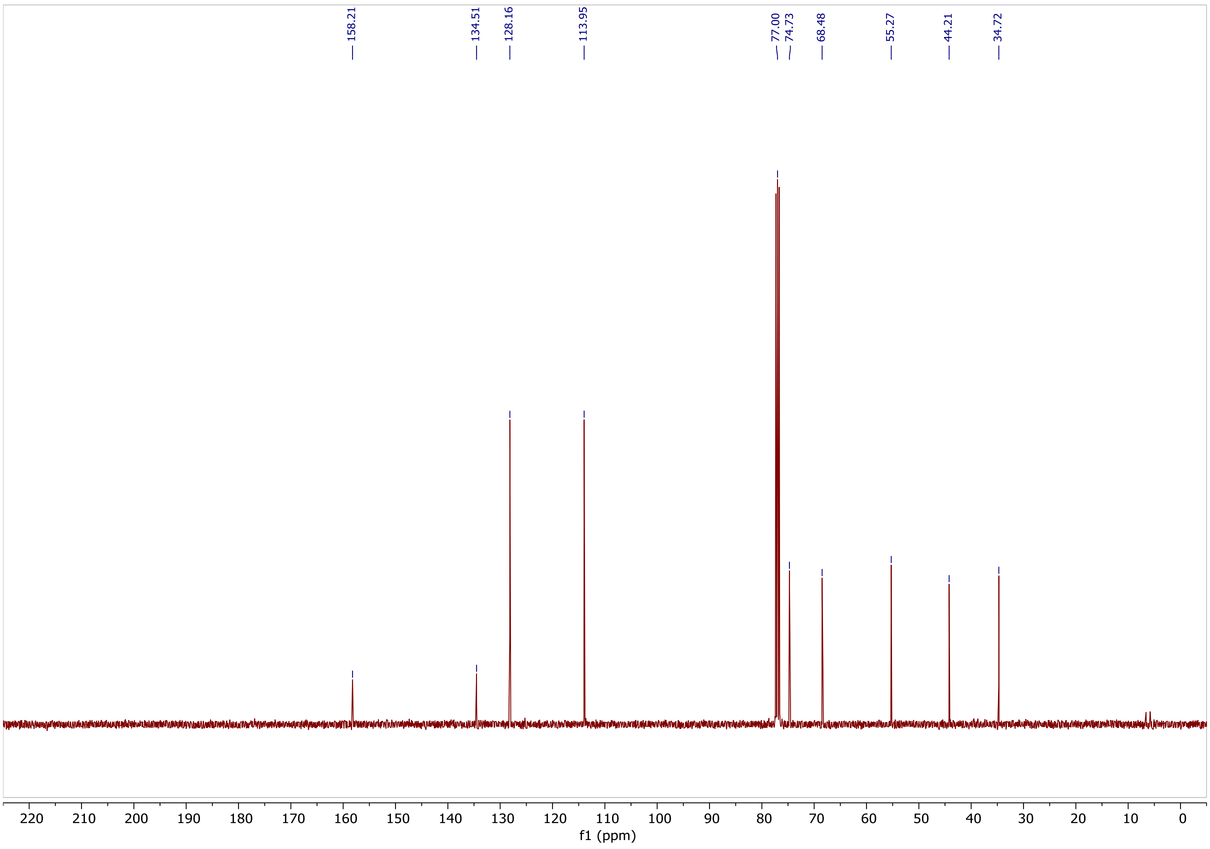
158.21
134.51
128.16
113.95
77.00
74.73
68.48
55.27
44.21
34.72
f1 (ppm)

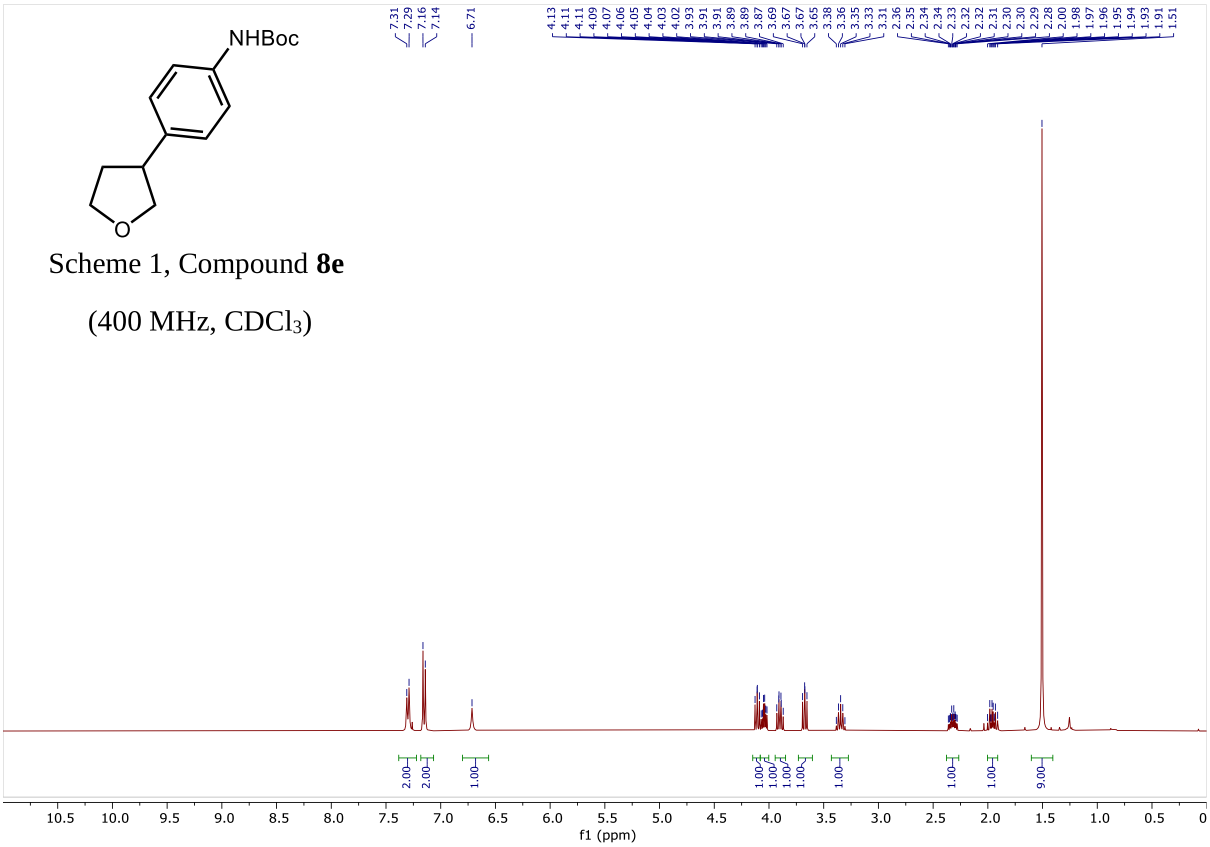

Scheme 1, Compound **8e**

(400 MHz, $CDCl_3$)

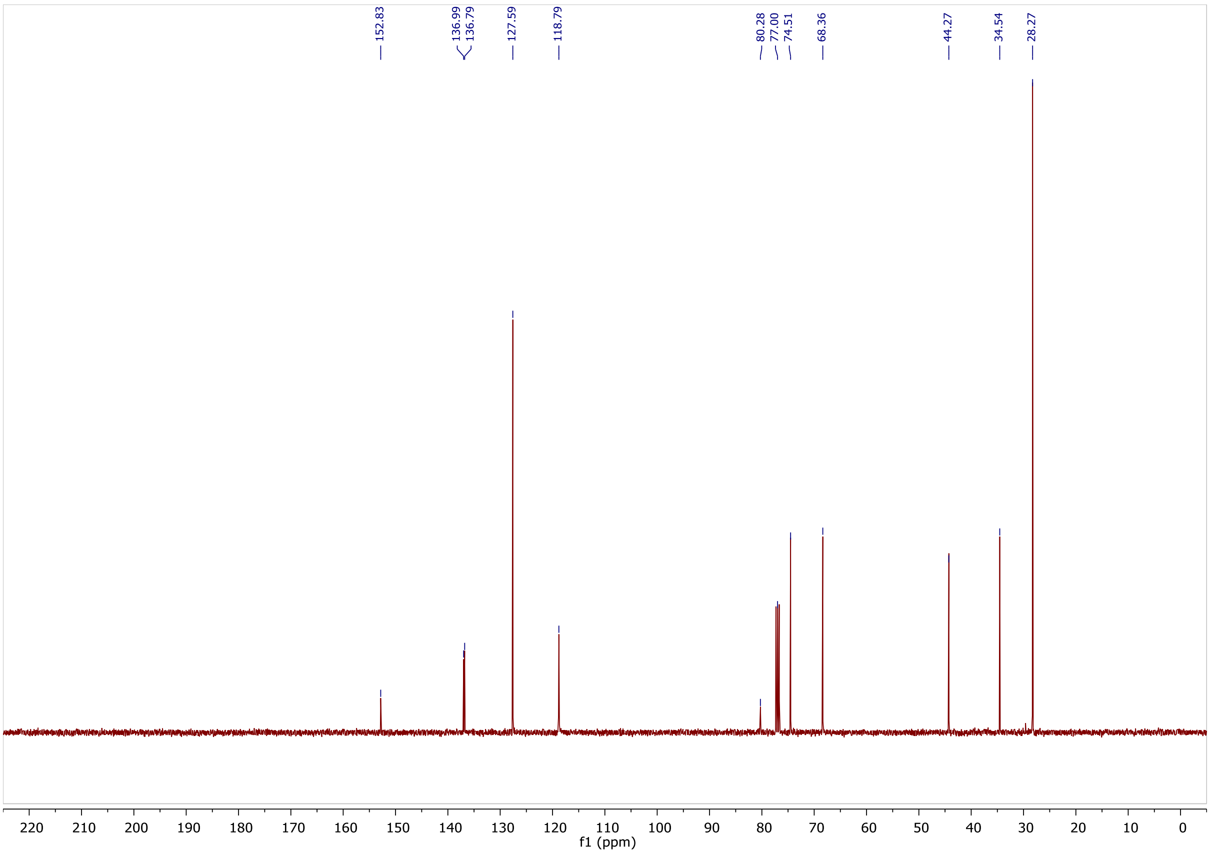

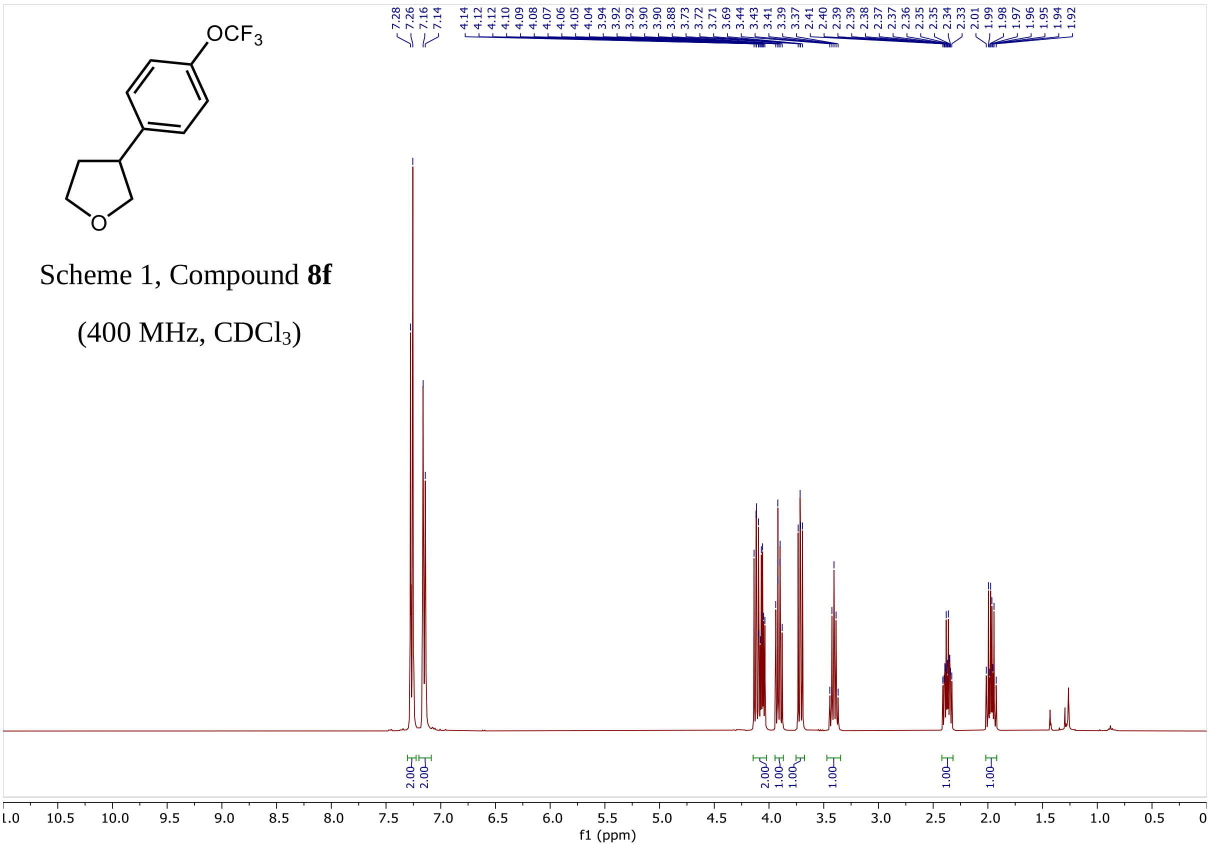
7.28
7.26
7.16
7.14
4.14 4.12 4.10 4.09 4.08 4.07 4.06 4.05 4.04 3.94 3.92 3.92 3.90 3.90 3.88 3.73 3.72 3.71 3.69 3.44 3.43 3.41 3.39 3.37 2.41 2.40 2.39 2.39 2.38 2.37 2.37 2.36 2.35 2.35 2.34 2.33 2.01 1.99 1.98 1.97 1.96 1.95 1.94 1.92
OCF3
O
Scheme 1, Compound **8f**
(400 MHz, CDCl3)
2.00 2.00 2.00 1.00 1.00 1.00 1.00 1.00
f1 (ppm)

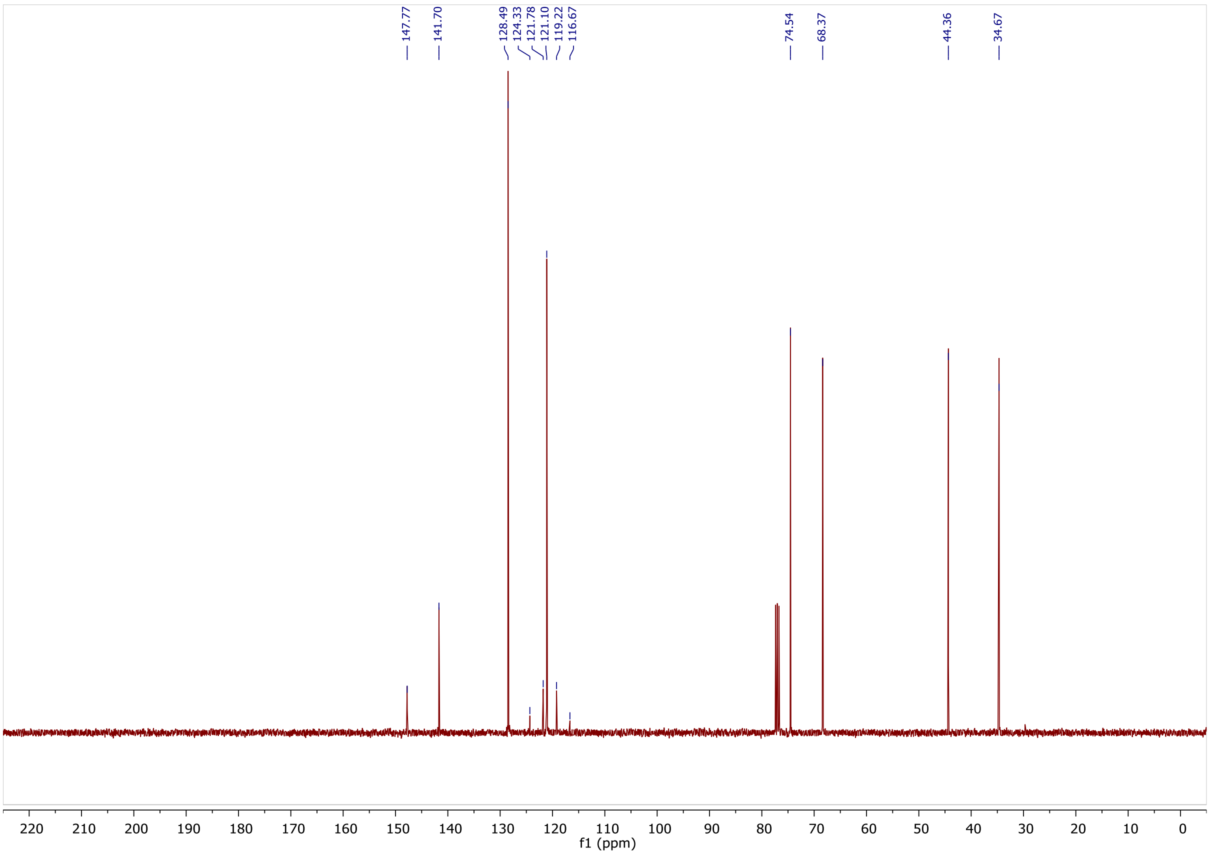
147.77
141.70
128.49 124.33 121.78 121.10 119.22 116.67
74.54
68.37
44.36
34.67
f1 (ppm)

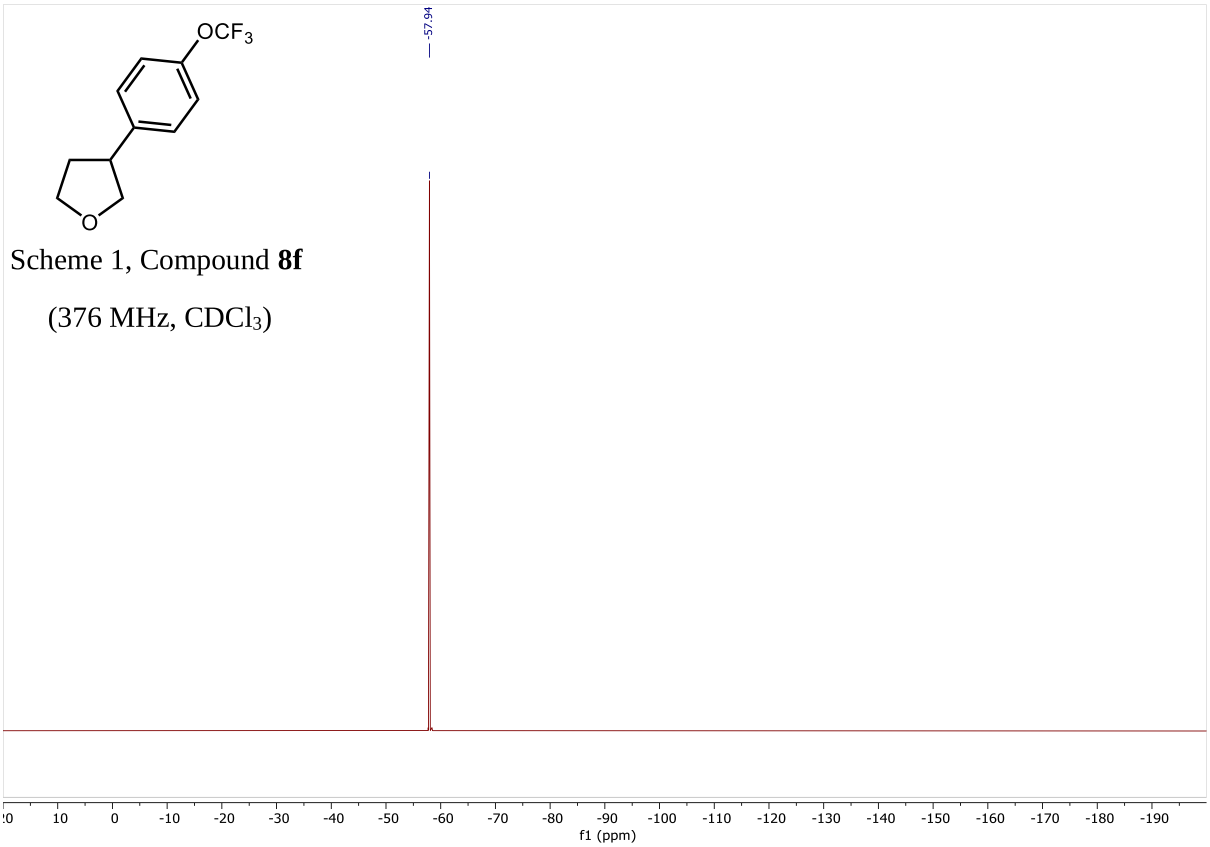

Scheme 1, Compound **8f**

(376 MHz, $CDCl_3$)

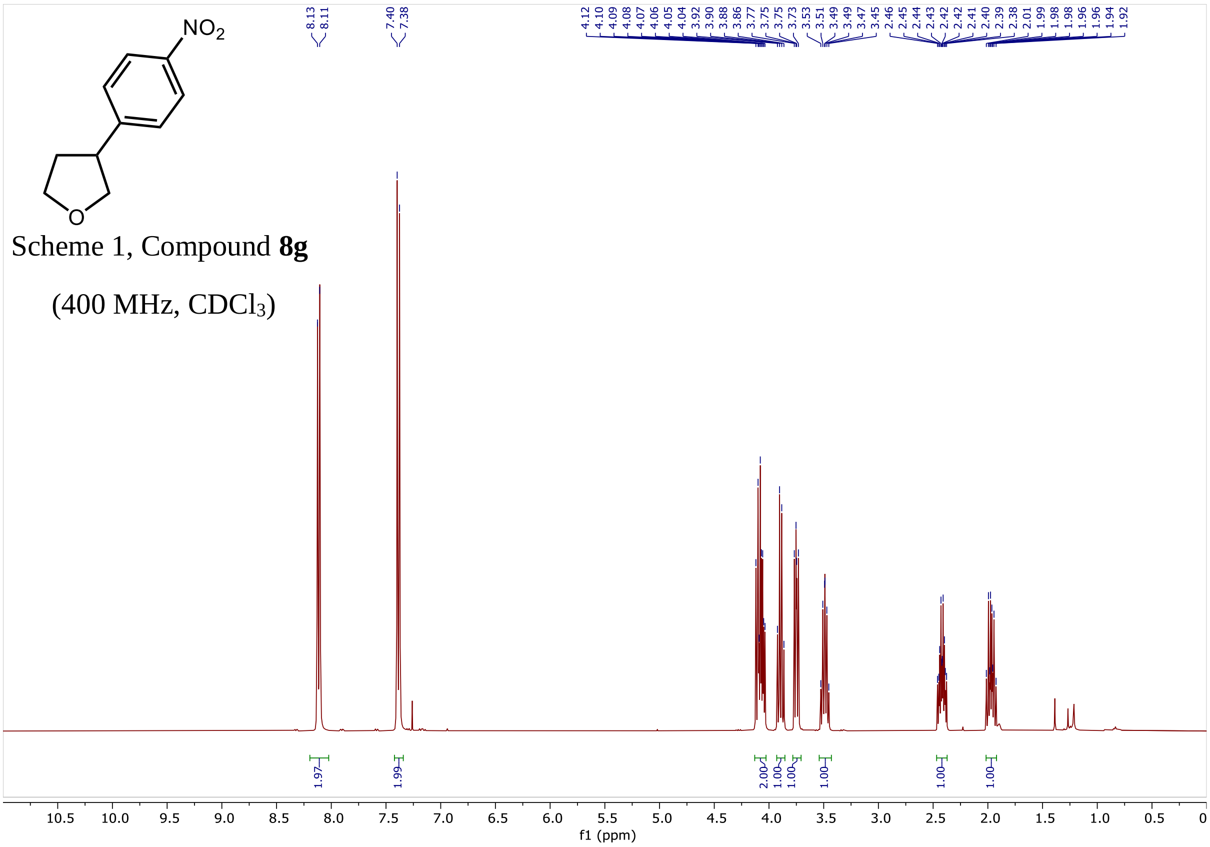
NO2
O
Scheme 1, Compound **8g**
(400 MHz, CDCl3)
f1 (ppm)

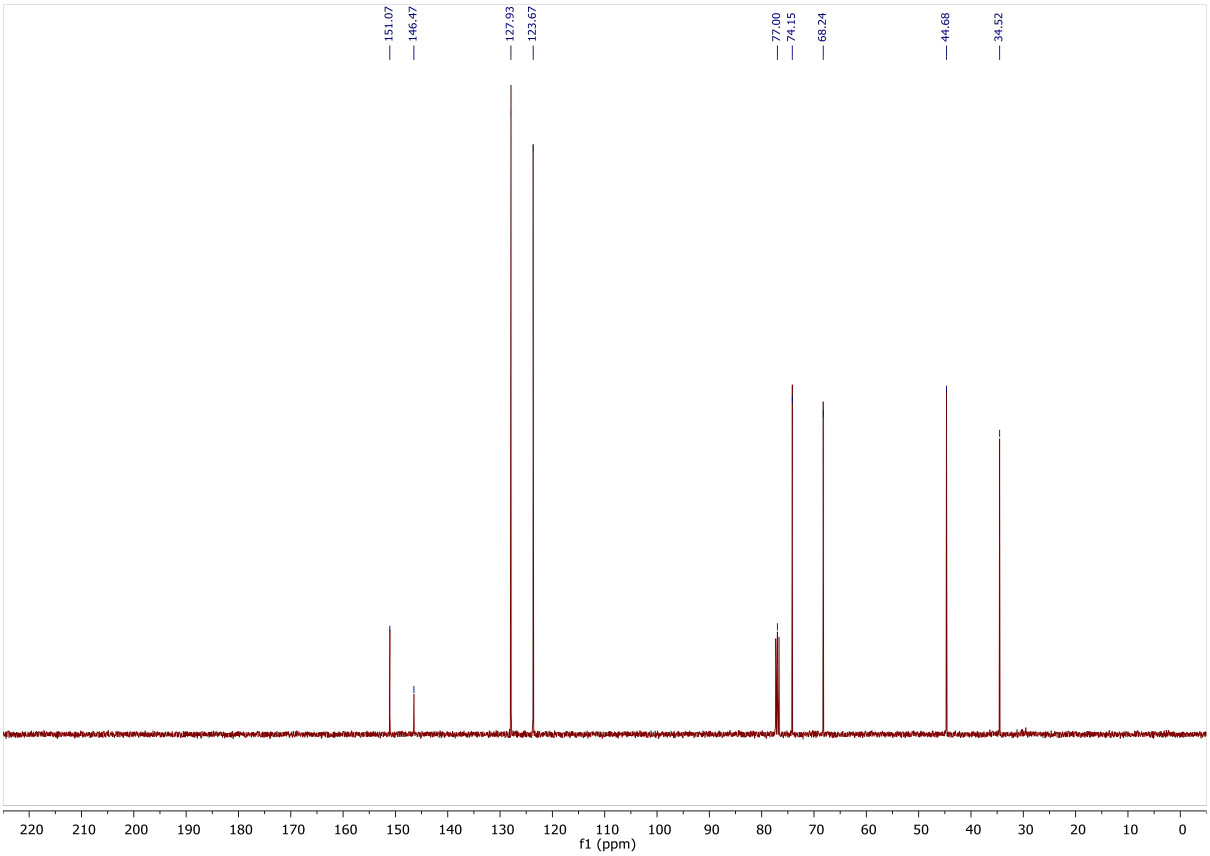
151.07
146.47
127.93
123.67
77.00
74.15
68.24
44.68
34.52
f1 (ppm)

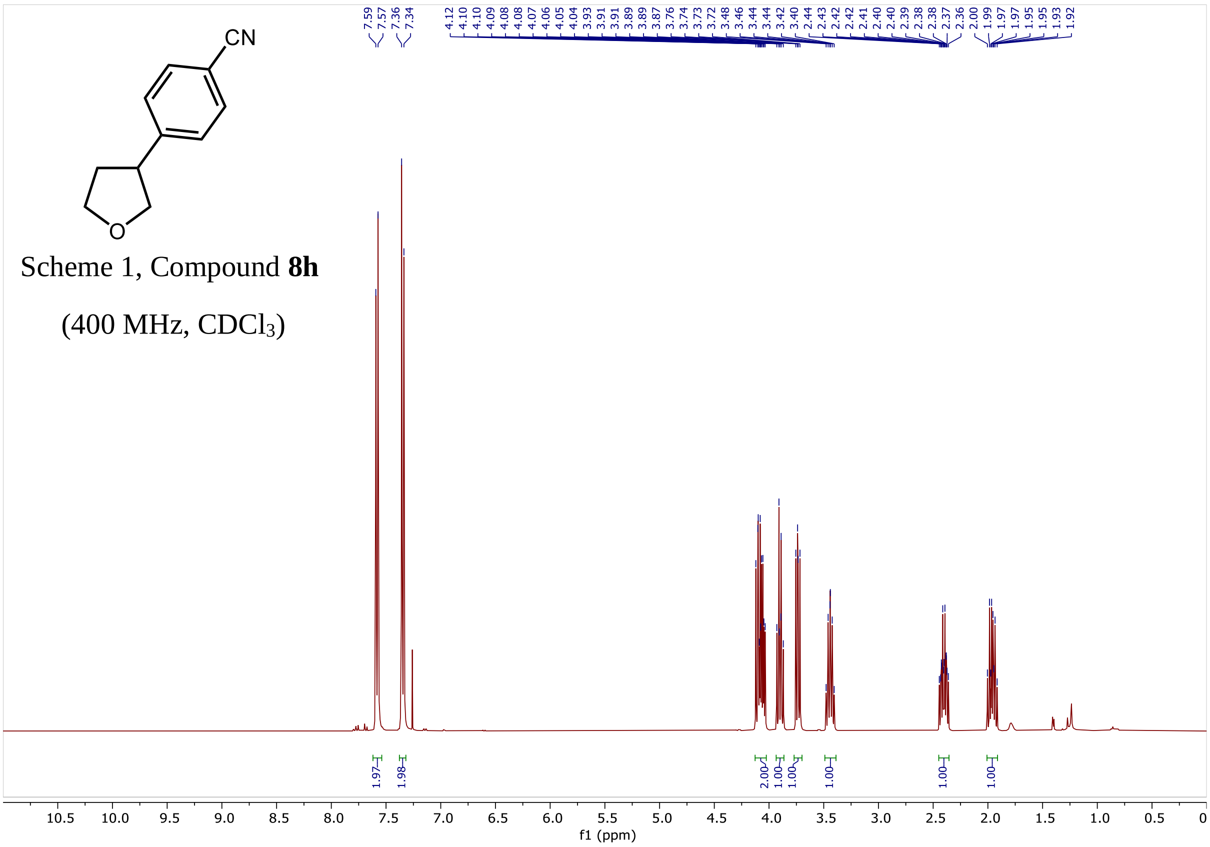
CN
O
Scheme 1, Compound 8h
(400 MHz, CDCl3)
7.59 7.57 7.36 7.34
4.12 4.10 4.10 4.09 4.08 4.08 4.07 4.06 4.05 4.04 3.93 3.91 3.91 3.89 3.89 3.87 3.76 3.74 3.73 3.72 3.48 3.46 3.44 3.44 3.42 3.40 2.44 2.43 2.42 2.42 2.41 2.40 2.40 2.39 2.38 2.38 2.37 2.37 2.36 2.00 1.99 1.97 1.97 1.95 1.95 1.93 1.92
1.97 1.98 2.00 1.00 1.00 1.00 1.00 1.00
f1 (ppm)

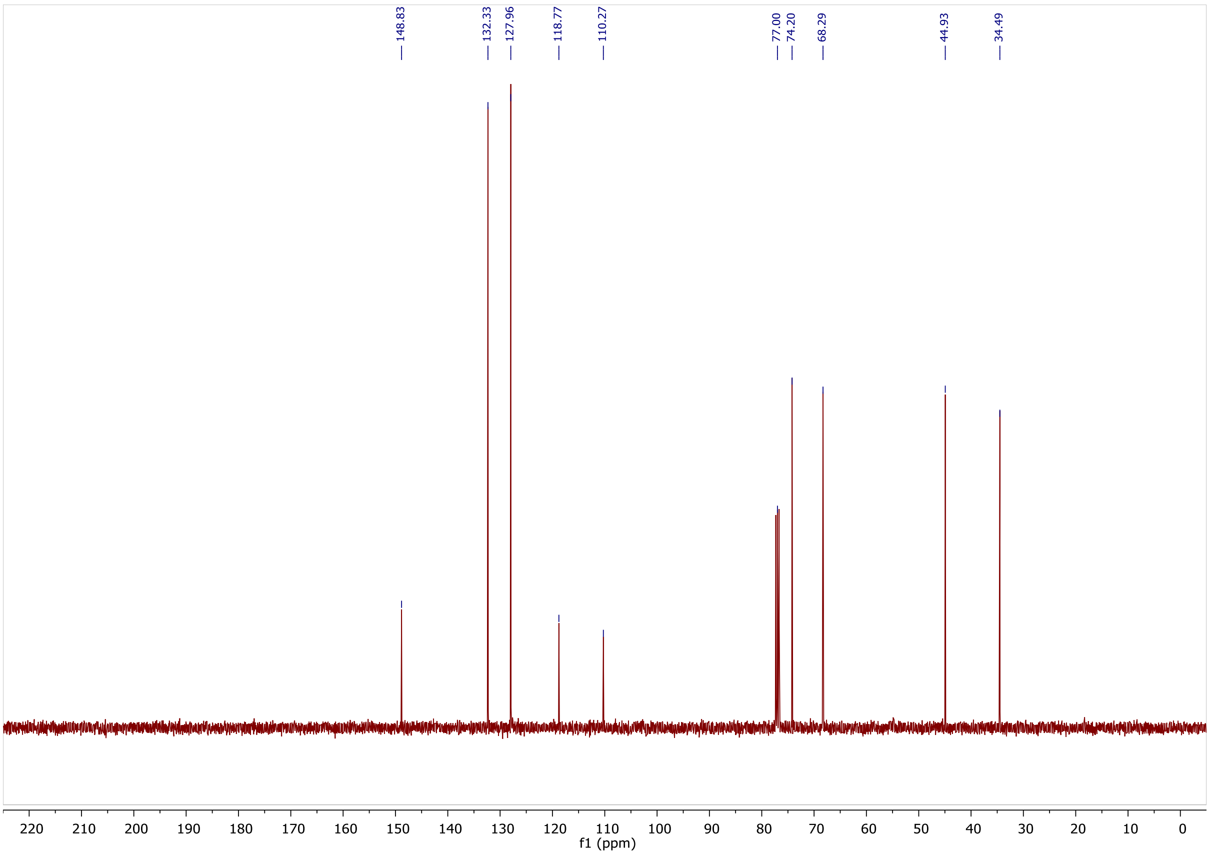
148.83
132.33
127.96
118.77
110.27
77.00
74.20
68.29
44.93
34.49
f1 (ppm)

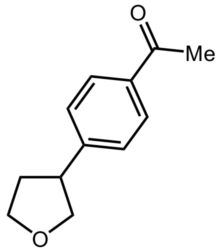

Scheme 1, Compound **8i**

(400 MHz, $CDCl_3$)

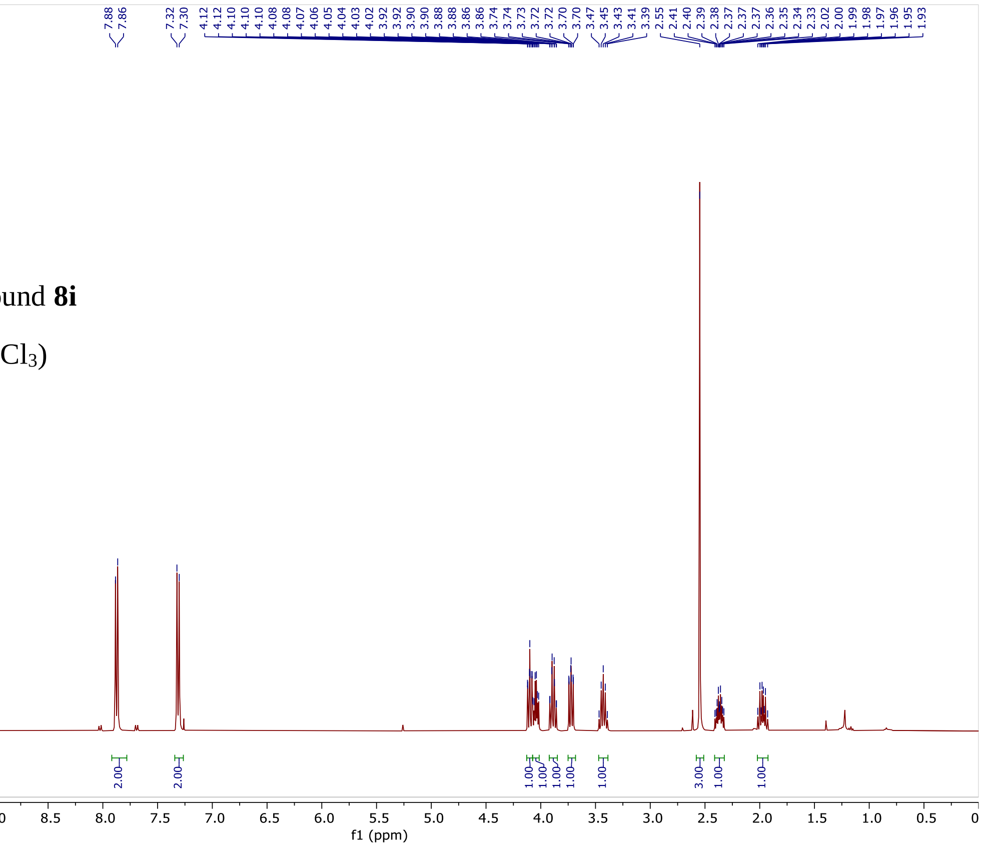

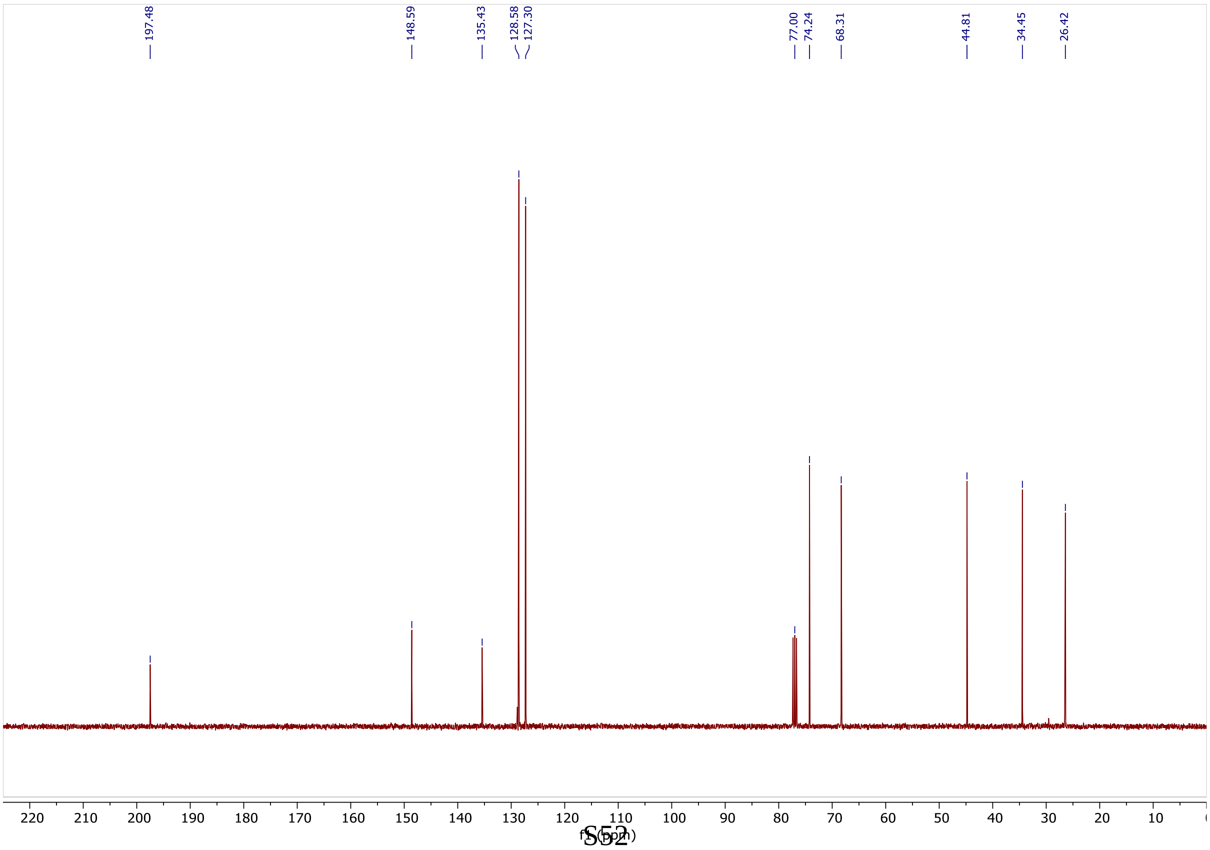

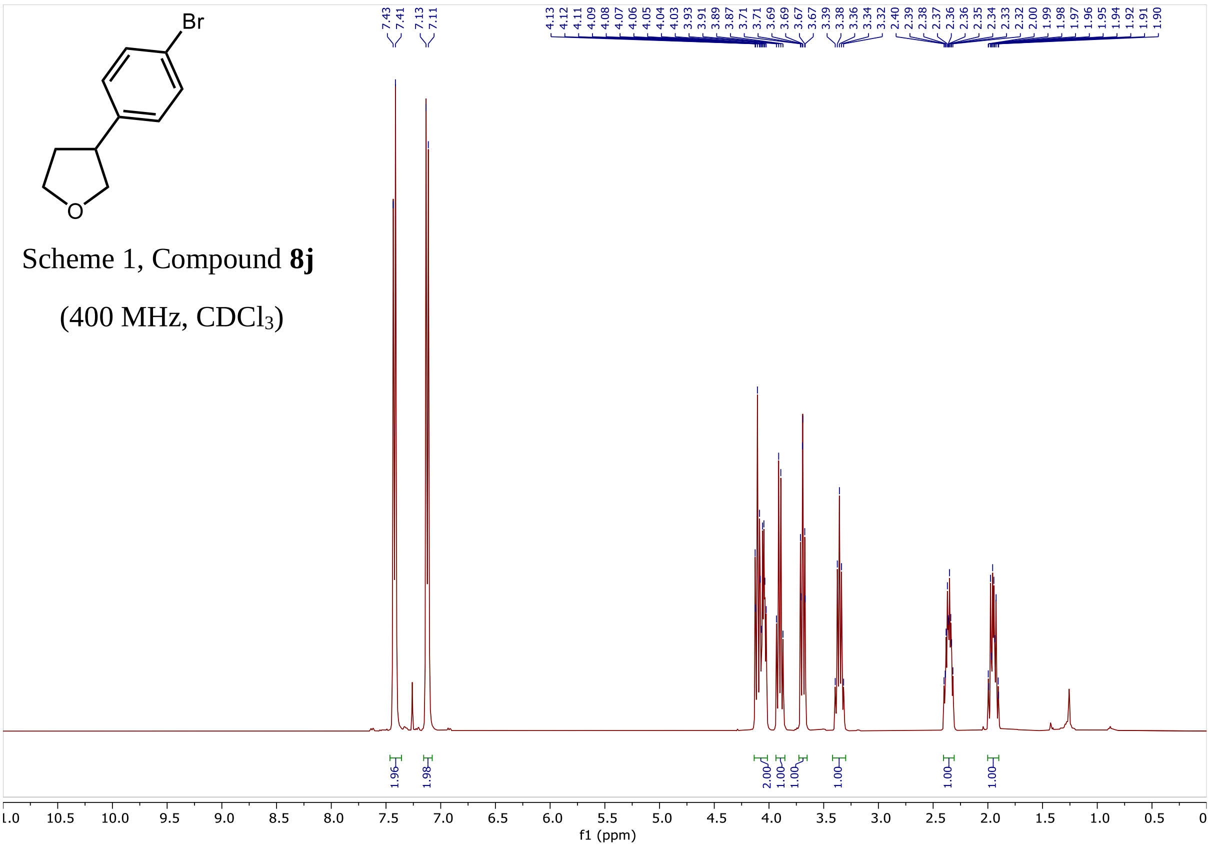

Scheme 1, Compound **8j**

(400 MHz, $CDCl_3$)

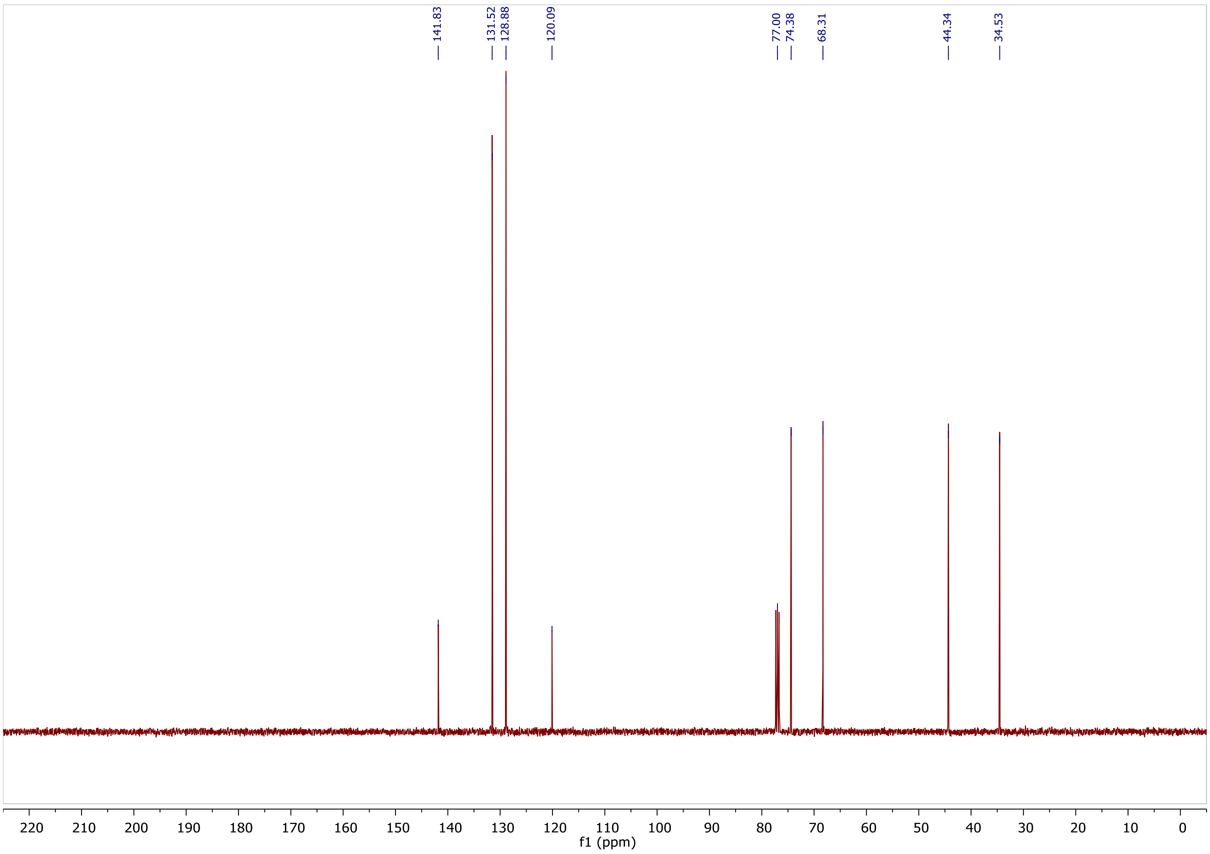

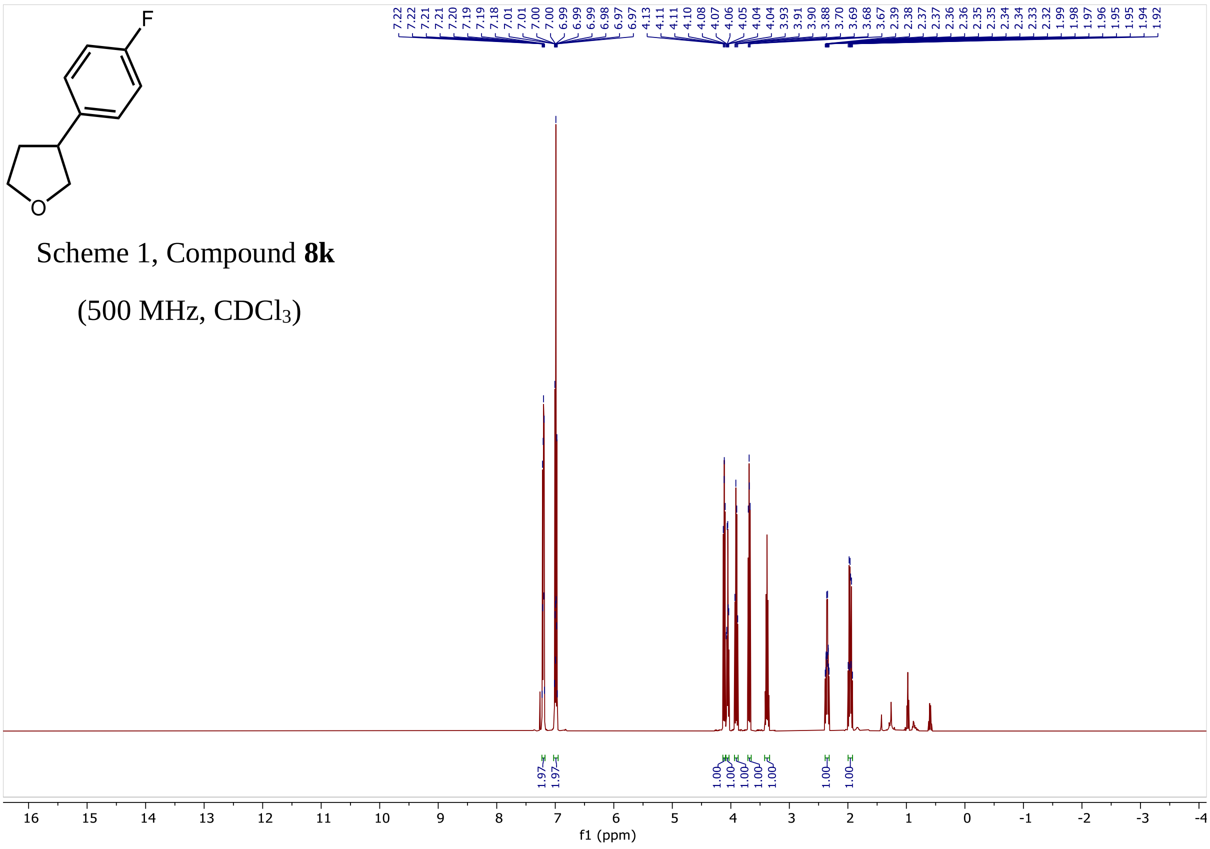
F
O
Scheme 1, Compound **8k**
(500 MHz, $CDCl_3$)
7.22 7.22 7.21 7.21 7.20 7.19 7.19 7.18 7.01 7.01 7.00 7.00 6.99 6.99 6.99 6.98 6.97 6.97 4.13 4.11 4.11 4.10 4.08 4.07 4.06 4.05 4.04 4.04 3.93 3.91 3.90 3.88 3.70 3.69 3.68 3.67 2.39 2.38 2.37 2.37 2.36 2.35 2.35 2.34 2.34 2.33 2.32 1.99 1.98 1.97 1.96 1.95 1.95 1.94 1.92
1.97 1.97 1.00 1.00 1.00 1.00 1.00 1.00 1.00
16 15 14 13 12 11 10 9 8 7 6 5 4 3 2 1 0 -1 -2 -3 -4
f1 (ppm)

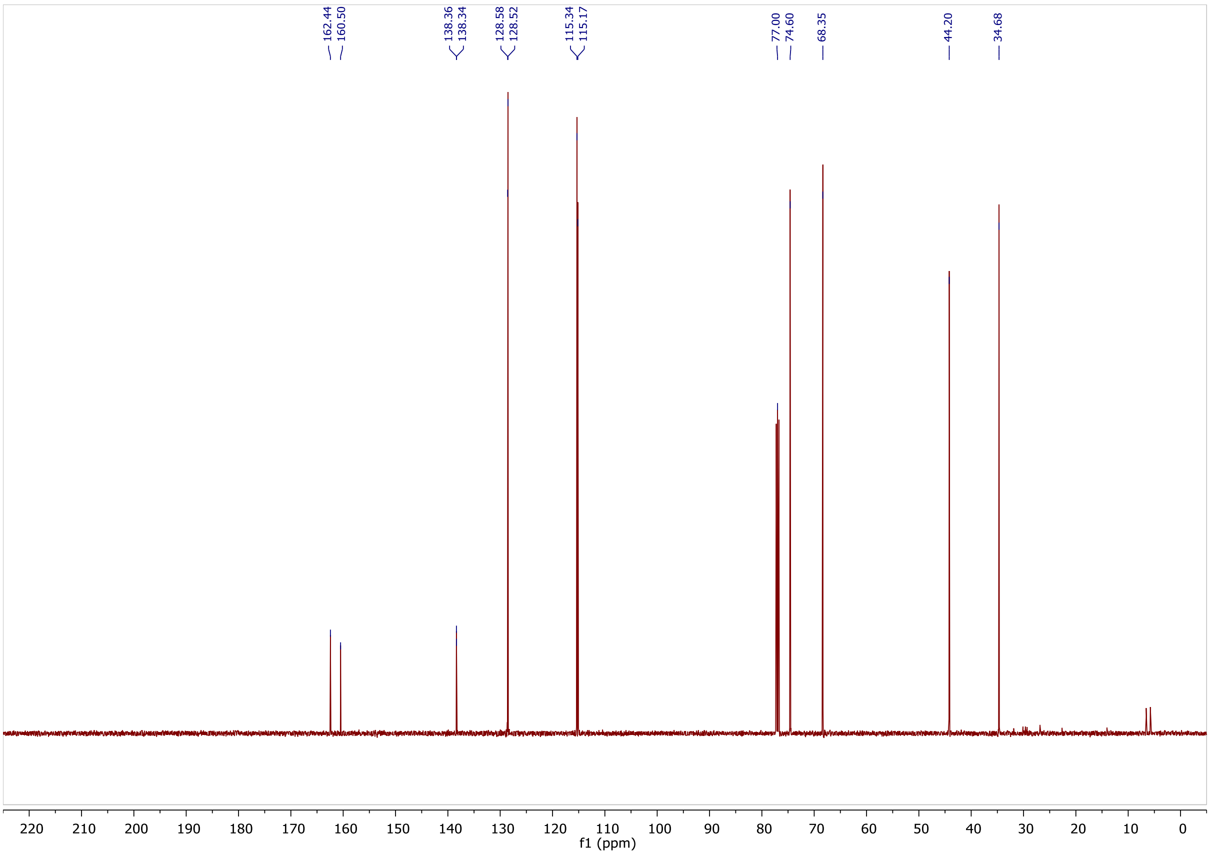
162.44 160.50 138.36 138.34 128.58 128.52 115.34 115.17 77.00 74.60 68.35 44.20 34.68
220 210 200 190 180 170 160 150 140 130 120 110 100 90 80 70 60 50 40 30 20 10 0
f1 (ppm)

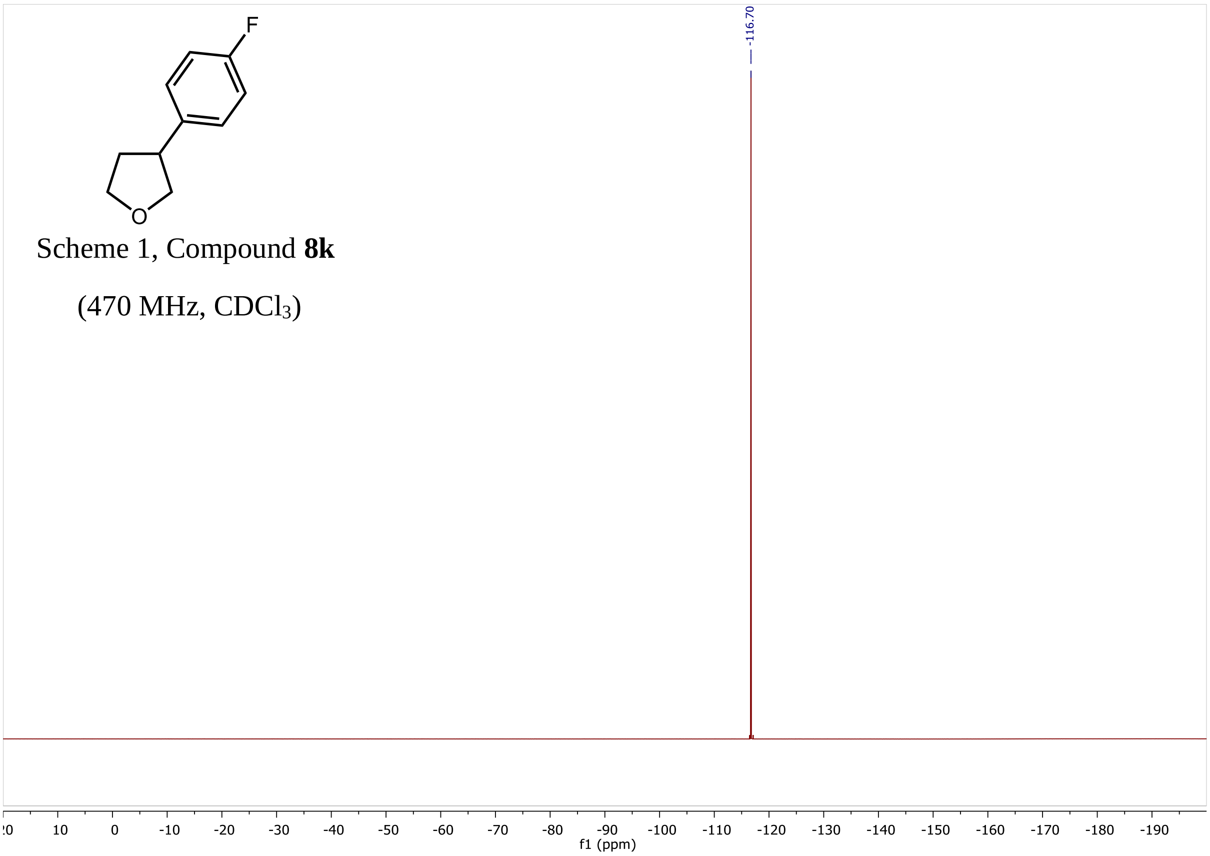
F
O
Scheme 1, Compound **8k**
(470 MHz, $CDCl_3$)
-116.70
10
0
-10
-20
-30
-40
-50
-60
-70
-80
-90
-100
-110
-120
-130
-140
-150
-160
-170
-180
-190
f1 (ppm)

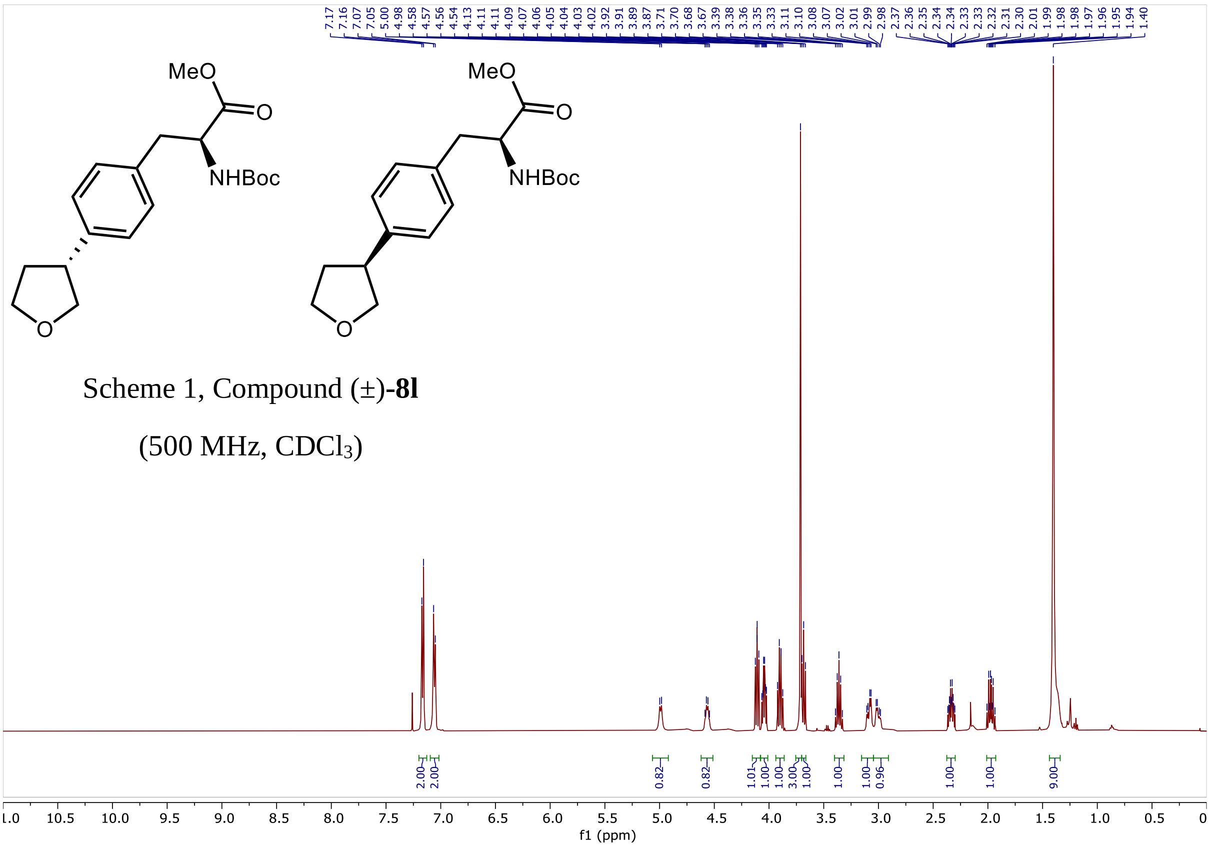

Scheme 1, Compound (±)-**8l**

(500 MHz, $CDCl_3$)

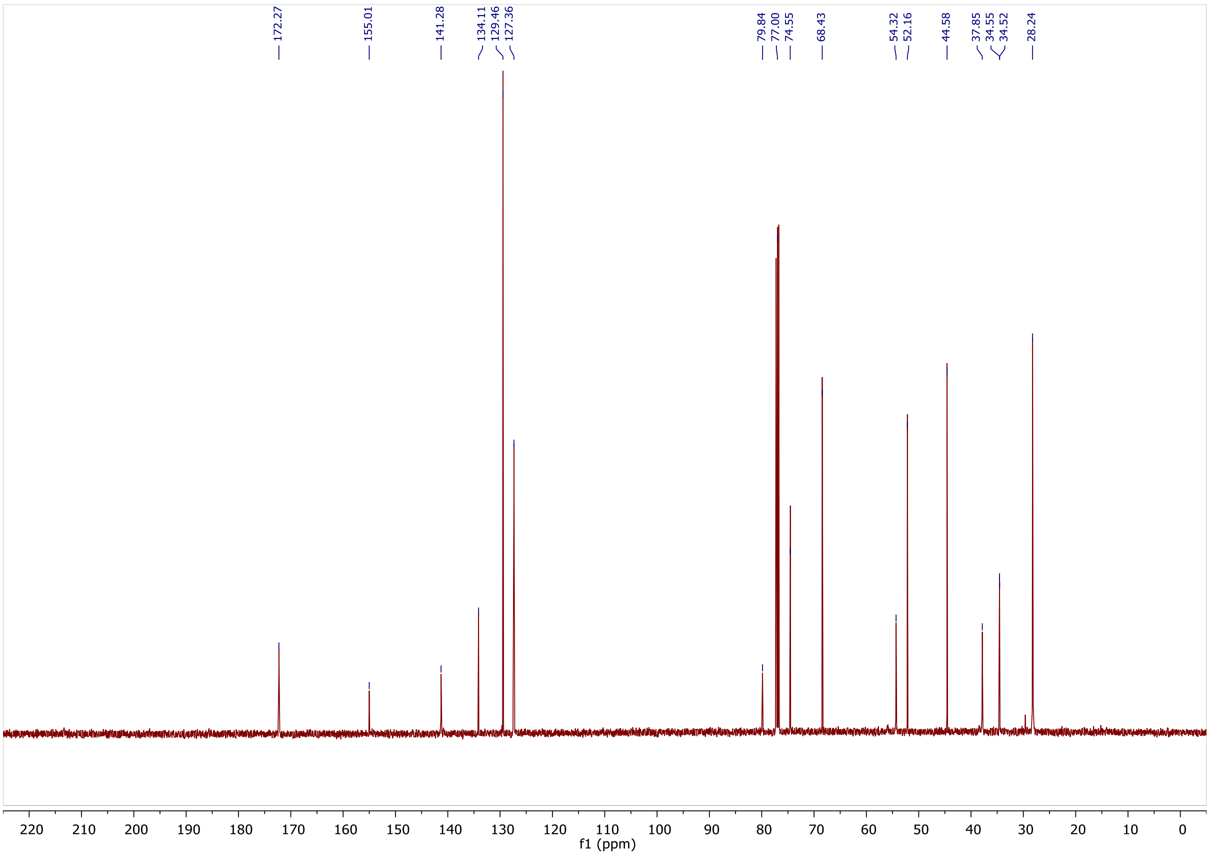

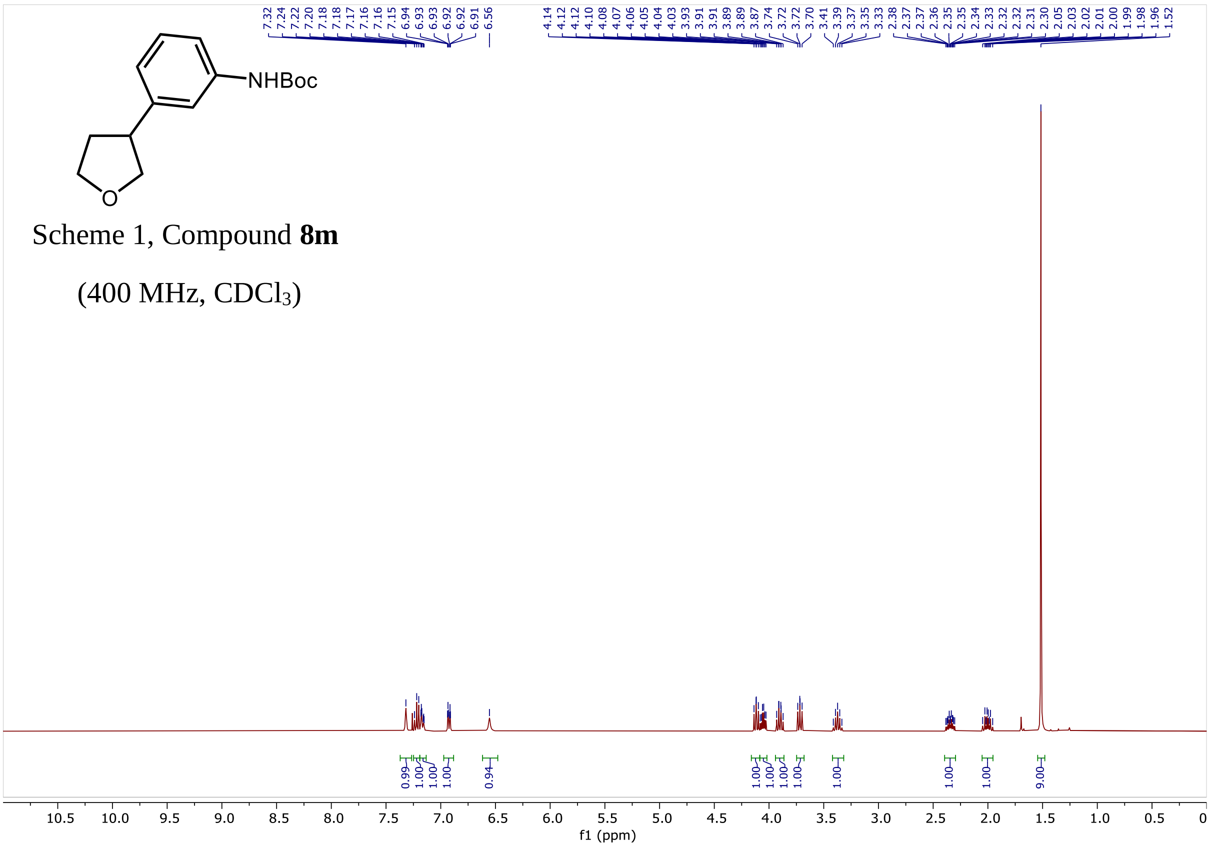
7.32 7.24 7.22 7.20 7.18 7.18 7.17 7.16 7.15 6.94 6.93 6.93 6.92 6.92 6.91 6.56
4.14 4.12 4.12 4.10 4.08 4.07 4.06 4.05 4.04 4.03 3.93 3.91 3.91 3.89 3.89 3.87 3.74 3.72 3.72 3.70 3.41 3.39 3.37 3.35 3.33 2.38 2.37 2.37 2.36 2.35 2.35 2.34 2.33 2.32 2.32 2.31 2.30 2.05 2.03 2.02 2.01 2.00 1.99 1.98 1.96 1.52
NHBoc
O
Scheme 1, Compound **8m**
(400 MHz, $CDCl_3$)
0.99 1.00 1.00 1.00 0.94 1.00 1.00 1.00 1.00 1.00 1.00 1.00 9.00
10.5 10.0 9.5 9.0 8.5 8.0 7.5 7.0 6.5 6.0 5.5 5.0 4.5 4.0 3.5 3.0 2.5 2.0 1.5 1.0 0.5 0
f1 (ppm)

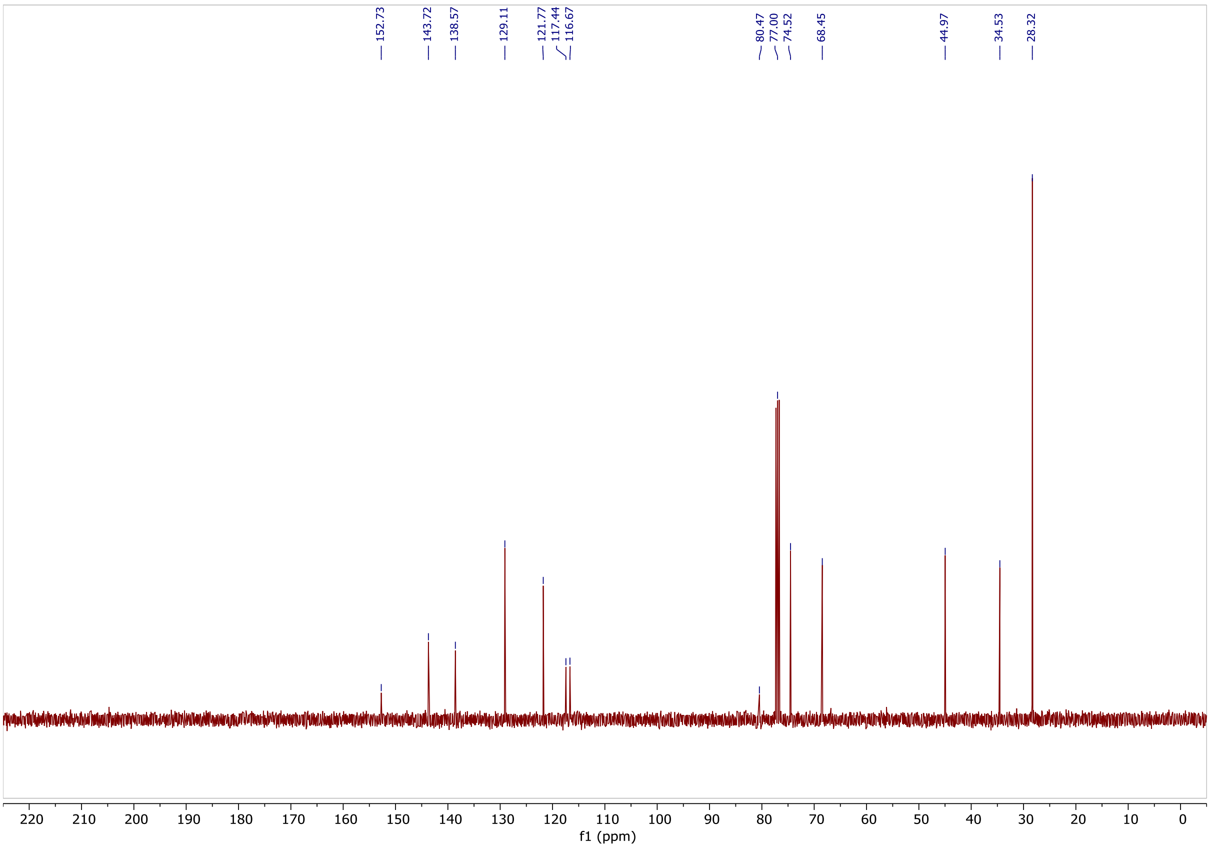
152.73 143.72 138.57 129.11 121.77 117.44 116.67 80.47 77.00 74.52 68.45 44.97 34.53 28.32
220 210 200 190 180 170 160 150 140 130 120 110 100 90 80 70 60 50 40 30 20 10 0
f1 (ppm)

Scheme 1, Compound **8n**

(400 MHz, $CDCl_3$)

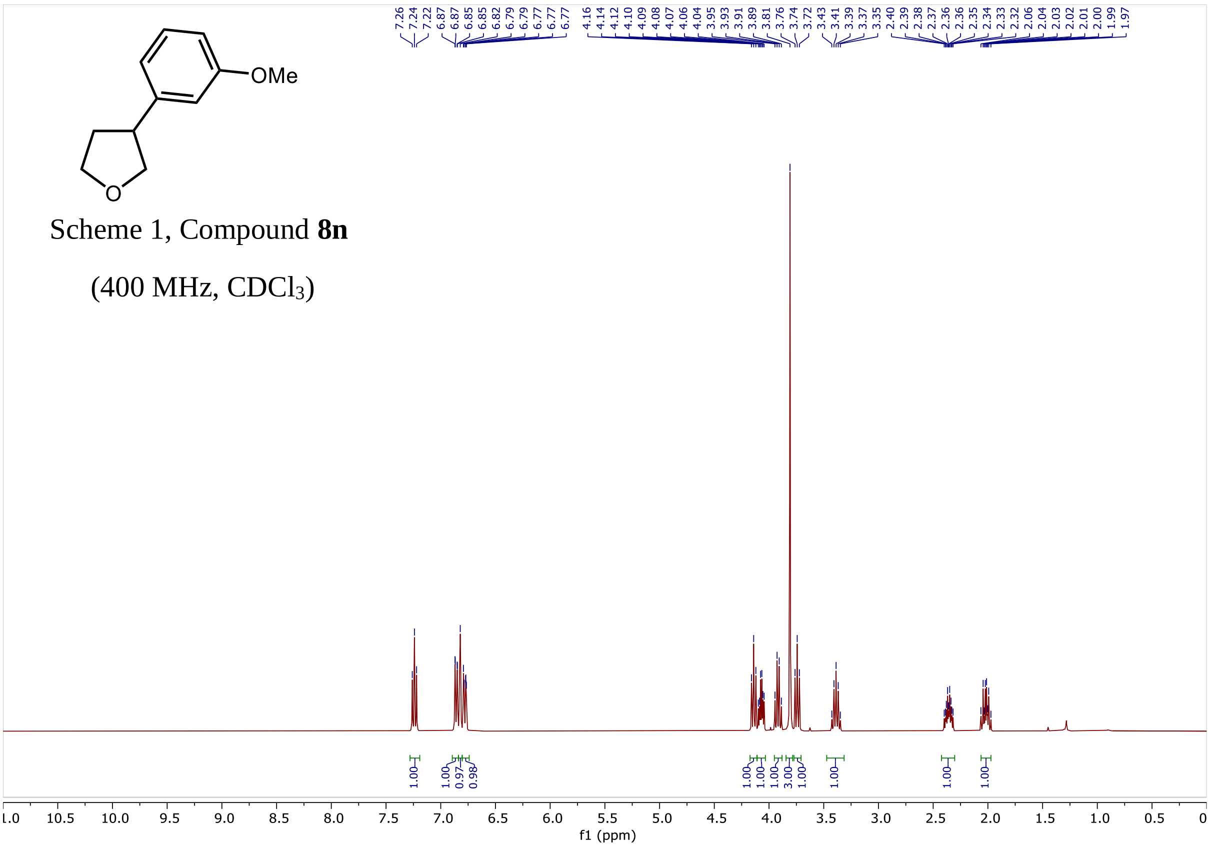

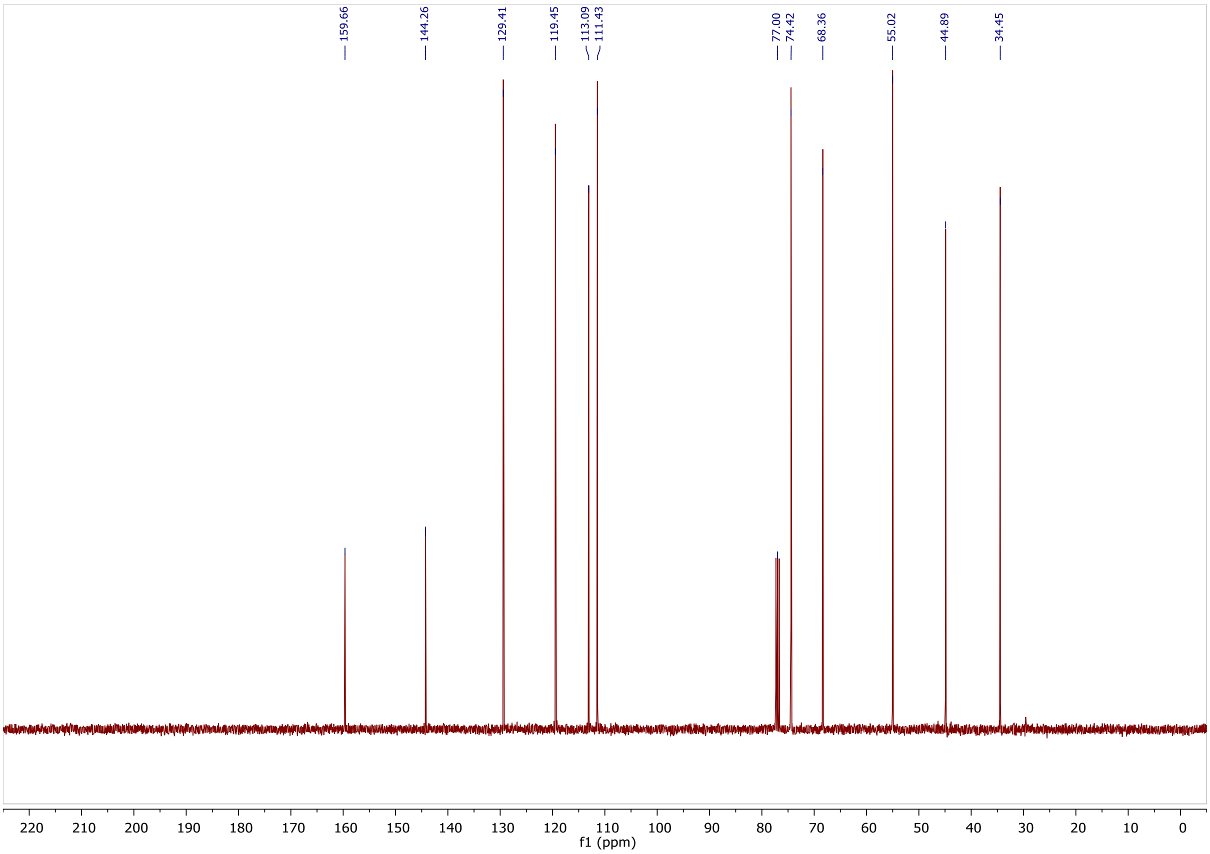

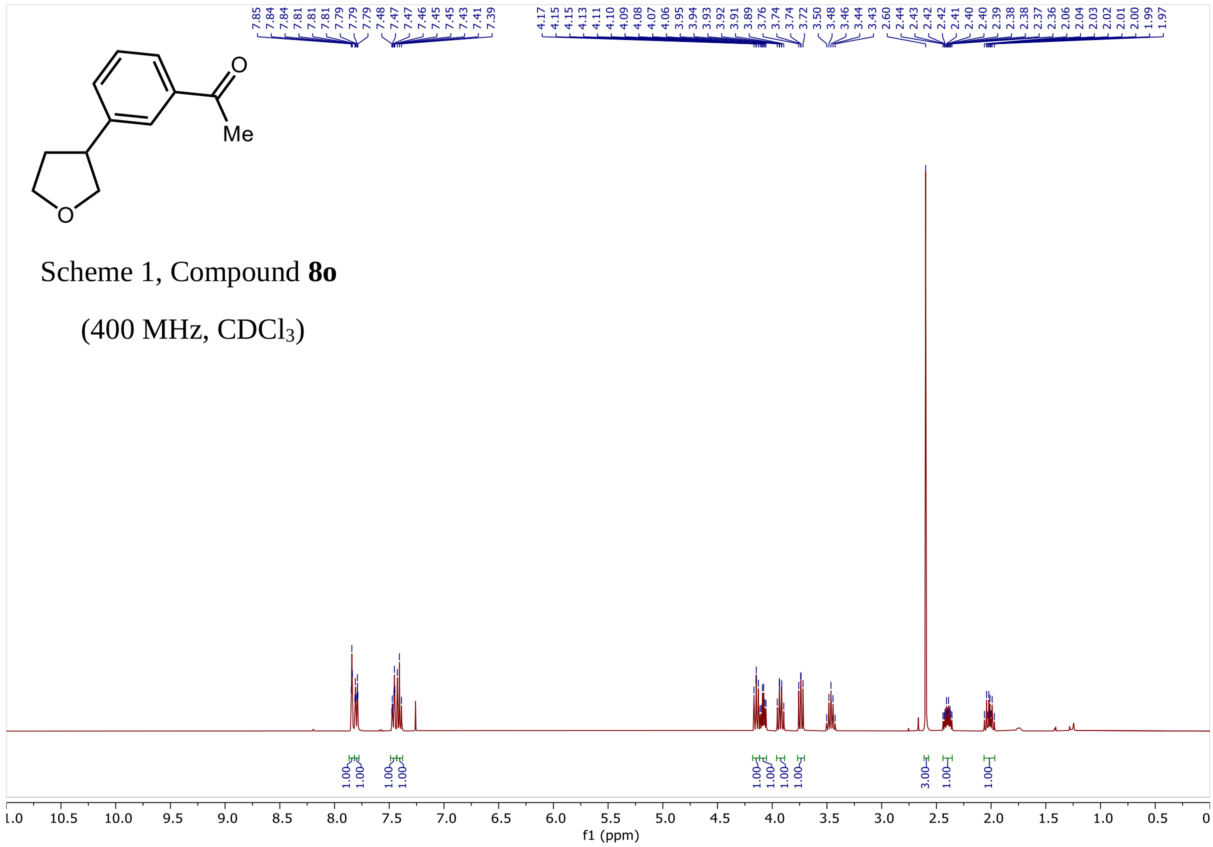
Scheme 1, Compound **8o**
(400 MHz, $CDCl_3$)
Me
O
f1 (ppm)

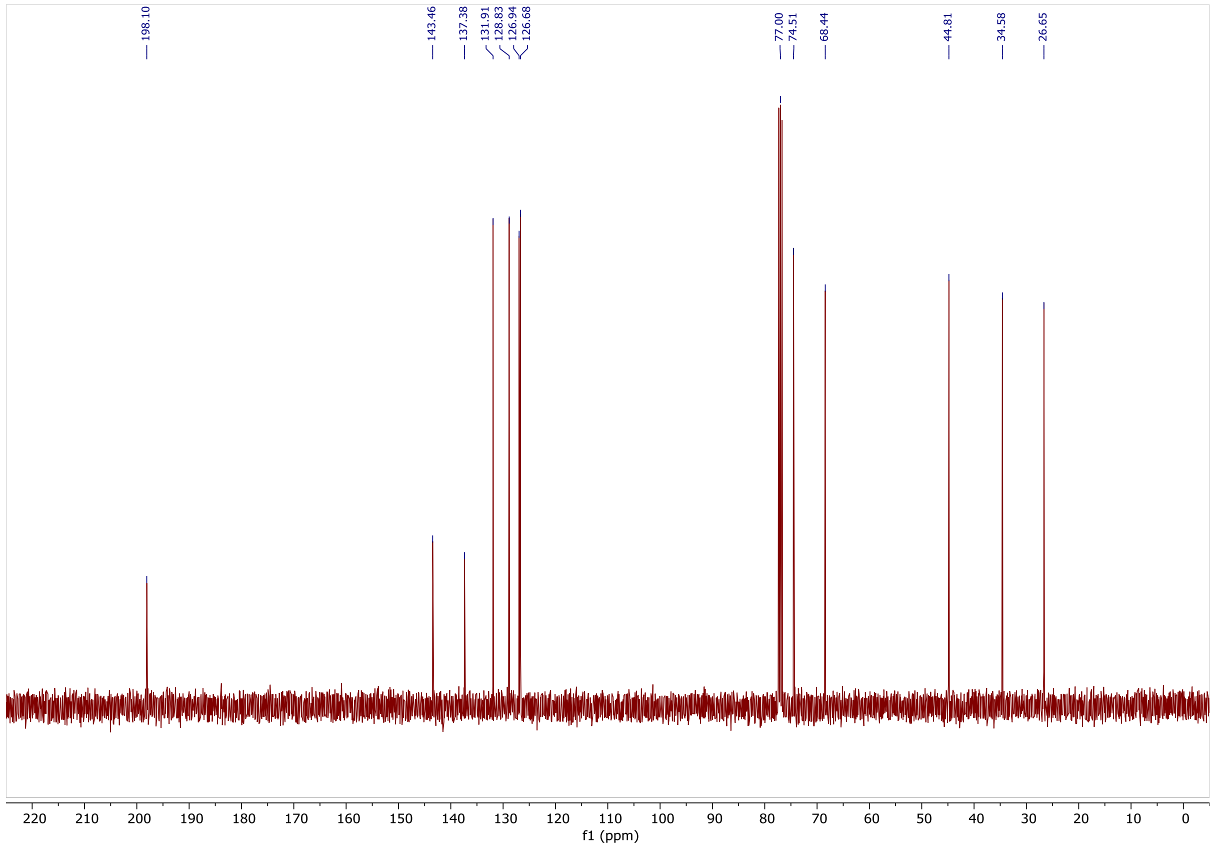
198.10
143.46
137.38
131.91
128.83
126.94
126.68
77.00
74.51
68.44
44.81
34.58
26.65
f1 (ppm)

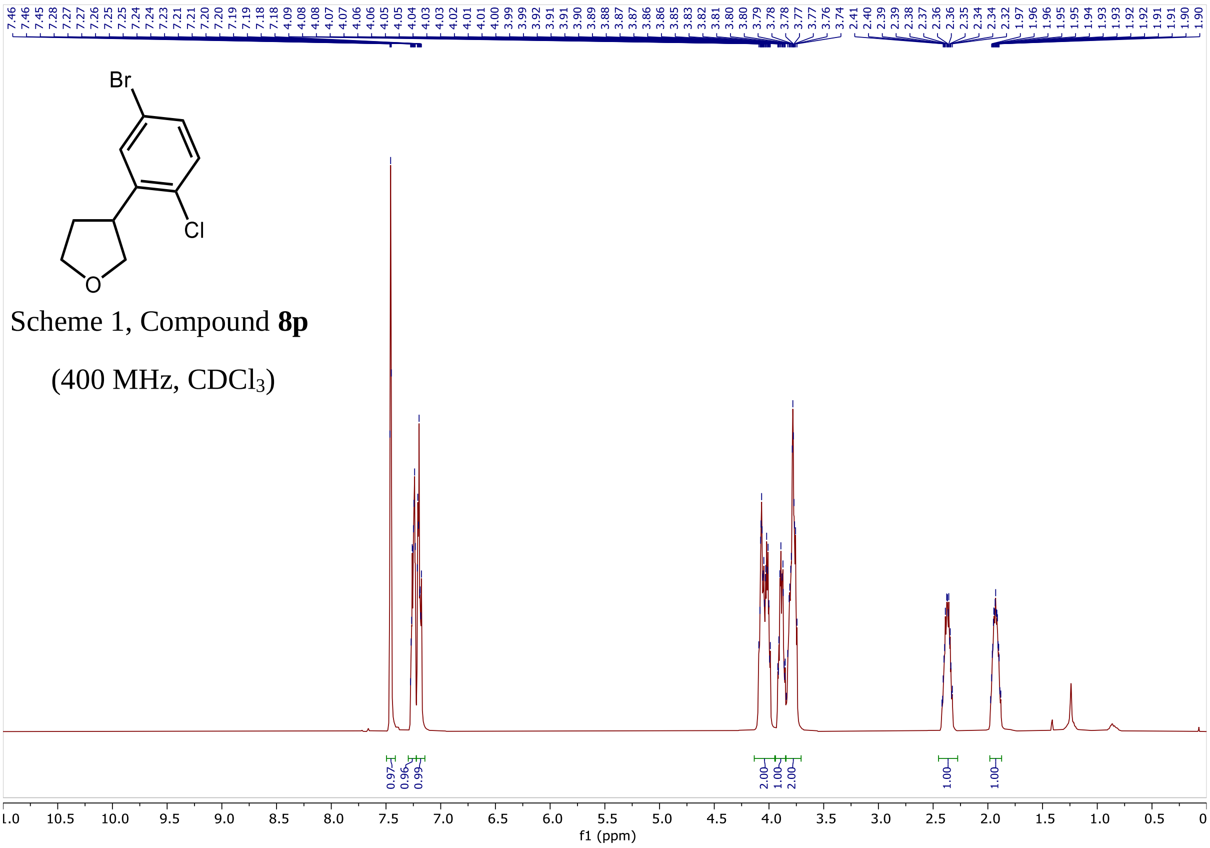
7.46 7.46 7.45 7.28 7.27 7.27 7.26 7.25 7.25 7.24 7.24 7.23 7.21 7.21 7.20 7.20 7.19 7.19 7.18 4.09 4.08 4.08 4.07 4.07 4.06 4.06 4.05 4.05 4.04 4.03 4.03 4.02 4.01 4.01 4.00 3.99 3.99 3.92 3.91 3.91 3.90 3.89 3.88 3.87 3.87 3.86 3.85 3.85 3.83 3.82 3.81 3.80 3.79 3.78 3.78 3.77 3.77 3.76 3.74 2.41 2.40 2.39 2.39 2.38 2.37 2.36 2.36 2.35 2.34 2.34 2.32 1.97 1.96 1.96 1.95 1.95 1.94 1.93 1.93 1.92 1.92 1.91 1.91 1.90 1.90
Br
Cl
O
Scheme 1, Compound **8p**
(400 MHz, $CDCl_3$)
0.97 0.96 0.99
2.00 1.00 2.00
1.00
1.00
f1 (ppm)

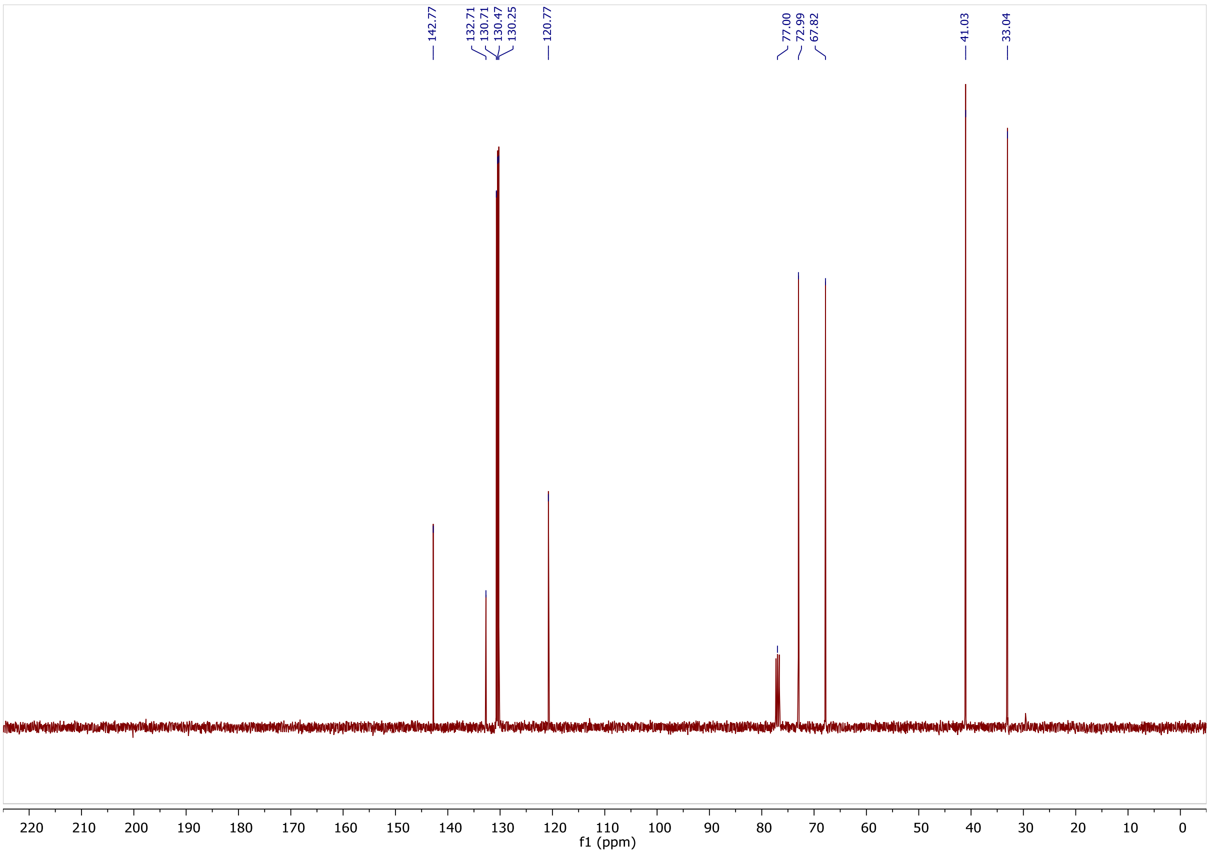
142.77
132.71 130.71 130.47 130.25
120.77
77.00 72.99 67.82
41.03
33.04
f1 (ppm)

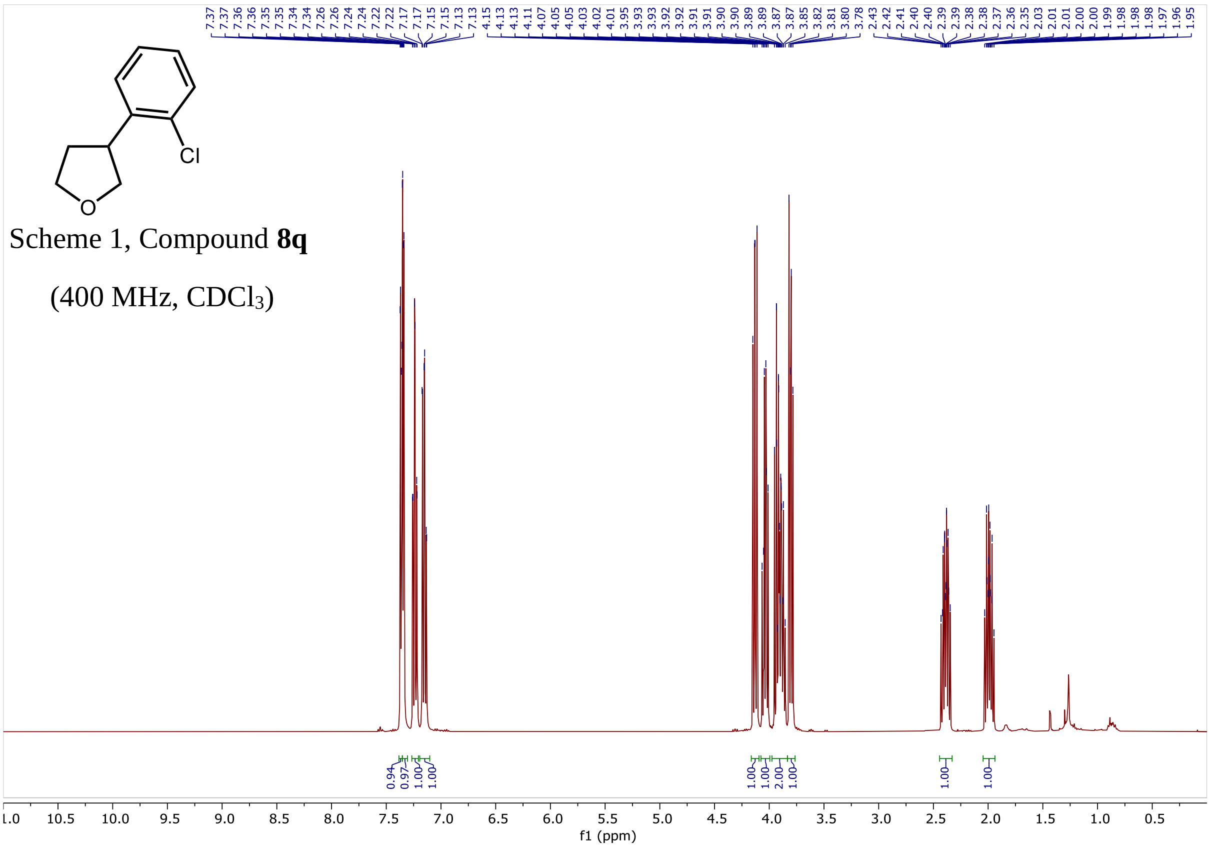
7.37 7.37 7.36 7.36 7.35 7.35 7.34 7.34 7.26 7.26 7.24 7.24 7.22 7.22 7.17 7.17 7.15 7.15 7.13 7.13
4.15 4.13 4.13 4.11 4.07 4.05 4.05 4.03 4.02 4.01 3.95 3.93 3.93 3.92 3.92 3.91 3.91 3.90 3.90 3.89 3.89 3.87 3.87 3.85 3.82 3.81 3.80 3.78
2.43 2.42 2.41 2.40 2.40 2.39 2.39 2.38 2.38 2.37 2.36 2.35 2.03 2.01 2.01 2.00 2.00 1.99 1.98 1.98 1.98 1.97 1.96 1.95
Cl
O
Scheme 1, Compound **8q**
(400 MHz, $CDCl_3$)
0.94 0.97 1.00 1.00
1.00 1.00 2.00 1.00
1.00
1.00
1.0 10.5 10.0 9.5 9.0 8.5 8.0 7.5 7.0 6.5 6.0 5.5 5.0 4.5 4.0 3.5 3.0 2.5 2.0 1.5 1.0 0.5
f1 (ppm)

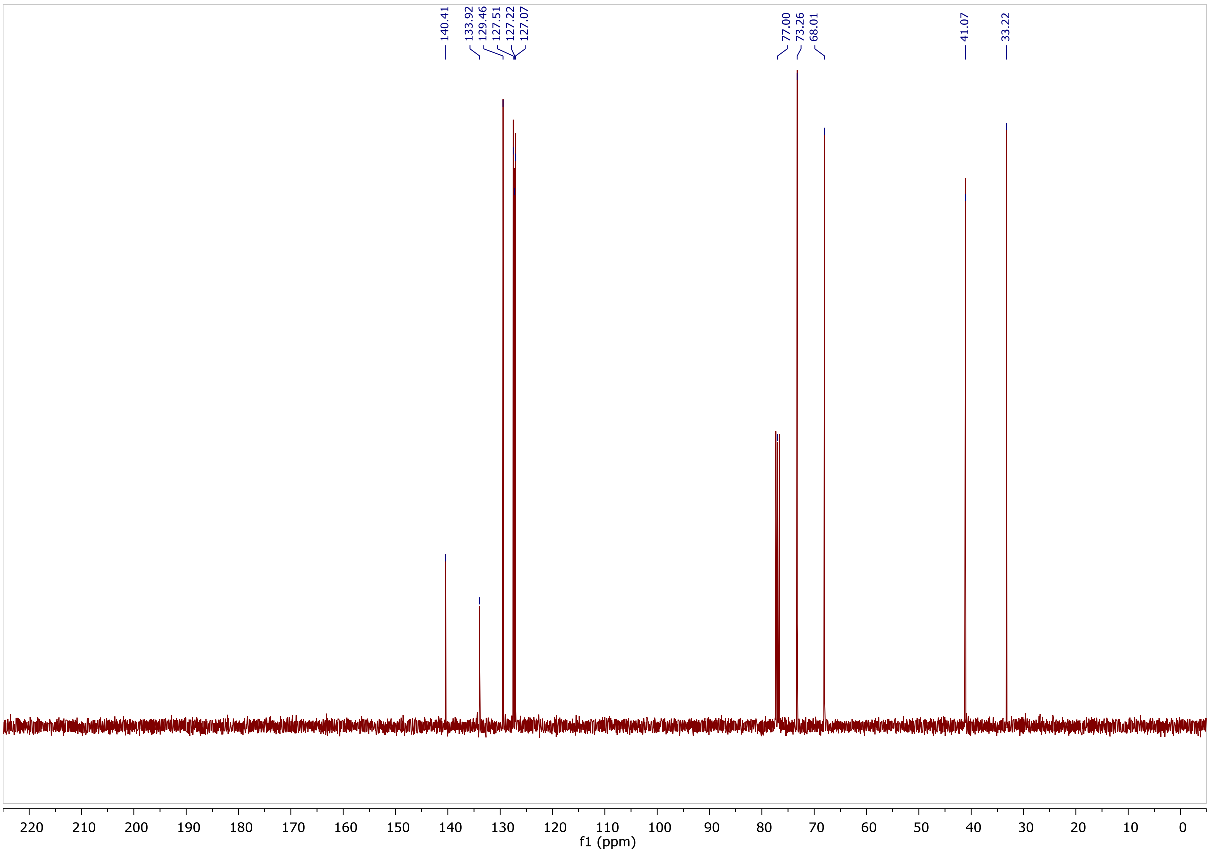
140.41
133.92 129.46 127.51 127.22 127.07
77.00 73.26 68.01
41.07
33.22
220 210 200 190 180 170 160 150 140 130 120 110 100 90 80 70 60 50 40 30 20 10 0
f1 (ppm)

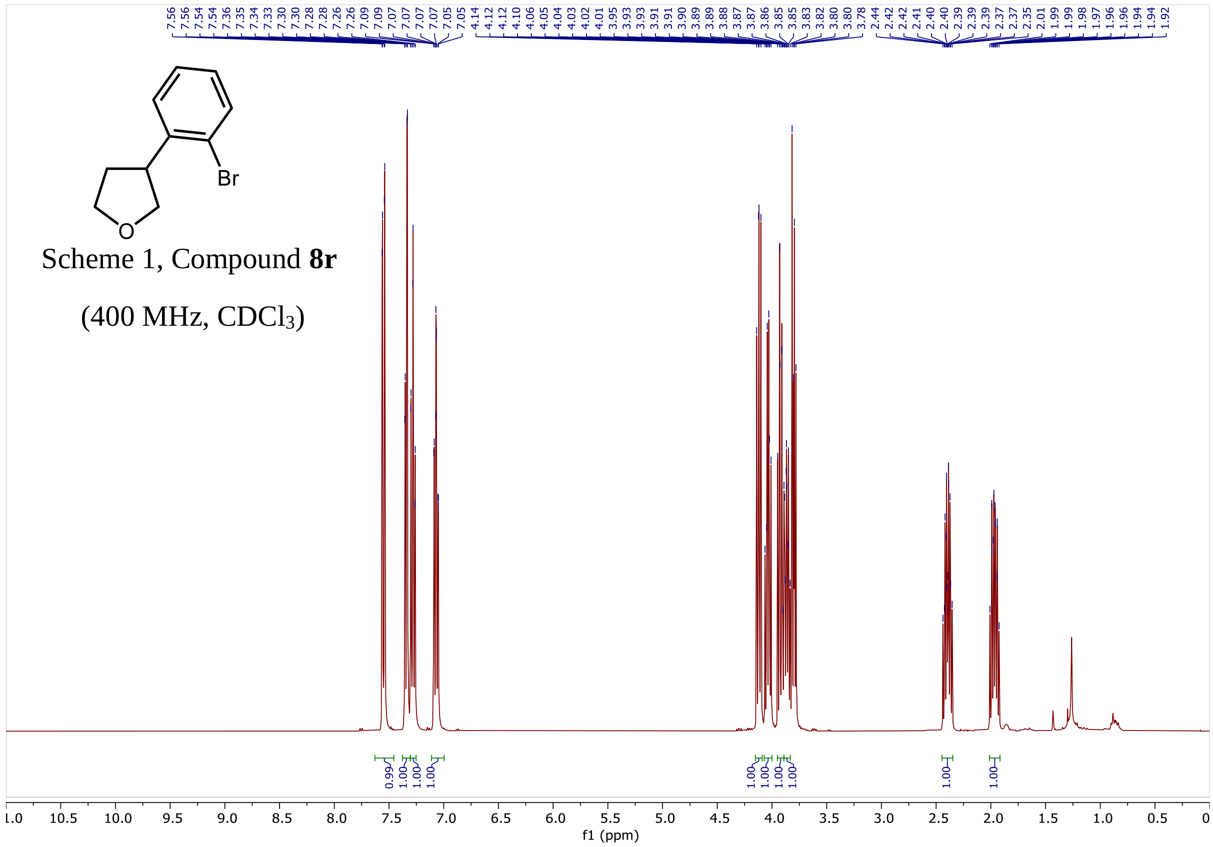
7.56 7.56 7.54 7.54 7.36 7.35 7.34 7.33 7.30 7.30 7.28 7.28 7.26 7.26 7.09 7.09 7.07 7.07 7.07 7.05 7.05 4.14 4.12 4.10 4.06 4.05 4.04 4.03 4.02 4.01 3.95 3.93 3.93 3.91 3.91 3.90 3.89 3.89 3.88 3.87 3.87 3.86 3.85 3.85 3.83 3.82 3.80 3.80 3.78 2.44 2.42 2.42 2.41 2.40 2.39 2.39 2.39 2.37 2.37 2.35 2.01 1.99 1.99 1.98 1.97 1.96 1.94 1.94 1.92
Br
O
Scheme 1, Compound **8r**
(400 MHz, $CDCl_3$)
0.99 1.00 1.00 1.00
1.00 1.00 1.00 1.00
1.00
1.00
f1 (ppm)

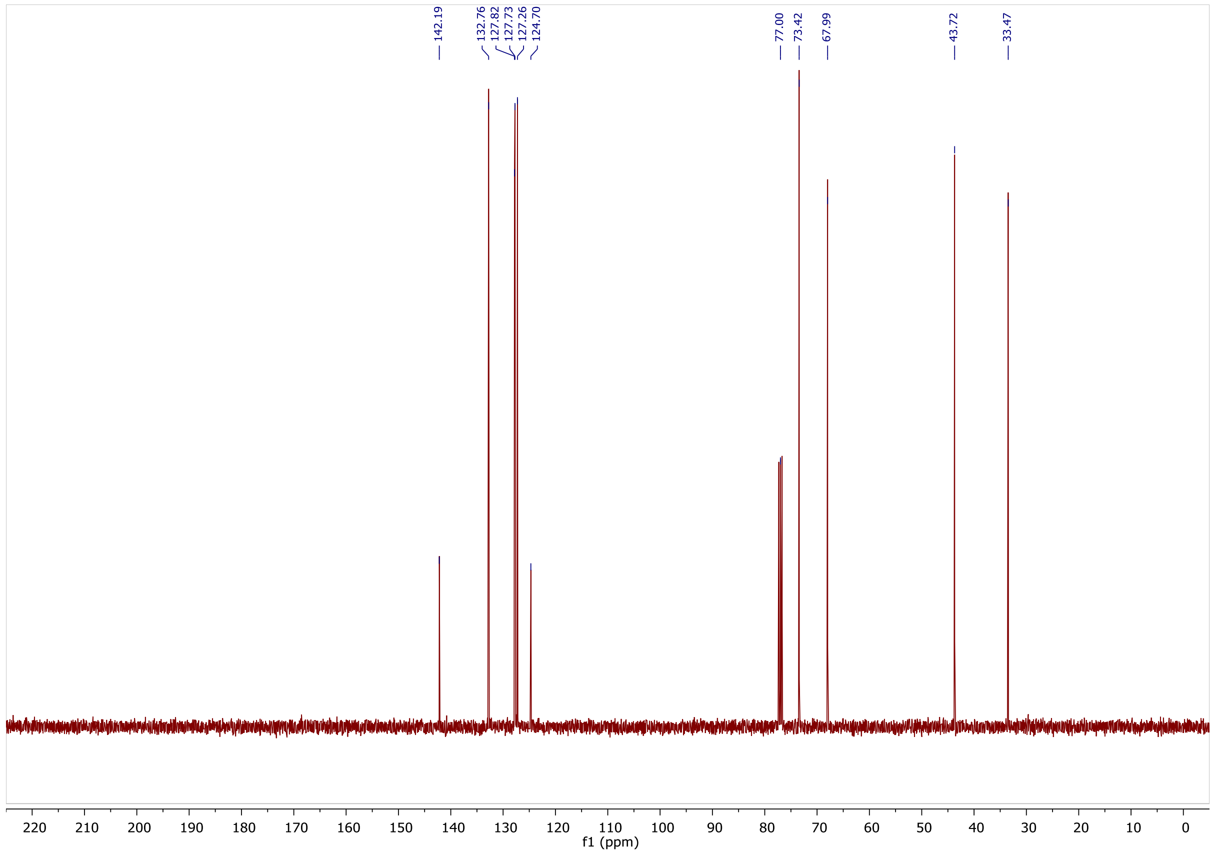
142.19
132.76 127.82 127.73 127.26 124.70
77.00 73.42 67.99
43.72
33.47
f1 (ppm)

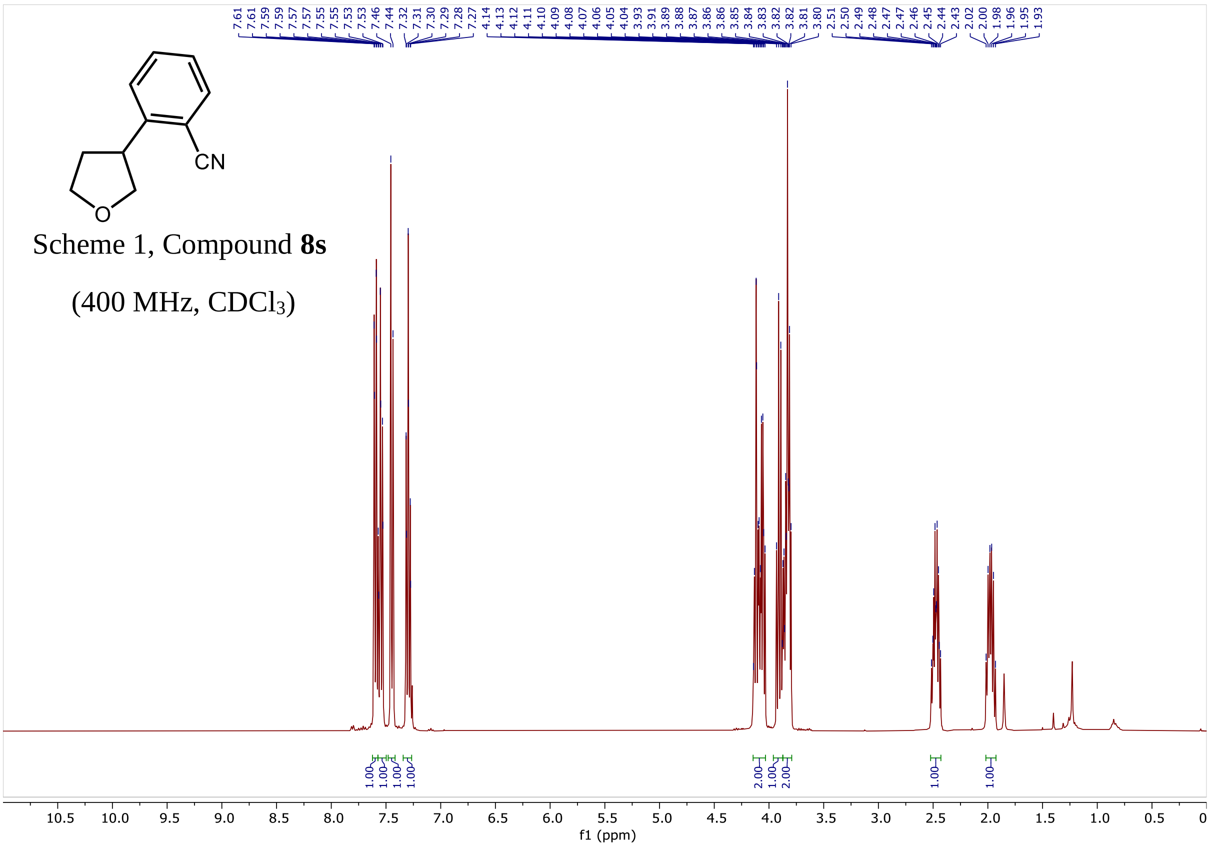
CN
O
Scheme 1, Compound **8s**
(400 MHz, $CDCl_3$)
7.61 7.61 7.59 7.59 7.57 7.57 7.55 7.55 7.53 7.53 7.46 7.44 7.32 7.31 7.30 7.29 7.28 7.27 4.14 4.13 4.12 4.11 4.10 4.09 4.08 4.07 4.06 4.05 4.04 3.93 3.91 3.89 3.88 3.87 3.86 3.86 3.85 3.84 3.83 3.82 3.82 3.81 3.80 2.51 2.50 2.49 2.48 2.47 2.47 2.46 2.45 2.44 2.43 2.02 2.00 1.98 1.96 1.95 1.93
1.00 1.00 1.00 1.00 2.00 1.00 2.00 1.00 1.00
10.5 10.0 9.5 9.0 8.5 8.0 7.5 7.0 6.5 6.0 5.5 5.0 4.5 4.0 3.5 3.0 2.5 2.0 1.5 1.0 0.5 0
f1 (ppm)

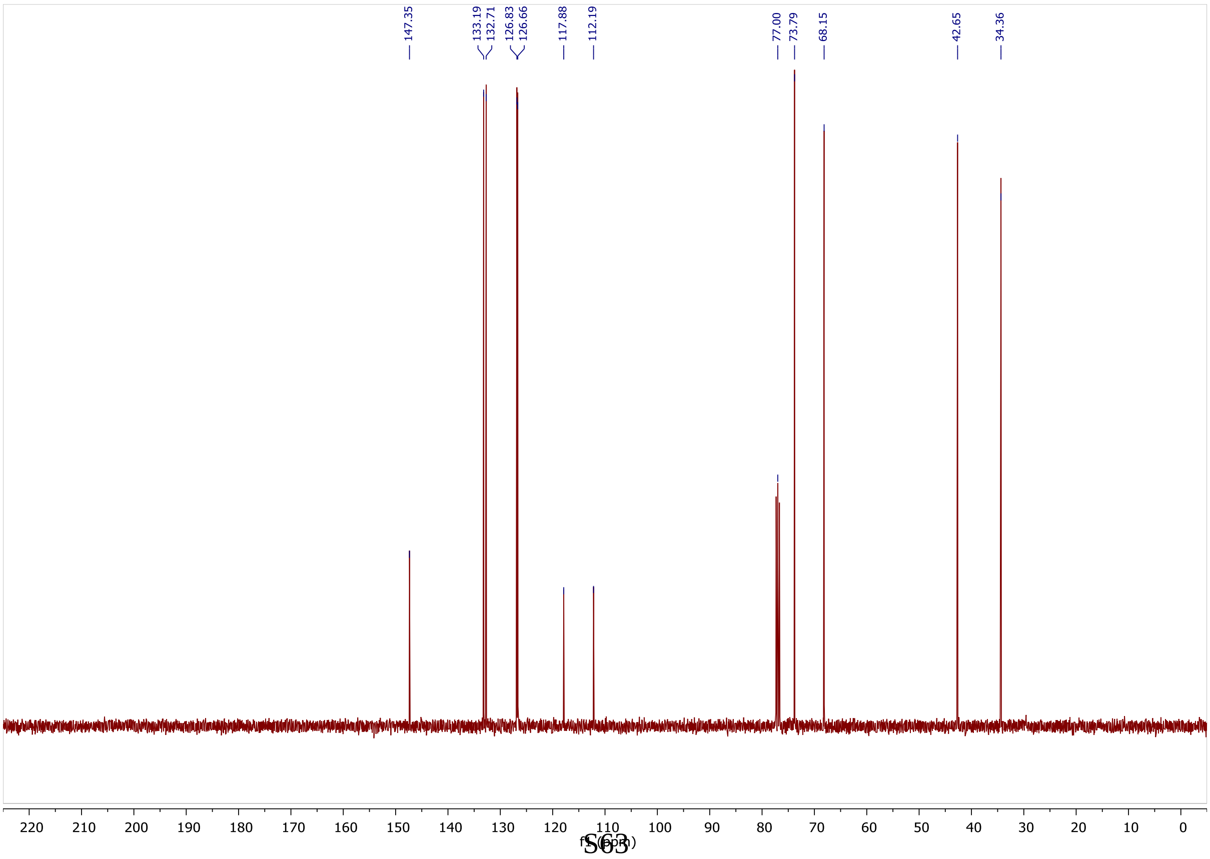
147.35 133.19 132.71 126.83 126.66 117.88 112.19 77.00 73.79 68.15 42.65 34.36
220 210 200 190 180 170 160 150 140 130 120 110 100 90 80 70 60 50 40 30 20 10 0
f1 (ppm)

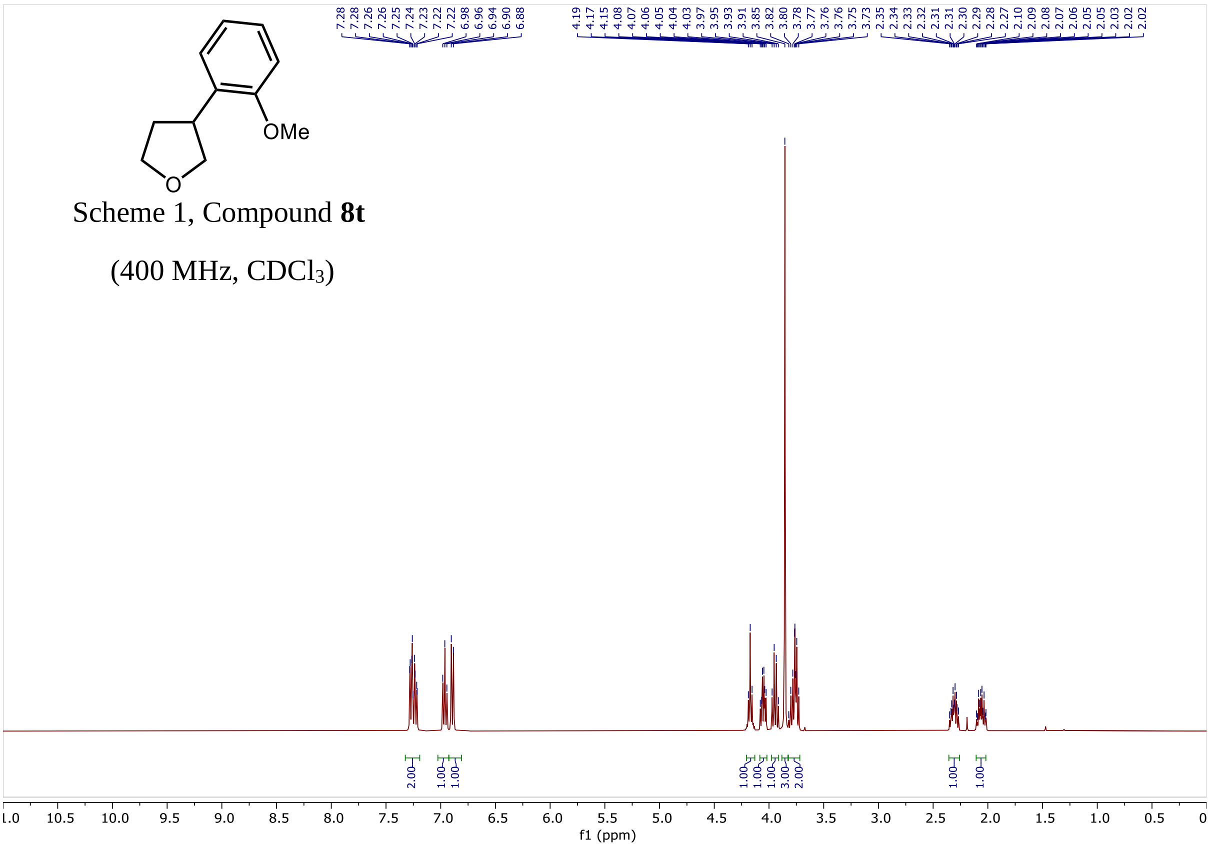

Scheme 1, Compound **8t**

(400 MHz, $CDCl_3$)

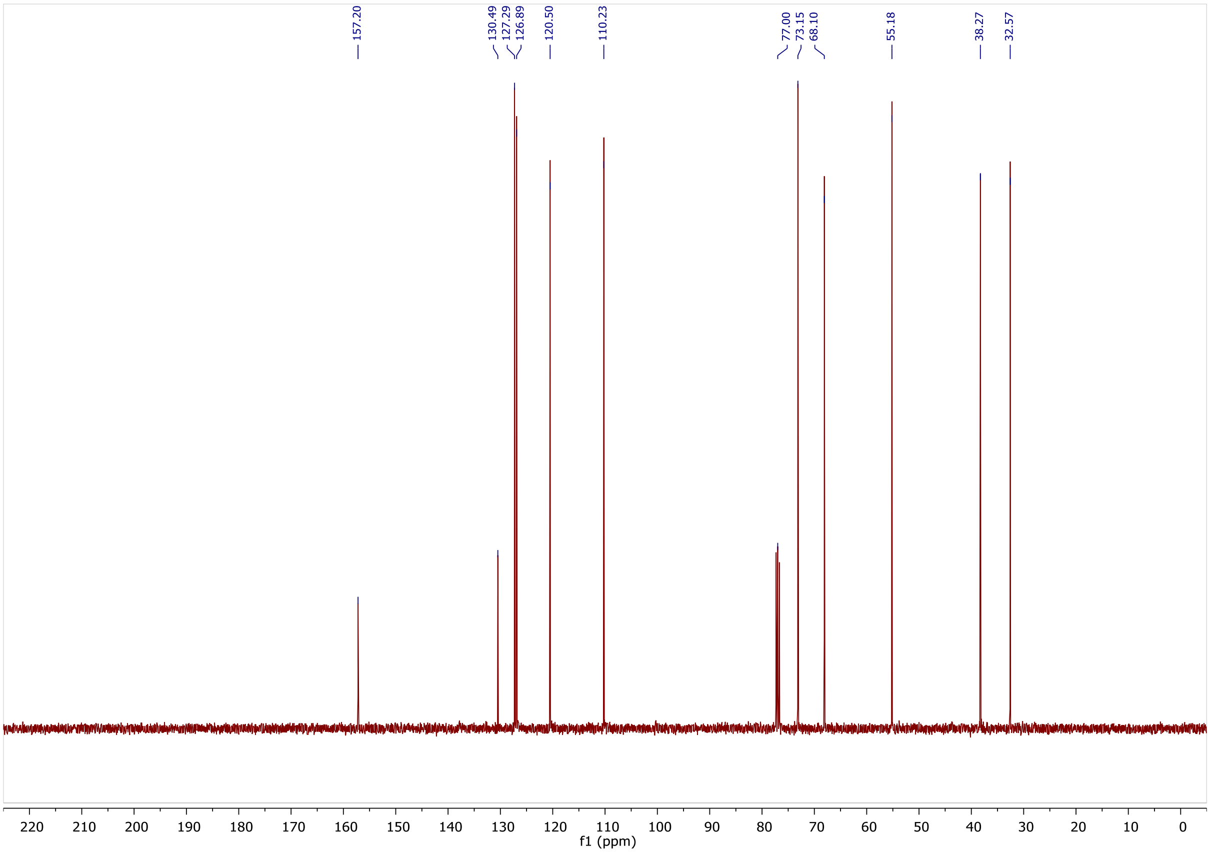

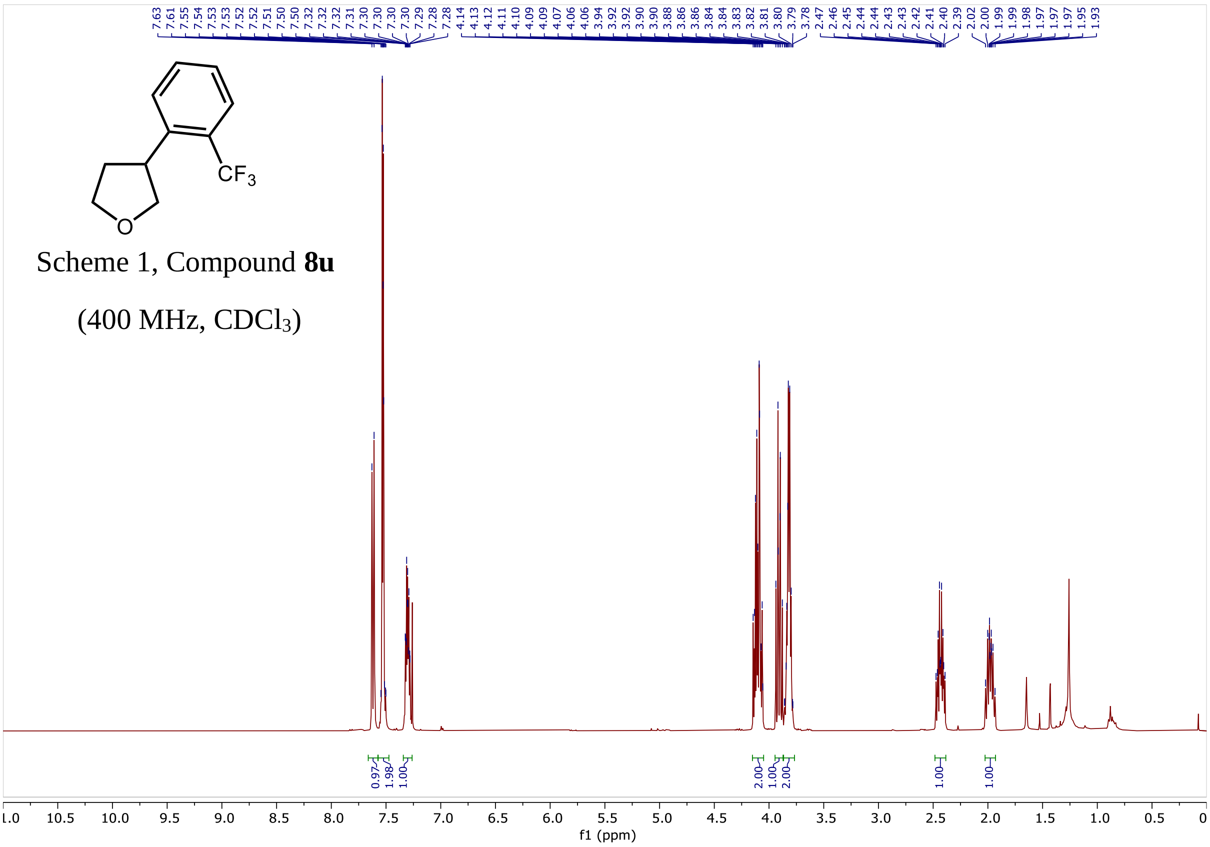

CF3
O
Scheme 1, Compound 8u
(400 MHz, CDCl3)
7.63 7.61 7.55 7.54 7.53 7.53 7.52 7.52 7.51 7.50 7.50 7.32 7.32 7.32 7.31 7.30 7.30 7.30 7.30 7.29 7.28 7.28 4.14 4.13 4.12 4.11 4.10 4.09 4.09 4.07 4.06 4.06 3.94 3.92 3.92 3.90 3.90 3.88 3.86 3.86 3.84 3.84 3.83 3.82 3.81 3.81 3.80 3.79 3.78 2.47 2.46 2.45 2.44 2.43 2.43 2.42 2.41 2.40 2.39 2.02 2.00 1.99 1.99 1.98 1.97 1.97 1.97 1.95 1.93
0.97 1.98 1.00 2.00 1.00 2.00 1.00 1.00
f1 (ppm)

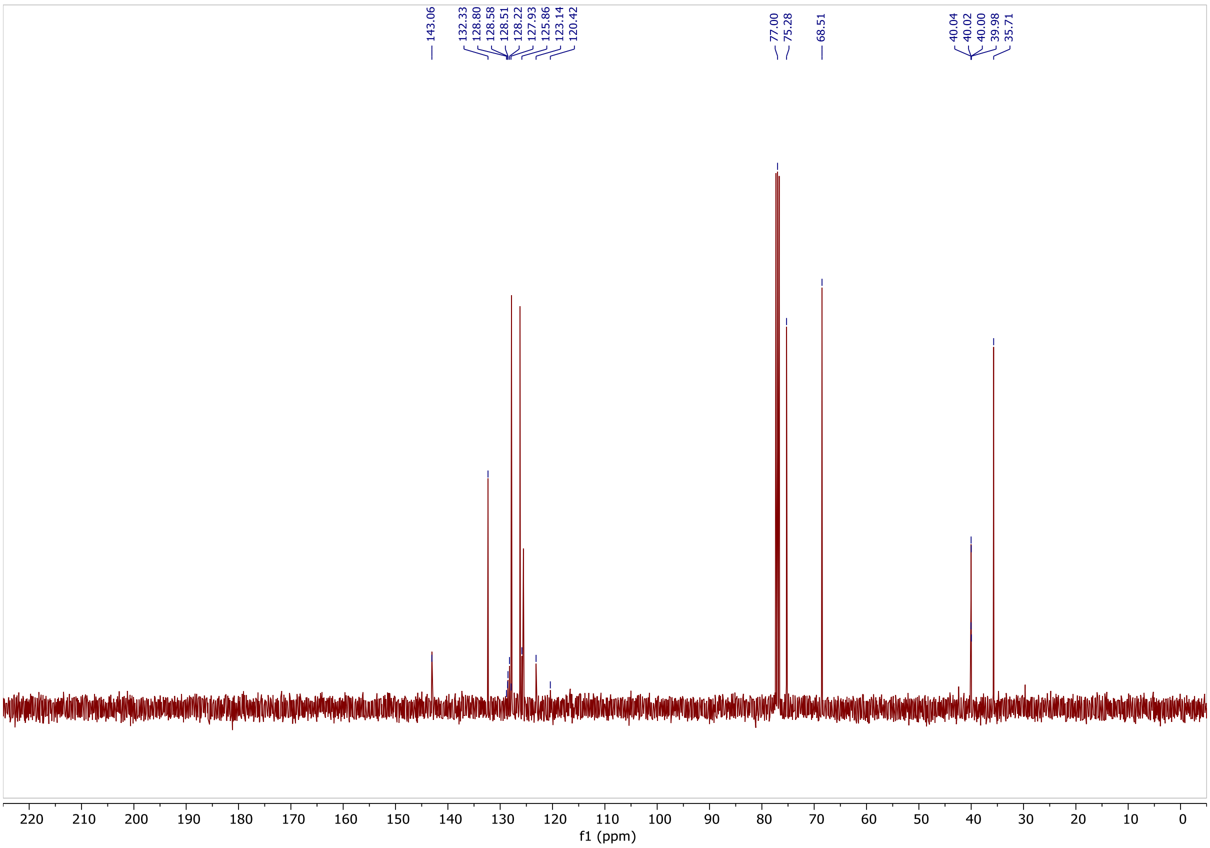

143.06
132.33 128.80 128.58 128.51 128.22 127.93 125.86 123.14 120.42
77.00 75.28
68.51
40.04 40.02 40.00 39.98 35.71
f1 (ppm)

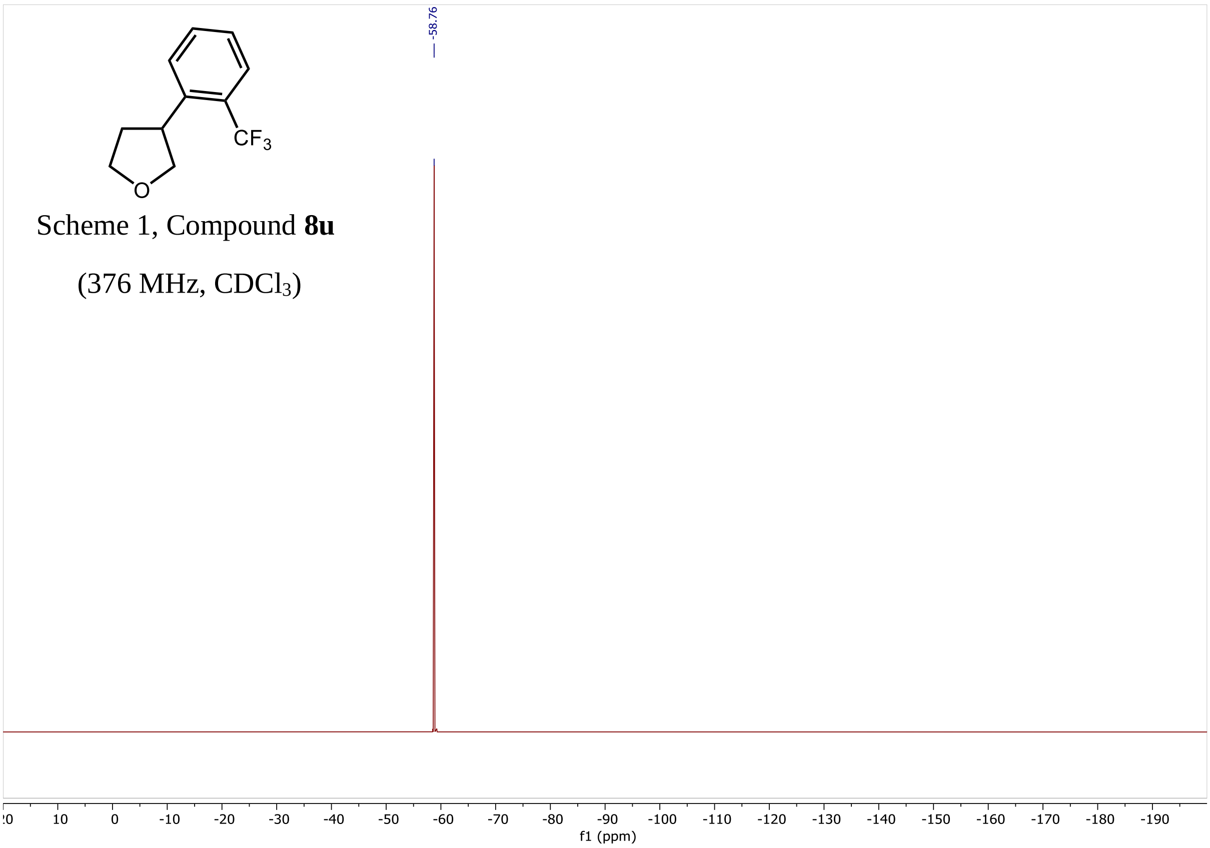

CF3
O
Scheme 1, Compound 8u
(376 MHz, CDCl3)
-58.76
f1 (ppm)

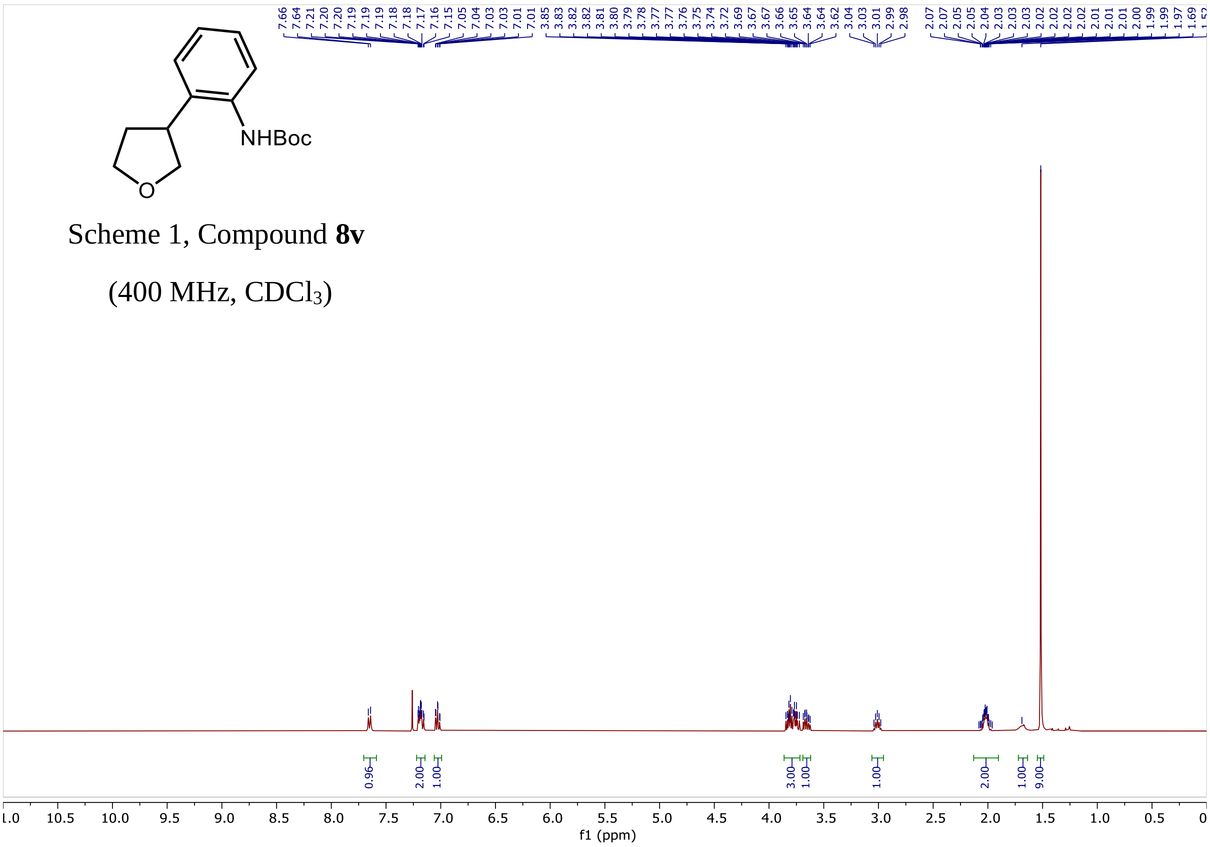
7.66 7.64 7.21 7.20 7.19 7.19 7.18 7.17 7.16 7.15 7.05 7.04 7.03 7.03 7.01 7.01 3.85 3.83 3.82 3.81 3.80 3.79 3.78 3.77 3.76 3.75 3.74 3.72 3.69 3.67 3.67 3.66 3.65 3.64 3.64 3.62 3.04 3.03 3.01 2.99 2.98 2.07 2.07 2.05 2.05 2.04 2.03 2.03 2.03 2.02 2.02 2.02 2.01 2.01 2.00 1.99 1.99 1.97 1.69
NHBoc
O
Scheme 1, Compound **8v**
(400 MHz, $CDCl_3$)
0.96 2.00 1.00 3.00 1.00 1.00 2.00 1.00 9.00
f1 (ppm)

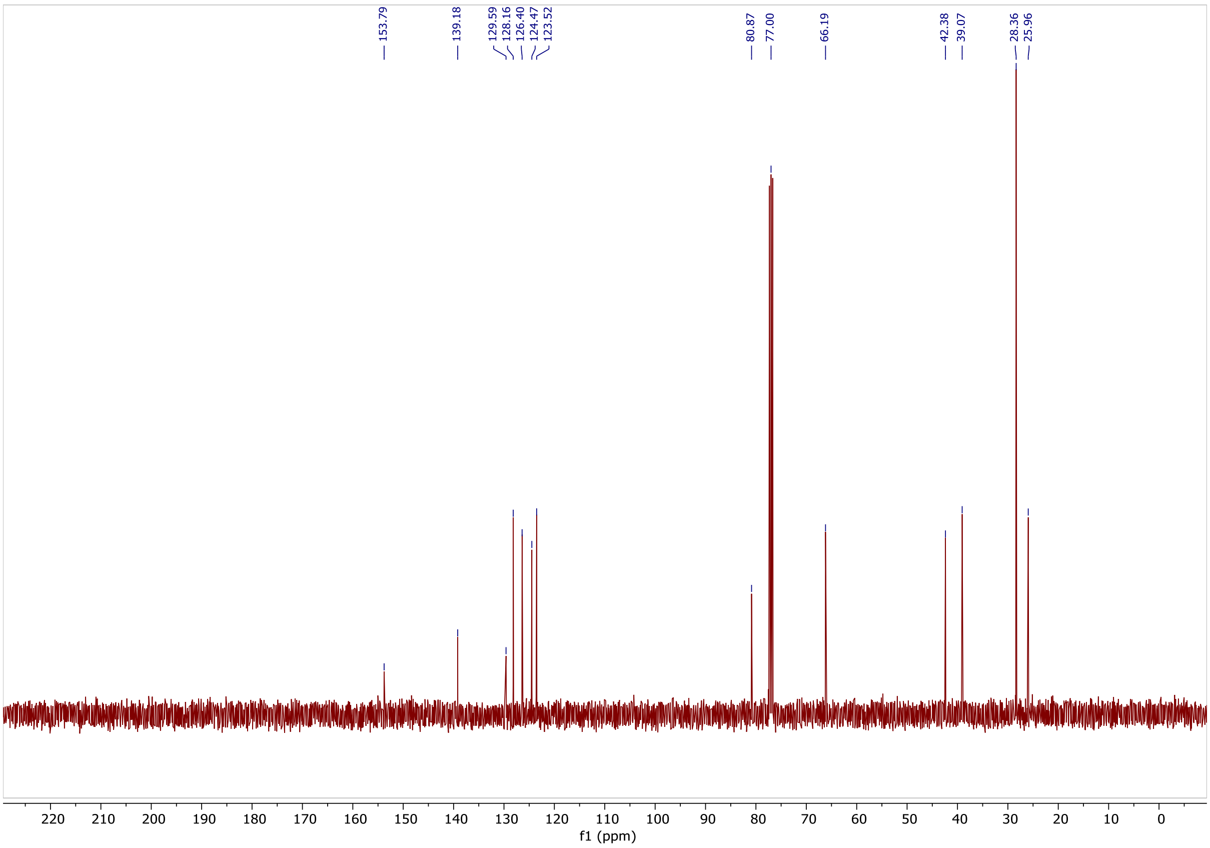
153.79 139.18 129.59 128.16 126.40 124.47 123.52 80.87 77.00 66.19 42.38 39.07 28.36 25.96
f1 (ppm)

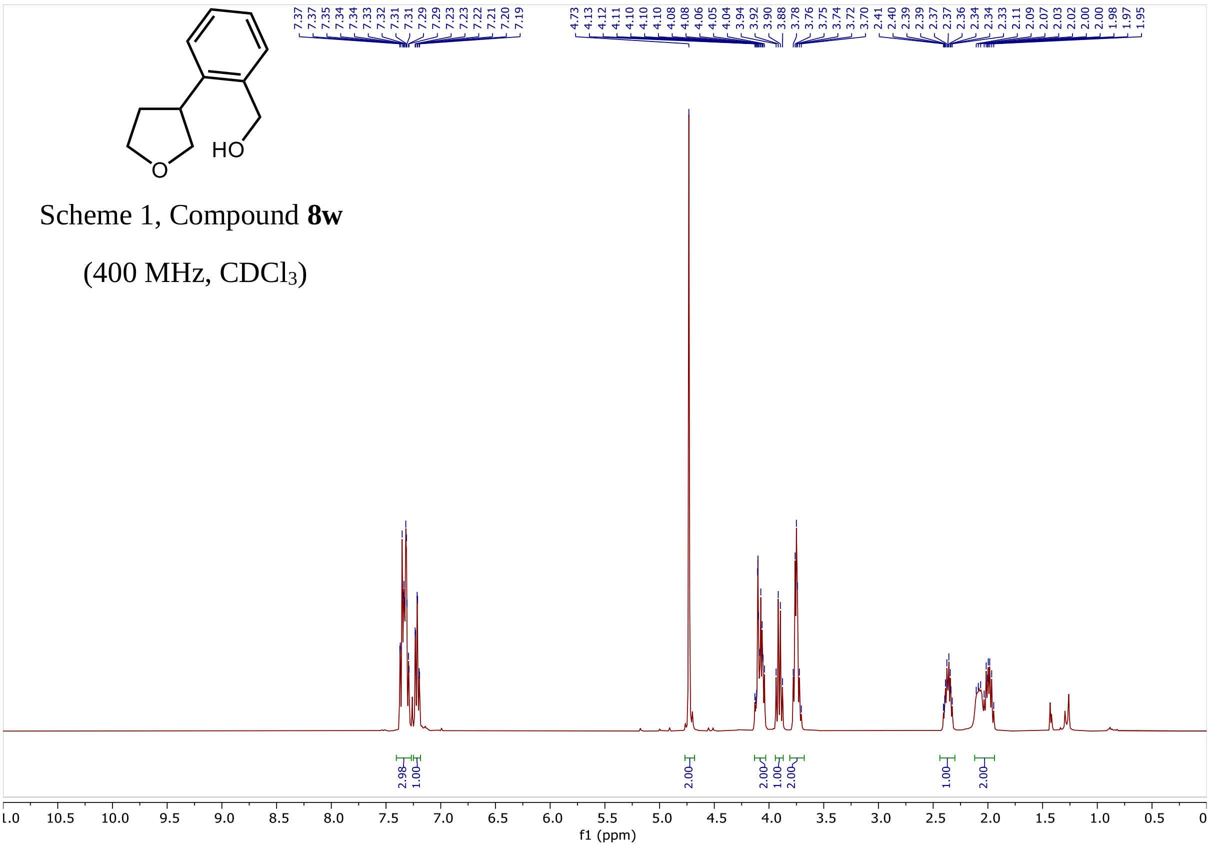
7.37 7.37 7.35 7.34 7.34 7.33 7.32 7.31 7.31 7.29 7.29 7.23 7.23 7.22 7.21 7.20 7.19
4.73 4.13 4.12 4.11 4.10 4.10 4.10 4.08 4.08 4.06 4.05 4.04 3.94 3.92 3.90 3.88 3.78 3.76 3.75 3.74 3.72 3.70
2.41 2.40 2.39 2.39 2.37 2.37 2.36 2.34 2.34 2.33 2.11 2.09 2.07 2.03 2.02 2.00 2.00 1.98 1.97 1.95
HO
O
Scheme 1, Compound **8w**
(400 MHz, CDCl$_3$)
2.98
1.00
2.00
2.00
1.00
2.00
1.00
2.00
f1 (ppm)

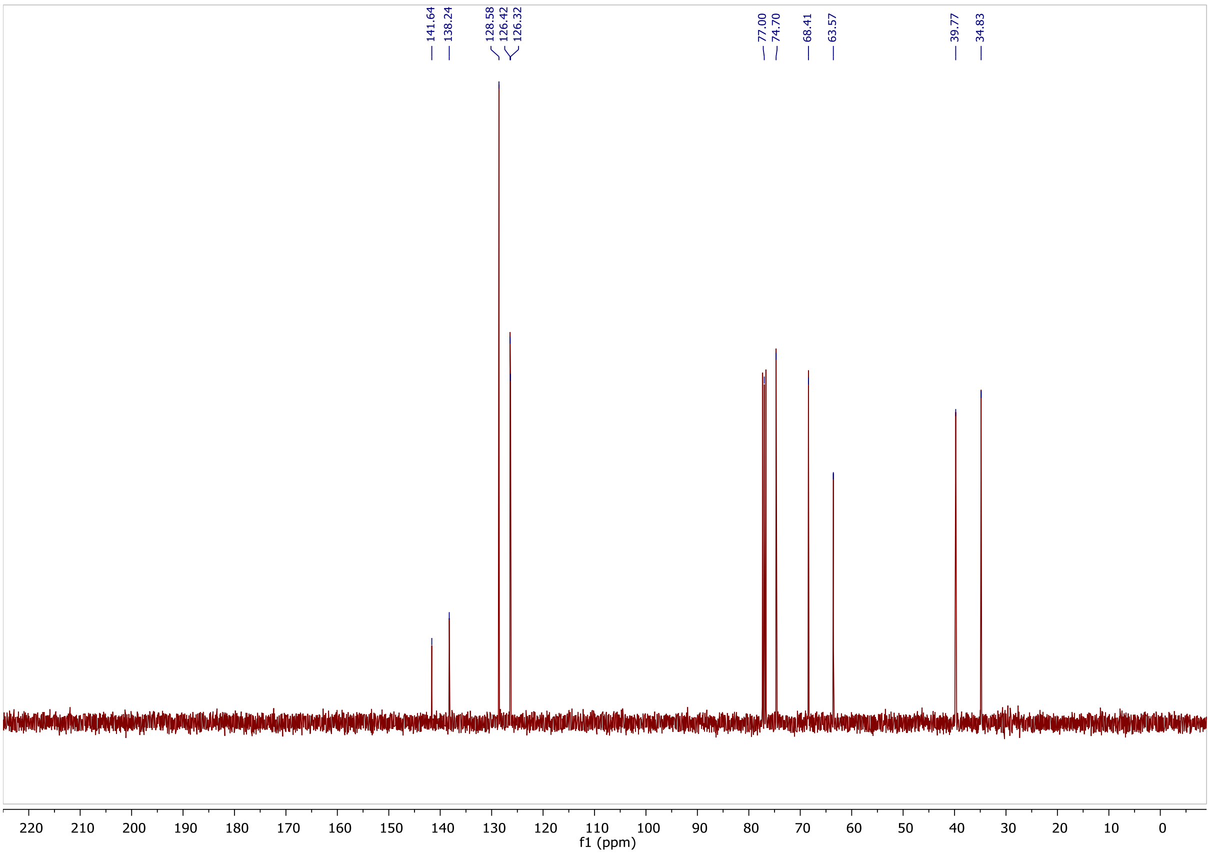
141.64
138.24
128.58
126.42
126.32
77.00
74.70
68.41
63.57
39.77
34.83
f1 (ppm)

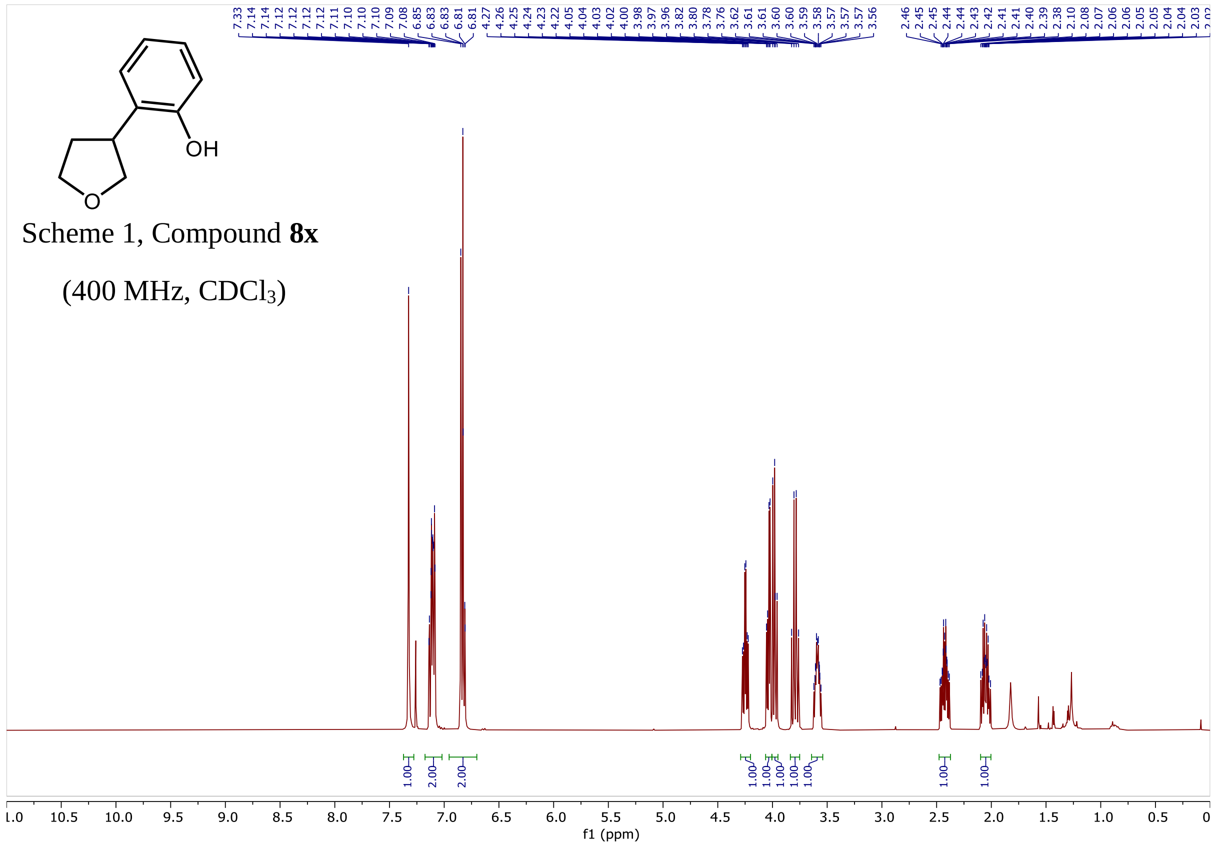
OH
O
Scheme 1, Compound **8x**
(400 MHz, CDCl3)
f1 (ppm)

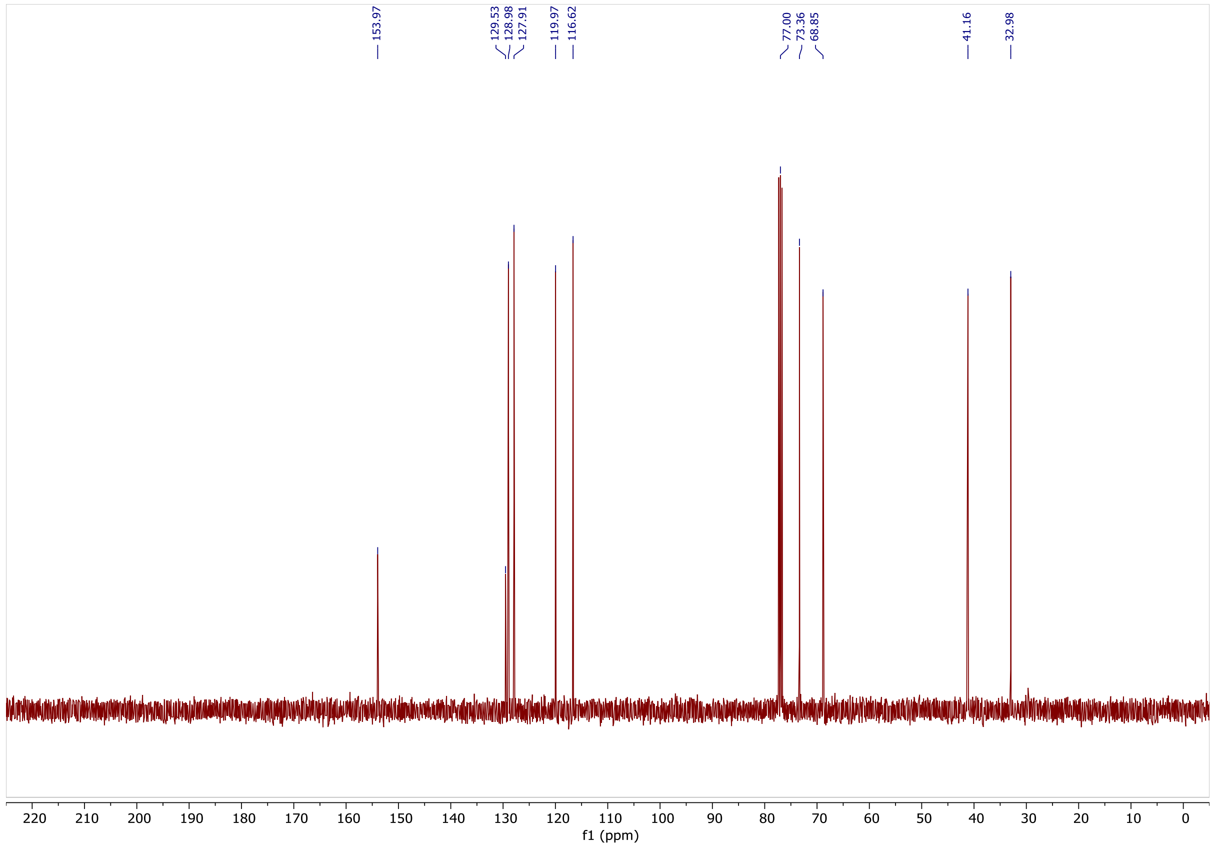
153.97
129.53
128.98
127.91
119.97
116.62
77.00
73.36
68.85
41.16
32.98
f1 (ppm)

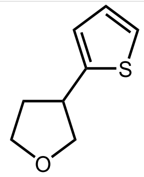

Scheme 1, Compound **8y**

(400 MHz, $CDCl_3$)

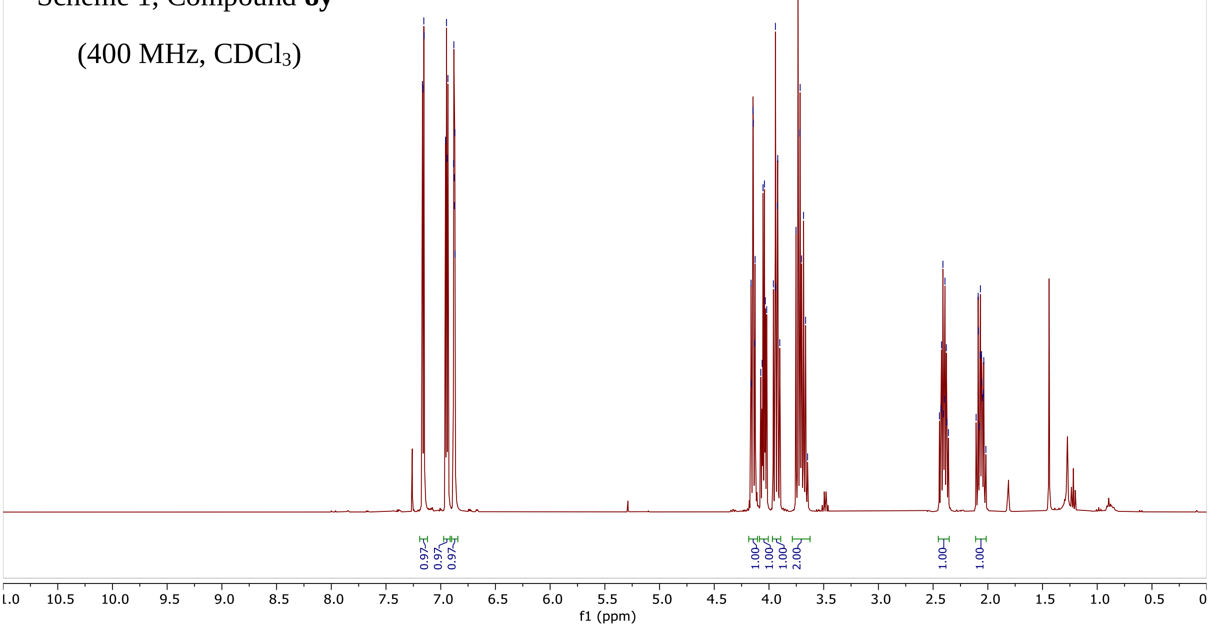

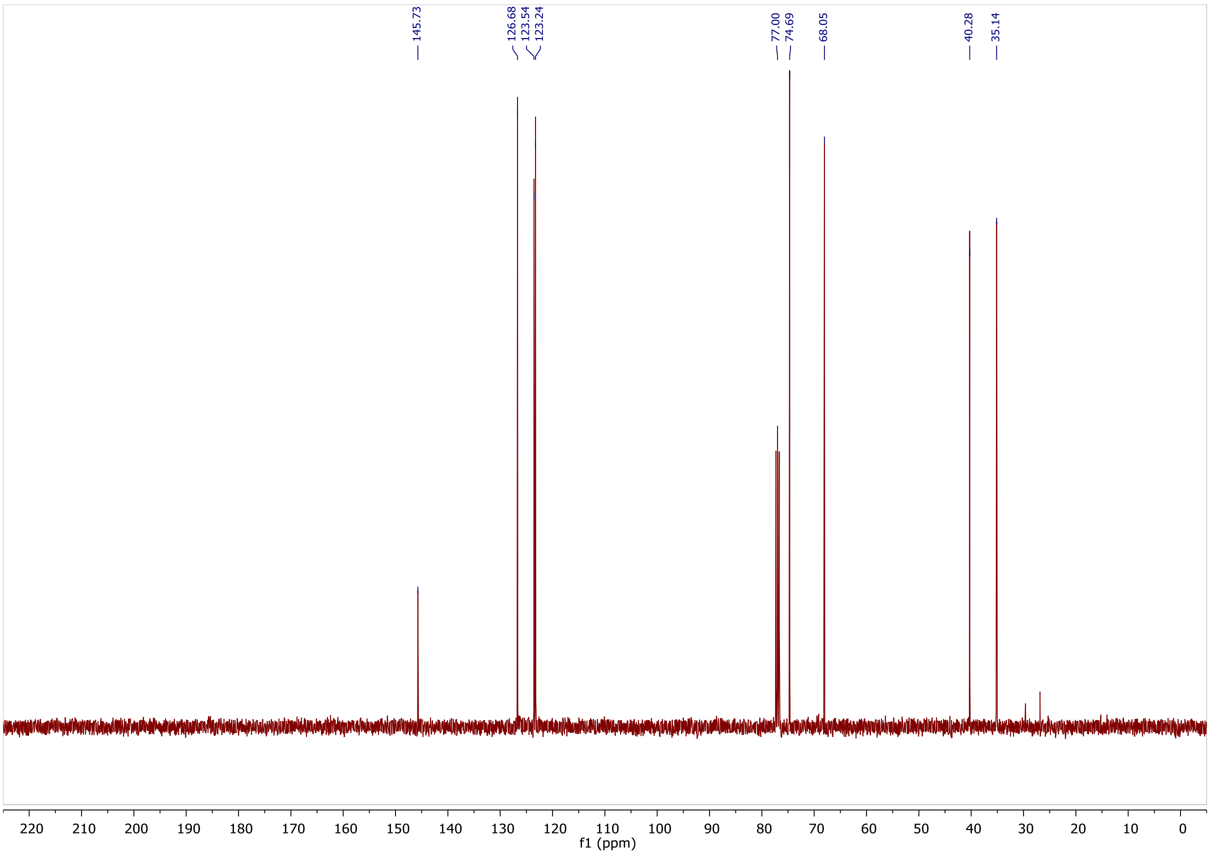

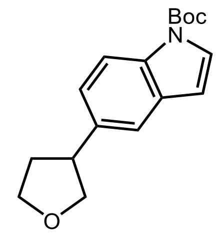

Scheme 1, Compound **8z**

(500 MHz, $CDCl_3$)

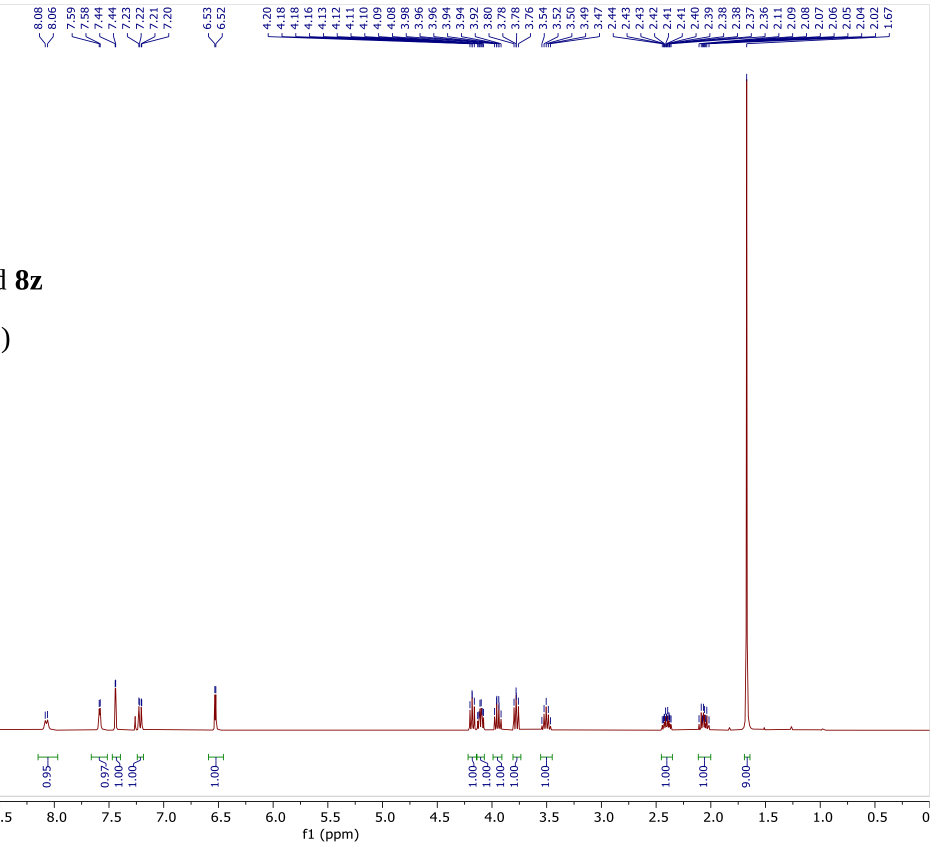

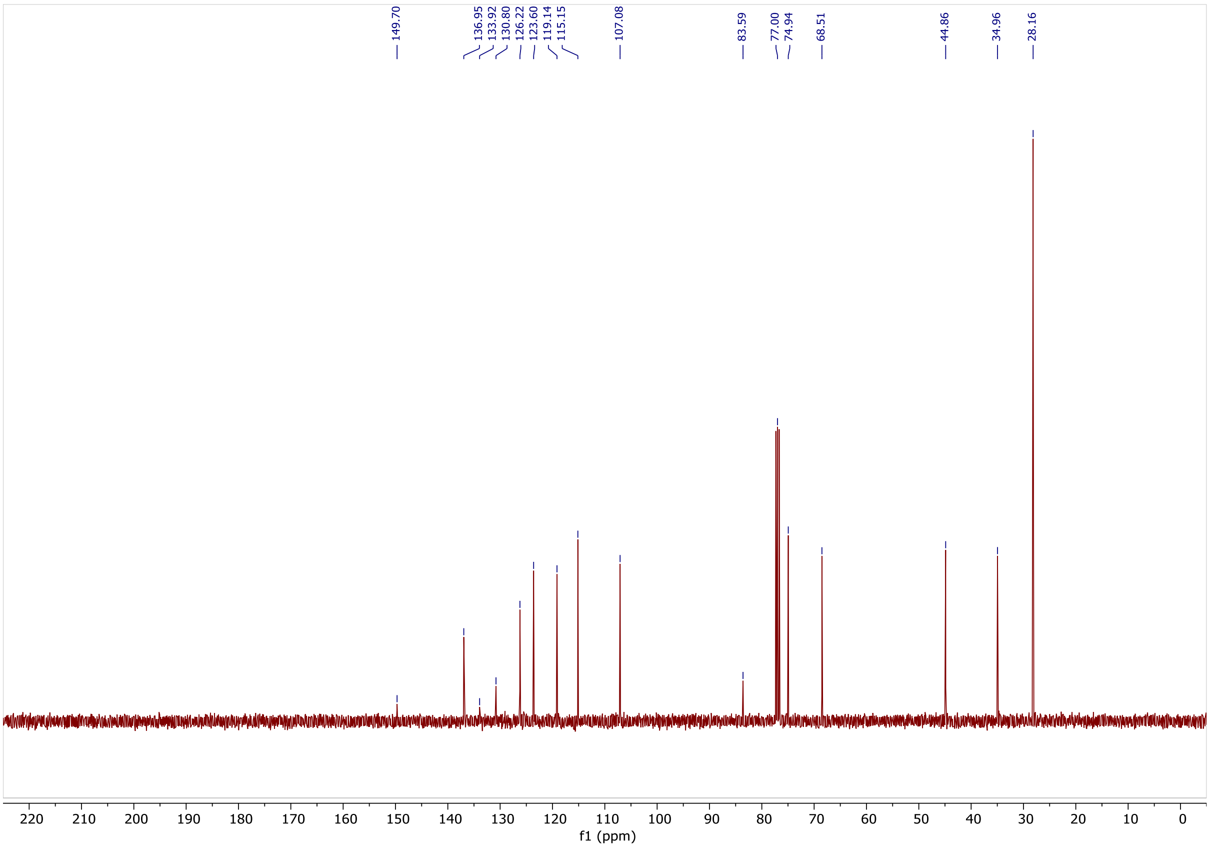

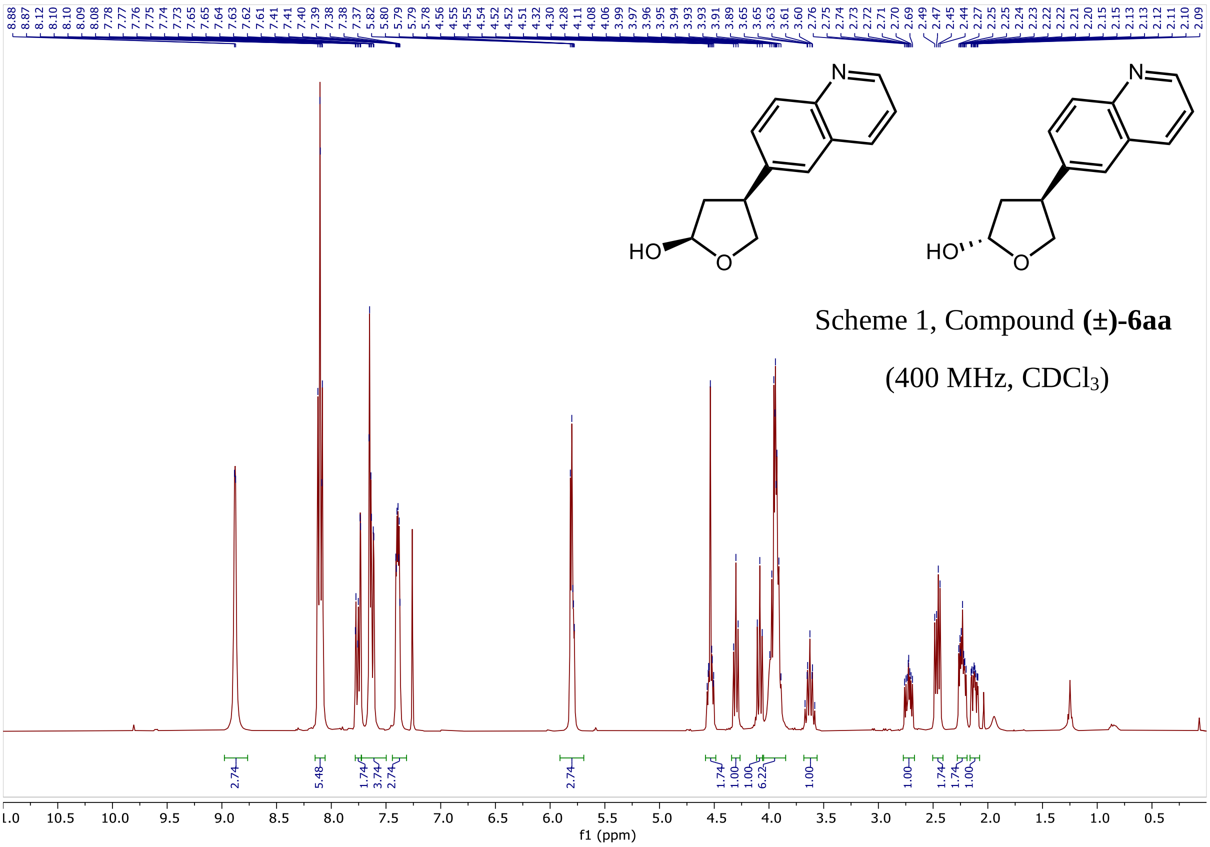
8.88 8.87 8.12 8.10 8.10 8.09 8.08 7.78 7.77 7.76 7.75 7.74 7.73 7.65 7.65 7.64 7.63 7.62 7.61 7.41 7.40 7.39 7.38 7.38 7.37 5.82 5.80 5.79 5.79 5.78 4.56 4.55 4.55 4.54 4.52 4.52 4.51 4.32 4.30 4.28 4.11 4.08 4.06 3.99 3.97 3.96 3.95 3.94 3.93 3.93 3.91 3.89 3.65 3.65 3.63 3.61 3.60 2.76 2.75 2.74 2.73 2.72 2.71 2.70 2.69 2.49 2.47 2.45 2.44 2.27 2.25 2.25 2.24 2.23 2.22 2.22 2.21 2.20 2.15 2.15 2.13 2.13 2.12 2.11 2.10 2.09
N
HO
O
N
HO
O
Scheme 1, Compound **(±)-6aa**
(400 MHz, CDCl3)
2.74
5.48
1.74
3.74
2.74
2.74
1.74
1.00
1.00
6.22
1.00
1.00
1.74
1.74
1.00
1.0 10.5 10.0 9.5 9.0 8.5 8.0 7.5 7.0 6.5 6.0 5.5 5.0 4.5 4.0 3.5 3.0 2.5 2.0 1.5 1.0 0.5
f1 (ppm)

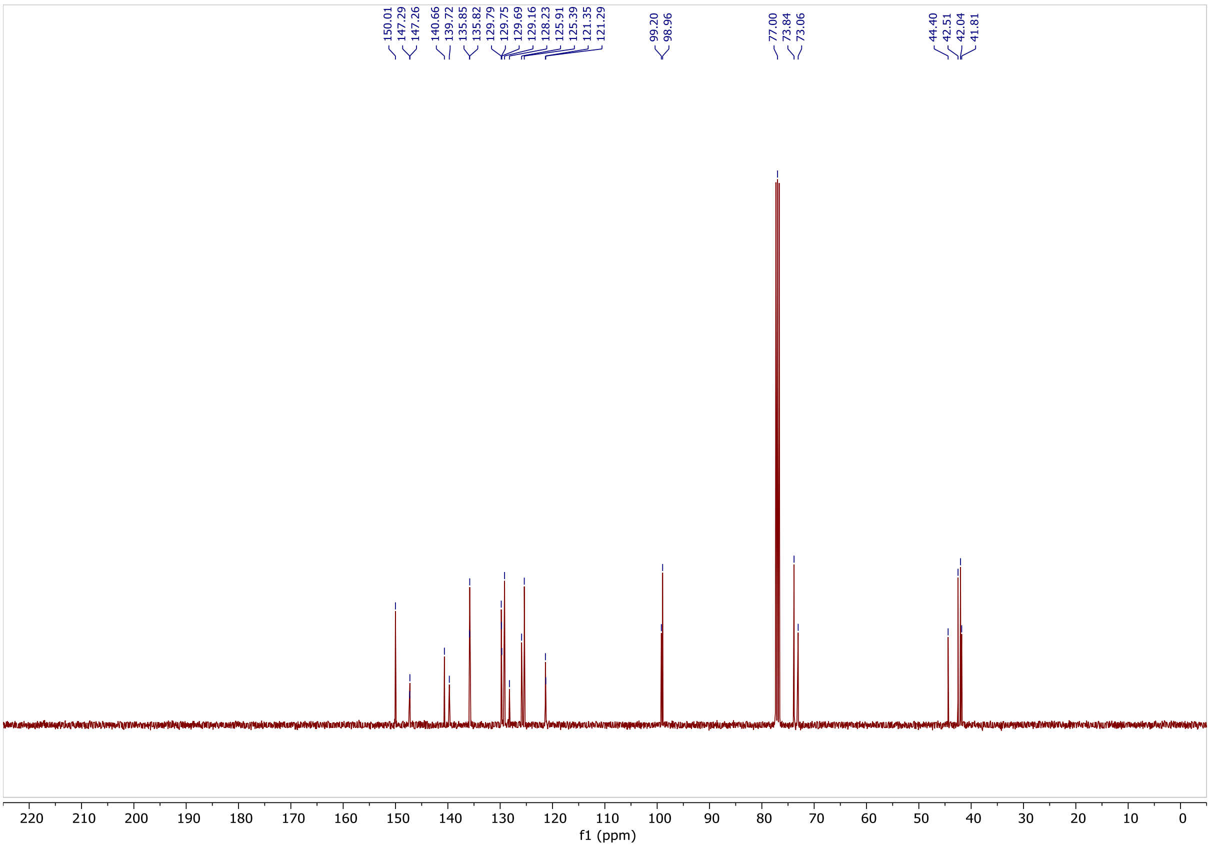
150.01 147.29 147.26 140.66 139.72 135.85 135.82 129.79 129.75 129.69 129.16 128.23 125.91 125.39 121.35 121.29
99.20 98.96
77.00 73.84 73.06
44.40 42.51 42.04 41.81
220 210 200 190 180 170 160 150 140 130 120 110 100 90 80 70 60 50 40 30 20 10 0
f1 (ppm)

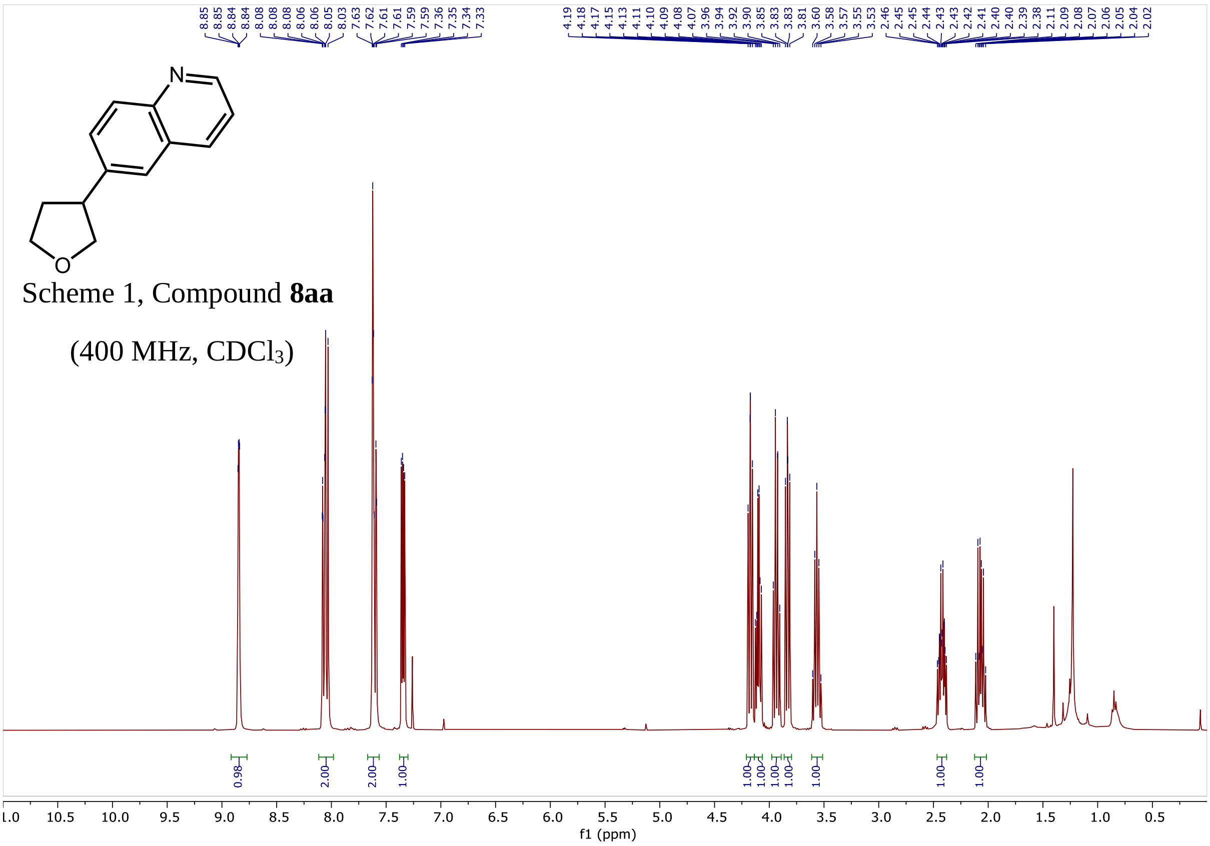
Scheme 1, Compound **8aa**
(400 MHz, $CDCl_3$)
f1 (ppm)

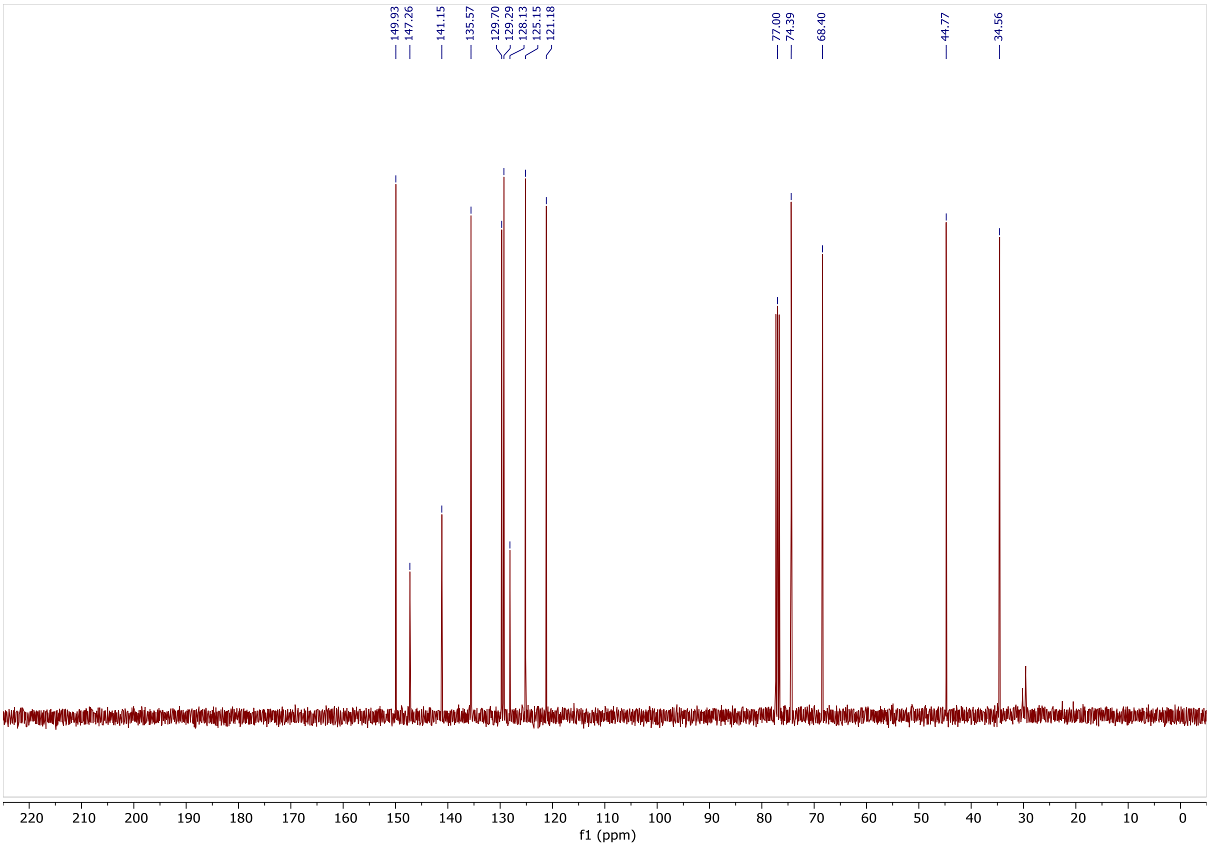
149.93
147.26
141.15
135.57
129.70
129.29
128.13
125.15
121.18
77.00
74.39
68.40
44.77
34.56
f1 (ppm)

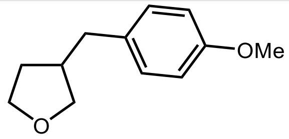

Scheme 2, Compound **10**

(500 MHz, $CDCl_3$)

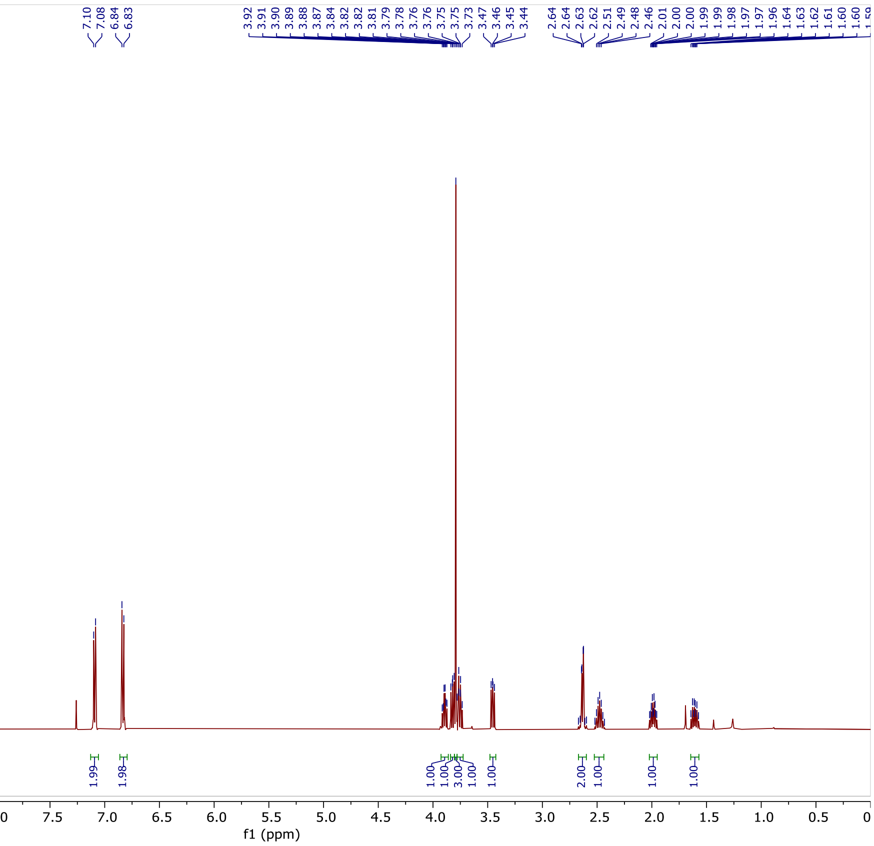

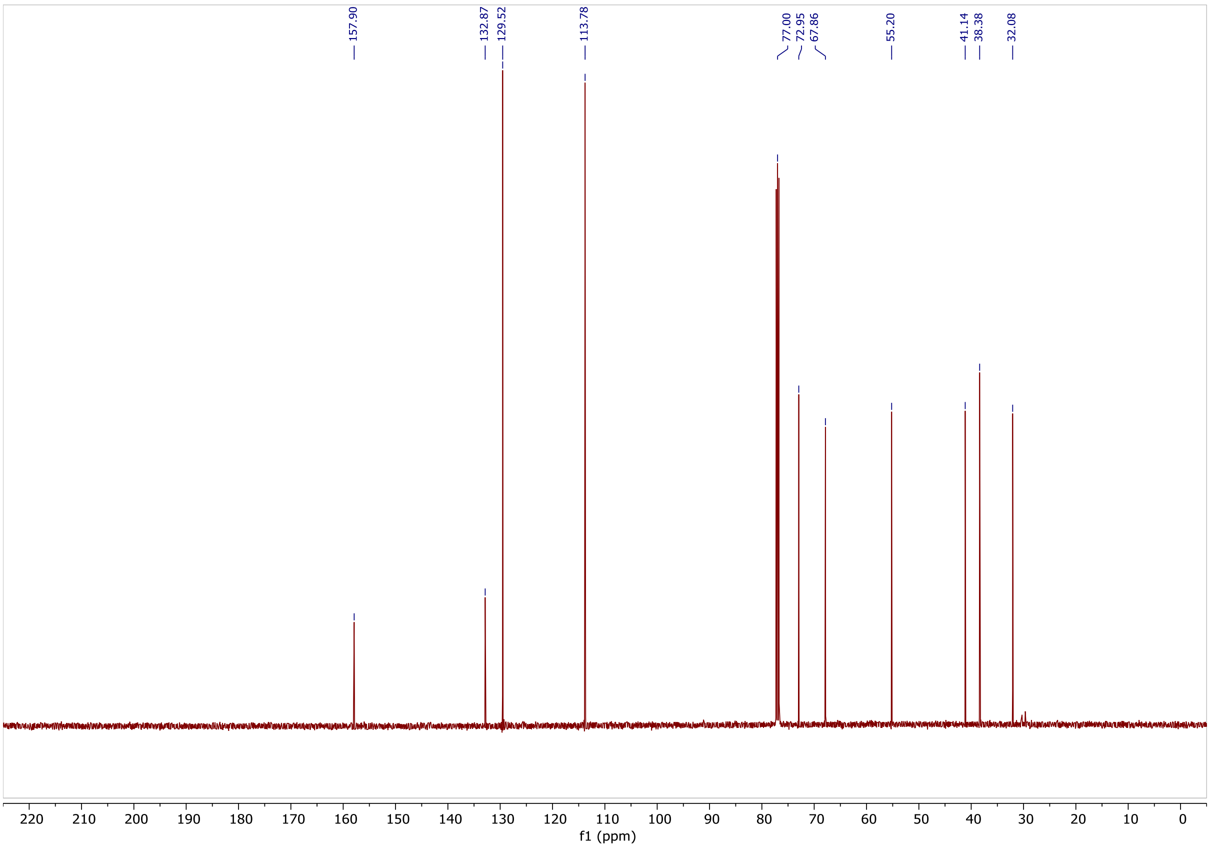

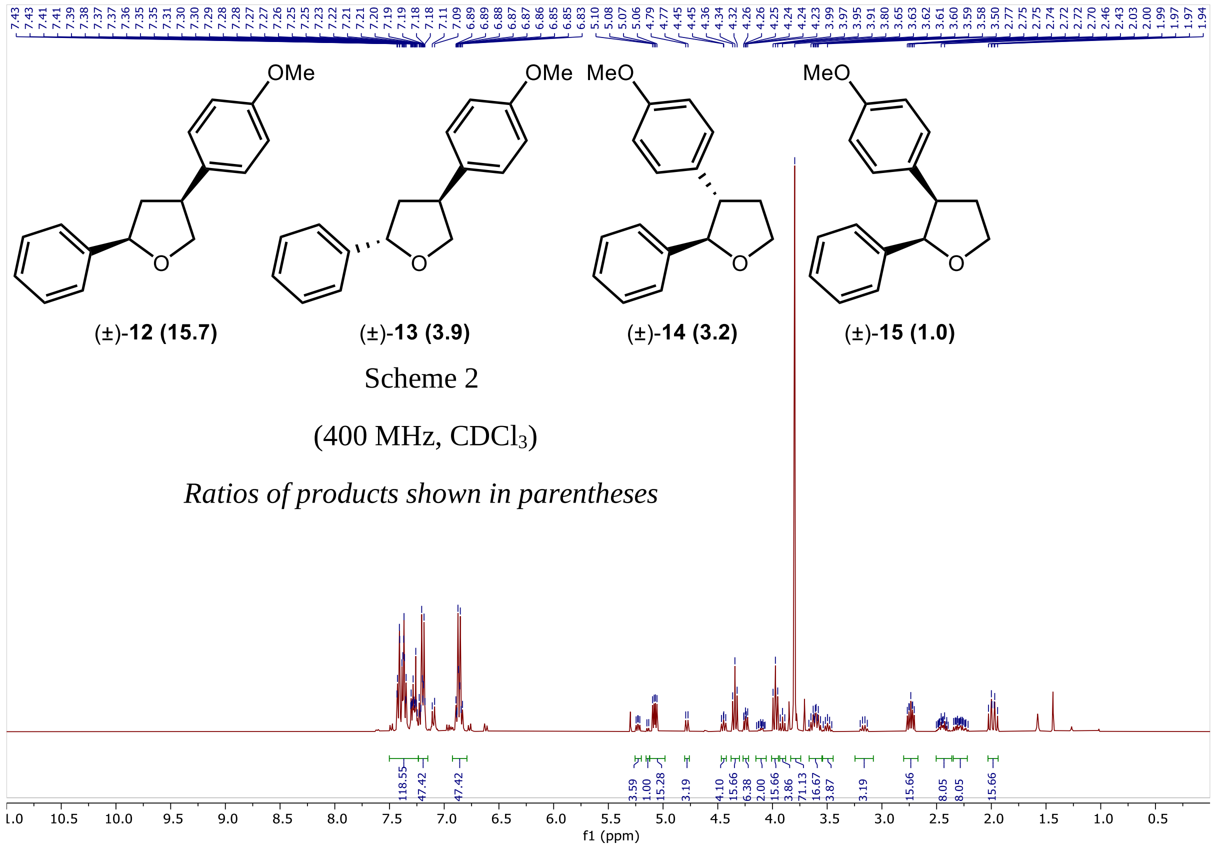
7.43 7.43 7.41 7.39 7.38 7.37 7.37 7.36 7.35 7.31 7.30 7.30 7.29 7.28 7.28 7.27 7.26 7.25 7.25 7.23 7.22 7.21 7.21 7.20 7.19 7.19 7.18 7.18 7.11 7.09 6.89 6.89 6.88 6.87 6.87 6.86 6.85 6.85 6.83 5.10 5.08 5.07 5.06 4.79 4.77 4.45 4.45 4.36 4.34 4.32 4.26 4.26 4.25 4.24 4.24 4.23 3.99 3.97 3.95 3.91 3.80 3.65 3.63 3.62 3.61 3.60 3.59 3.58 3.50 2.77 2.75 2.75 2.74 2.72 2.72 2.70 2.46 2.43 2.03 2.00 1.99 1.97 1.97 1.94
OMe
OMe
MeO
MeO
O
(±)-**12** **(15.7)**
(±)-**13** **(3.9)**
(±)-**14** **(3.2)**
(±)-**15** **(1.0)**
Scheme 2
(400 MHz, $CDCl_3$)
Ratios of products shown in parentheses
118.55 47.42 47.42 3.59 1.00 15.28 3.19 4.10 15.66 6.38 2.00 15.66 3.86 71.13 16.67 3.87 3.19 15.66 8.05 8.05 15.66
f1 (ppm)

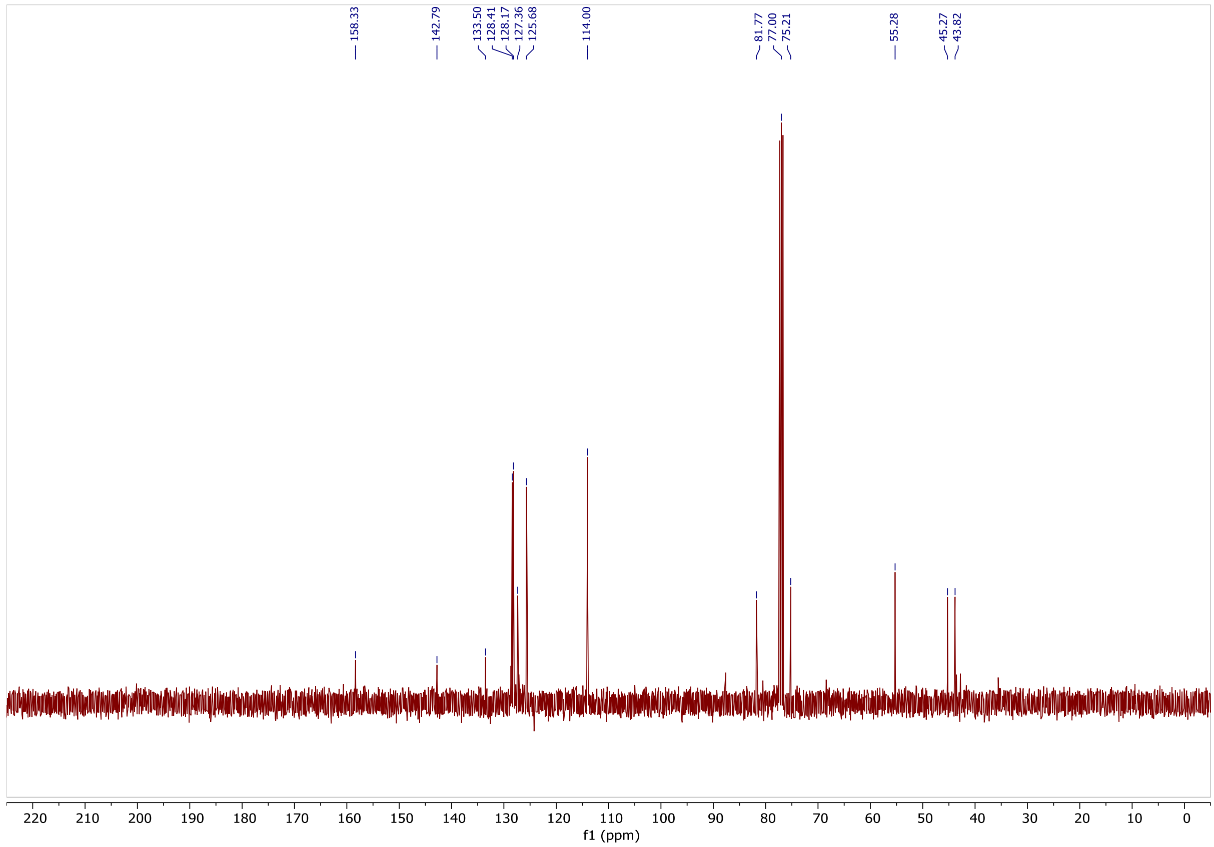
158.33
142.79
133.50 128.41 128.17 127.36 125.68
114.00
81.77 77.00 75.21
55.28
45.27 43.82
f1 (ppm)

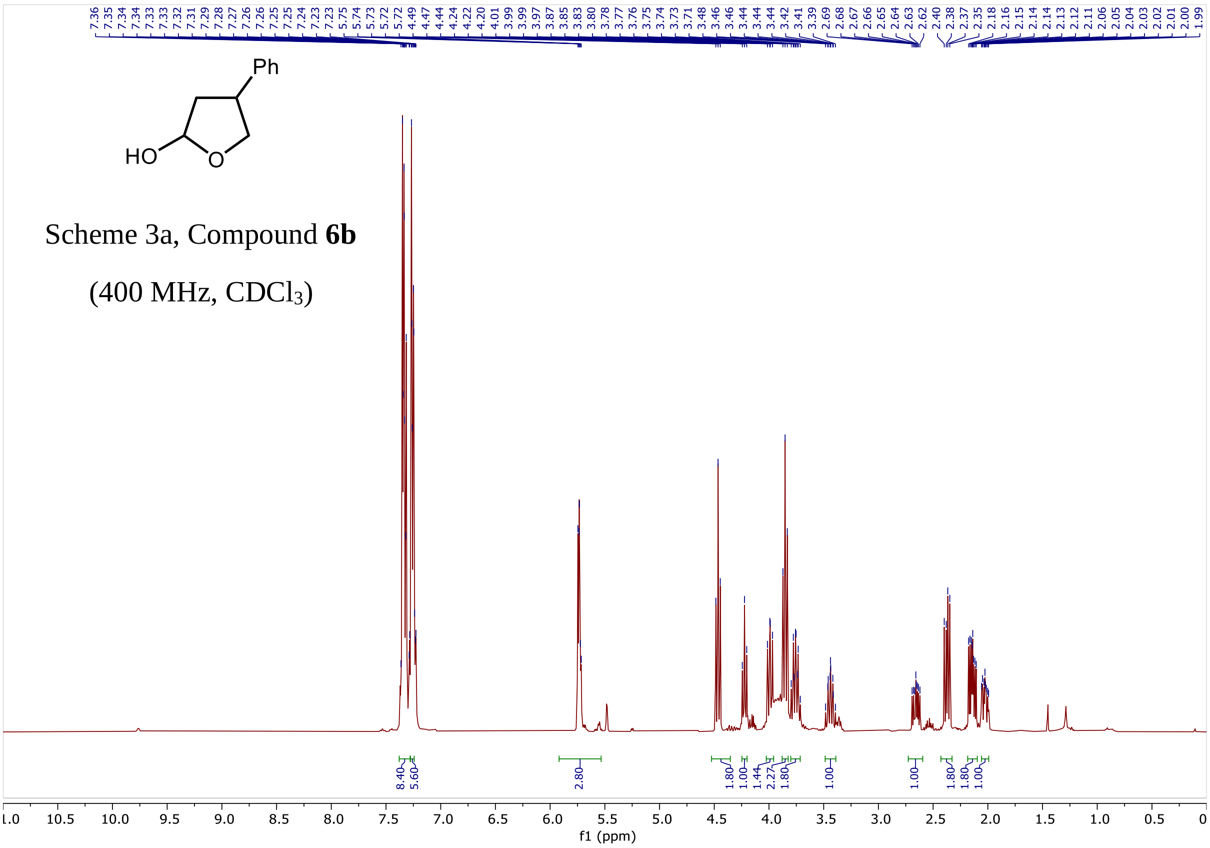
Ph
HO
O
Scheme 3a, Compound **6b**
(400 MHz, $CDCl_3$)
7.36 7.35 7.34 7.34 7.33 7.32 7.31 7.29 7.28 7.27 7.26 7.26 7.25 7.25 7.24 7.23 7.23 5.75 5.74 5.73 5.72 5.72 4.49 4.47 4.44 4.24 4.22 4.20 4.01 3.99 3.99 3.97 3.87 3.85 3.83 3.80 3.78 3.77 3.76 3.75 3.74 3.73 3.71 3.48 3.46 3.46 3.44 3.44 3.44 3.42 3.41 3.39 2.69 2.68 2.67 2.66 2.65 2.64 2.63 2.62 2.40 2.38 2.37 2.35 2.18 2.16 2.15 2.14 2.14 2.13 2.12 2.11 2.06 2.05 2.04 2.03 2.02 2.01 2.00 1.99
8.40 5.60 2.80 1.80 1.00 1.44 2.27 1.80 1.00 1.00 1.80 1.80 1.00
f1 (ppm)

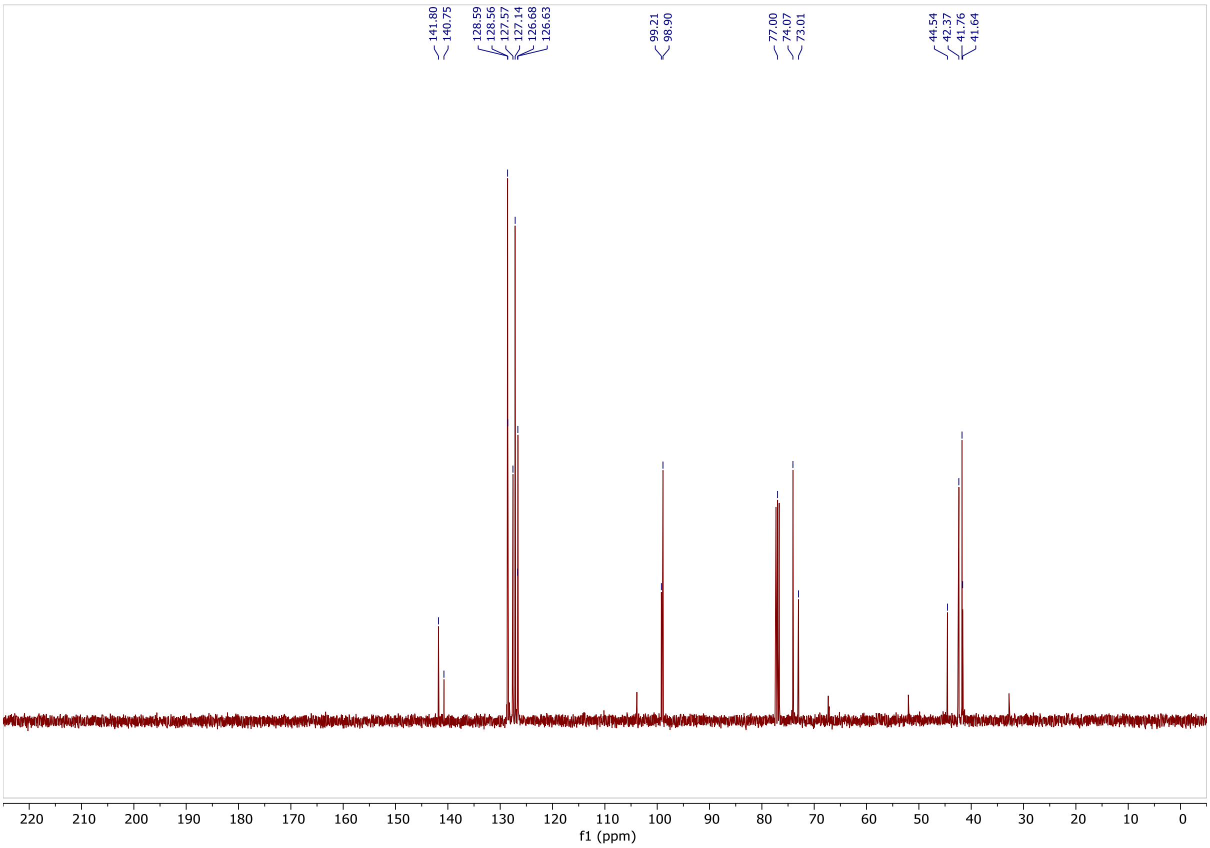
141.80 140.75 128.59 128.56 127.57 127.14 126.68 126.63 99.21 98.90 77.00 74.07 73.01 44.54 42.37 41.76 41.64
f1 (ppm)

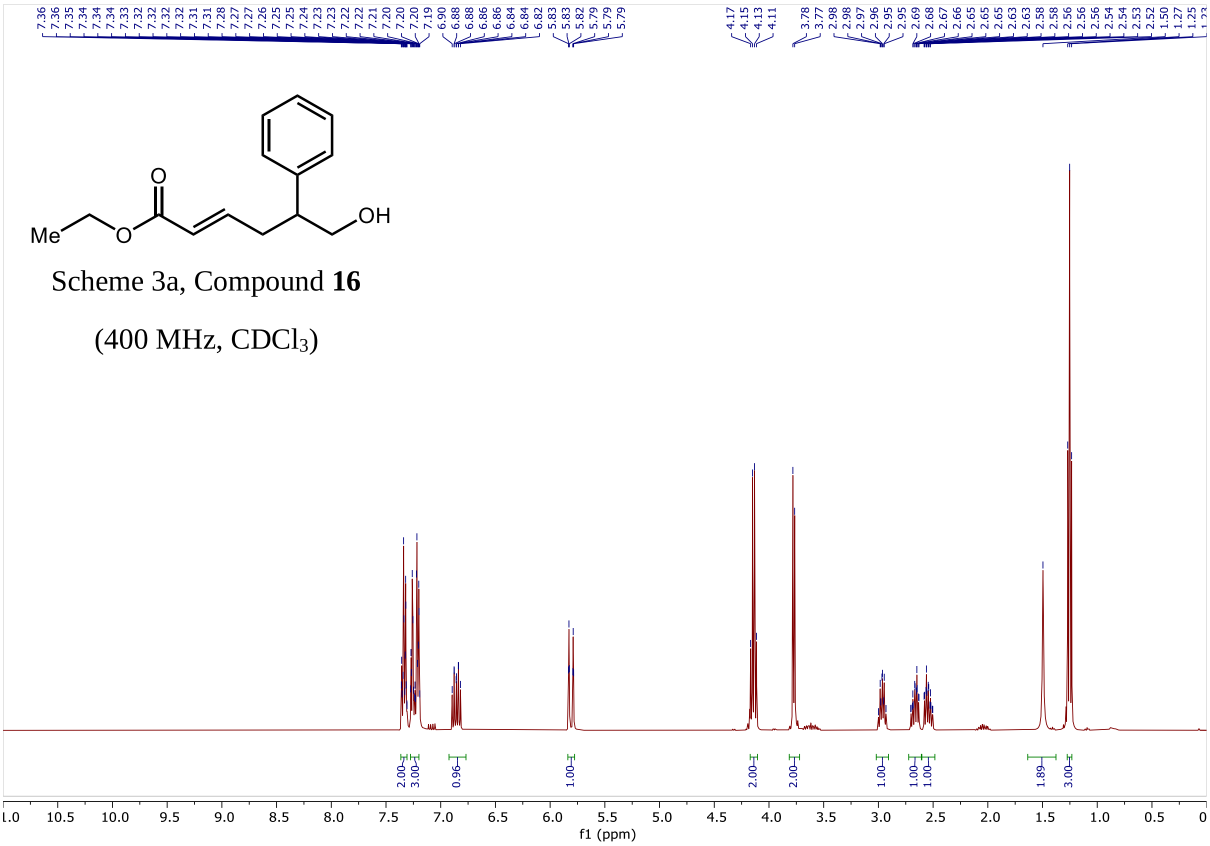

Me
O
O
OH
Scheme 3a, Compound **16**
(400 MHz, $CDCl_3$)
7.36 7.36 7.35 7.34 7.34 7.34 7.33 7.32 7.32 7.32 7.32 7.31 7.31 7.28 7.27 7.27 7.26 7.25 7.25 7.24 7.23 7.23 7.22 7.22 7.21 7.20 7.20 7.20 7.19 6.90 6.88 6.88 6.86 6.86 6.84 6.84 6.82 5.83 5.83 5.82 5.79 5.79
4.17 4.15 4.13 4.11 3.78 3.77 2.98 2.98 2.97 2.96 2.95 2.95 2.69 2.68 2.67 2.66 2.65 2.65 2.65 2.63 2.63 2.58 2.58 2.56 2.56 2.54 2.54 2.53 2.52 1.50 1.27 1.25
2.00 3.00 0.96 1.00 2.00 2.00 1.00 1.00 1.00 1.89 3.00
f1 (ppm)

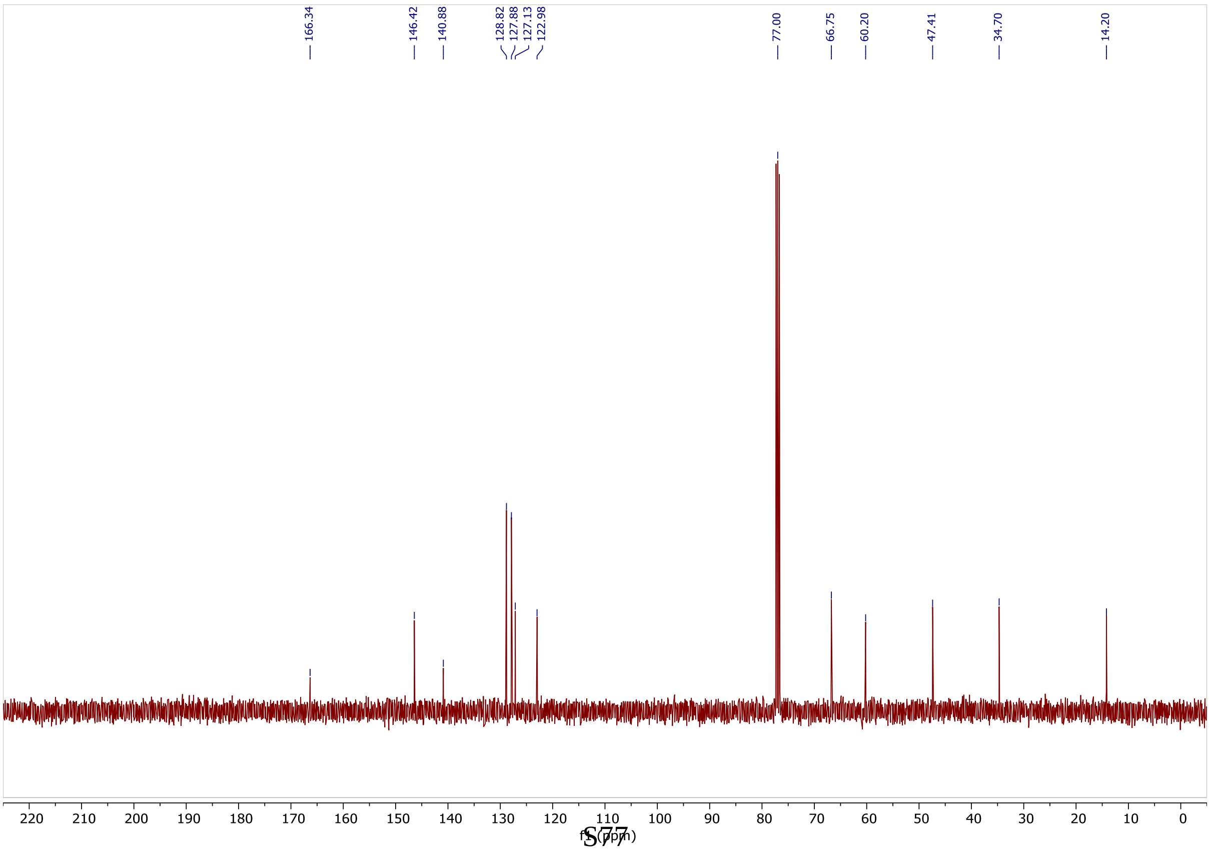

166.34
146.42
140.88
128.82
127.88
127.13
122.98
77.00
66.75
60.20
47.41
34.70
14.20
f1 (ppm)

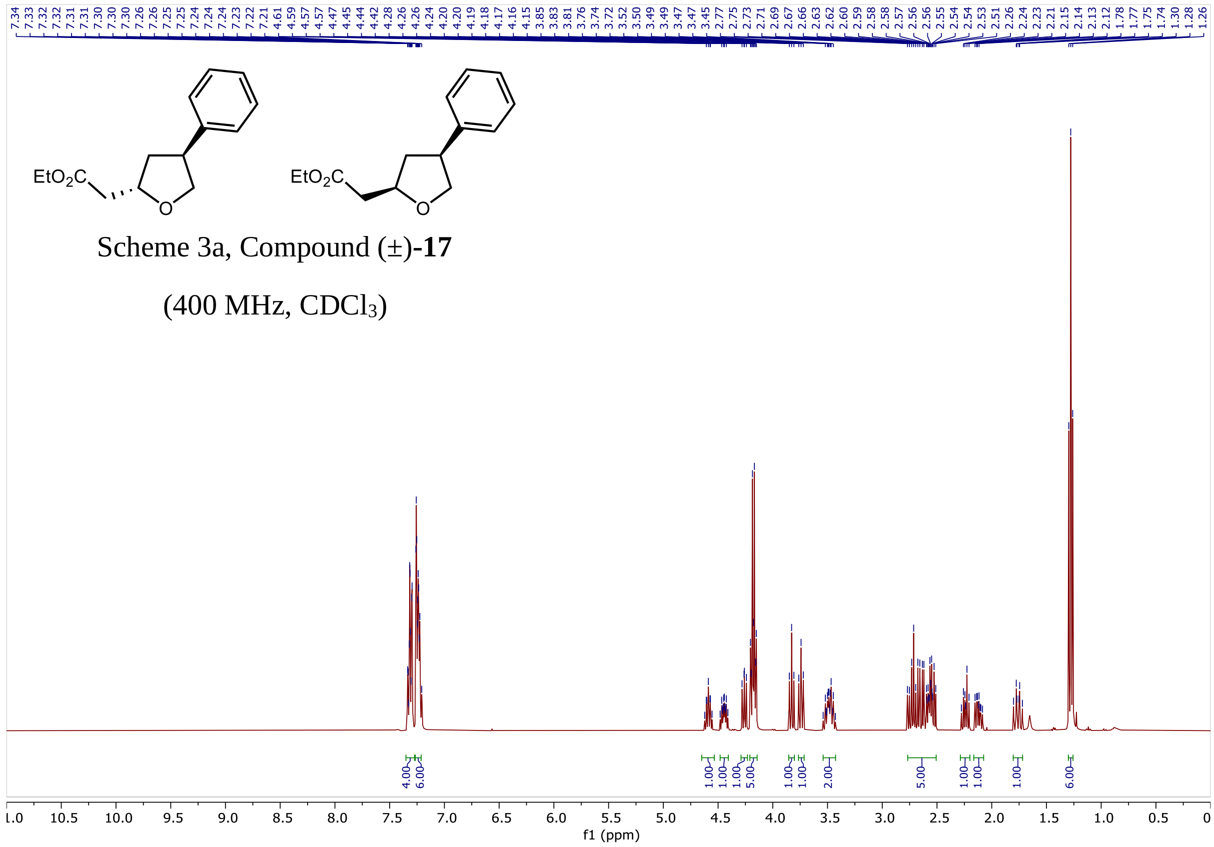
EtO2C
Scheme 3a, Compound (±)-17
(400 MHz, CDCl3)
f1 (ppm)

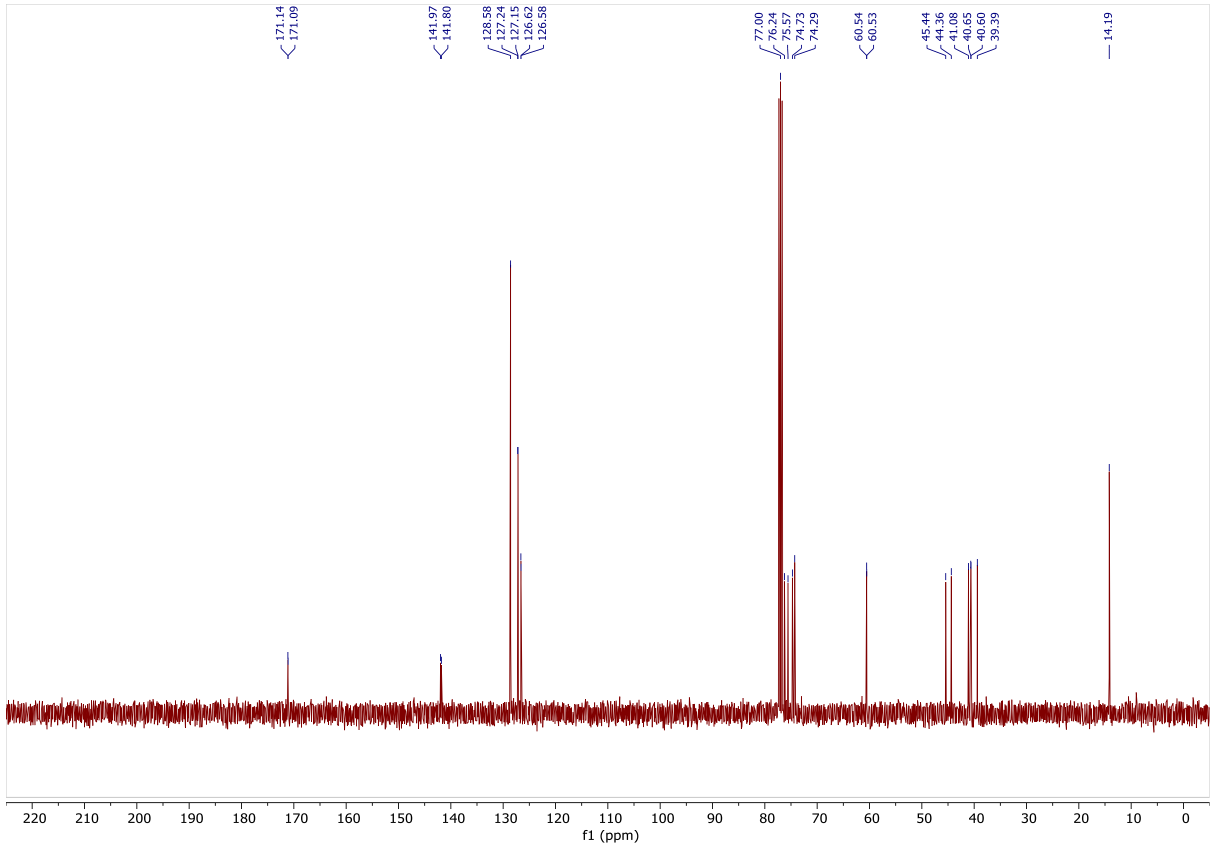
f1 (ppm)

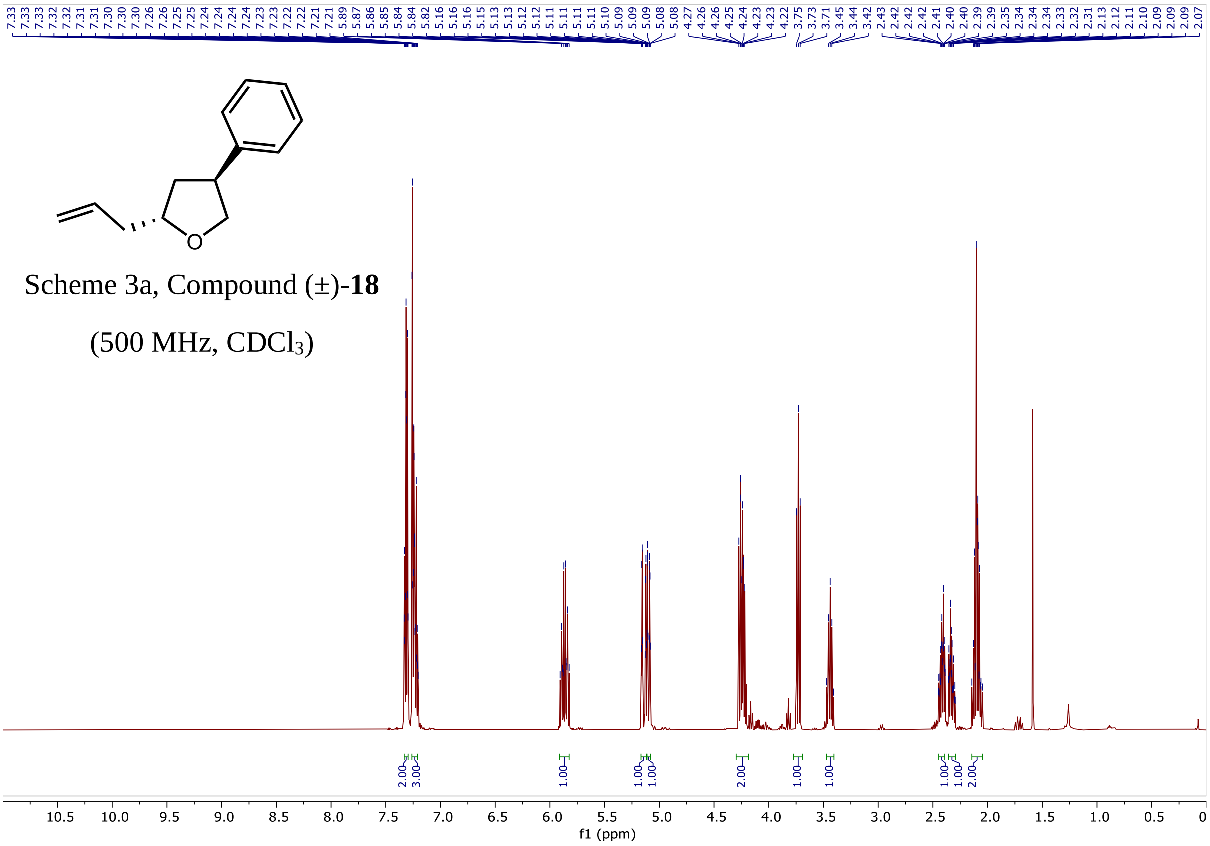
Scheme 3a, Compound (±)-**18**
(500 MHz, $CDCl_3$)
2.00
3.00
1.00
1.00
1.00
2.00
1.00
1.00
1.00
1.00
2.00
10.5 10.0 9.5 9.0 8.5 8.0 7.5 7.0 6.5 6.0 5.5 5.0 4.5 4.0 3.5 3.0 2.5 2.0 1.5 1.0 0.5 0
f1 (ppm)

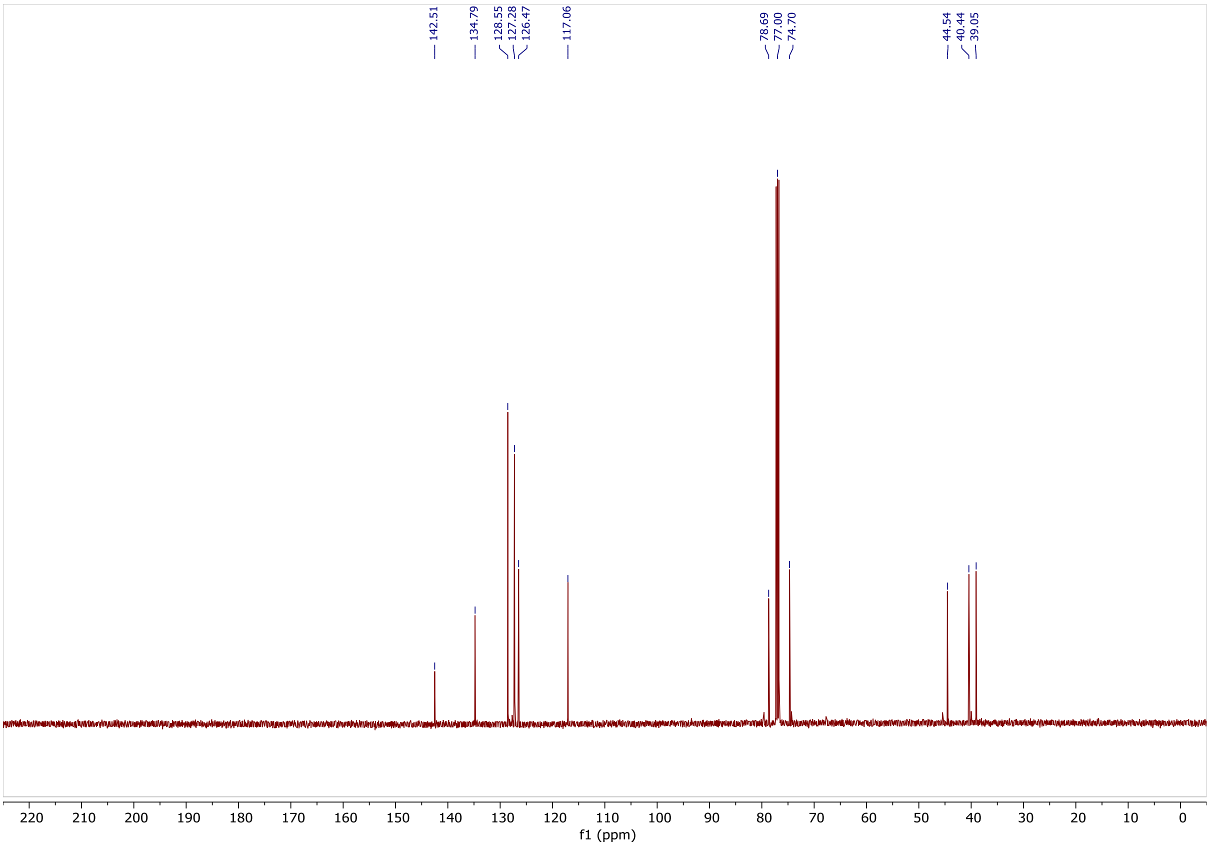
142.51
134.79
128.55
127.28
126.47
117.06
78.69
77.00
74.70
44.54
40.44
39.05
220 210 200 190 180 170 160 150 140 130 120 110 100 90 80 70 60 50 40 30 20 10 0
f1 (ppm)

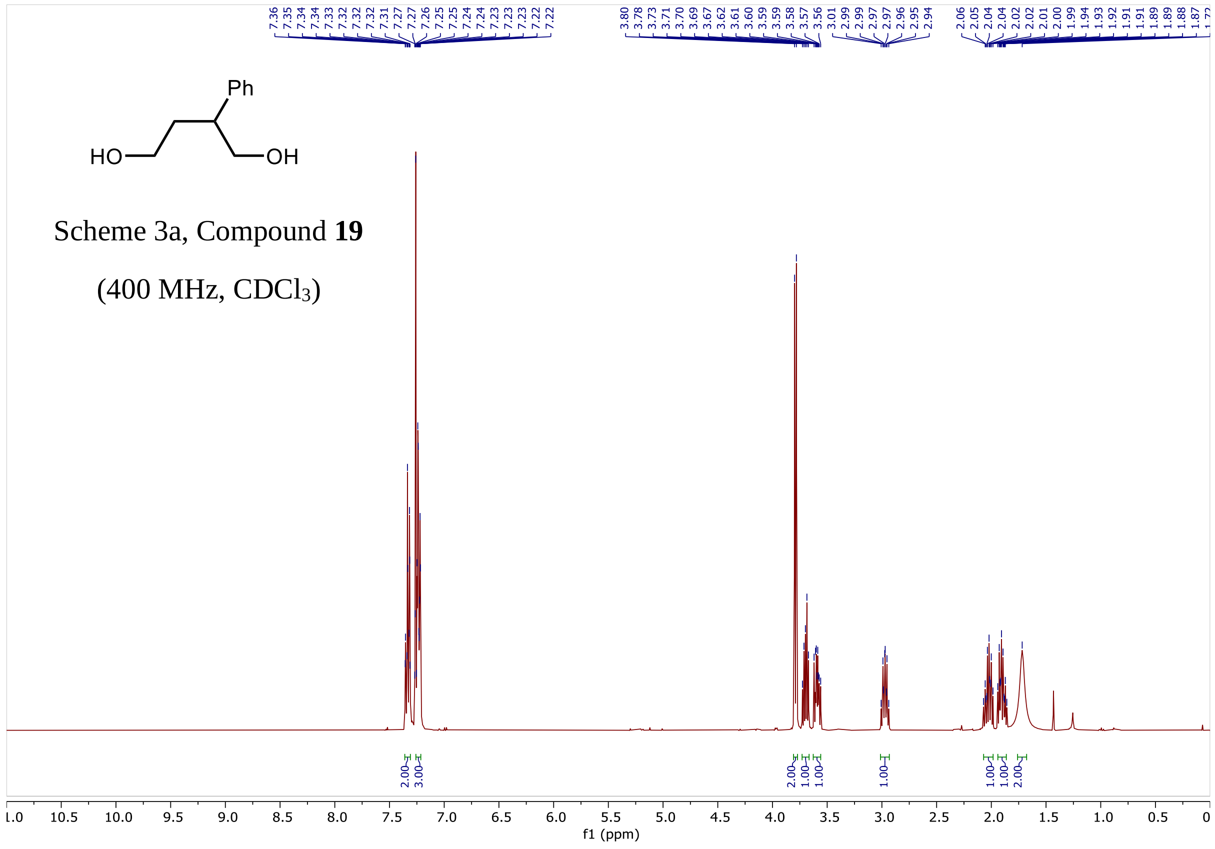

Ph
HO
OH
Scheme 3a, Compound **19**
(400 MHz, $CDCl_3$)
7.36 7.35 7.34 7.34 7.33 7.32 7.32 7.32 7.31 7.27 7.27 7.26 7.25 7.25 7.24 7.24 7.23 7.23 7.23 7.22 7.22
3.80 3.78 3.73 3.71 3.70 3.69 3.67 3.62 3.61 3.60 3.59 3.59 3.58 3.57 3.56
3.01 2.99 2.99 2.97 2.97 2.96 2.95 2.94
2.06 2.05 2.04 2.04 2.02 2.02 2.01 2.00 1.99 1.94 1.93 1.92 1.91 1.91 1.89 1.88 1.87
2.00 3.00 2.00 1.00 1.00 1.00 1.00 1.00 2.00
f1 (ppm)

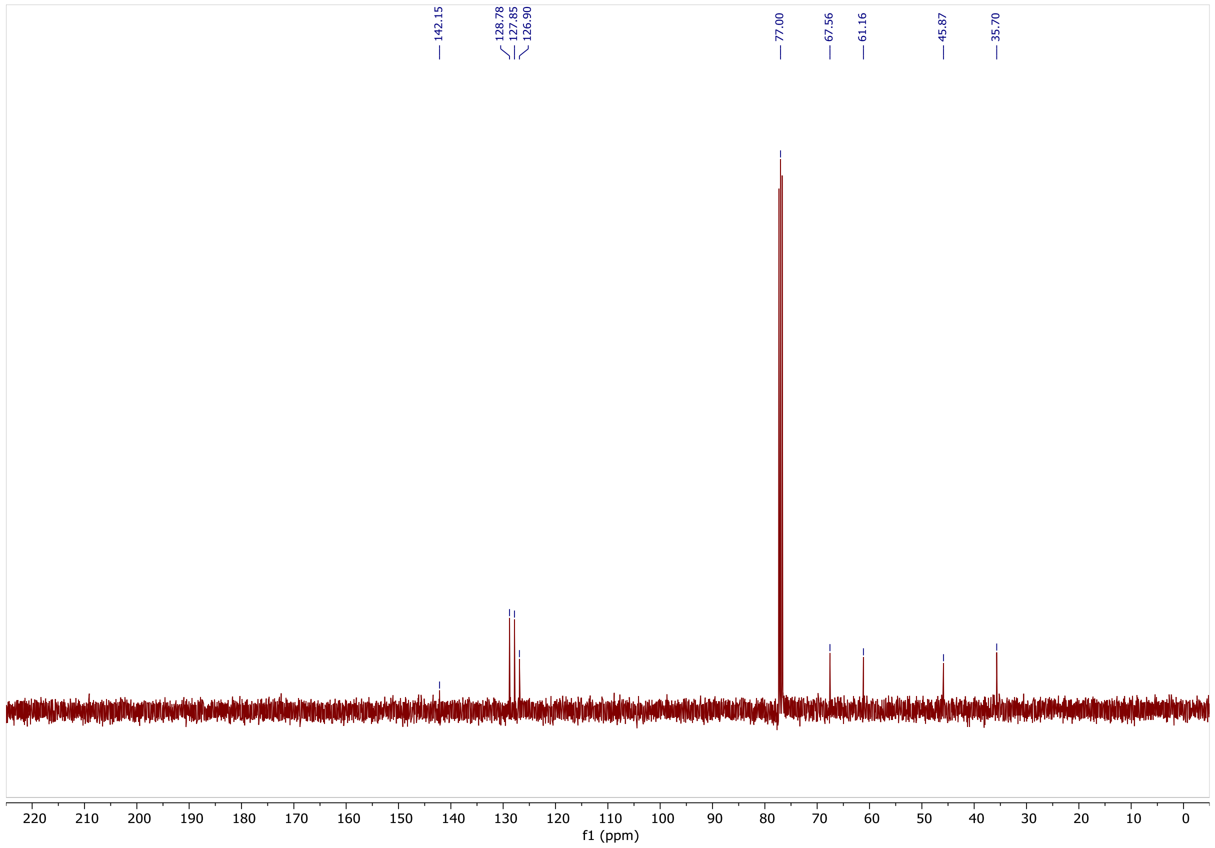

142.15
128.78 127.85 126.90
77.00
67.56
61.16
45.87
35.70
f1 (ppm)

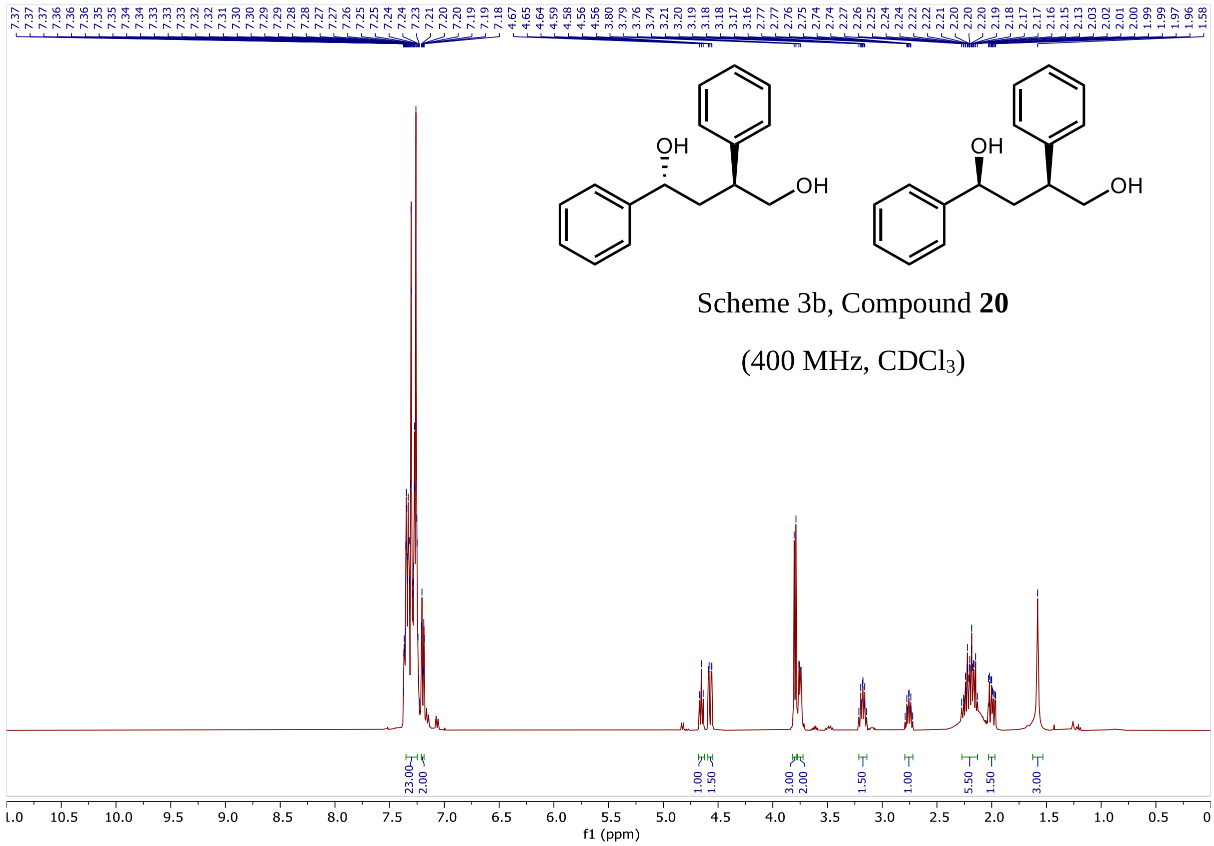
OH
OH
OH
OH
Scheme 3b, Compound 20
(400 MHz, CDCl3)
f1 (ppm)

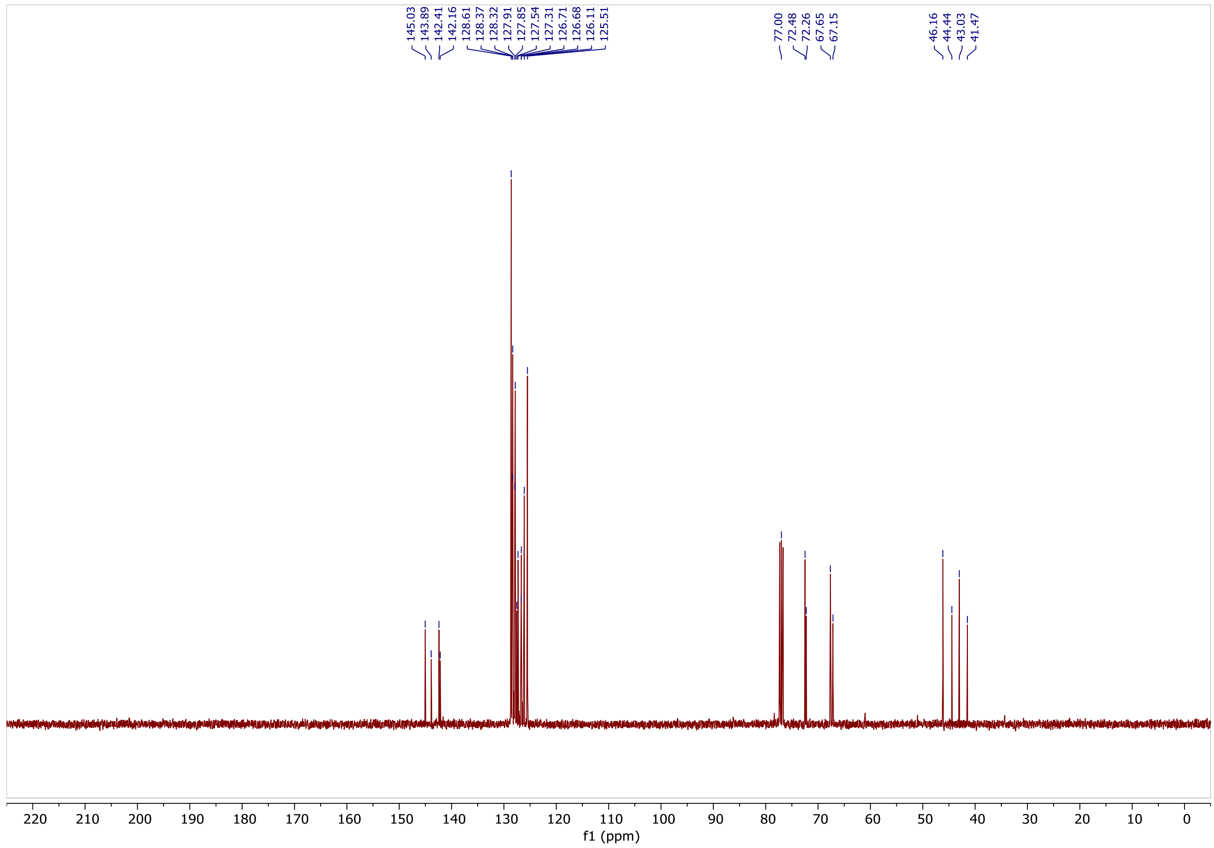
f1 (ppm)

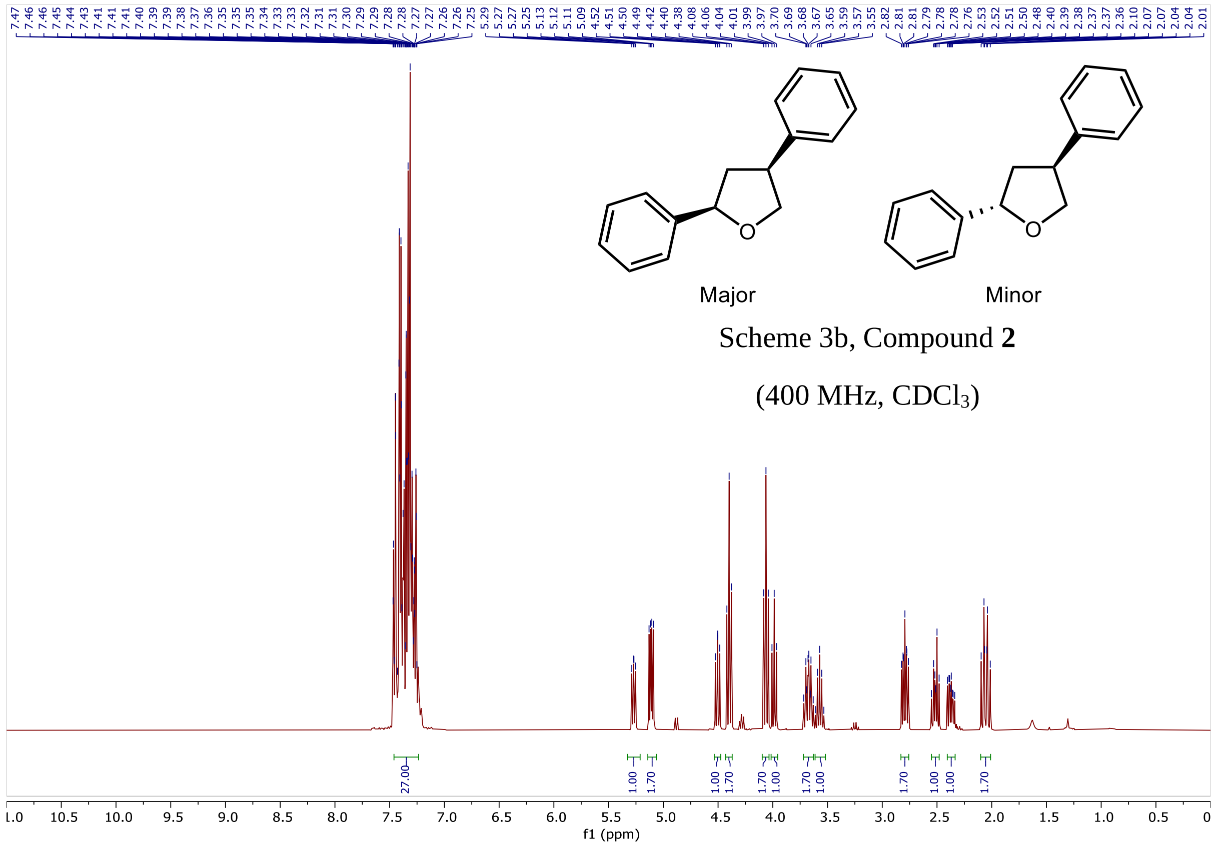
Major
Minor
Scheme 3b, Compound 2
(400 MHz, CDCl3)
f1 (ppm)

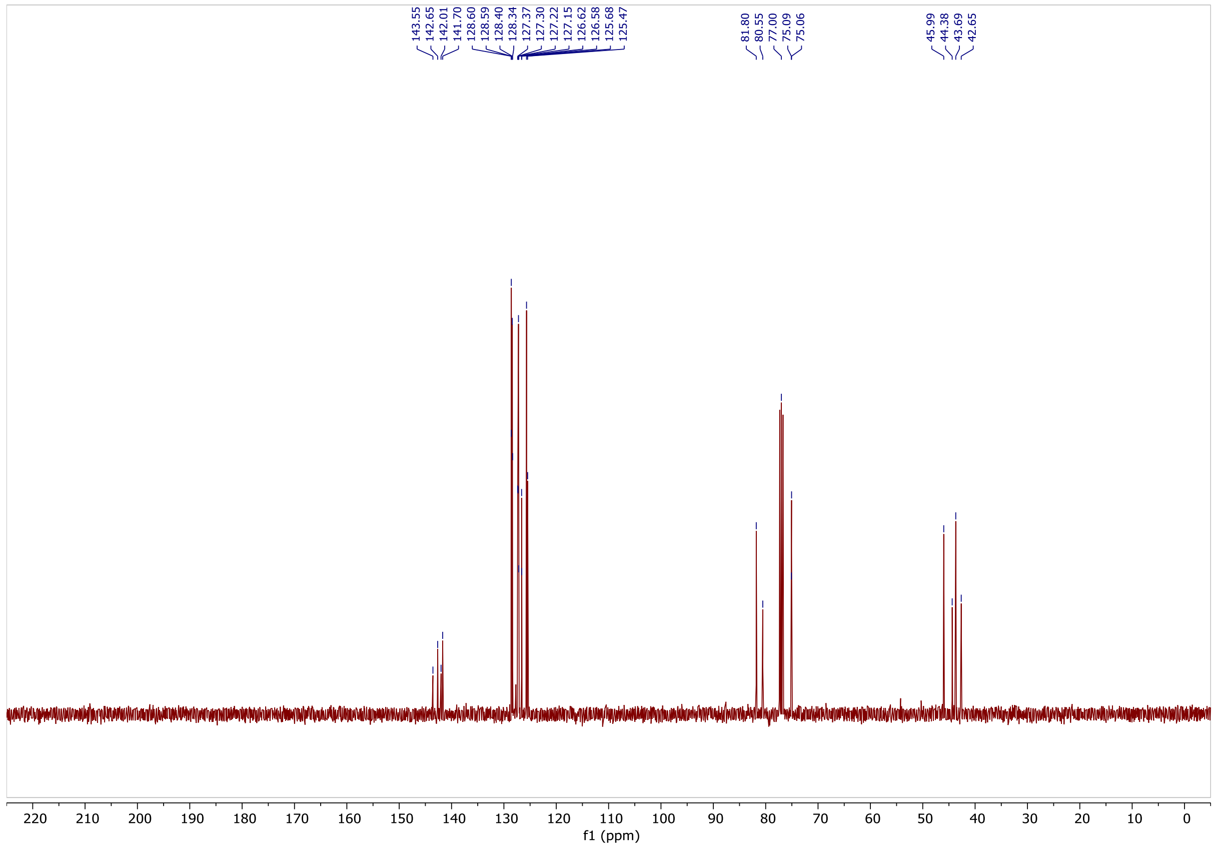
f1 (ppm)

Scheme 3b, Compound **21b**

(400 MHz, $CDCl_3$)

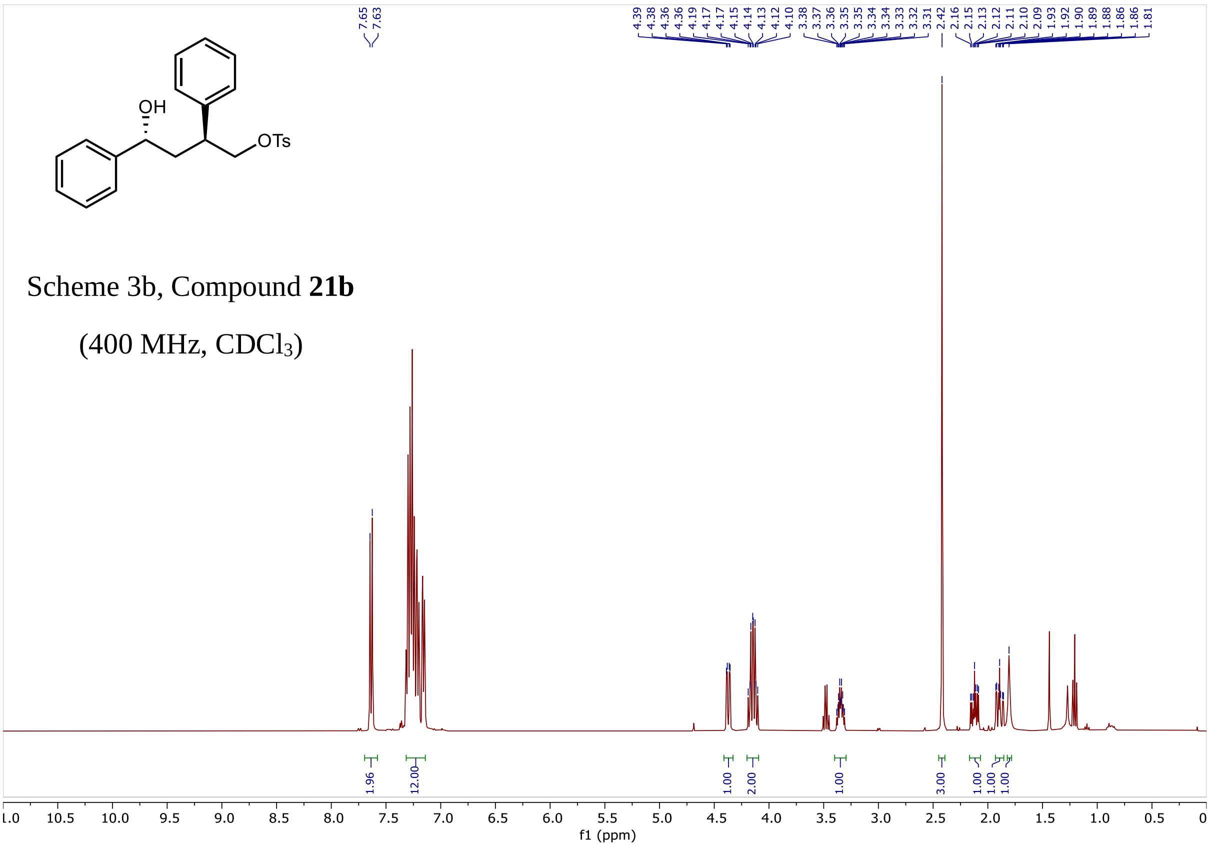

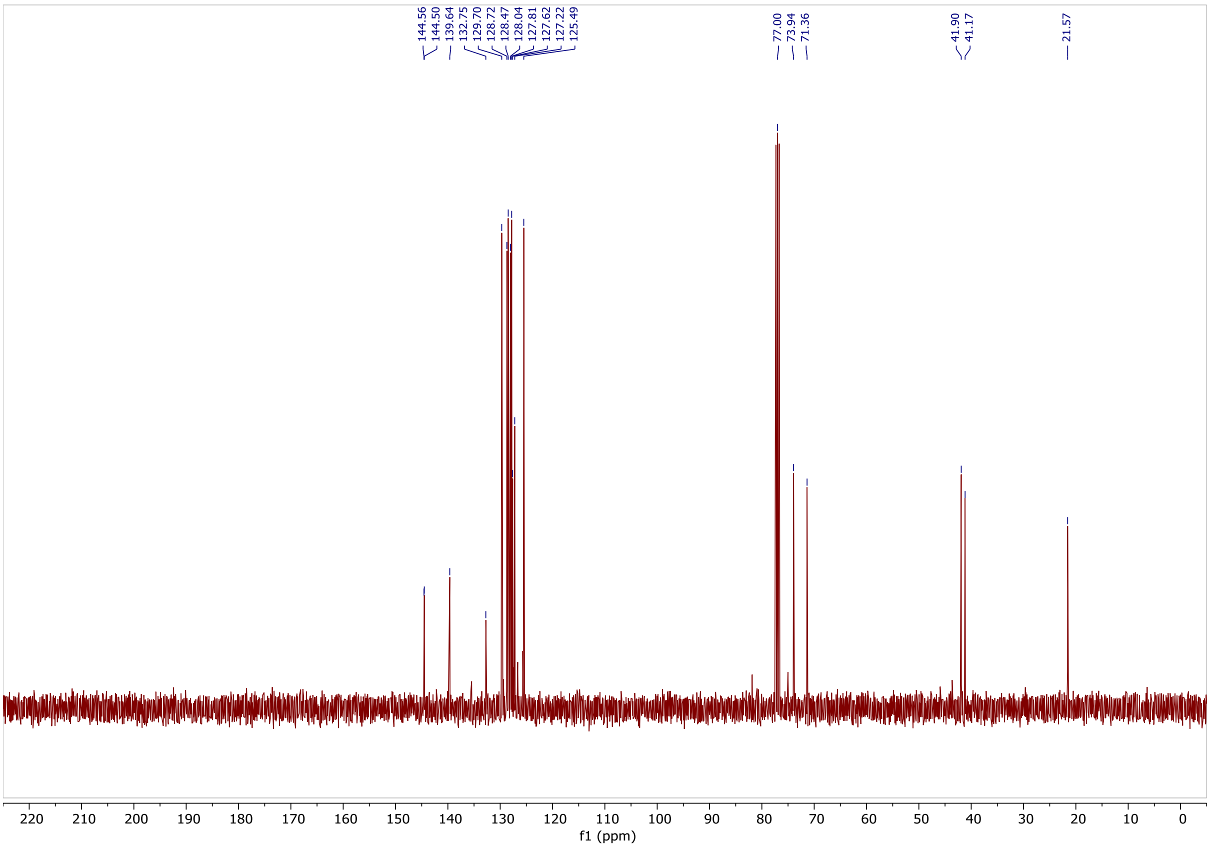

Supplement: Supplementary file 1 — ol3c00769_si_001.pdf [file ol3c00769_si_001.pdf]
